# Deciphering the single-cell transcriptome network in keloids with intra-lesional injection of triamcinolone acetonide combined with 5-fluorouracil

**DOI:** 10.3389/fimmu.2023.1106289

**Published:** 2023-05-19

**Authors:** Yijun Xia, Youbin Wang, Yan Hao, Mengjie Shan, Hao Liu, Zhengyun Liang, Xinwen Kuang

**Affiliations:** ^1^ Department of Plastic Surgery, Peking Union Medical College Hospital, Chinese Academy of Medical Sciences, Peking Union Medical College, Beijing, China; ^2^ Department of Plastic Surgery, Peking Union Medical College Hospital, Beijing, China

**Keywords:** triamcinolone acetonide, 5-fluorouracil, keloid, scRNA, transcriptome

## Abstract

**Objectives:**

Keloid is a highly aggressive fibrotic disease resulting from excessive extracellular matrix deposition after dermal injury. Intra-lesional injection of triamcinolone acetonide (TAC) in combination with 5-fluorouracil (5-FU) is a commonly used pharmacological regimen and long-term repeated injections can achieve sustained inhibition of keloid proliferation. However, the molecular mechanisms underlying the inhibitory effect on keloids remain insufficiently investigated.

**Methods and materials:**

This study performed single-cell RNA sequencing analysis of keloids treated with TAC+5-FU injections, keloids, and skins to explore patterns of gene expression regulation and cellular reprogramming.

**Results:**

The results revealed that TAC+5-FU interrupted the differentiation trajectory of fibroblasts toward pro-fibrotic subtypes and induced keloid atrophy possibly by inhibiting the FGF signaling pathway in intercellular communication. It also stimulated partial fibroblasts to develop the potential for self-replication and multidirectional differentiation, which may be a possible cellular source of keloid recurrence. T cell dynamics demonstrated elevated expression of secretory globulin family members, which may be possible immunotherapeutic targets. Schwann cell populations achieved functional changes by increasing the proportion of apoptotic or senescence-associated cell populations and reducing cell clusters that promote epidermal development and fibroblast proliferation.

**Conclusions:**

Our findings elucidated the molecular and cellular reprogramming of keloids by intra-lesional injection of TAC+5-FU, which will provide new insights to understand the mechanism of action and therapeutic targets.

## 1 Introduction

Keloids, caused by an imbalance of extracellular matrix deposition and autophagy during wound healing, are a group of fibrotic skin diseases characterized by invasion into the surrounding skin and uncontrolled proliferation (1–3). The appearance is that of a hard pathologic scar that protrudes from the skin and manifests microscopically as dense, swirling or irregularly arranged bundles of collagen fibers (4). A variety of factors, including race, local tension, immunity, and hypoxia, have been reported to be associated with the development of keloids, but no theory exists that can independently and adequately elucidate the pathogenesis of keloids (5–7). Owing to their severe aesthetic impact and uncomfortable symptoms like itching and soreness, keloids impose a heavy physical and psychological burden on patients.

Triamcinolone acetonide (TAC) is the most commonly used synthetic glucocorticoid for the treatment of keloid scars, with vasoconstrictive, anti-inflammatory and immunosuppressive effects (8). TAC inhibits inflammatory responses in wound healing by inhibiting leukocyte and monocyte migration and phagocytosis, and induces fibroblast apoptosis by blocking transforming growth factor (TGF)-β1 expression (9, 10). 5-Fluorouracil (5-FU) prevents the synthesis of pyrimidine thymidine, which is essential for DNA replication, and its scarcity leads to death in rapidly dividing cells. *In vitro* studies have demonstrated that 5-FU is effective in preventing the synthesis of collagen (11, 12). Combination injections of these two drugs can effectively inhibit fibroblast proliferation and degrade total collagen. Moreover, 5-FU+TAC has been reported to significantly diminish the retention of CD103 memory T cells locally and infiltration of CD 8 T cells in the skin (13). Multiple, long-term repeated injections can achieve sustained inhibition of keloid proliferation (14, 15). However, the key mechanisms by which this treatment regimen inhibits keloid growth and reduces painful and pruritic symptoms are still not well understood.

Single-cell RNA sequencing (scRNA-seq) allows unbiased recording of transcriptional profiles with unprecedented resolution by sequencing mRNA from individual cells, bringing a data-driven innovation in understanding the mechanisms of disease (16, 17). The application of single-cell sequencing to detect post-treatment keloids is instructive for understanding the gene expression changes and biomolecular mechanisms by which pharmacotherapy inhibits keloid growth. In this study, we performed scRNA-seq analysis in keloid tissues after TAC+5-FU injection treatment to learn more about the altered program and microenvironment composition, which contributes to the resolution of potential mechanisms of TAC+5-FU effects on keloids at the cellular level.

## 2 Method

### 2.1 Sample acquisition

This study was approved by the Medical Ethics Committee of Peking Union Medical College Hospital (JS-2907). Two keloid patients who had been treated with TAC + 5-FU injections for 1 month at other hospitals and voluntarily requested surgical excision because of the aesthetic impact of the atrophic lesions were recruited into the post-treatment keloid group (PK). The combination treatment drug was made by mixing 0.6 mL of 2.5% 5-FU, 5 mL of 1% TAC, and 1 mL of 2% lidocaine (one intra-lesion injection). 2 patients with untreated keloids had keloid tissue and normal skin tissue within 5 cm of the lesion surgically collected (18). All collected lesions were taken from the chest region. The four patients included were all female, with a mean age of 25.25 years, and all had a disease duration of more than 3 years. Exclusion criteria were as follows (1): patients with severe systemic or immune diseases (2); patients who had undergone previous surgery, radiotherapy, laser, or cryotherapy for keloids (3); patients with lesions that were infected or ulcerated (4); patients with skin diseases; and (5) women who were pregnant or lactating.

Fresh specimens were washed with saline and then placed in sCelLiveTM tissue preservation solution (Singleron Bio Com, Nanjing, China) for freezing within 30 minutes after surgery. Chopped specimens were digested with Singleron PythoN™ Automated Tissue Dissociation System (Singleron Bio Com, Nanjing, China) for 15 min, followed by removal of erythrocytes using 2 ml of GEXSCOPE^®^ Erythrocyte Lysis Solution (Singleron Bio Com, Nanjing, China). Cell viability was assessed by microscopy and cell counts were performed on the prepared cell suspensions to ensure a concentration of 1 × 10^5^ cells/ml.

### 2.2 Single cell RNA sequencing

Libraries were constructed from prepared cell suspensions according to the protocol of GEXSCOPE^®^ Single Cell RNA Library Kits (Singleron Bio Com, Nanjing, China) and sequenced on the Illumina HiSeq X platform (19). Raw reads were completed using CeleScope v1.3.0 to construct a human reference genome to generate a gene expression matrix (https://github.com/singleron-RD/CeleScope). The expression matrix was filtered using Scanpy v1.8.2 to complete quality control by the following criteria: 1) exclusion of cells with less than 200 genes or greater than 50% mitochondrial content; 2) exclusion of cells with the highest 2% of UMI counts or gene counts; and 3) exclusion of genes expressed in less than 5 cells (20). Batch effects between samples were removed. After normalization of the expression matrix, the top 2000 were selected as highly variable genes for scaling the matrix. The top 20 principal components obtained after principal component analysis were selected and the aggregated cell clusters were visualized by Uniform Manifold Approximation and Projection (UMAP) by downscaling and clustering the data using the Louvain algorithm. Based on the SynEcoSys database (Singleron Bio Com, Nanjing, China), the cell type identity of each cluster was defined by the expression of typical markers identified in each cell cluster for differential gene discovery. After visualization of the annotated cell clusters, keratinocytes accounted for more than half of the total number of cells. As keloids are caused by excessive deposition of extracellular matrix in the dermis, the effect of keratinocytes was removed from the visualisation of cell proportions in order to provide a clearer view of the changes in fibroblasts and other major functional cell populations. Based on the nonparametric Wilcoxon likelihood ratio test, genes with a P value less than 0.05 and average log2(Fold Change) of greater than 0.25 were selected as differentially expressed genes.

### 2.3 Enrichment analysis

To predict the biological functions of differentially expressed genes (DEGs) and to reveal differences in molecular mechanisms between different cell clusters, potential pathways and biological processes of DEGs enrichment were evaluated by the “clusterProfiler” package and using Gene Ontology (GO) and Kyoto Encyclopedia of Genes and Genomes (KEGG) analysis (21). Using the GSVA package, the average gene expression of the target cell type was used as input data to assess the enrichment of different pathways across different cell clusters (22).

### 2.4 Trajectory analysis and RNA velocity

To map the respective differentiation trajectories between fibroblast clusters and Schwann cell clusters, a pseudo-time trajectory analysis was performed with Monocle2 (23). Transition trajectories between different differentiation states of cells were constructed based on the first 2000 highly variable genes identified, and the DDRTree algorithm was used to achieve descending and cell sorting of the data. Dependent genes that changed significantly with pseudo-time were identified by the DifferentialGeneTest function and visualized as heat maps. Information on the targeted dynamics of the cells was inferred based on the observed ratio of spliced to unspliced mRNA using velocyto analysis in python, and the results were projected onto UMAP plots (24).

### 2.5 Intercellular communication

CellPhoneDB v2.1.0 (1000 iterations) was employed to predict differences in cell-cell interactions between keloid after treatment, keloid and normal skin based on a database of known ligand receptors, with a focus on changes in fibroblast-cell cell interactions. Heatmaps and bubble plots were presented to demonstrate the number of pairs of interactions and ligand-receptor pairs between different cell types (25).

### 2.6 Transcription factor regulatory network analysis

After searching for genes encoding transcription factors (TFs) in the AnimalTFDB database (http://bioinfo.life.hust.edu.cn/AnimalTFDB/), transcription factor networks were constructed by pySCENIC using expression matrixes of fibroblast cell populations (26). Potential targets of each transcription factor were predicted based on the co-expression of regulators and targets, and regulator activity was quantified by AUCell and both active and inactive regulators were identified.

### 2.7 Immunohistochemical staining

Excised keloid specimens were immediately fixed in 4% paraformaldehyde and subjected to conventional gradient dehydration, wax immersion, and paraffin embedding on day 2. Paraffin sections were sequentially dewaxed, hydrated, and antigen repaired. After blocking endogenous peroxidase activity with hydrogen peroxide for 10 minutes, tissue isolates were incubated with primary antibodies overnight at 4°C. Anti-Ki-67 (1:100, Abcam, Cat#: ab15580), anti-BAX (1:200, Abcam, Cat#: ab32503) were used for immunohistochemistry. Secondary antibodies were then applied. 3,3’-diaminobenzidine solution was added for staining development. Finally, neutral resin blocking, microscopic observation and photography were performed.

### 2.8 Immunofluorescence

Paraffin sections were deparaffinized and antigen retrieval was completed. Sections were placed in 3% hydrogen peroxide solution and incubated for 25 minutes at room temperature to block endogenous peroxidase. The sections were then serum blocked. The blocking solution was removed and anti-human Osteopontin antibody (Abcam, Cat#: ab8448) and anti-Fibronectin antibody (Abcam, Cat#: ab2413) were added dropwise and incubated flat in a moist box at 4°C overnight. The sections were gently shaken and dried, and the appropriate fluorescent secondary antibody was added dropwise, and DAPI was used to re-stain the nuclei of the cells. Fluorescence blocker was applied to seal the sections.

## 3 Results

### 3.1 ScRNA-seq reveals the cellular landscape of keloids after TAC+5-FU intra-lesional injection treatment

**Figure 1:**
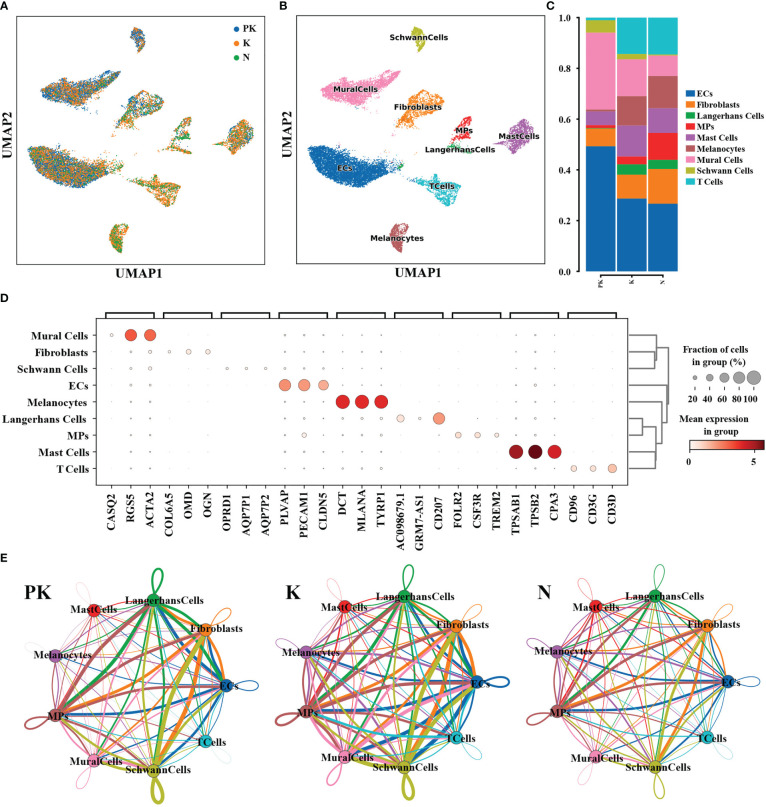
Dissection of keloids treated with TAC+5-FU injection (n=2), keloids (n=2), and keloid-adjacent skins (n=2) by scRNA-seq. **(A)** UMAP plots of the 3 groups of cells after dimensional reduction clustering. Single-cell sequencing of the 3 groups yielded a total of 93,190 cells, and cluster analysis after removal of keratinocytes in the epidermis yielded a total of 18,605 cells. **(B)** The UMAP plot of 9 cell subtypes. **(C)** Proportion of cell lineages in keloids treated with TAC+5-FU injection (PK), keloids (K) and normal skins (N). **(D)** The bubble plot of differentially expressed genes. For each cluster, the top 3 genes and their relative expression levels in all sequenced cells were displayed. **(E)** Interaction network plots between all cell types in keloids treated with TAC+5-FU injection (PK), keloids (K) and normal skins (N), where network edge thickness indicates the total number of ligand-receptor pairs and line color is consistent with ligand cell type. UMAP, uniform manifold approximation and projection; TAC, triamcinolone acetonide; 5-FU, 5-fluorouracil; ECs, endothelial cells; MPs, mononuclear phagocytes.

To dissect the regulation of transcriptional profiles of keloids by TAC+5-FU, we performed single-cell RNA sequencing on 2 post-treatment keloids (PK group), 2 keloids without any therapy (K group), and 2 adjacent normal skins (N group). A total of 14,443 transcriptome data from cells in the PK group, 39,494 from cells in the K group and 39,253 from cells in the N group were obtained. After visualization of the annotated cell populations, keratinocytes accounted for the majority of the total cell population. Considering that keloids are caused by excessive deposition of extracellular matrix in the dermis, the effect of keratinocytes was excluded from the downstream analysis in order to visualize more clearly the changes in fibroblasts and other major functional cell populations. Cell clusters were characterized using well-established cellular markers, and a total of 9 cell types were identified, namely endothelial cells, fibroblasts, Langerhans cells, mononuclear phagocytes, mast cells, melanocytes, mural cells, Schwann cells, and T cells (**Figures 1A, B**). The genes specifically expressed in each cell type as illustrated in **Figure 1D**; **Figure S1**. Subsequently, we analyzed the ratio of cell lineages between the three groups, with fibroblasts, mast cells, mononuclear phagocytes, Langerhans cells, melanocytes and T cells being reduced in the PK group compared to the K group (**Figure 1C**). In addition, the expression level of intercellular interaction signals was more abundant between the K group than the other two groups, especially between fibroblasts and T cells (**Figure 1E**). Overall, TAC+5-FU injection treatment not only altered the cellular composition of keloids, but also impacted the level of intercellular communication.

### 3.2 The differentiation trajectory and intercellular interactions of fibroblasts are reshaped

**Figure 2:**
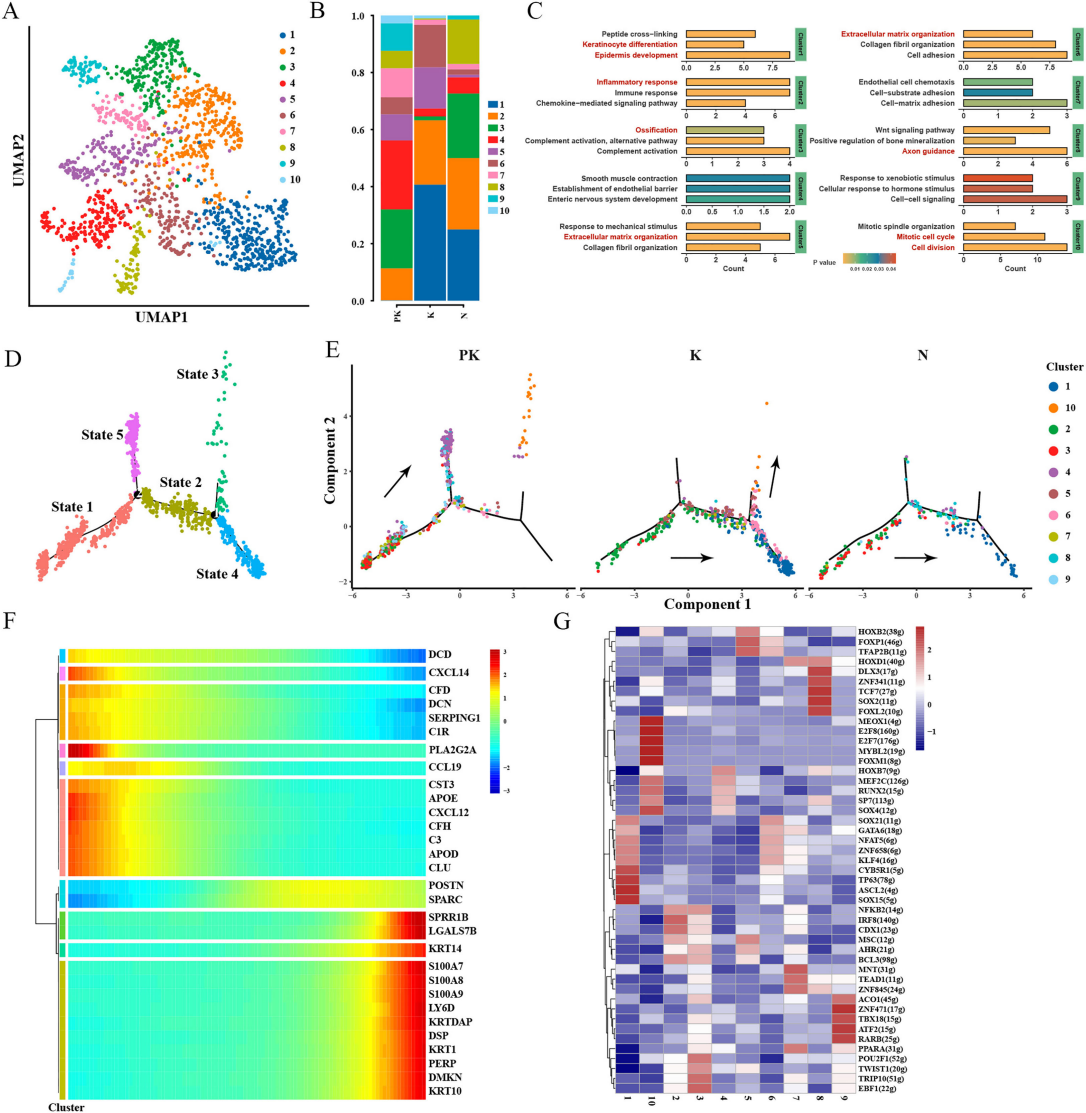
Single-cell transcriptome of fibroblasts. **(A)** The UMAP plot of fibroblasts, each cell with a color code of its cell cluster origin. **(B)** Histogram illustrating the proportion of fibroblast clusters in keloids treated with TAC+5-FU injection (PK), keloids (K) and normal skins (N). **(C)** Functional enrichment of upregulated genes in each fibroblast cluster with significance threshold set at P value < 0.05. **(D)** Pseudo-time ordering of fibroblasts revealed branching trajectories and identified five differentiation states. **(E)** Cell fate transitions of fibroblasts in post-treated keloid (left panel), keloid (middle panel), and normal skin (right panel), with colors indicating fibroblast clusters. **(F)** Hierarchical clustering of branching-dependent genes revealed 10 gene modules and displayed the expression of representative genes for each gene module over pseudo-time. **(G)** Activity of the major regulators identified by pySCENIC in fibroblast clusters. Heatmap showing the clustering of regulator AUC matrices in each cell cluster at the mean value. Horizontal coordinates represent fibroblast clusters, vertical coordinates are regulon names, and numbers in parentheses represent the number of target genes of transcription factors. UMAP, uniform manifold approximation and projection; AUC, area under the curve.

Since excessive proliferation of fibroblasts is one of the principal mechanisms of keloid formation, we proceeded to perform descending clustering of fibroblast populations and detected a total of 10 clusters (**Figures 2A, B**). To determine the specific functions of the different clusters, we performed enrichment analysis of upregulated genes (**Figure 2C**) and examined the expression of markers (**Figure S2A**) for the four identified fibroblast subpopulations (mesenchymal fibroblasts, pro-inflammatory fibroblasts, secretory-papillary fibroblasts and secretory-reticular fibroblasts) (27). Cluster 1 was characterized by the expression of genes associated with epidermal development (**Figure 2C**) including KRTDAP, KRT1, and KRT10 (**Figure S2B**). Expression of pro-inflammatory fibroblast markers (CCL19, CXCL2, CXCL3) was concentrated in cluster 2 (**Figure S2A**), where the upregulated genes were significantly associated with inflammatory response (**Figure 2C**). Secretory-reticular fibroblast markers (WISP2, SLPI, TSPAN8) were predominantly expressed in cluster 3, and secretory-papillary fibroblast markers (APCDD1, ID1, WIF1) were mostly expressed in cluster 8 (**Figure S2A**). Mesenchymal fibroblast markers ASPN and POSTN were mainly expressed in clusters 5 and 6, and the results of functional enrichment analysis of clusters 5 and 6 showed correlation with extracellular matrix tissue and collagen fibril tissue (**Figure 2C**). The proportion of cluster 1, 2, 5, and 6 was decreased in the PK group compared with keloids (**Figures 2A, B**), suggesting a reduced composition of pro-epidermal, pro-inflammatory, and mesenchymal-type fibroblast clusters. The results were consistent with clinical observations that keloids treated with TAC+5-FU experienced local remission of inflammation and atrophy of the dermis and epidermis.

To further explore the differentiation trajectory underlying the variations in fibroblast proportions, we performed a pseudo-time analysis using Monocle2 and identified five states (**Figure 2D**). Fibroblasts in keloids followed a “state1-state2-state4” trajectory, with more fibroblasts differentiating towards mesenchymal clusters 5 and 6 than in normal skins (**Figure 2E**), accompanied by increased expression of KRT1, KRT10 (**Figure S2C**), and POSTN (**Figure 2F**). The fibroblasts in the PK group exhibited a novel direction of differentiation (from state 1 to state 5/state 3), and the tendency to differentiate toward mesenchymal type clusters 5 (state 2) and 6 (state 4) was suppressed, during which the expression of inflammatory response-related genes C3, CXCL12, and CXCL14 was reduced (**Figure S2C**). This was consistent with the results described above for changes in cell proportions. Fibroblasts in the PK group were more differentiated towards cluster 4 (state 5) and cluster 10 (state 3) (**Figure 2E**). Gene ontology enrichment analysis showed that genes involved in smooth muscle contraction, establishment of endothelial barrier, and enteric nervous system development were enriched in cluster 4 (**Figure 2D**), with a tendency of multidirectional differentiation and absence of function of promoting extracellular matrix formation. Cluster 10 was characterized by the expression of genes associated with mitosis and cell division. A low level of Ki67 expression was observed in keloids after treatment, which was almost absent in normal skin and more abundant in keloids. Increased expression of the pro-apoptotic gene BAX was observed in the PK group. Expression of extracellular matrix protein FN1 and bone matrix protein OPN was identified in keloids, whereas these proteins were reduced in the PK group (**Figure S3**). This suggests that TAC+5-FU injection stimulates fibroblasts to develop a potential for self-replication and multidirectional differentiation after causing local tissue damage to keloids, similar to the acute wound healing process.

Due to the importance of transcription factors (TFs) in regulating genomic DNA openness, modulating numerous immune responses, and developmental patterns (28, 29), we then inferred information on the regulation of TFs behind each fibroblast subtypes (**Figure 2G**). The results revealed that activation of HOXB7, MEF2C, RUNX2, SP7, SOX4 was observed in cluster 4. These transcription factor-regulated network modules were mainly involved in chondrocyte development (SFRP2, COL11A1, FGF18, RUNX2), cell differentiation (MEF2C, FOXD1, NDE1, MDK, SOX18, TCF4, RXRG, CAMK2G, ZFPM1, SOX4), neuron differentiation (NAPA, MEF2C, CCND1, NNAT, RUNX3, RXRG, HDAC9, RUNX2), T cell differentiation (DLL4, PTPRK, SOX4, RUNX2), which further corroborated the multidirectional differentiation potential of cluster 4 (**Table S1**).

**Figure 3:**
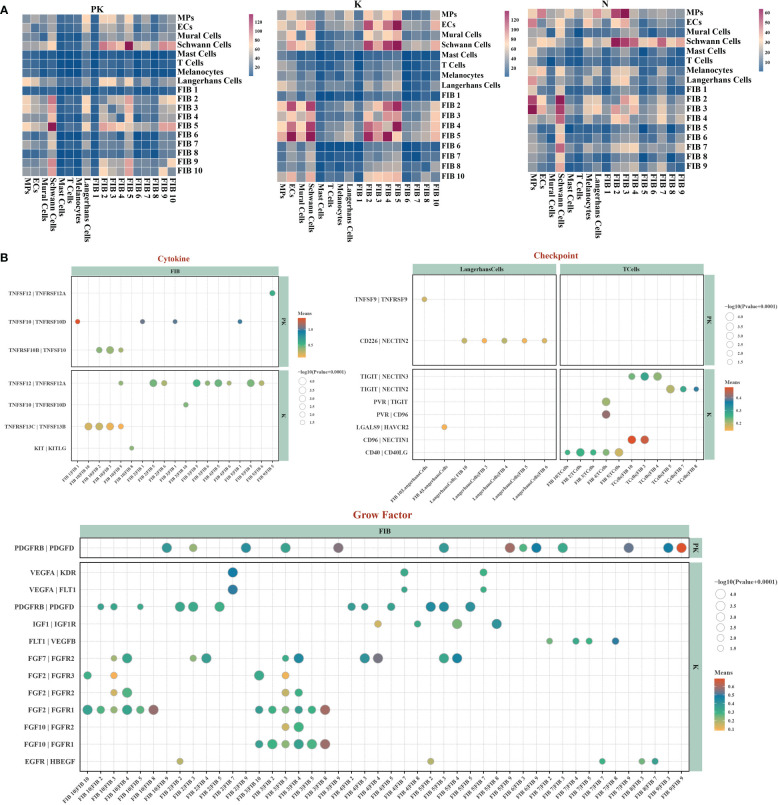
Single-cell transcriptome network of keloid after TAC+5-FU injection treatment. **(A)** Cell-cell communication networks of post-treated keloids (left panel), keloids (middle panel), and normal skins (right panel) were constructed using CellPhoneDB. Heatmap showing the numbers of inter-populations communications with each other in the three groups. **(B)** Bubble diagrams demonstrating the ligand-receptor interactions associated with cytokines, checkpoints and growth factors present in the keloid after TAC+5-FU injection treatment and keloid, respectively.

Intercellular interactions between fibroblasts and other cells within the microenvironment are a prerequisite for fibrosis formation (30, 31). Therefore, we constructed cell-cell communication networks for each of the three groups and observed the presence of a denser communication network in keloids, with partial intercellular communication in the PK group being weakened or even completely inhibited (**Figure 3A**), as in the case of checkpoint signaling (**Figure 3B**). The intercellular crosstalk in the PK group was primarily present between fibroblasts-fibroblasts. Notably, among the growth factor-related cellular interactions, cluster 10 and cluster 3 fibroblasts in keloids were the major cellular sources of FGF signaling (**Figure 3B**). FGF2, FGF7 and FGF10, as members of the fibroblast growth factor family, are involved in various biological processes, including embryonic development, cell growth, tissue repair, tumor growth and invasion, and have been reported to be critical targets in the fibrosis process (32, 33). TAC+5-FU significantly inhibited FGF signaling among fibroblasts.

Overall, the tissue damage caused by TAC+5-FU injection to keloids stimulated fibroblast differentiation toward cluster 10 with self-replicating potential and cluster 4 with multidirectional differentiation potential, interrupting the normal keloid differentiation trajectory of fibroblasts toward the pro-fibrotic subtype of clusters 5-6. The FGF signaling pathway was extensively inhibited in fibroblast interactions, which may be a potential pathway of action in keloid atrophy.

### 3.3 Regulation of immune transcriptional profiles

**Figure 4:**
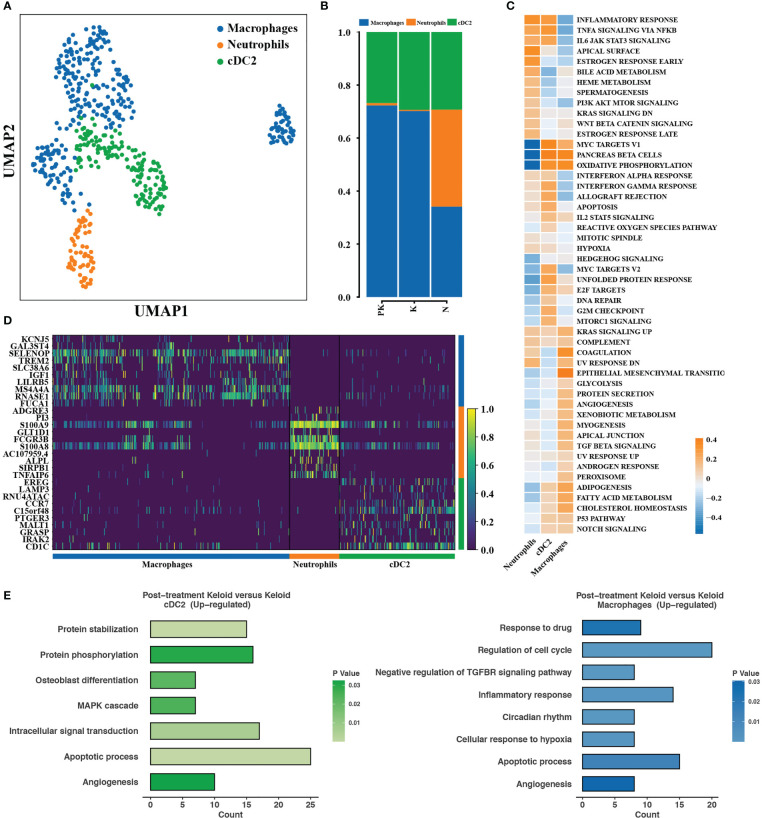
Transcriptional profiles of mononuclear phagocytes with changing characteristics. **(A)** Color-coded UMAP plots showing each immune cell subpopulation defined as: macrophages, cDC2, and neutrophils. **(B)** The percentage of mononuclear phagocyte subpopulations in post-treated keloids (PK), keloids (K), and normal skins (N) were displayed. **(C)** Differences in pathway activity between mononuclear phagocyte subpopulations calculated based on GSVA scores. **(D)** Heat map displaying representative differentially expressed genes between different types of mononuclear phagocyte subpopulations. **(E)** Functional enrichment of upregulated genes in cDC2 (left panel) and macrophages (right panel) with significance threshold set at P value < 0.05. UMAP, uniform manifold approximation and projection; GSVA, gene set variation analysis; cDC2, conventional dendritic cell subset 2.

Keloids are histologically composed of massive irregular infiltration of collagen fibers and inflammatory cells, and a complex immune microenvironment is essential in pro-fibrosis. We proceeded to resolve cellular heterogeneity and investigated changes in the transcriptional profiles of mononuclear phagocytes and T cells. Conventional dendritic cell type 2 (cDC2) and macrophages were the predominant cell populations in the PK and K groups, which did not differ significantly in proportion (**Figures 4A, B**). Macrophages were deactivated in inflammatory response, TNFα/NF-kB signaling pathway, IL6/JAK2/STAT3 signaling pathway, but upregulated epithelial mesenchymal transition, TGF beta signaling pathway (**Figure 4C**), indicating that macrophages had M2 type macrophage characteristics that suppressed inflammation and promoted tissue damage repair (34). cDC2 activated oxidative phosphorylation, inflammatory response, TNF α/NF-kB signaling pathway and upregulated the expression of genes related to inflammatory response and antigen presentation, including EREG, LAMP3, CD1c, and CCR7 (**Figures 4C, D**). cDC2 and macrophages in the PK group significantly upregulated the expression of genes involved in apoptosis and angiogenesis (**Figure 4E**).

**Figure 5:**
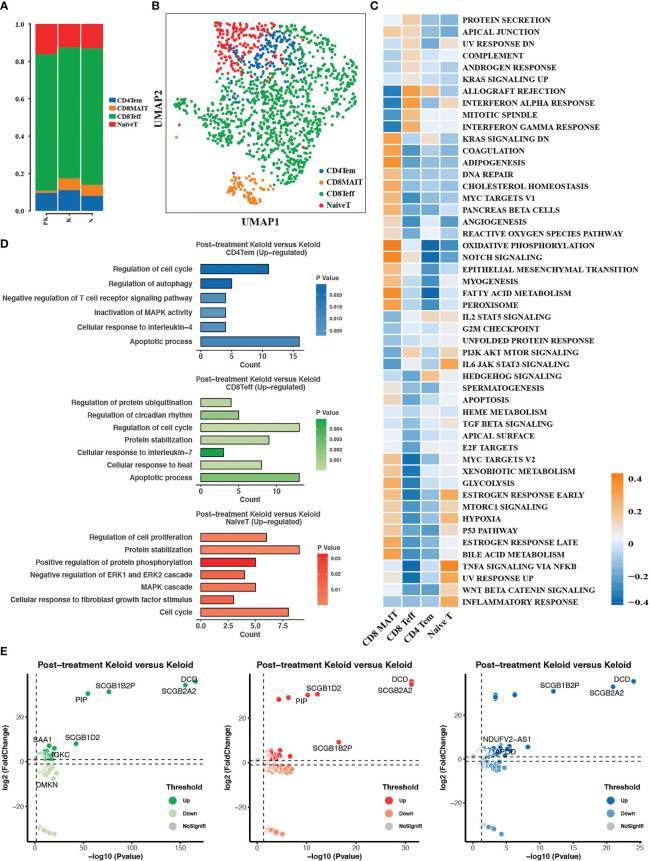
T cells were subdivided into different cell subpopulations. **(A)** The proportion of the 4 T cell subpopulations in post-treated keloid (PK), keloid (K) and normal skin (N) was displayed. **(B)** Descending clustering of T cells identified 4 distinct T cell subpopulations: naive T cells (NaiveT), CD4+ effector memory T cells (CD4Tem), CD8+ effector T cells (CD8Teff) and CD8+ mucosa-associated invariant T cells (CD8MAIT). **(C)** Heat map demonstrating differences in pathway activity between T cell subsets calculated based on GSVA scores. **(D)** Functional enrichment analysis of upregulated genes in CD4+ effector memory T cells, CD8+ effector T cells and CD8+ mucosa-associated invariant T cells. **(E)** Genes differentially expressed in CD4+ effector memory T cells, CD8+ effector T cells and CD8+ mucosa-associated invariant T cells in treated keloids compared to keloids with significance threshold set at P value < 0.05 and absolute value of log2 (Fold change) > 0.25. UMAP, uniform manifold approximation and projection; GSVA, gene set variation analysis.

Subclustering analysis of T cells identified four T cell subpopulations, comprising naive T cells, CD4+ effector memory T cells (CD4Tem), CD8+ effector T cells (CD8Teff), and CD8+ mucosa-associated invariant T cells (MAIT), with a reduced proportion of CD8+ mucosa-associated invariant T cells in the PK group (**Figures 5A, B**). MAIT cells were enriched in epithelial mesenchymal transition and hypoxia pathways (**Figure 5C**), and MAIT cells have been reported to be the major lymphocyte population producing IL-17A, capable of initiating immune response and fibrosis formation in an IL-1, IL-18, and antigen-dependent manner (35, 36). Three major T cell subpopulations in the PK group upregulated the expression of genes associated with regulation of cell cycle, protein stabilization, apoptotic process, and regulation of autophagy compared to untreated keloids (**Figure 5D**). In addition, the expression of immunomodulatory-related secretoglobin family members (SCGB2A2, SCGB1D2, SCGB1B2P) was upregulated in all three T cell subtypes (**Figure 5E**).

### 3.4 Behavioral changes in Schwann cell differentiation

**Figure 6:**
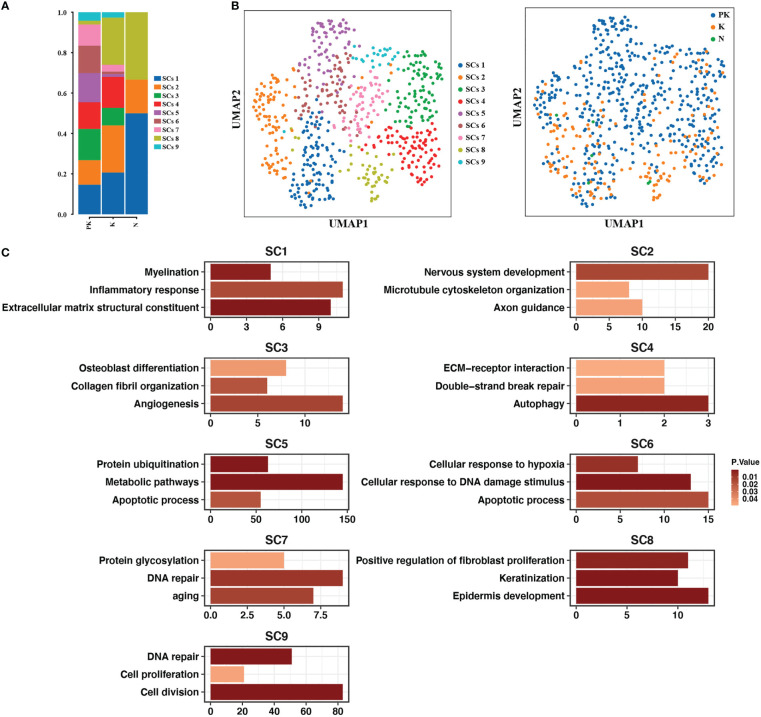
Subpopulation identification of Schwann cells. **(A)** Bar graph showing the proportion of Schwann cell clusters in post-treated keloids (PK), keloids (K), and normal skins (N). **(B)** UMAP plots showing the descending clustering of Schwann cells by cell cluster (left panel) and group (right panel), respectively, with the left panel color representing the cluster origin of each cell and the right panel color representing the group origin of each cell. **(C)** Functional enrichment of upregulated genes in each Schwann cell cluster with significance threshold set at P value < 0.05.

Since pharmacological treatment can effectively relieve the pruritus and tingling of keloids, we characterized in detail the Schwann cell clusters that maintain sensory neuron function. A total of nine Schwann cell subtypes were examined. The Schwann cell clusters in keloids were substantially increased in number compared to normal skins (**Figures 6A, B**). The Schwann cell population in normal skin consisted of cluster 1, cluster 2, and cluster 8 (**Figure 6A**). Cluster 1 expressed genes for myelin formation (MBP, PLLP, LPAR1, MAL, EGR2) and cluster 2 upregulated the expression of axon guidance-related genes (LGI1, NOTCH3, EFNB1, TRIO, LAMA2, BDNF, DPYSL2, MYCBP2, NEO1, SCN1B). Cluster 8 was characterized by genes that promoted epidermis development and fibroblast proliferation (**Figure 6C**). A greater diversity of Schwann cell clusters was observed in keloids, as evidenced by the detection of cluster 9 associated with cell proliferation and division and cluster 3 promoting angiogenesis and collagen fibril organization. The proportion of cells in clusters 5, 6, and 7, which upregulated the expression of apoptotic or senescence-related genes, was increased in treated keloids, while the ratio of cluster 2, which was associated with the development of the nervous system, and cluster 8, which promoted fibroblast proliferation, were reduced (**Figures 6A, C**).

**Figure 7:**
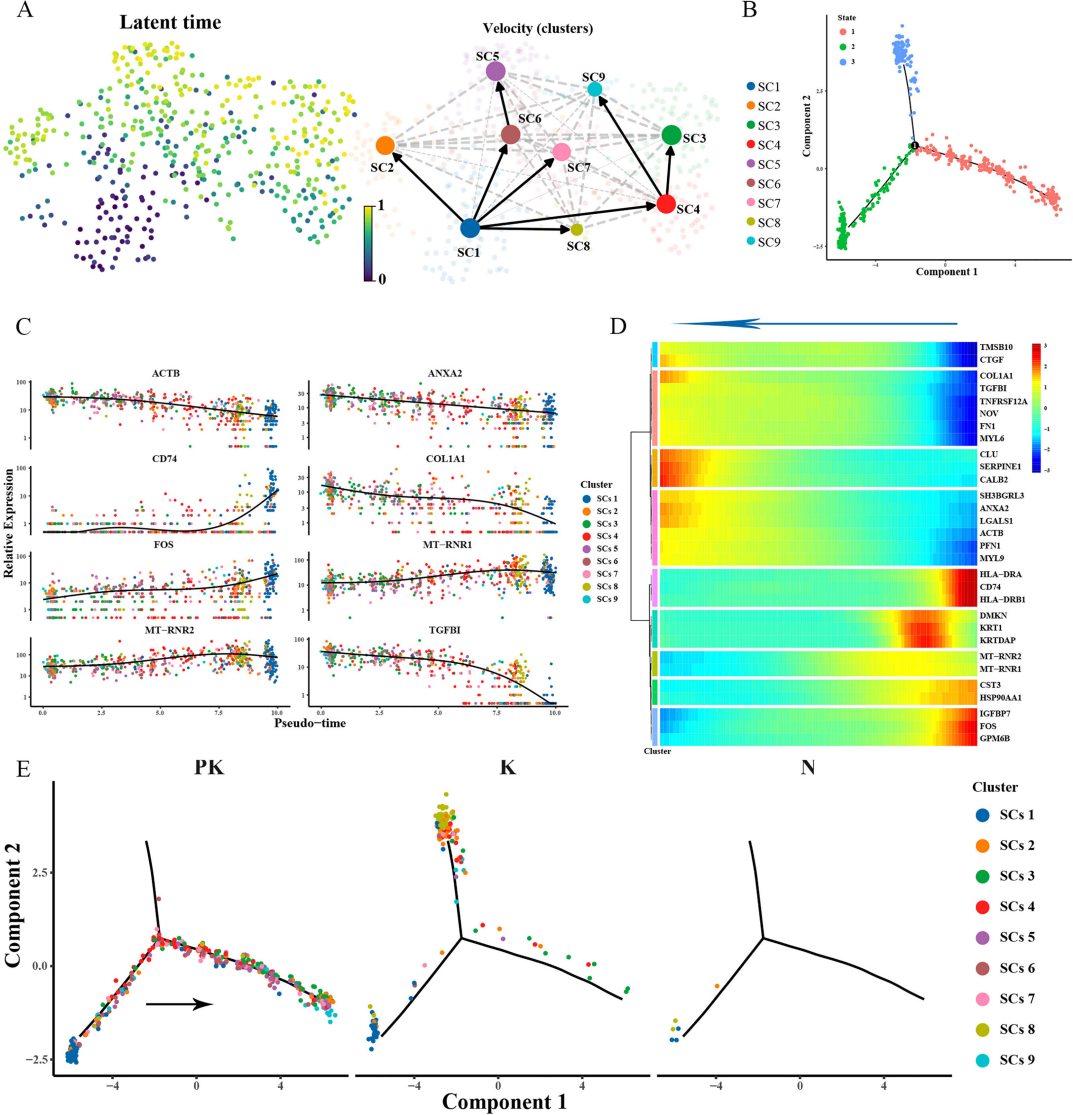
Remodeling of the differentiation trajectory of Schwann cells. **(A)** Presentation of the differentiation latency times of Schwann cells inferred from scvelo analysis on the left, from 0 to 1 representing the early and late times of generation of the cells, respectively. Display of the direction of differentiation of individual cells with arrows representing the direction of differentiation is shown on the right. **(B)** Pseudotime sorting of Schwann cells revealed a branching trajectory and identified 3 differentiation states. **(C)** Display of genes differentially expressed with pseudotime. The vertical coordinate is the expression of the gene, and different colors indicate different Schwann cell clusters. **(D)** Hierarchical clustering of branching-dependent genes revealed 9 gene modules and demonstrated representative genes for each gene module with pseudo-time. **(E)** Cell fate transition of Schwann cells in keloid after TAC+5-FU injection treatment (left panel), keloid (middle panel), and skin (right panel). Colors indicate Schwann cell clusters.

RNA velocity analysis revealed that the population of Schwann cells in keloid originated from myelin formation-associated Schwann cell cluster 1 (**Figure 7A**). The pseudotime trajectory in keloids included states 2 and 3 (**Figures 7B**, **E**), while treated keloid cluster 1 cells gradually acquired apoptotic (cluster 5, cluster 6) and proliferative (cluster 9) states during differentiation (**Figure 7E**), suggesting that myelin formation-associated Schwann cell clusters dedifferentiated and decreased their ability to promote myelin formation. This process was accompanied by increased expression of CLU, a gene involved in cell death and neurodegenerative diseases, and decreased expression of the immune genes HLA-DRA, HLADRB1 and CD74 (**Figures 7C, D**).

## 4 Discussion

Keloid scars, as a representative of skin fibrosis, are the consequence of excessive extracellular matrix deposition following dermal injury (37, 38). Although keloid scars have been extensively studied by many scholars, theories are not yet available to thoroughly explain the pathology and molecular mechanisms of keloids. A variety of treatment options are applied to keloid scarring, including pharmacological treatment, surgical excision of the lesion, radiation therapy, cryotherapy, and laser therapy (39). Triamcinolone acetonide combined with 5-fluorouracil injection therapy is recommended as the first-line drug treatment option for keloids. The injection of the combination of these two drugs, which is more than 80% effective in treating keloids, can markedly inhibit fibroblast proliferation, the key mechanisms underlying this treatment regimen remain understudied (40). ScRNA-seq has yielded groundbreaking insights into the molecular mechanisms of multiple diseases by enabling the construction of disease microenvironmental maps through cell clustering and the simulation of cell developmental trajectories. ScRNA-seq documents a wide range of gene expression differences at the single-cell level, and these aberrant gene expression modulations are challenging to achieve independent and comprehensive measurements by traditional molecular experimental techniques (41, 42). Therefore, we applied scRNA-seq to capture transcriptional profile changes in keloids after TAC+5-FU injection treatment and concentrated on altered pathways involved in the fibrotic process, which could provide new molecular-level insights for understanding this treatment option.

In this study, we systematically evaluated the cellular composition alterations in keloids after TAC+5-FU injection treatment. Fibroblasts, the central cells of fibrosis, had a reduced proportion after treatment and communication with other cells was impaired, particularly in the FGF2 signaling pathway. FGF2 has a regulatory role in the formation of fibrotic disease, and aberrant expression of the FGF2 signaling pathway was reported in lung, liver, kidney, myocardial and skin fibrosis (43–45). The FGF2 signaling pathway among fibroblasts was significantly inhibited in keloids treated with TAC+5-FU, suggesting that it may be a potential pathway target. Moreover, the trend of differentiation toward mesenchymal fibroblasts was suppressed in treated keloids in pseudotime analysis. Mesenchymal fibroblasts are highly expressed in genes related to ossification and osteogenesis and are a remarkably increased fibroblast subtype in keloids compared to normal scars (27). Treatment of mesenchymal fibroblasts with neutralizing antibodies to POSTN, a marker gene for mesenchymal fibroblasts, significantly inhibited collagen synthesis (46). A similar increase in the proportion of mesenchymal fibroblasts was observed in scleroderma, and this trend may be a general mechanism for promoting skin fibrosis (46). The decrease in differentiation toward mesenchymal fibroblasts in treated keloids implies that mesenchymal fibroblasts may be the potential target cells for TAC+5-FU effects. Furthermore, previous clinical experience has found that multiple, long-term injections of TAC+5-FU effectively promote durable softening of keloids, whereas single-drug injections, of either TAC or 5-FU, frequently result in a period of symptomatic relief and subsequent recurrence (47, 48). Our results suggested that treated keloid fibroblasts produced self-replicating competent cluster 10 and multidirectional differentiated cluster 4, which were induced to arise after tissue injury and possess a strong ability to reconstitute the keloid microenvironment and may be the cellular source of keloid recurrence.

The function of Schwann cells is not merely confined to the secretion of neurotrophic factors and the promotion of nerve damage repair. Under specific circumstances, Schwann cell precursors have the potential to generate into melanoblasts, fibroblasts and other cell populations (49). TAC+5-FU injection increased the number of Schwann cell clusters that upregulated apoptosis or senescence-related gene expression and decreased the proportion of Schwann cell clusters with the potential to promote epidermal development and fibroblast proliferation (the major Schwann cell clusters in normal keloids). Previous studies have demonstrated that Schwann cells in keloids originate from myeloid Schwann cells and gradually dedifferentiate to possess fibroblastic or endothelial cell properties and proliferative characteristics (50, 51). Our results also indicated that the differentiation trajectory of Schwann cell clusters started from myelin-promoting Schwann cell clusters, however, TAC+5-FU induced Schwann cells to diverge more toward apoptotic, senescent Schwann cell populations.

The pathogenesis of keloid scars has been recognized widely as extracellular matrix deposition driven by a sustained inflammatory response in the dermis (7). Previous profiling of cell types associated with immune responses in keloids has identified an increased proportion of M2 type macrophages with characteristics of tumor-associated macrophages (18). M2-type macrophages are essential cells that play a role in inducing fibrotic disease by secreting growth factors and regulating fibroblast proliferation and extracellular matrix synthesis (52). Our results also identified macrophages in the subpopulation identification of the mononuclear phagocyte system and had the characteristics of M2-type macrophages, which is consistent with previous reports. Additionally, the expression of secretoglobulin family members SCGB2A2, SCGB1D2 and SCGB1B2P was upregulated after treatment in the T cell expression profile. Secretoglobulins are a group of small, non-glycosylated protein families represented by steroid hormone-induced production of uteroglobulins, and several family members have been reported to play an anti-inflammatory role in allergic diseases (53, 54). Alterations in secretoglobulins expression can remove the drivers of keloid scarring by inhibiting the inflammatory response and is expected to be a potential drug target for inhibiting keloid growth.

In conclusion, we performed the systematic analysis of keloids after TAC+5-FU injection treatment at single-cell resolution for the first time. Our findings elucidated the developmental dynamic patterns of fibroblasts and Schwann cells and the abnormal regulation of immune-related gene expression, which will contribute to an in-depth understanding of the potential mechanisms of action and possible therapeutic targets of TAC+5-FU on keloids.

## Data availability statement

The datasets presented in this study can be found in online repositories. The name of the repository and accession number can be found below: NCBI Sequence Read Archive; PRJNA967834.

## Ethics statement

The experimental plan for the present study was approved (approval no. JS-2907) by The Medical Ethics Committee of Peking Union Medical College Hospital (Beijing, China). Written and photographic informed consents were obtained from all participants.

## Author contributions

YX performed the data analysis and were major contributors to the preparation of the manuscript. YW provided technical support in the methodology of the raw analysis. YW, YH and MS made significant contributions to the design of the study. HL confirmed the authenticity of all raw data. ZL and XK checked the final manuscript. All authors read and approved the final manuscript.

## Supplementary material

Supplementary Figure 1 ScRNA-seq profiling of keloids after TAC+5-FU injection treatment, keloids, and normal skins. **(A)** UMAP projections of 18, 605 cells with unbiased clustering, distinguished by different samples. **(B)** Heat map of differentially expressed genes. For each cluster, the top 10 genes and their relative expression levels in all sequenced cells were shown. **(C)** The proportion of each cell type in each sample. UMAP, uniform manifold approximation and projection; TAC, triamcinolone acetonide; 5-FU, 5-fluorouracil; ECs, endothelial cells; MPs, mononuclear phagocytes.

Supplementary Figure 2 The subtypes of fibroblasts. **(A)** Expression of markers of four fibroblast subtypes (mesenchymal fibroblasts, pro-inflammatory fibroblasts, secretory-papillary fibroblasts and secretory-reticular fibroblasts) in fibroblast clusters. The color indicates the expression level and the size of the bubble denotes the percentage of expression. **(B)** Demonstration of genes that were differentially expressed with pseudo-time. The vertical coordinate is the amount of gene expression, and different colors represent different fibroblast clusters. **(C)** Heat map of differentially expressed genes. For each fibroblast cluster, the top 10 genes and their relative expression levels were displayed. Horizontal coordinates represent fibroblast clusters. UMAP, uniform manifold approximation and projection; TAC, triamcinolone acetonide; 5-FU, 5-fluorouracil.

Supplementary Figure 3 Histologic validation of differences in TAC+5-FU injection, keloid, and keloid-adjacent skin, including immunohistochemical analysis of Ki67 and BAX and immunofluorescence of FN1 and OPN.

## References

[1] Kim HJ Park J Kim SK Park H Kim JE Lee S. Autophagy: guardian of skin barrier. Biomedicines (2022) 10(8):1817. doi: 10.3390/biomedicines10081817 36009363 PMC9405116

[2] Sylakowski K Wells A. Ecm-regulation of autophagy: the yin and the yang of autophagy during wound healing. Matrix Biol (2021) 100-101:197–206. doi: 10.1016/j.matbio.2020.12.006 33421547 PMC8257752

[3] Limandjaja GC Niessen FB Scheper RJ Gibbs S. The keloid disorder: heterogeneity, histopathology, mechanisms and models. Front Cell Dev Biol (2020) 8:360. doi: 10.3389/fcell.2020.00360 32528951 PMC7264387

[4] Sunaga A Kamochi H Sarukawa S Uda H Sugawara Y Asahi R. Reconstitution of human keloids in mouse skin. Plast Reconstr Surg Glob Open (2017) 5(4):e1304. doi: 10.1097/gox.0000000000001304 28507865 PMC5426884

[5] Berman B Maderal A Raphael B. Keloids and hypertrophic scars: pathophysiology, classification, and treatment. Dermatol Surg (2017) 43(Suppl 1):S3–s18. doi: 10.1097/dss.0000000000000819 27347634

[6] Harn HI Ogawa R Hsu CK Hughes MW Tang MJ Chuong CM. The tension biology of wound healing. Exp Dermatol (2019) 28(4):464–71. doi: 10.1111/exd.13460 29105155

[7] Ogawa R. Keloid and hypertrophic scars are the result of chronic inflammation in the reticular dermis. Int J Mol Sci (2017) 18(3):606. doi: 10.3390/ijms18030606 28287424 PMC5372622

[8] Zhuang Z Li Y Wei X. The safety and efficacy of intralesional triamcinolone acetonide for keloids and hypertrophic scars: a systematic review and meta-analysis. Burns (2021) 47(5):987–98. doi: 10.1016/j.burns.2021.02.013 33814214

[9] Roques C Téot L. The use of corticosteroids to treat keloids: a review. Int J Low Extrem Wounds (2008) 7(3):137–45. doi: 10.1177/1534734608320786 18611924

[10] Trisliana Perdanasari A Lazzeri D Su W Xi W Zheng Z Ke L. Recent developments in the use of intralesional injections keloid treatment. Arch Plast Surg (2014) 41(6):620–9. doi: 10.5999/aps.2014.41.6.620 PMC4228202 25396172

[11] Wendling J Marchand A Mauviel A Verrecchia F. 5-fluorouracil blocks transforming growth factor-Beta-Induced alpha 2 type I collagen gene (Col1a2) expression in human fibroblasts *Via* c-jun Nh2-terminal Kinase/Activator protein-1 activation. Mol Pharmacol (2003) 64(3):707–13. doi: 10.1124/mol.64.3.707 12920208

[12] Bijlard E Steltenpool S Niessen FB. Intralesional 5-fluorouracil in keloid treatment: a systematic review. Acta Derm Venereol (2015) 95(7):778–82. doi: 10.2340/00015555-2106 25805099

[13] Wu Y Wang GJ He HQ Qin HH Shen WT Yu Y. Low-dose intralesional injection of 5-fluorouracil and triamcinolone reduces tissue resident memory T cells in chronic eczema. World J Clin cases (2022) 10(1):166–76. doi: 10.12998/wjcc.v10.i1.166 PMC8727240 35071516

[14] Morelli Coppola M Salzillo R Segreto F Persichetti P. Triamcinolone acetonide intralesional injection for the treatment of keloid scars: patient selection and perspectives. Clin Cosmet Investig Dermatol (2018) 11:387–96. doi: 10.2147/ccid.S133672 PMC6063260 30087573

[15] Shah VV Aldahan AS Mlacker S Alsaidan M Samarkandy S Nouri K. 5-fluorouracil in the treatment of keloids and hypertrophic scars: a comprehensive review of the literature. Dermatol Ther (Heidelb) (2016) 6(2):169–83. doi: 10.1007/s13555-016-0118-5 PMC4906112 27105629

[16] Chen G Ning B Shi T. Single-cell rna-seq technologies and related computational data analysis. Front Genet (2019) 10:317. doi: 10.3389/fgene.2019.00317 31024627 PMC6460256

[17] Hwang B Lee JH Bang D. Single-cell rna sequencing technologies and bioinformatics pipelines. Exp Mol Med (2018) 50(8):1–14. doi: 10.1038/s12276-018-0071-8 PMC6082860 30089861

[18] Feng C Shan M Xia Y Zheng Z He K Wei Y. Single-cell rna sequencing reveals distinct immunology profiles in human keloid. Front Immunol (2022) 13:940645. doi: 10.3389/fimmu.2022.940645 35990663 PMC9381754

[19] Dura B Choi JY Zhang K Damsky W Thakral D Bosenberg M. Scftd-seq: freeze-thaw lysis based, portable approach toward highly distributed single-cell 3' mrna profiling. Nucleic Acids Res (2019) 47(3):e16. doi: 10.1093/nar/gky1173 30462277 PMC6379653

[20] Wolf FA Angerer P Theis FJ. Scanpy: Large-scale single-cell gene expression data analysis. Genome Biol (2018) 19(1):15. doi: 10.1186/s13059-017-1382-0 29409532 PMC5802054

[21] Yu G Wang LG Han Y He QY. Clusterprofiler: an r package for comparing biological themes among gene clusters. Omics (2012) 16(5):284–7. doi: 10.1089/omi.2011.0118 PMC3339379 22455463

[22] Hänzelmann S Castelo R Guinney J. Gsva: gene set variation analysis for microarray and rna-seq data. BMC Bioinf (2013) 14:7. doi: 10.1186/1471-2105-14-7 PMC3618321 23323831

[23] Qiu X Mao Q Tang Y Wang L Chawla R Pliner HA. Reversed graph embedding resolves complex single-cell trajectories. Nat Methods (2017) 14(10):979–82. doi: 10.1038/nmeth.4402 PMC5764547 28825705

[24] Bergen V Lange M Peidli S Wolf FA Theis FJ. Generalizing rna velocity to transient cell states through dynamical modeling. Nat Biotechnol (2020) 38(12):1408–14. doi: 10.1038/s41587-020-0591-3 32747759

[25] Efremova M Vento-Tormo M Teichmann SA Vento-Tormo R. Cellphonedb: inferring cell-cell communication from combined expression of multi-subunit ligand-receptor complexes. Nat Protoc (2020) 15(4):1484–506. doi: 10.1038/s41596-020-0292-x 32103204

[26] Kumar N Mishra B Athar M Mukhtar S. Inference of gene regulatory network from single-cell transcriptomic data using pyscenic. Methods Mol Biol (2021) 2328:171–82. doi: 10.1007/978-1-0716-1534-8_10 34251625

[27] Solé-Boldo L Raddatz G Schütz S Mallm JP Rippe K Lonsdorf AS. Single-cell transcriptomes of the human skin reveal age-related loss of fibroblast priming. Commun Biol (2020) 3(1):188. doi: 10.1038/s42003-020-0922-4 32327715 PMC7181753

[28] Lambert SA Jolma A Campitelli LF Das PK Yin Y Albu M. The human transcription factors. Cell (2018) 172(4):650–65. doi: 10.1016/j.cell.2018.01.029 PMC12908702 29425488

[29] Vaquerizas JM Kummerfeld SK Teichmann SA Luscombe NM. A census of human transcription factors: function, expression and evolution. Nat Rev Genet (2009) 10(4):252–63. doi: 10.1038/nrg2538 19274049

[30] Armingol E Officer A Harismendy O Lewis NE. Deciphering cell-cell interactions and communication from gene expression. Nat Rev Genet (2021) 22(2):71–88. doi: 10.1038/s41576-020-00292-x 33168968 PMC7649713

[31] Li L Ji J Song F Hu J. Intercellular receptor-ligand binding: effect of protein-membrane interaction. J Mol Biol (2022) 435(1):167787. doi: 10.1016/j.jmb.2022.167787 35952805

[32] Itoh N Ohta H. Pathophysiological roles of fgf signaling in the heart. Front Physiol (2013) 4:247. doi: 10.3389/fphys.2013.00247 24046748 PMC3764331

[33] Yang L Zhou F Zheng D Wang D Li X Zhao C. Fgf/Fgfr signaling: from lung development to respiratory diseases. Cytokine Growth Factor Rev (2021) 62:94–104. doi: 10.1016/j.cytogfr.2021.09.002 34593304

[34] Yunna C Mengru H Lei W Weidong C. Macrophage M1/M2 polarization. Eur J Pharmacol (2020) 877:173090. doi: 10.1016/j.ejphar.2020.173090 32234529

[35] Böttcher K Rombouts K Saffioti F Roccarina D Rosselli M Hall A. Mait cells are chronically activated in patients with autoimmune liver disease and promote profibrogenic hepatic stellate cell activation. Hepatology (2018) 68(1):172–86. doi: 10.1002/hep.29782 29328499

[36] Constantinides MG Link VM Tamoutounour S Wong AC Perez-Chaparro PJ Han SJ. Mait cells are imprinted by the microbiota in early life and promote tissue repair. Science (2019) 366(6464):eaax6624. doi: 10.1126/science.aax6624 31649166 PMC7603427

[37] Barone N Safran T Vorstenbosch J Davison PG Cugno S Murphy AM. Current advances in hypertrophic scar and keloid management. Semin Plast Surg (2021) 35(3):145–52. doi: 10.1055/s-0041-1731461 PMC8432993 34526861

[38] Feng F Liu M Pan L Wu J Wang C Yang L. Biomechanical regulatory factors and therapeutic targets in keloid fibrosis. Front Pharmacol (2022) 13:906212. doi: 10.3389/fphar.2022.906212 35614943 PMC9124765

[39] Viera MH Vivas AC Berman B. Update on keloid management: clinical and basic science advances. Adv Wound Care (New Rochelle) (2012) 1(5):200–6. doi: 10.1089/wound.2011.0313 PMC3839006 24527306

[40] Ren Y Zhou X Wei Z Lin W Fan B Feng S. Efficacy and safety of triamcinolone acetonide alone and in combination with 5-fluorouracil for treating hypertrophic scars and keloids: a systematic review and meta-analysis. Int Wound J (2017) 14(3):480–7. doi: 10.1111/iwj.12629 PMC7949502 27427423

[41] Haque A Engel J Teichmann SA Lönnberg T. A practical guide to single-cell rna-sequencing for biomedical research and clinical applications. Genome Med (2017) 9(1):75. doi: 10.1186/s13073-017-0467-4 28821273 PMC5561556

[42] Ke M Elshenawy B Sheldon H Arora A Buffa FM. Single cell rna-sequencing: a powerful yet still challenging technology to study cellular heterogeneity. Bioessays (2022) 44(11):e2200084. doi: 10.1002/bies.202200084 36068142

[43] Heggli I Blache U Herger N Mengis T Jaeger PK Schuepbach R. Fgf2 overrides key pro-fibrotic features of bone marrow stromal cells isolated from modic type 1 change patients. Eur Cell Mater (2022) 44:101–14. doi: 10.22203/eCM.v044a07 36254571

[44] Livingston MJ Shu S Fan Y Li Z Jiao Q Yin XM. Tubular cells produce Fgf2 via autophagy after acute kidney injury leading to fibroblast activation and renal fibrosis. Autophagy (2022) 19(1):256–77. doi: 10.1080/15548627.2022.2072054 PMC9809951 35491858

[45] Xiao L Du Y Shen Y He Y Zhao H Li Z. Tgf-beta 1 induced fibroblast proliferation is mediated by the fgf-2/Erk pathway. Front Biosci (Landmark Ed) (2012) 17(7):2667–74. doi: 10.2741/4077 22652804

[46] Deng CC Hu YF Zhu DH Cheng Q Gu JJ Feng QL. Single-cell rna-seq reveals fibroblast heterogeneity and increased mesenchymal fibroblasts in human fibrotic skin diseases. Nat Commun (2021) 12(1):3709. doi: 10.1038/s41467-021-24110-y 34140509 PMC8211847

[47] Srivastava S Patil A Prakash C Kumari H. Comparison of intralesional triamcinolone acetonide, 5-fluorouracil, and their combination in treatment of keloids. World J Plast Surg (2018) 7(2):212–9. PMC6066718 30083505

[48] Khalid FA Mehrose MY Saleem M Yousaf MA Mujahid AM Rehman SU. Comparison of efficacy and safety of intralesional triamcinolone and combination of triamcinolone with 5-fluorouracil in the treatment of keloids and hypertrophic scars: randomised control trial. Burns (2019) 45(1):69–75. doi: 10.1016/j.burns.2018.08.011 30340861

[49] Jessen KR Mirsky R Lloyd AC. Schwann cells: development and role in nerve repair. Cold Spring Harb Perspect Biol (2015) 7(7):a020487. doi: 10.1101/cshperspect.a020487 25957303 PMC4484967

[50] Direder M Weiss T Copic D Vorstandlechner V Laggner M Pfisterer K. Schwann cells contribute to keloid formation. Matrix Biol (2022) 108:55–76. doi: 10.1016/j.matbio.2022.03.001 35278628

[51] Direder M Wielscher M Weiss T Laggner M Copic D Klas K. The transcriptional profile of keloidal schwann cells. Exp Mol Med (2022) 54(11):1886–900. doi: 10.1038/s12276-022-00874-1 PMC9722693 36333467

[52] Li X Wang Y Yuan B Yang H Qiao L. Status of M1 and M2 type macrophages in keloid. Int J Clin Exp Pathol (2017) 10(11):11098–105. PMC6965870 31966458

[53] Jackson BC Thompson DC Wright MW McAndrews M Bernard A Nebert DW. Update of the human secretoglobin (Scgb) gene superfamily and an example of 'Evolutionary bloom' of androgen-binding protein genes within the mouse scgb gene superfamily. Hum Genomics (2011) 5(6):691–702. doi: 10.1186/1479-7364-5-6-691 22155607 PMC3251818

[54] Stoeckelhuber M Messmer EM Schmidt C Xiao F Schubert C Klug J. Immunohistochemical analysis of secretoglobin scgb 2a1 expression in human ocular glands and tissues. Histochem Cell Biol (2006) 126(1):103–9. doi: 10.1007/s00418-005-0137-2 16395610

