# Supplementary material for: Deciphering the single-cell transcriptome network in keloids with intra-lesional injection of triamcinolone acetonide combined with 5-fluorouracil
Source: Front Immunol. 2023 May 19;14:1106289. doi: 10.3389/fimmu.2023.1106289 (PMC10235510; doi:10.3389/fimmu.2023.1106289)
Supplement: Supplementary Figure 1 — ScRNA-seq profiling of keloids after TAC+5-FU injection treatment, keloids, and normal skins. (A) UMAP projections of 18, 605 cells with unbiased clustering, distinguished by different samples. (B) Heat map of differentially expressed genes. For each cluster, the top 10 genes and their relative expression levels in all sequenced cells were shown. (C) The proportion of each cell type in each sample. UMAP, uniform manifold approximation and projection; TAC, triamcinolone acetonide; 5-FU, 5-fluorouracil; ECs, endothelial cells; MPs, mononuclear phagocytes. [file DataSheet_1.docx]

**Figure S1 ScRNA-seq profiling of keloids after TAC+5-FU injection treatment, keloids, and normal skins.** (A) UMAP projections of 18, 605 cells with unbiased clustering, distinguished by different samples. (B) Heat map of differentially expressed genes. For each cluster, the top 10 genes and their relative expression levels in all sequenced cells were shown. (C) The proportion of each cell type in each sample. UMAP, uniform manifold approximation and projection; TAC, triamcinolone acetonide; 5-FU, 5-fluorouracil; ECs, endothelial cells; MPs, mononuclear phagocytes.


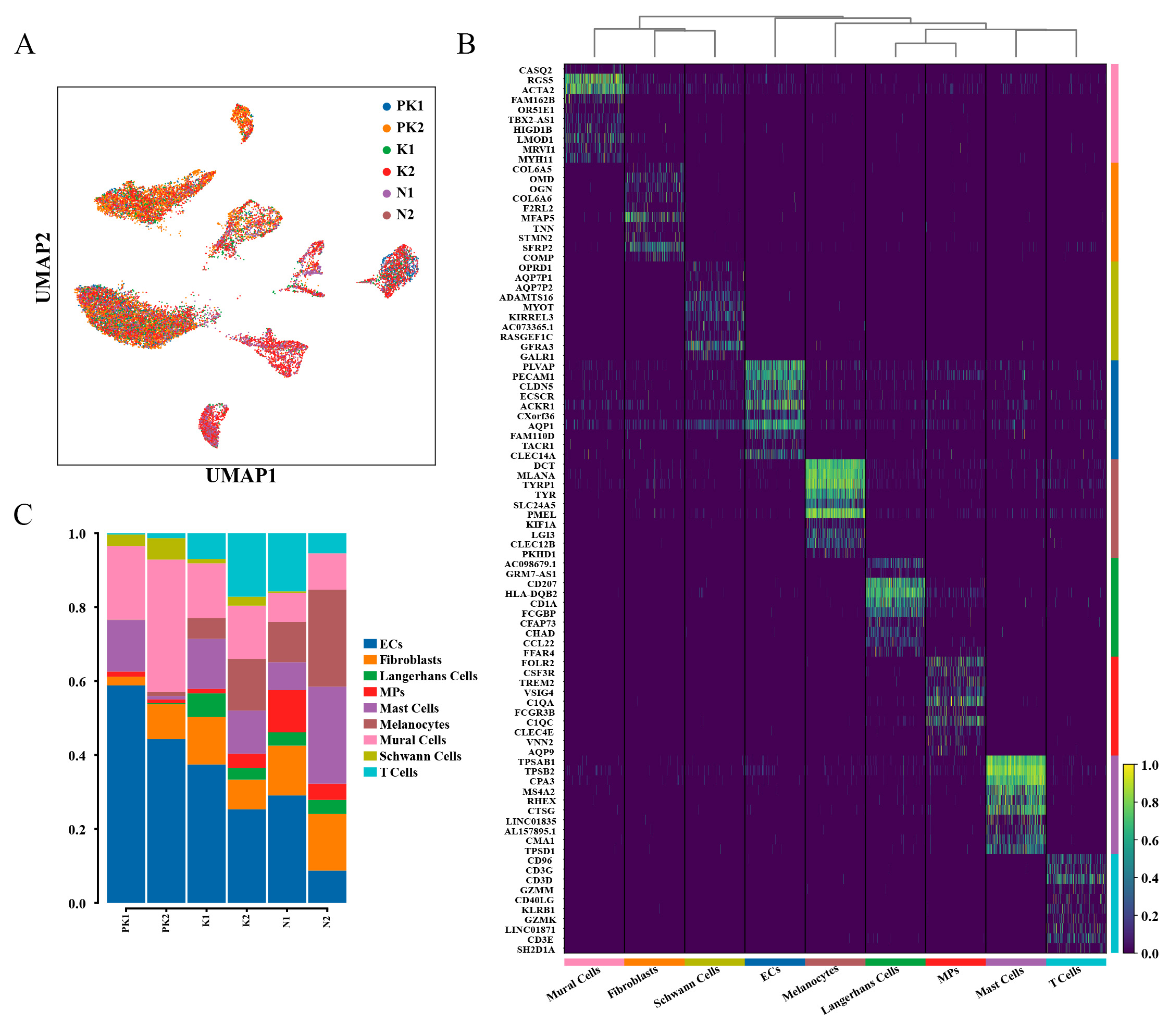


**Figure S2 The subtypes of fibroblasts.** (A) Expression of markers of four fibroblast subtypes (mesenchymal fibroblasts, pro-inflammatory fibroblasts, secretory-papillary fibroblasts and secretory-reticular fibroblasts) in fibroblast clusters. The color indicates the expression level and the size of the bubble denotes the percentage of expression. (B) Demonstration of genes that were differentially expressed with pseudo-time. The vertical coordinate is the amount of gene expression, and different colors represent different fibroblast clusters. (C) Heat map of differentially expressed genes. For each fibroblast cluster, the top 10 genes and their relative expression levels were displayed. Horizontal coordinates represent fibroblast clusters. UMAP, uniform manifold approximation and projection; TAC, triamcinolone acetonide; 5-FU, 5-fluorouracil.


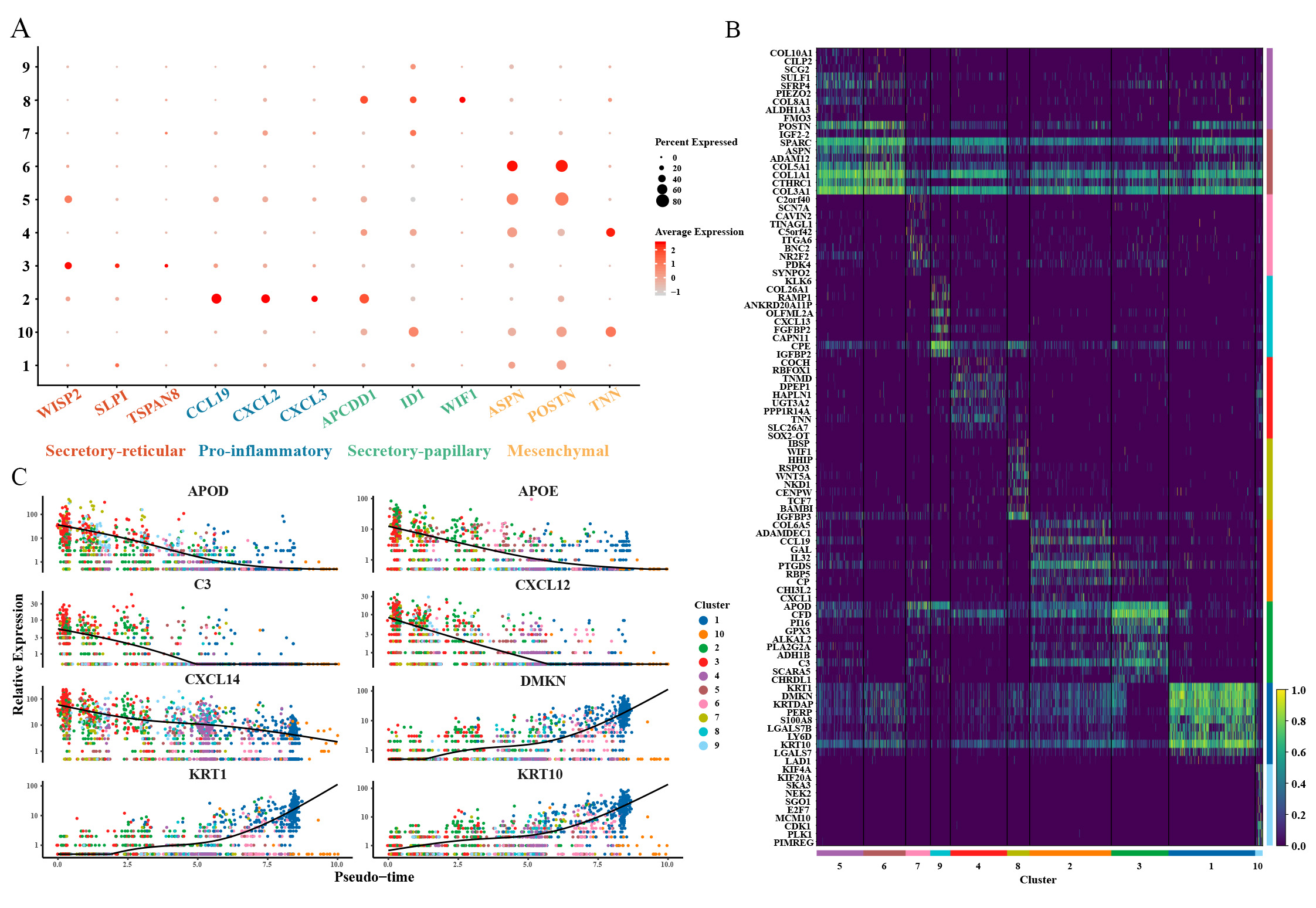


**Figure S3 Histologic validation of differences in TAC+5-FU injection, keloid, and keloid-adjacent skin, including immunohistochemical analysis of Ki67 and BAX and immunofluorescence of FN1 and OPN.**


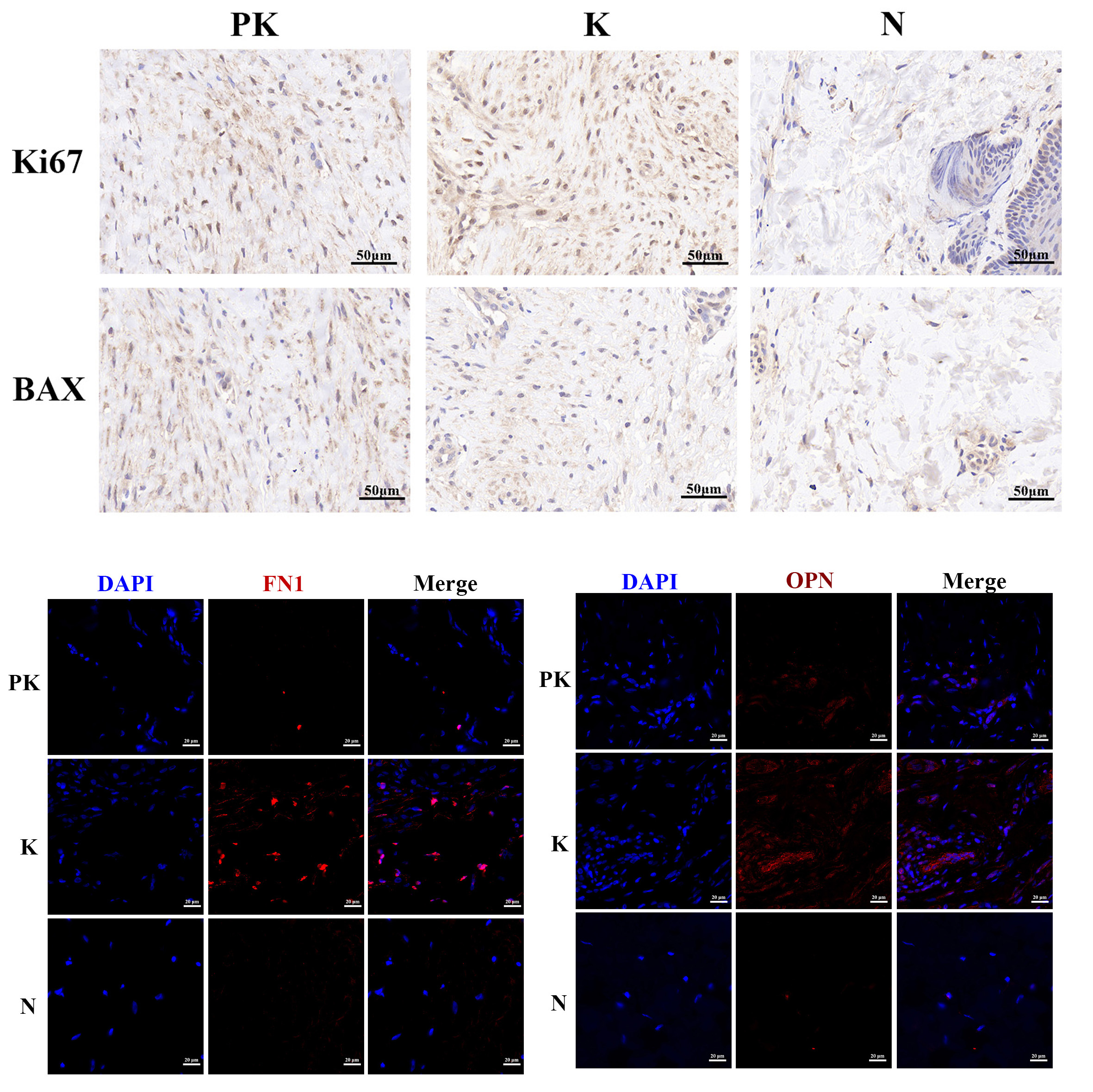


Table S1. Transcription factors and their target genes in fibroblasts were inferred based on pySCENIC analysis.

| TF | Gene | Weight |
| --- | --- | --- |
| ACAA1 | RPL13AP5 | 4.823813069 |
| ACAA1 | KCTD12 | 3.661011118 |
| ACAA1 | RPL11 | 2.90448647 |
| ACAA1 | HOXD8 | 1.426140758 |
| ACAA1 | PRPF4 | 1.417444246 |
| ACAA1 | UBR4 | 0.815431332 |
| ACAA1 | KLHL12 | 0.5993804 |
| ACAA1 | KCNMB3 | 0.318413064 |
| ACO1 | STMN2 | 8.248540734 |
| ACO1 | TGFB2 | 3.010686362 |
| ACO1 | HIVEP2 | 2.837766184 |
| ACO1 | NACC2 | 2.09503227 |
| ACO1 | ADD3 | 1.659320142 |
| ACO1 | PER2 | 1.237556271 |
| ACO1 | FNDC3A | 1.203506415 |
| ACO1 | ATF7 | 1.080200826 |
| ACO1 | HOPX | 1.064414056 |
| ACO1 | MGRN1 | 0.974915442 |
| ACO1 | PPL | 0.894433604 |
| ACO1 | E2F7 | 0.841719052 |
| ACO1 | SEMA3C | 0.739901009 |
| ACO1 | FBXL3 | 0.66546068 |
| ACO1 | LDLRAP1 | 0.630660565 |
| ACO1 | ACVR2A | 0.550187648 |
| ACO1 | NIPBL | 0.522973071 |
| ACO1 | UBE4B | 0.5067227 |
| ACO1 | DCAF6 | 0.498000032 |
| ACO1 | METRNL | 0.459370556 |
| ACO1 | SIAH1 | 0.44741361 |
| ACO1 | EFHD1 | 0.421545925 |
| ACO1 | ZFHX3 | 0.394526471 |
| ACO1 | ATXN1 | 0.360921157 |
| ACO1 | BCL6 | 0.311803404 |
| ACO1 | INPP5F | 0.269082163 |
| ACO1 | SOX12 | 0.267769394 |
| ACO1 | RFTN2 | 0.263151237 |
| ACO1 | LATS2 | 0.254357214 |
| ACO1 | SLC39A8 | 0.247068503 |
| ACO1 | PLEKHA5 | 0.241774563 |
| ACO1 | CREB1 | 0.223890513 |
| ACO1 | RLIM | 0.207315796 |
| ACO1 | NFATC1 | 0.199716719 |
| ACO1 | SP9 | 0.176083581 |
| ACO1 | PLOD1 | 0.152778333 |
| ACO1 | SPSB1 | 0.145503411 |
| ACO1 | NLGN1 | 0.071344386 |
| ACO1 | BARX2 | 0.066966271 |
| ACO1 | IGF2BP2 | 0.054659092 |
| ACO1 | C12orf65 | 0.052587734 |
| ACO1 | PRKCE | 0.036032567 |
| ACO1 | ZBTB16 | 0.000179729 |
| ACO1 | TRIM59 | 2.13E-07 |
| AHR | COL18A1 | 10.39599844 |
| AHR | NFIB | 7.67193311 |
| AHR | RUNX1 | 7.499841127 |
| AHR | FBLN2 | 5.749374237 |
| AHR | SMOC2 | 4.974683417 |
| AHR | SNED1 | 4.874774272 |
| AHR | CYP1B1 | 4.482416409 |
| AHR | BGN | 3.731732619 |
| AHR | SLC38A10 | 2.757057991 |
| AHR | TNK2 | 1.858085742 |
| AHR | CSRNP3 | 1.591377079 |
| AHR | RNF187 | 1.359437558 |
| AHR | FAM46A | 1.236709334 |
| AHR | ZMYND11 | 1.188633947 |
| AHR | SMAD2 | 0.921751972 |
| AHR | FOXP1 | 0.821331042 |
| AHR | PHYHIP | 0.624194988 |
| AHR | FGF1 | 0.607591209 |
| AHR | KCNQ1OT1 | 0.574528413 |
| AHR | RELT | 0.522832434 |
| AHR | TSPAN1 | 0.467453037 |
| ARID3A | NRP1 | 5.919258649 |
| ARID3A | C1QBP | 3.117773149 |
| ARID3A | FEM1B | 2.271123374 |
| ARID3A | ZNF217 | 2.228455397 |
| ARID3A | WDR82 | 2.224538009 |
| ARID3A | NDUFA4L2 | 2.071785801 |
| ARID3A | RYBP | 1.976736711 |
| ARID3A | PLXDC2 | 1.802966091 |
| ARID3A | GJA1 | 1.776614901 |
| ARID3A | LEMD2 | 1.769917505 |
| ARID3A | CTDSP1 | 1.558885573 |
| ARID3A | GYG1 | 1.296678976 |
| ARID3A | ZBTB1 | 1.247560953 |
| ARID3A | ARID3A | 1 |
| ARID3A | BNIP3L | 0.988875816 |
| ARID3A | VPS13A | 0.509920598 |
| ARNT | RAB27A | 4.221914195 |
| ARNT | EIF3E | 3.793724769 |
| ARNT | RPS27 | 3.169331595 |
| ARNT | FAF2 | 2.481688825 |
| ARNT | USP25 | 2.05891114 |
| ARNT | FEN1 | 2.022419931 |
| ARNT | PSMD1 | 1.940475823 |
| ARNT | F2R | 1.887522635 |
| ARNT | B3GNT9 | 1.791295725 |
| ARNT | ATXN10 | 1.769014737 |
| ARNT | MCCC2 | 1.61873085 |
| ARNT | TCOF1 | 1.601879288 |
| ARNT | CDK4 | 1.573259189 |
| ARNT | GALC | 1.495516993 |
| ARNT | LPCAT3 | 1.489279015 |
| ARNT | PPP1R12A | 1.483740225 |
| ARNT | CASP6 | 1.423274941 |
| ARNT | C18orf25 | 1.41941347 |
| ARNT | RUNX1 | 1.418548788 |
| ARNT | ENAH | 1.410541803 |
| ARNT | AIG1 | 1.409991216 |
| ARNT | PRR14 | 1.397744392 |
| ARNT | CD47 | 1.385949018 |
| ARNT | MBTD1 | 1.328572386 |
| ARNT | ALPK2 | 1.325548048 |
| ARNT | TXLNG | 1.311104456 |
| ARNT | MCF2L | 1.25481168 |
| ARNT | LPIN2 | 1.229782988 |
| ARNT | PARP11 | 1.207950923 |
| ARNT | SBF2 | 1.194563855 |
| ARNT | CNOT6L | 1.17260352 |
| ARNT | STMN2 | 1.154954571 |
| ARNT | MEF2D | 1.150397278 |
| ARNT | PIP5K1A | 1.095184918 |
| ARNT | BASP1 | 1.089576369 |
| ARNT | PARP16 | 1.084620986 |
| ARNT | ACD | 1.072104548 |
| ARNT | MLLT10 | 1.045275476 |
| ARNT | SLC25A10 | 1.030433353 |
| ARNT | C4orf33 | 1.014852387 |
| ARNT | TP53BP1 | 1.006141484 |
| ARNT | COMMD8 | 0.978985907 |
| ARNT | ATF7IP | 0.962712969 |
| ARNT | CCDC115 | 0.948936483 |
| ARNT | TRAPPC3 | 0.914078362 |
| ARNT | VOPP1 | 0.857301033 |
| ARNT | MASP1 | 0.842014762 |
| ARNT | PCSK2 | 0.838485868 |
| ARNT | ACAP2 | 0.836671295 |
| ARNT | UTP18 | 0.83336277 |
| ARNT | SYMPK | 0.825427629 |
| ARNT | SLC25A37 | 0.772052386 |
| ARNT | RPS6KA5 | 0.758931829 |
| ARNT | TUSC1 | 0.744580284 |
| ARNT | SYNRG | 0.704665434 |
| ARNT | AKAP11 | 0.701413354 |
| ARNT | SH3KBP1 | 0.665532557 |
| ARNT | AHDC1 | 0.664493913 |
| ARNT | C15orf39 | 0.646376573 |
| ARNT | CHRNB1 | 0.646312685 |
| ARNT | RLIM | 0.635884092 |
| ARNT | RNF19A | 0.633606548 |
| ARNT | TTLL4 | 0.630440234 |
| ARNT | VPS26B | 0.62740438 |
| ARNT | NKRF | 0.568889963 |
| ARNT | SGIP1 | 0.547929045 |
| ARNT | HNRNPD | 0.541550885 |
| ARNT | ZNF777 | 0.533118709 |
| ARNT | PAK1 | 0.530356504 |
| ARNT | ASB7 | 0.52501466 |
| ARNT | PTH1R | 0.515725597 |
| ARNT | RBFOX2 | 0.504834368 |
| ARNT | STX6 | 0.470731202 |
| ARNT | AKAP12 | 0.453959368 |
| ARNT | LACTB2 | 0.428845446 |
| ARNT | TNK2 | 0.405909845 |
| ARNT | HIST1H3A | 0.390374906 |
| ARNT | RBMS3 | 0.367082845 |
| ARNT | SEC11C | 0.330513508 |
| ARNT | ZMYND11 | 0.318188491 |
| ARNT | WSB2 | 0.280930419 |
| ARNT | PXN | 0.260223211 |
| ARNT | RABEP1 | 0.258614793 |
| ARNT | KHSRP | 0.23411084 |
| ARNT | ARMCX3 | 0.229898217 |
| ARNT | AATF | 0.223651126 |
| ARNT | WIPF1 | 0.220321212 |
| ARNT | G6PC3 | 0.206886062 |
| ARNT | FEZ2 | 0.19631372 |
| ARNT | MITD1 | 0.186583441 |
| ARNT | RFTN1 | 0.177408631 |
| ARNT | PCDHB14 | 0.156528743 |
| ARNT | AARS | 0.097300992 |
| ARNT | KRT23 | 0.06291279 |
| ARNT | KLHL12 | 0.054109592 |
| ARNT2 | KIAA1586 | 5.019757602 |
| ARNT2 | WDR13 | 2.38685432 |
| ARNT2 | FAM120AOS | 2.295567417 |
| ARNT2 | ZNF599 | 1.896738072 |
| ARNT2 | RABL3 | 1.369377197 |
| ARNT2 | NAA16 | 1.365744965 |
| ARNT2 | G6PC3 | 1.341048474 |
| ARNT2 | DNAJC3 | 1.259834702 |
| ARNT2 | KCNK15 | 1.242710727 |
| ARNT2 | DNAJA3 | 1.137343014 |
| ARNT2 | VRK2 | 1.098343509 |
| ARNT2 | TRADD | 1.049650774 |
| ARNT2 | CUL3 | 1.036164564 |
| ARNT2 | ARNT2 | 1 |
| ARNT2 | C2 | 0.961794044 |
| ARNT2 | ROR1 | 0.884131257 |
| ARNT2 | IMPDH1 | 0.869417339 |
| ARNT2 | PURA | 0.836187057 |
| ARNT2 | YAP1 | 0.821174539 |
| ARNT2 | FAM110B | 0.607100953 |
| ARNT2 | MTPAP | 0.466137447 |
| ARNT2 | TCTN2 | 0.460190702 |
| ARNT2 | NSD1 | 0.413315421 |
| ARNT2 | ANKRD12 | 0.383713559 |
| ARNT2 | FKBP11 | 0.36049005 |
| ARNT2 | DNM3 | 0.309017355 |
| ARNT2 | SLC39A13 | 0.297945375 |
| ARNT2 | FAM192A | 0.179352172 |
| ARNT2 | OTUD1 | 0.166351124 |
| ARNT2 | CD276 | 0.165225837 |
| ARNT2 | ARHGAP6 | 0.158868713 |
| ARNT2 | GPR157 | 0.132502892 |
| ARNTL | ZFP62 | 2.587020385 |
| ARNTL | ZNF207 | 1.746855204 |
| ARNTL | GABARAPL2 | 1.662217645 |
| ARNTL | ADK | 1.642020031 |
| ARNTL | FGFR3 | 1.58056999 |
| ARNTL | PDPN | 1.335085001 |
| ARNTL | ATP6AP2 | 1.15339392 |
| ARNTL | ARNTL | 1 |
| ARNTL | EDIL3 | 0.955593138 |
| ARNTL | FBXO34 | 0.693891041 |
| ASCL2 | SYT8 | 4.398049979 |
| ASCL2 | SOX15 | 1.70336164 |
| ASCL2 | ASCL2 | 1 |
| ASCL2 | KRT5 | 0.445366143 |
| ATF1 | GDI2 | 7.38305877 |
| ATF1 | CENPW | 4.274074339 |
| ATF1 | BEND6 | 3.493902707 |
| ATF1 | NR1H3 | 3.356399607 |
| ATF1 | ZNF595 | 2.934031565 |
| ATF1 | LSM5 | 2.772555115 |
| ATF1 | KLHL36 | 2.069947141 |
| ATF1 | GINS3 | 2.042328457 |
| ATF1 | MIR3188 | 1.856782122 |
| ATF1 | PAF1 | 1.831214562 |
| ATF1 | NEFM | 1.817579167 |
| ATF1 | VAMP3 | 1.801533768 |
| ATF1 | C19orf66 | 1.758918964 |
| ATF1 | MRPL3 | 1.731172107 |
| ATF1 | MIF | 1.710854096 |
| ATF1 | POLD2 | 1.69400522 |
| ATF1 | RHBDF1 | 1.654849947 |
| ATF1 | MYO1C | 1.567503955 |
| ATF1 | ZNF462 | 1.553841967 |
| ATF1 | UROS | 1.551583799 |
| ATF1 | SEMA4B | 1.515443399 |
| ATF1 | FAM200B | 1.511607161 |
| ATF1 | NXT2 | 1.46986618 |
| ATF1 | EDA2R | 1.457956128 |
| ATF1 | GNE | 1.412653881 |
| ATF1 | C1orf53 | 1.376567136 |
| ATF1 | TMEM123 | 1.281374896 |
| ATF1 | DSE | 1.146711642 |
| ATF1 | PTPN4 | 1.139760021 |
| ATF1 | MAN1A2 | 1.119726103 |
| ATF1 | HPCAL1 | 1.108348895 |
| ATF1 | ICAM1 | 1.106321899 |
| ATF1 | TSPAN18 | 1.068820437 |
| ATF1 | INHBA | 1.027861714 |
| ATF1 | ATF1 | 1 |
| ATF1 | PPP1R14A | 0.986829526 |
| ATF1 | CCT4 | 0.984889319 |
| ATF1 | CDKN1B | 0.979341067 |
| ATF1 | AMMECR1L | 0.956872599 |
| ATF1 | SHF | 0.937637657 |
| ATF1 | SMAD2 | 0.892405414 |
| ATF1 | WDR47 | 0.848508054 |
| ATF1 | CETN3 | 0.843777838 |
| ATF1 | CDC42EP2 | 0.811838446 |
| ATF1 | ARL6 | 0.809539914 |
| ATF1 | MSRB3 | 0.773278044 |
| ATF1 | LONP1 | 0.764959445 |
| ATF1 | CASC4 | 0.742845341 |
| ATF1 | NRBP1 | 0.714842543 |
| ATF1 | APLP2 | 0.708134999 |
| ATF1 | SCG2 | 0.699469502 |
| ATF1 | CCNL1 | 0.679806544 |
| ATF1 | ZNF720 | 0.67022466 |
| ATF1 | SRRM2 | 0.657425099 |
| ATF1 | ITGA8 | 0.651569938 |
| ATF1 | BCL9L | 0.651039372 |
| ATF1 | C1orf112 | 0.636064029 |
| ATF1 | MFAP2 | 0.63585325 |
| ATF1 | CDC42 | 0.633531942 |
| ATF1 | GLIPR2 | 0.626758809 |
| ATF1 | CLPX | 0.620990274 |
| ATF1 | DSG3 | 0.607103076 |
| ATF1 | BTG3 | 0.606011813 |
| ATF1 | C9orf72 | 0.599767812 |
| ATF1 | REEP1 | 0.597331595 |
| ATF1 | ZNF77 | 0.589397773 |
| ATF1 | GRB2 | 0.588983149 |
| ATF1 | HDLBP | 0.587295347 |
| ATF1 | NEK7 | 0.58627933 |
| ATF1 | RAB2A | 0.5837951 |
| ATF1 | DNAJB1 | 0.580410222 |
| ATF1 | MID1 | 0.563314772 |
| ATF1 | APOM | 0.558247347 |
| ATF1 | HLF | 0.54763974 |
| ATF1 | KLF10 | 0.538626388 |
| ATF1 | PTPRK | 0.535733335 |
| ATF1 | CLSTN3 | 0.531669075 |
| ATF1 | CCNI | 0.530669585 |
| ATF1 | TRAPPC3 | 0.522470166 |
| ATF1 | STOM | 0.520564607 |
| ATF1 | ANGPTL1 | 0.507072347 |
| ATF1 | NDRG1 | 0.50236447 |
| ATF1 | HOPX | 0.50038051 |
| ATF1 | SRRM3 | 0.497197467 |
| ATF1 | SOCS1 | 0.492553581 |
| ATF1 | ORMDL1 | 0.489812927 |
| ATF1 | COX6A1 | 0.4897899 |
| ATF1 | LSM12 | 0.478365412 |
| ATF1 | DAP | 0.474104955 |
| ATF1 | AHI1 | 0.459586309 |
| ATF1 | LZTFL1 | 0.454516504 |
| ATF1 | BCLAF1 | 0.446897081 |
| ATF1 | SERP1 | 0.446584837 |
| ATF1 | TRNP1 | 0.436938473 |
| ATF1 | NFE2L3 | 0.410241153 |
| ATF1 | FANCM | 0.397842909 |
| ATF1 | HLA-DMB | 0.371549502 |
| ATF1 | LONRF3 | 0.340575995 |
| ATF1 | ARRDC3 | 0.332394196 |
| ATF1 | FANCI | 0.329249276 |
| ATF1 | PIK3C2B | 0.325550597 |
| ATF1 | RNF149 | 0.304322659 |
| ATF1 | VAV3 | 0.285055162 |
| ATF1 | C5orf46 | 0.28427563 |
| ATF1 | TMEM115 | 0.284181012 |
| ATF1 | HLA-DRB1 | 0.27806546 |
| ATF1 | WLS | 0.252089656 |
| ATF1 | NFE2L1 | 0.219949757 |
| ATF1 | RAB6A | 0.188812044 |
| ATF1 | PPME1 | 0.173356289 |
| ATF1 | FOXF2 | 0.098659273 |
| ATF1 | MMP9 | 0.047728997 |
| ATF2 | NRP1 | 3.466727018 |
| ATF2 | HIVEP2 | 3.070006326 |
| ATF2 | SERPINB7 | 2.445116339 |
| ATF2 | TIMP3 | 1.724697778 |
| ATF2 | GABARAPL2 | 1.628838535 |
| ATF2 | SPATA2 | 1.581879889 |
| ATF2 | MAP3K3 | 1.434810532 |
| ATF2 | ZDHHC2 | 1.369495623 |
| ATF2 | ZFYVE9 | 1.283523302 |
| ATF2 | RALGPS2 | 1.232125366 |
| ATF2 | MAP2 | 1.206261759 |
| ATF2 | SLK | 1.136560038 |
| ATF2 | PPP1R13B | 1.083471146 |
| ATF2 | PRSS23 | 1.026957837 |
| ATF2 | ATF2 | 1 |
| ATF3 | FOSB | 24.94576237 |
| ATF3 | NFKBIA | 24.77822693 |
| ATF3 | NR4A1 | 20.52114813 |
| ATF3 | FOS | 19.04385122 |
| ATF3 | CXCL2 | 18.96490607 |
| ATF3 | HES1 | 18.43538613 |
| ATF3 | ICAM1 | 17.54381097 |
| ATF3 | IL6 | 14.12368615 |
| ATF3 | RGS16 | 13.72504753 |
| ATF3 | HSPA8 | 13.45328994 |
| ATF3 | DNAJB1 | 13.37646416 |
| ATF3 | PPP1R15A | 12.68650155 |
| ATF3 | ZFP36 | 12.68191287 |
| ATF3 | TNFAIP3 | 12.195757 |
| ATF3 | DUSP1 | 11.98966087 |
| ATF3 | IRF1 | 11.9877213 |
| ATF3 | EGR1 | 11.85522618 |
| ATF3 | HSPB1 | 11.1739303 |
| ATF3 | KLF6 | 10.94629492 |
| ATF3 | MYC | 10.79893156 |
| ATF3 | IER3 | 10.71125218 |
| ATF3 | PHLDA1 | 9.453216767 |
| ATF3 | JUNB | 9.413764217 |
| ATF3 | HSPA1A | 8.914836631 |
| ATF3 | GADD45A | 8.751041431 |
| ATF3 | JUND | 8.575485235 |
| ATF3 | HSPE1 | 8.116927205 |
| ATF3 | ERRFI1 | 8.086155378 |
| ATF3 | ADAMTS1 | 7.985766172 |
| ATF3 | GEM | 7.906938242 |
| ATF3 | SOCS3 | 7.187402877 |
| ATF3 | CDKN1A | 6.547605472 |
| ATF3 | TNFAIP6 | 6.434042249 |
| ATF3 | HSP90AA1 | 6.259610471 |
| ATF3 | IER2 | 5.503059305 |
| ATF3 | DUSP5 | 5.476720092 |
| ATF3 | BAG3 | 5.378529714 |
| ATF3 | SRSF7 | 5.081822627 |
| ATF3 | KLF4 | 4.999128772 |
| ATF3 | UBB | 4.89648664 |
| ATF3 | NFKBIZ | 4.839950488 |
| ATF3 | RND3 | 4.577075408 |
| ATF3 | DNAJB4 | 4.328373863 |
| ATF3 | EIF4A3 | 4.26879203 |
| ATF3 | MXRA5 | 4.241969526 |
| ATF3 | CEBPD | 4.210678017 |
| ATF3 | EIF1 | 4.193602278 |
| ATF3 | JUN | 4.125345343 |
| ATF3 | S100A9 | 4.003794289 |
| ATF3 | EPS8L1 | 3.904549835 |
| ATF3 | GADD45B | 3.806725634 |
| ATF3 | NFIL3 | 3.694566706 |
| ATF3 | SOD2 | 3.628927453 |
| ATF3 | H3F3B | 3.458224185 |
| ATF3 | PDE4DIP | 3.454689679 |
| ATF3 | HSPH1 | 3.230757485 |
| ATF3 | XG | 3.208992003 |
| ATF3 | HNRNPC | 3.203099588 |
| ATF3 | HSP90AB1 | 3.06894814 |
| ATF3 | POLR2G | 2.95784873 |
| ATF3 | CYR61 | 2.921843052 |
| ATF3 | UBC | 2.788827488 |
| ATF3 | ID3 | 2.621013193 |
| ATF3 | CXCL1 | 2.427062406 |
| ATF3 | MAFF | 2.384049272 |
| ATF3 | CXCL3 | 2.346550791 |
| ATF3 | IER5 | 2.338617762 |
| ATF3 | LAMC1 | 2.332147061 |
| ATF3 | RASD1 | 2.323934591 |
| ATF3 | AEBP1 | 2.313460681 |
| ATF3 | CRYAB | 2.301165439 |
| ATF3 | HECA | 2.23174986 |
| ATF3 | ARRDC3 | 2.186316833 |
| ATF3 | RGS2 | 2.173223518 |
| ATF3 | IL1RL1 | 2.1498537 |
| ATF3 | BHLHE40 | 2.138351374 |
| ATF3 | IL6ST | 2.129622681 |
| ATF3 | BRD2 | 2.12138945 |
| ATF3 | FSTL3 | 2.100875753 |
| ATF3 | PTGS2 | 2.09075559 |
| ATF3 | BCAS2 | 2.083709134 |
| ATF3 | HAS2 | 2.073796168 |
| ATF3 | SERPINE1 | 2.062469849 |
| ATF3 | TSPO | 2.061571997 |
| ATF3 | HP1BP3 | 2.057951428 |
| ATF3 | NCOA7 | 2.017076199 |
| ATF3 | HSPA6 | 2.005915596 |
| ATF3 | ARL6IP5 | 2.000092297 |
| ATF3 | ALOX5AP | 1.962180072 |
| ATF3 | MEX3C | 1.939519932 |
| ATF3 | HOXA9 | 1.905652554 |
| ATF3 | KLF10 | 1.875373578 |
| ATF3 | MAP1LC3B | 1.875061591 |
| ATF3 | CDCA3 | 1.859945084 |
| ATF3 | PPP6R1 | 1.852438774 |
| ATF3 | KRT5 | 1.843681111 |
| ATF3 | RPL3 | 1.819574785 |
| ATF3 | TOX3 | 1.803550456 |
| ATF3 | ARHGAP10 | 1.79776693 |
| ATF3 | EMP1 | 1.773061695 |
| ATF3 | CRYZL1 | 1.748010525 |
| ATF3 | DAB2 | 1.745100104 |
| ATF3 | MMP2 | 1.694756623 |
| ATF3 | FBN1 | 1.68786011 |
| ATF3 | MT2A | 1.680869577 |
| ATF3 | CSRNP1 | 1.664910077 |
| ATF3 | RRBP1 | 1.661120692 |
| ATF3 | MYH11 | 1.652342478 |
| ATF3 | SAT1 | 1.651711202 |
| ATF3 | SIX4 | 1.64911388 |
| ATF3 | SUPT16H | 1.6167484 |
| ATF3 | POSTN | 1.600279352 |
| ATF3 | AGBL5 | 1.582171191 |
| ATF3 | ID4 | 1.569506436 |
| ATF3 | DPT | 1.554892937 |
| ATF3 | MID1IP1 | 1.535482224 |
| ATF3 | JAG2 | 1.488490144 |
| ATF3 | MMP3 | 1.477995286 |
| ATF3 | HSPD1 | 1.447023585 |
| ATF3 | FAM49A | 1.44427754 |
| ATF3 | ZFP36L1 | 1.431737932 |
| ATF3 | HTRA1 | 1.425787774 |
| ATF3 | PTTG1IP | 1.405718996 |
| ATF3 | CD81 | 1.402468466 |
| ATF3 | JOSD1 | 1.371974999 |
| ATF3 | IDH2 | 1.369599273 |
| ATF3 | ARL4D | 1.36925593 |
| ATF3 | FEN1 | 1.36332352 |
| ATF3 | FAM114A1 | 1.344810616 |
| ATF3 | KIF5C | 1.32941706 |
| ATF3 | RPUSD2 | 1.311286252 |
| ATF3 | SGK1 | 1.309831233 |
| ATF3 | HLA-DRB5 | 1.307296268 |
| ATF3 | NEAT1 | 1.298279046 |
| ATF3 | DDX20 | 1.29687556 |
| ATF3 | GCAT | 1.295445467 |
| ATF3 | TFPT | 1.290769898 |
| ATF3 | SLC38A2 | 1.277850043 |
| ATF3 | ITGB1 | 1.273549694 |
| ATF3 | MYADM | 1.24556301 |
| ATF3 | RCBTB1 | 1.23910372 |
| ATF3 | PIM2 | 1.227526296 |
| ATF3 | SLN | 1.223372085 |
| ATF3 | ZNF516 | 1.185578344 |
| ATF3 | ARHGAP30 | 1.183098788 |
| ATF3 | SLC23A2 | 1.179950827 |
| ATF3 | CPVL | 1.178939044 |
| ATF3 | DDX5 | 1.178469437 |
| ATF3 | NOLC1 | 1.170176154 |
| ATF3 | APP | 1.169539912 |
| ATF3 | NOP58 | 1.151730091 |
| ATF3 | STOM | 1.137028886 |
| ATF3 | DDIT4 | 1.136492485 |
| ATF3 | TST | 1.127431515 |
| ATF3 | PER1 | 1.114147896 |
| ATF3 | RANGAP1 | 1.109025444 |
| ATF3 | S100A13 | 1.093214196 |
| ATF3 | CD2BP2 | 1.078689584 |
| ATF3 | OGFR | 1.073869906 |
| ATF3 | MAP2K6 | 1.063436547 |
| ATF3 | APCDD1 | 1.059750853 |
| ATF3 | CCBE1 | 1.033562297 |
| ATF3 | NAT10 | 1.032943024 |
| ATF3 | SLC22A15 | 1.032648694 |
| ATF3 | AGTPBP1 | 1.027038226 |
| ATF3 | SIRT5 | 1.024129811 |
| ATF3 | RRAD | 1.023840167 |
| ATF3 | PCGF3 | 1.022536987 |
| ATF3 | TXN | 1.01836876 |
| ATF3 | EIF4EBP2 | 1.014975597 |
| ATF3 | WDYHV1 | 1.014530996 |
| ATF3 | MAEA | 1.010497895 |
| ATF3 | CCNL1 | 1.008652141 |
| ATF3 | ATF3 | 1 |
| ATF3 | VDAC1 | 0.990188697 |
| ATF3 | NCAPH | 0.988947445 |
| ATF3 | ATP8B1 | 0.987310974 |
| ATF3 | FANCM | 0.985831882 |
| ATF3 | AGPAT4 | 0.984478548 |
| ATF3 | NUCKS1 | 0.959936946 |
| ATF3 | HPS4 | 0.956325593 |
| ATF3 | TMEM147 | 0.954045173 |
| ATF3 | FLRT3 | 0.95116645 |
| ATF3 | EIF3H | 0.950095435 |
| ATF3 | PTN | 0.948315718 |
| ATF3 | SLCO4A1 | 0.938303127 |
| ATF3 | IL1R2 | 0.934715874 |
| ATF3 | DAPK2 | 0.923817117 |
| ATF3 | AP2A2 | 0.917254507 |
| ATF3 | ISCA1 | 0.911929448 |
| ATF3 | MAP3K8 | 0.910871893 |
| ATF3 | LBH | 0.910789199 |
| ATF3 | BDKRB1 | 0.909047339 |
| ATF3 | IRF9 | 0.907999958 |
| ATF3 | MEST | 0.900523452 |
| ATF3 | PNRC1 | 0.899803503 |
| ATF3 | TRIB1 | 0.898996387 |
| ATF3 | SERTAD1 | 0.891004985 |
| ATF3 | PARK7 | 0.880890997 |
| ATF3 | RNF39 | 0.880890399 |
| ATF3 | ZFAND5 | 0.863518143 |
| ATF3 | ARFGEF2 | 0.863023195 |
| ATF3 | ISG15 | 0.859988492 |
| ATF3 | RRAGC | 0.859470327 |
| ATF3 | LPIN2 | 0.858534238 |
| ATF3 | EEF1A1 | 0.846320392 |
| ATF3 | CPT1A | 0.839968412 |
| ATF3 | MAST2 | 0.832773012 |
| ATF3 | STRBP | 0.8275744 |
| ATF3 | TUBB2A | 0.827297411 |
| ATF3 | SLC6A6 | 0.827247421 |
| ATF3 | NTNG2 | 0.826297933 |
| ATF3 | TBC1D19 | 0.825162873 |
| ATF3 | COL15A1 | 0.81853332 |
| ATF3 | CAB39 | 0.817141071 |
| ATF3 | FSTL1 | 0.81611643 |
| ATF3 | CDKN2AIP | 0.815542103 |
| ATF3 | CDCA7L | 0.796657039 |
| ATF3 | COL4A2 | 0.796202641 |
| ATF3 | C5orf51 | 0.794733704 |
| ATF3 | NR4A2 | 0.794130667 |
| ATF3 | CITED2 | 0.794111974 |
| ATF3 | PDXDC1 | 0.786939423 |
| ATF3 | AHNAK | 0.779032095 |
| ATF3 | POPDC2 | 0.777239109 |
| ATF3 | WDFY3 | 0.763317596 |
| ATF3 | ARPC3 | 0.763306548 |
| ATF3 | UXT | 0.762376526 |
| ATF3 | GIMAP7 | 0.761762672 |
| ATF3 | PLK2 | 0.761730864 |
| ATF3 | PRKCD | 0.759296215 |
| ATF3 | ADM | 0.758659466 |
| ATF3 | S100A10 | 0.756187064 |
| ATF3 | MTDH | 0.75232396 |
| ATF3 | EPB41L5 | 0.752128342 |
| ATF3 | UBN2 | 0.749130907 |
| ATF3 | TBCC | 0.744385991 |
| ATF3 | TSPAN12 | 0.743512504 |
| ATF3 | UAP1 | 0.740919304 |
| ATF3 | HMOX1 | 0.740275252 |
| ATF3 | HERC1 | 0.736803314 |
| ATF3 | LDB1 | 0.735445131 |
| ATF3 | ATP6AP2 | 0.729994495 |
| ATF3 | TBC1D16 | 0.729438133 |
| ATF3 | SOD3 | 0.716144779 |
| ATF3 | EPOR | 0.713929282 |
| ATF3 | EGR2 | 0.713477345 |
| ATF3 | TIAM1 | 0.713385169 |
| ATF3 | ZNF10 | 0.710107157 |
| ATF3 | SFRP4 | 0.709574691 |
| ATF3 | DSP | 0.709128298 |
| ATF3 | SLC25A3 | 0.708957155 |
| ATF3 | EIF4A2 | 0.708397827 |
| ATF3 | BIRC3 | 0.700864305 |
| ATF3 | FBLN1 | 0.695704115 |
| ATF3 | LIF | 0.694873511 |
| ATF3 | SAV1 | 0.686859546 |
| ATF3 | PIK3CB | 0.68582236 |
| ATF3 | RCN1 | 0.682810086 |
| ATF3 | GIMAP2 | 0.680607064 |
| ATF3 | PCMTD1 | 0.677082503 |
| ATF3 | RAB1A | 0.676829399 |
| ATF3 | CTSB | 0.676250145 |
| ATF3 | SPRR1B | 0.672617979 |
| ATF3 | SET | 0.671017655 |
| ATF3 | PJA2 | 0.667115081 |
| ATF3 | RPSA | 0.664966872 |
| ATF3 | PROX1 | 0.658937814 |
| ATF3 | ORMDL3 | 0.655575117 |
| ATF3 | NRIP2 | 0.655509393 |
| ATF3 | SLC25A33 | 0.654551186 |
| ATF3 | SPATA9 | 0.654010346 |
| ATF3 | RPS7 | 0.653651201 |
| ATF3 | PTK6 | 0.649095535 |
| ATF3 | GNG7 | 0.646109707 |
| ATF3 | ZIC1 | 0.640071419 |
| ATF3 | SPTAN1 | 0.633798671 |
| ATF3 | ZNF462 | 0.6309055 |
| ATF3 | BBOX1 | 0.630585625 |
| ATF3 | STARD8 | 0.628214194 |
| ATF3 | ANKRD46 | 0.626038156 |
| ATF3 | PDLIM3 | 0.624590544 |
| ATF3 | ARHGEF38 | 0.623220797 |
| ATF3 | PALLD | 0.618256709 |
| ATF3 | CYB5R3 | 0.616592718 |
| ATF3 | BMP6 | 0.615592626 |
| ATF3 | KLK10 | 0.614962801 |
| ATF3 | ST6GALNAC2 | 0.610331199 |
| ATF3 | EDNRA | 0.60830365 |
| ATF3 | IDS | 0.606840341 |
| ATF3 | AQP3 | 0.605409375 |
| ATF3 | DDIT3 | 0.605368466 |
| ATF3 | TMBIM6 | 0.603403528 |
| ATF3 | PLD3 | 0.601507897 |
| ATF3 | FCER1A | 0.59679436 |
| ATF3 | KIAA0556 | 0.596395075 |
| ATF3 | BEND3 | 0.595095396 |
| ATF3 | PRNP | 0.593980725 |
| ATF3 | LSP1 | 0.591629296 |
| ATF3 | LOX | 0.591247088 |
| ATF3 | MS4A2 | 0.590250502 |
| ATF3 | RHOB | 0.589665328 |
| ATF3 | CTDSPL | 0.585846808 |
| ATF3 | MFAP5 | 0.584447689 |
| ATF3 | ZNF396 | 0.58211405 |
| ATF3 | SRSF3 | 0.580288567 |
| ATF3 | SCAF1 | 0.580002911 |
| ATF3 | IRGQ | 0.579438741 |
| ATF3 | SFN | 0.574410248 |
| ATF3 | SLC39A1 | 0.570821054 |
| ATF3 | BLNK | 0.5707189 |
| ATF3 | IRX4 | 0.570292056 |
| ATF3 | ENTPD7 | 0.569628575 |
| ATF3 | TNN | 0.569115334 |
| ATF3 | ID1 | 0.567122939 |
| ATF3 | TOX | 0.565443232 |
| ATF3 | NBEAL2 | 0.565185277 |
| ATF3 | CYP4F22 | 0.565172673 |
| ATF3 | ULBP2 | 0.563704258 |
| ATF3 | GPR137B | 0.56312411 |
| ATF3 | RTN4 | 0.562393523 |
| ATF3 | KDR | 0.562308791 |
| ATF3 | ARHGAP23 | 0.560532017 |
| ATF3 | PART1 | 0.560065035 |
| ATF3 | REP15 | 0.552563223 |
| ATF3 | CMTM3 | 0.55069823 |
| ATF3 | ABI3BP | 0.549968584 |
| ATF3 | CHDH | 0.549302295 |
| ATF3 | LRRC20 | 0.548937413 |
| ATF3 | LRCH2 | 0.546173386 |
| ATF3 | SERP1 | 0.546026933 |
| ATF3 | ITGB5 | 0.545467763 |
| ATF3 | DCD | 0.545383144 |
| ATF3 | LPAR3 | 0.539824817 |
| ATF3 | SHB | 0.538831082 |
| ATF3 | C14orf93 | 0.538652432 |
| ATF3 | LAMB4 | 0.536927587 |
| ATF3 | INTU | 0.534214704 |
| ATF3 | ZMYND8 | 0.533311888 |
| ATF3 | WNT9A | 0.533188181 |
| ATF3 | ANXA3 | 0.530831343 |
| ATF3 | S100A2 | 0.528596485 |
| ATF3 | ZNF205 | 0.527726578 |
| ATF3 | AVPI1 | 0.527060864 |
| ATF3 | WDR20 | 0.526293467 |
| ATF3 | LMOD1 | 0.52594049 |
| ATF3 | MFSD6 | 0.525089115 |
| ATF3 | PSAP | 0.524556985 |
| ATF3 | CTGF | 0.523440104 |
| ATF3 | S100A8 | 0.523309731 |
| ATF3 | ADAMTSL1 | 0.521892887 |
| ATF3 | PPP1R15B | 0.521064965 |
| ATF3 | GSN | 0.518648271 |
| ATF3 | FBXL17 | 0.516591986 |
| ATF3 | TNFAIP8L1 | 0.513733855 |
| ATF3 | ADCK1 | 0.512411232 |
| ATF3 | OLFML1 | 0.511181507 |
| ATF3 | WIZ | 0.509422427 |
| ATF3 | TK2 | 0.509077501 |
| ATF3 | SSH2 | 0.508921624 |
| ATF3 | ANK1 | 0.506700187 |
| ATF3 | FAM43A | 0.506645916 |
| ATF3 | SFRP2 | 0.506113942 |
| ATF3 | CACNA2D3 | 0.50541844 |
| ATF3 | SECISBP2L | 0.505404107 |
| ATF3 | RPL31 | 0.504767911 |
| ATF3 | GJB5 | 0.503544501 |
| ATF3 | LCP2 | 0.503231862 |
| ATF3 | INPP5A | 0.502354399 |
| ATF3 | INTS9 | 0.501096186 |
| ATF3 | C20orf27 | 0.498064584 |
| ATF3 | RAB40C | 0.497633127 |
| ATF3 | DENND4B | 0.49751086 |
| ATF3 | MOAP1 | 0.496773205 |
| ATF3 | RHBDF1 | 0.496353908 |
| ATF3 | CTSO | 0.495876452 |
| ATF3 | PRKAG2 | 0.492557987 |
| ATF3 | PRSS12 | 0.492169618 |
| ATF3 | ZSWIM6 | 0.489912307 |
| ATF3 | RPS27A | 0.489634731 |
| ATF3 | CCNA2 | 0.48941991 |
| ATF3 | LAPTM4A | 0.487313898 |
| ATF3 | AIFM2 | 0.487105923 |
| ATF3 | COL4A3BP | 0.485243342 |
| ATF3 | RASGEF1A | 0.485230541 |
| ATF3 | SHE | 0.485133223 |
| ATF3 | AHCYL2 | 0.484642056 |
| ATF3 | ESYT1 | 0.484432037 |
| ATF3 | RGS1 | 0.483689965 |
| ATF3 | DPY19L1P1 | 0.48262317 |
| ATF3 | CD36 | 0.482622969 |
| ATF3 | HTRA3 | 0.48014045 |
| ATF3 | IFI16 | 0.479903413 |
| ATF3 | EEF2 | 0.479855431 |
| ATF3 | VPS26B | 0.479267072 |
| ATF3 | PTPN3 | 0.47911029 |
| ATF3 | PDZD2 | 0.477881566 |
| ATF3 | NAMPT | 0.477713709 |
| ATF3 | CEBPB | 0.477173853 |
| ATF3 | PCDH19 | 0.477052516 |
| ATF3 | NFKBIE | 0.476932438 |
| ATF3 | KIF13A | 0.476236392 |
| ATF3 | ERICH1 | 0.476158117 |
| ATF3 | POGLUT1 | 0.475723646 |
| ATF3 | SFXN5 | 0.474194515 |
| ATF3 | RAB5B | 0.473273223 |
| ATF3 | LXN | 0.47321606 |
| ATF3 | C6orf141 | 0.469650101 |
| ATF3 | ALG6 | 0.469169585 |
| ATF3 | PRSS27 | 0.467914823 |
| ATF3 | LYSMD2 | 0.467908633 |
| ATF3 | GEMIN6 | 0.467407951 |
| ATF3 | APBA1 | 0.467397661 |
| ATF3 | PKP4 | 0.467304603 |
| ATF3 | S100A3 | 0.46667009 |
| ATF3 | SULT1B1 | 0.465890201 |
| ATF3 | CRTAP | 0.464117203 |
| ATF3 | KLC1 | 0.463409249 |
| ATF3 | KRT17 | 0.462389226 |
| ATF3 | MAP2 | 0.457542616 |
| ATF3 | THBS2 | 0.457429466 |
| ATF3 | RPS14 | 0.456757566 |
| ATF3 | EIF5 | 0.455972211 |
| ATF3 | NUP107 | 0.455754258 |
| ATF3 | UBE2QL1 | 0.454490945 |
| ATF3 | IGFBP5 | 0.451670804 |
| ATF3 | CD4 | 0.451252263 |
| ATF3 | ZNF511 | 0.451037766 |
| ATF3 | FN1 | 0.450797775 |
| ATF3 | NINL | 0.45026313 |
| ATF3 | KIF13B | 0.449718255 |
| ATF3 | ATP5S | 0.449490024 |
| ATF3 | TMEM189 | 0.44774172 |
| ATF3 | PMS2 | 0.447538966 |
| ATF3 | SUPT3H | 0.446803607 |
| ATF3 | PLEKHG5 | 0.445277111 |
| ATF3 | INTS6 | 0.444936476 |
| ATF3 | CNOT4 | 0.444623314 |
| ATF3 | ZC4H2 | 0.444610632 |
| ATF3 | DGKH | 0.443262217 |
| ATF3 | DNAJC24 | 0.441536593 |
| ATF3 | PCBP4 | 0.440905489 |
| ATF3 | PRC1 | 0.43936937 |
| ATF3 | KHDRBS3 | 0.438774491 |
| ATF3 | AK5 | 0.438275278 |
| ATF3 | PRRG3 | 0.43785167 |
| ATF3 | SARM1 | 0.436881063 |
| ATF3 | KIF18A | 0.436674267 |
| ATF3 | PRKAR1A | 0.436649024 |
| ATF3 | ALCAM | 0.436613011 |
| ATF3 | PCYT2 | 0.435616143 |
| ATF3 | ITGA3 | 0.435385781 |
| ATF3 | THBS1 | 0.434173915 |
| ATF4 | STXBP3 | 4.478905241 |
| ATF4 | HBP1 | 3.825179217 |
| ATF4 | SNHG5 | 3.678703863 |
| ATF4 | CCNI | 3.374650315 |
| ATF4 | SBDS | 2.917243895 |
| ATF4 | SIVA1 | 2.83706834 |
| ATF4 | CCDC59 | 2.773008397 |
| ATF4 | MAFF | 2.686791939 |
| ATF4 | DDX42 | 2.558553224 |
| ATF4 | RPL27 | 2.548829499 |
| ATF4 | KHDRBS1 | 2.540830739 |
| ATF4 | ASAP1 | 2.529781269 |
| ATF4 | EIF1 | 2.470077638 |
| ATF4 | KLF4 | 2.41770223 |
| ATF4 | GNB1 | 2.399640538 |
| ATF4 | PHIP | 2.327280133 |
| ATF4 | RPL26 | 2.309116911 |
| ATF4 | ICAM2 | 2.291153009 |
| ATF4 | ITM2B | 2.258835019 |
| ATF4 | PRNP | 2.19213062 |
| ATF4 | DNAJC30 | 2.171884226 |
| ATF4 | FAF2 | 2.125130378 |
| ATF4 | EWSR1 | 1.971354329 |
| ATF4 | SRSF5 | 1.964207244 |
| ATF4 | EEF1D | 1.941249896 |
| ATF4 | RGS1 | 1.860863874 |
| ATF4 | MYL12A | 1.860154569 |
| ATF4 | SRSF3 | 1.805230395 |
| ATF4 | HNRNPC | 1.781785294 |
| ATF4 | RPS3A | 1.710704907 |
| ATF4 | CCT4 | 1.666596877 |
| ATF4 | KLF10 | 1.621312645 |
| ATF4 | SNRPD2 | 1.607180179 |
| ATF4 | NID2 | 1.55828268 |
| ATF4 | GPX4 | 1.52939732 |
| ATF4 | CDCA5 | 1.516239786 |
| ATF4 | SOD1 | 1.499683098 |
| ATF4 | EIF5 | 1.411786805 |
| ATF4 | UBQLN2 | 1.409818091 |
| ATF4 | SPC25 | 1.377282411 |
| ATF4 | DGKD | 1.369588686 |
| ATF4 | ANK2 | 1.346302657 |
| ATF4 | NCOA6 | 1.339827526 |
| ATF4 | TPST1 | 1.332646881 |
| ATF4 | KIF11 | 1.327925693 |
| ATF4 | TUBB | 1.32620844 |
| ATF4 | RRM2 | 1.27805604 |
| ATF4 | ZC3H4 | 1.277200153 |
| ATF4 | MRPL41 | 1.241868496 |
| ATF4 | ZBTB20 | 1.188265463 |
| ATF4 | SAP18 | 1.180229083 |
| ATF4 | DHX33 | 1.174087084 |
| ATF4 | EEF1B2 | 1.159718473 |
| ATF4 | CENPW | 1.158773154 |
| ATF4 | LRFN4 | 1.151594675 |
| ATF4 | SKIL | 1.150030216 |
| ATF4 | RAPGEF1 | 1.129519369 |
| ATF4 | TUBGCP4 | 1.124004132 |
| ATF4 | DENND4A | 1.094424255 |
| ATF4 | SRSF6 | 1.065692276 |
| ATF4 | CFL1 | 1.047093629 |
| ATF4 | UBE2J1 | 1.038819759 |
| ATF4 | EFR3A | 1.037491826 |
| ATF4 | CA5B | 1.029502278 |
| ATF4 | S1PR2 | 1.027264302 |
| ATF4 | ATF4 | 1 |
| ATF5 | RREB1 | 3.878680794 |
| ATF5 | SMARCA5 | 2.136191503 |
| ATF5 | MED30 | 2.032502483 |
| ATF5 | HLA-C | 1.818658305 |
| ATF5 | CREB3L2 | 1.452672789 |
| ATF5 | B4GALT3 | 1.408177071 |
| ATF5 | ZER1 | 1.374872519 |
| ATF5 | CLK4 | 1.331515084 |
| ATF5 | ARPC5 | 1.329962658 |
| ATF5 | HDAC11 | 1.257265634 |
| ATF5 | NUPL2 | 1.175298891 |
| ATF5 | RRAS2 | 1.099599749 |
| ATF5 | LSM5 | 1.064989569 |
| ATF5 | THRAP3 | 1.061074395 |
| ATF5 | UNKL | 1.011765672 |
| ATF5 | RELA | 1.006804076 |
| ATF5 | KIF13B | 1.006744446 |
| ATF5 | GMEB2 | 0.851805994 |
| ATF5 | TSC22D3 | 0.829186926 |
| ATF5 | TAPT1 | 0.821044838 |
| ATF5 | ENTPD4 | 0.816322046 |
| ATF5 | RPS6KA3 | 0.780517877 |
| ATF5 | TAGLN2 | 0.749592908 |
| ATF5 | CSTF3 | 0.693301182 |
| ATF5 | TRA2B | 0.594763383 |
| ATF5 | XRCC6 | 0.533625874 |
| ATF5 | CDK2AP2 | 0.525911814 |
| ATF5 | AHCYL1 | 0.520493117 |
| ATF5 | HIST1H4H | 0.514029466 |
| ATF5 | LMTK2 | 0.406717999 |
| ATF5 | DTX3 | 0.359020803 |
| ATF5 | TRPC1 | 0.319497615 |
| ATF5 | PEX11G | 0.288252558 |
| ATF5 | RAB25 | 0.236565141 |
| ATF5 | TGFB3 | 0.224460144 |
| ATF5 | IFT172 | 0.213515556 |
| ATF5 | CYCS | 0.18811693 |
| ATF5 | BET1 | 0.179673693 |
| ATF5 | SSSCA1 | 0.178739784 |
| ATF5 | CD2AP | 0.16891779 |
| ATF5 | RCE1 | 0.109562834 |
| ATF5 | FTH1 | 0.106046432 |
| ATF5 | GLYR1 | 0.000379307 |
| ATF5 | SEC24A | 2.23E-06 |
| ATF6 | COL11A1 | 3.720995288 |
| ATF6 | TRMT1 | 2.480048725 |
| ATF6 | MSRB3 | 2.375042581 |
| ATF6 | LAMC2 | 2.148883611 |
| ATF6 | ZNF696 | 1.883422556 |
| ATF6 | GAPDH | 1.76158627 |
| ATF6 | NFIB | 1.753943958 |
| ATF6 | LZTFL1 | 1.740925163 |
| ATF6 | ETF1 | 1.729589847 |
| ATF6 | NOL7 | 1.72401433 |
| ATF6 | TM9SF3 | 1.628669498 |
| ATF6 | ZFAND1 | 1.615663079 |
| ATF6 | MECR | 1.584530849 |
| ATF6 | INO80D | 1.561313492 |
| ATF6 | BASP1 | 1.528507416 |
| ATF6 | KCNK6 | 1.423764458 |
| ATF6 | RAB21 | 1.383746993 |
| ATF6 | UBC | 1.362362303 |
| ATF6 | TMEM108 | 1.30769312 |
| ATF6 | TPSB2 | 1.298752108 |
| ATF6 | DHX40 | 1.230386342 |
| ATF6 | LOXL2 | 1.167911216 |
| ATF6 | BPTF | 1.12375704 |
| ATF6 | GADD45A | 1.066133191 |
| ATF6 | ATF6 | 1 |
| ATF6 | RNF128 | 0.97201115 |
| ATF6 | NAT10 | 0.934422665 |
| ATF6 | FAM168A | 0.901429488 |
| ATF6 | WSB2 | 0.876934059 |
| ATF6 | COL25A1 | 0.828348504 |
| ATF6 | SCARA3 | 0.774254398 |
| ATF6 | MUC1 | 0.758297663 |
| ATF6 | SLC10A7 | 0.724188602 |
| ATF6 | CDCA5 | 0.721632061 |
| ATF6 | SYN1 | 0.644355201 |
| ATF6 | TSC1 | 0.582158095 |
| ATF6 | MTCH1 | 0.578330383 |
| ATF6 | OVOL1 | 0.561725454 |
| ATF6 | OCIAD1 | 0.560992664 |
| ATF6 | ARPP19 | 0.560761406 |
| ATF6 | TMOD3 | 0.546918379 |
| ATF6 | FSCN1 | 0.525102715 |
| ATF6 | GOLIM4 | 0.519678235 |
| ATF6 | IDH3G | 0.503320693 |
| ATF6 | STC2 | 0.477233438 |
| ATF6 | DTNA | 0.471443067 |
| ATF6 | UBXN6 | 0.460418773 |
| ATF6 | GRP | 0.445806097 |
| ATF6 | LARP7 | 0.439025425 |
| ATF6 | MAP4 | 0.400024107 |
| ATF6 | ST20 | 0.397201752 |
| ATF6 | SLC6A9 | 0.340969053 |
| ATF6 | ZHX2 | 0.328712613 |
| ATF6 | FOXP2 | 0.322981241 |
| ATF6 | SMARCD3 | 0.29807683 |
| ATF6 | HAUS5 | 0.293909273 |
| ATF6 | TMTC4 | 0.289453111 |
| ATF6 | DLG4 | 0.288528219 |
| ATF6 | ETV2 | 0.286936375 |
| ATF6 | MTSS1L | 0.280787353 |
| ATF6 | CACNB2 | 0.28056744 |
| ATF6 | BTAF1 | 0.27087451 |
| ATF6 | PRR3 | 0.266087553 |
| ATF6 | PPIL4 | 0.257289454 |
| ATF6 | ARMCX2 | 0.256810537 |
| ATF6 | ZBTB5 | 0.244559558 |
| ATF6 | SLC35B1 | 0.231988315 |
| ATF6 | ZFYVE27 | 0.220053435 |
| ATF6 | NXPH4 | 0.205501459 |
| ATF6 | HSPA1L | 0.184781785 |
| ATF6 | PAK1 | 0.177132786 |
| ATF6 | CDKN2B | 0.17124712 |
| ATF6 | PLCXD3 | 0.170667256 |
| ATF6 | GSC | 0.15603542 |
| ATF6 | ZIC1 | 0.15052754 |
| ATF6 | SEZ6L2 | 0.149914304 |
| ATF6 | ZNF200 | 0.141346713 |
| ATF6 | ZNF496 | 0.12898121 |
| ATF6 | ATP9B | 0.126171278 |
| ATF6 | PHF8 | 0.10189119 |
| ATF6 | MEX3C | 0.022184669 |
| ATF6 | AHDC1 | 0.003029661 |
| BACH1 | WWC3 | 7.734621571 |
| BACH1 | VDAC1 | 7.578628008 |
| BACH1 | KCNMA1 | 2.999611879 |
| BACH1 | PTK2B | 2.537308902 |
| BACH1 | MTUS1 | 2.14678934 |
| BACH1 | PISD | 2.038050477 |
| BACH1 | IGF1R | 1.558168744 |
| BACH1 | GTF2H1 | 1.441298541 |
| BACH1 | S1PR1 | 1.346202541 |
| BACH1 | CDC42EP3 | 1.299732804 |
| BACH1 | RNF24 | 1.29657983 |
| BACH1 | EIF5A2 | 1.278283836 |
| BACH1 | ITPRIP | 1.168524559 |
| BACH1 | PRICKLE1 | 1.125110399 |
| BACH1 | PPP2R5C | 1.063480948 |
| BACH1 | PIM1 | 1.061212324 |
| BACH1 | FRMD4A | 1.039648501 |
| BACH1 | TIMM22 | 1.015691461 |
| BACH1 | BACH1 | 1 |
| BACH1 | DEPDC1 | 0.997872717 |
| BACH1 | GPT2 | 0.918554401 |
| BACH1 | DENND1B | 0.887598596 |
| BACH1 | MCC | 0.886445097 |
| BACH1 | PDHA1 | 0.863985214 |
| BACH1 | EIF2AK2 | 0.843566516 |
| BACH1 | BMP7 | 0.832814824 |
| BACH1 | PGRMC1 | 0.823982044 |
| BACH1 | ORAI2 | 0.814726941 |
| BACH1 | KLF9 | 0.781190935 |
| BACH1 | SHB | 0.766087751 |
| BACH1 | NFIX | 0.759712484 |
| BACH1 | ATP6V1A | 0.754382883 |
| BACH1 | RPL17 | 0.752865016 |
| BACH1 | PRDM10 | 0.749201396 |
| BACH1 | TCEAL2 | 0.744688516 |
| BACH1 | LDHA | 0.707940999 |
| BACH1 | NFKBIZ | 0.661054577 |
| BACH1 | WBP2 | 0.651822878 |
| BACH1 | SEC23A | 0.648549774 |
| BACH1 | PRDM2 | 0.621016981 |
| BACH1 | ZNF385B | 0.601225946 |
| BACH1 | DCAF8 | 0.582580627 |
| BACH1 | RAB1A | 0.56697 |
| BACH1 | NAA50 | 0.552566011 |
| BACH1 | EPHB4 | 0.546231619 |
| BACH1 | U2AF2 | 0.517129908 |
| BACH1 | TPP1 | 0.511839193 |
| BACH1 | NEGR1 | 0.506746134 |
| BACH1 | FEN1 | 0.499675399 |
| BACH1 | DCP1B | 0.497766727 |
| BACH1 | CTNS | 0.487740322 |
| BACH1 | PPM1B | 0.473713501 |
| BACH1 | CUL4B | 0.463038662 |
| BACH1 | ACSM3 | 0.46200014 |
| BACH1 | HDAC4 | 0.461262277 |
| BACH1 | EGR2 | 0.460209068 |
| BACH2 | DCTN4 | 5.816920178 |
| BACH2 | BNIP1 | 2.794732583 |
| BACH2 | TMF1 | 2.67180451 |
| BACH2 | OXSR1 | 2.333274075 |
| BACH2 | PRKAG1 | 2.329724753 |
| BACH2 | UBL3 | 2.296744973 |
| BACH2 | TM9SF3 | 2.205223612 |
| BACH2 | SULF2 | 2.150650842 |
| BACH2 | EEF1E1 | 2.11832562 |
| BACH2 | KIAA0141 | 1.848514962 |
| BACH2 | PRSS35 | 1.64400349 |
| BACH2 | RNMT | 1.585065344 |
| BACH2 | DSTYK | 1.523174808 |
| BACH2 | SUMO3 | 1.475534883 |
| BACH2 | ILF3 | 1.470031543 |
| BACH2 | RAB10 | 1.367166962 |
| BACH2 | MEST | 1.312392429 |
| BACH2 | GPD2 | 1.132541886 |
| BACH2 | KLHL20 | 1.090169204 |
| BACH2 | ANXA2P2 | 1.081307413 |
| BACH2 | TMBIM1 | 1.05818065 |
| BACH2 | BACH2 | 1 |
| BATF | GPR183 | 3.38427389 |
| BATF | MXD1 | 2.767705847 |
| BATF | ACVR1B | 2.450710181 |
| BATF | ADNP2 | 1.849007778 |
| BATF | CD37 | 1.645687106 |
| BATF | TP63 | 1.569762569 |
| BATF | MAP2K3 | 1.301335066 |
| BATF | PPP1R14C | 1.288291727 |
| BATF | PPIG | 1.227177059 |
| BATF | NOTCH3 | 1.160032692 |
| BATF | COL6A6 | 1.124588721 |
| BATF | CD59 | 1.057861586 |
| BATF | BATF | 1 |
| BATF | TNFRSF21 | 0.813419737 |
| BATF | NTNG2 | 0.811400028 |
| BATF | EIF2S3 | 0.356839615 |
| BATF | RHEB | 0.289676008 |
| BATF | AIG1 | 0.225532578 |
| BATF3 | SGSM2 | 5.970088096 |
| BATF3 | CSRP2 | 5.425350656 |
| BATF3 | NUP98 | 4.789866111 |
| BATF3 | IL32 | 4.573445501 |
| BATF3 | GCH1 | 4.243003289 |
| BATF3 | CDYL | 3.908830134 |
| BATF3 | CCDC47 | 3.540945946 |
| BATF3 | MRPS18C | 3.371608564 |
| BATF3 | UBQLN1 | 3.178275865 |
| BATF3 | ZNF330 | 3.067341902 |
| BATF3 | PRCP | 2.997906684 |
| BATF3 | GOSR2 | 2.72596509 |
| BATF3 | PARP2 | 2.662141971 |
| BATF3 | APPBP2 | 2.617007234 |
| BATF3 | FAM177A1 | 2.435820377 |
| BATF3 | INF2 | 2.410927398 |
| BATF3 | P4HTM | 2.128660588 |
| BATF3 | RAB27A | 2.099265245 |
| BATF3 | HIGD2A | 2.096018882 |
| BATF3 | ABHD2 | 2.086796887 |
| BATF3 | NOP16 | 2.060637031 |
| BATF3 | ALDH7A1 | 1.945004057 |
| BATF3 | IL6 | 1.913847755 |
| BATF3 | RPA2 | 1.681487824 |
| BATF3 | NOC4L | 1.601230187 |
| BATF3 | HSPA9 | 1.467240708 |
| BATF3 | SNCAIP | 1.188266354 |
| BATF3 | RFC2 | 1.073020639 |
| BATF3 | BATF3 | 1 |
| BATF3 | KCNT2 | 0.415056846 |
| BCL11A | CD83 | 2.106336556 |
| BCL11A | YEATS4 | 2.024256035 |
| BCL11A | HM13 | 1.943626539 |
| BCL11A | TUBB2A | 1.497891668 |
| BCL11A | ELOVL6 | 1.433906667 |
| BCL11A | DYNC1I2 | 1.324374275 |
| BCL11A | ARPC1B | 1.274239558 |
| BCL11A | BCL9 | 1.161896279 |
| BCL11A | BCL11A | 1 |
| BCL3 | CTSH | 12.30475141 |
| BCL3 | GGT5 | 10.56592888 |
| BCL3 | CCL19 | 9.600429912 |
| BCL3 | C3 | 8.141789438 |
| BCL3 | VEGFA | 7.058653826 |
| BCL3 | PDGFRB | 5.282899398 |
| BCL3 | OAF | 4.870385338 |
| BCL3 | CD82 | 4.229108129 |
| BCL3 | ATP11C | 3.33437441 |
| BCL3 | CLSTN3 | 2.949318258 |
| BCL3 | YWHAZ | 2.782595158 |
| BCL3 | ABCA8 | 2.622226164 |
| BCL3 | POR | 2.534012778 |
| BCL3 | GPX1 | 2.436495561 |
| BCL3 | C7 | 2.36842624 |
| BCL3 | BATF3 | 2.307014393 |
| BCL3 | EIF5 | 2.158941548 |
| BCL3 | PGF | 2.117145879 |
| BCL3 | GDAP1 | 2.101628152 |
| BCL3 | NFKBIZ | 2.081042484 |
| BCL3 | DNAJB1 | 2.050927182 |
| BCL3 | TNFSF10 | 1.935070612 |
| BCL3 | SLC38A2 | 1.899582348 |
| BCL3 | SPHK1 | 1.896762252 |
| BCL3 | IFITM2 | 1.805067802 |
| BCL3 | TNFAIP2 | 1.711957217 |
| BCL3 | TEAD4 | 1.686319775 |
| BCL3 | MGST3 | 1.679276866 |
| BCL3 | ABI3BP | 1.670625091 |
| BCL3 | YPEL3 | 1.653950658 |
| BCL3 | CXXC5 | 1.629704108 |
| BCL3 | ICAM1 | 1.531930927 |
| BCL3 | PLK1 | 1.512046457 |
| BCL3 | ABCA2 | 1.494033572 |
| BCL3 | ANXA1 | 1.492683735 |
| BCL3 | ATP6V1C2 | 1.486814652 |
| BCL3 | SNED1 | 1.479586889 |
| BCL3 | NGFR | 1.461739508 |
| BCL3 | NLRC5 | 1.448748057 |
| BCL3 | CDYL | 1.439257354 |
| BCL3 | NDRG1 | 1.389779052 |
| BCL3 | PDLIM4 | 1.363275384 |
| BCL3 | MBNL1 | 1.273996405 |
| BCL3 | FKBP5 | 1.272409937 |
| BCL3 | INF2 | 1.209597627 |
| BCL3 | UBQLN1 | 1.207160614 |
| BCL3 | CXCL2 | 1.194276541 |
| BCL3 | TRAF3IP2 | 1.171385371 |
| BCL3 | SMG1 | 1.15459543 |
| BCL3 | EVPL | 1.092176259 |
| BCL3 | ADAMTS4 | 1.056368704 |
| BCL3 | IL13RA1 | 1.037949407 |
| BCL3 | BCL3 | 1 |
| BCL3 | IL6 | 0.99580847 |
| BCL3 | KRT31 | 0.971892263 |
| BCL3 | SOCS3 | 0.937547094 |
| BCL3 | HOXA10 | 0.907867182 |
| BCL3 | RAB27A | 0.879303172 |
| BCL3 | CRABP2 | 0.856144601 |
| BCL3 | KIF18B | 0.827246029 |
| BCL3 | SVEP1 | 0.817430238 |
| BCL3 | PSME2 | 0.817000736 |
| BCL3 | EPHA2 | 0.772506814 |
| BCL3 | CCL2 | 0.771004586 |
| BCL3 | FBN1 | 0.751720287 |
| BCL3 | SNRPD1 | 0.735566095 |
| BCL3 | SH3KBP1 | 0.729827159 |
| BCL3 | OSMR | 0.721408367 |
| BCL3 | CTHRC1 | 0.720767199 |
| BCL3 | TNFAIP3 | 0.686394001 |
| BCL3 | IMMP2L | 0.680883953 |
| BCL3 | TFAP4 | 0.679035144 |
| BCL3 | HLA-C | 0.670030357 |
| BCL3 | LST1 | 0.652027729 |
| BCL3 | PTCH1 | 0.646437783 |
| BCL3 | HLA-B | 0.631829668 |
| BCL3 | CALM1 | 0.611899368 |
| BCL3 | PTX3 | 0.609012743 |
| BCL3 | EFEMP1 | 0.602450766 |
| BCL3 | CDKN1A | 0.601572332 |
| BCL3 | PMP22 | 0.586886226 |
| BCL3 | RFTN1 | 0.576840402 |
| BCL3 | SP100 | 0.571474178 |
| BCL3 | ABHD2 | 0.552783623 |
| BCL3 | CLDN11 | 0.534668349 |
| BCL3 | RPL19 | 0.53127055 |
| BCL3 | ADRB2 | 0.506735531 |
| BCL3 | SERPINE2 | 0.495415505 |
| BCL3 | ATF3 | 0.488635714 |
| BCL3 | IL15 | 0.486113357 |
| BCL3 | PRRX1 | 0.463781825 |
| BCL3 | ICA1 | 0.461426163 |
| BCL3 | PEA15 | 0.453468503 |
| BCL3 | RHOF | 0.446495688 |
| BCL3 | CLU | 0.436749466 |
| BCL3 | VCAN | 0.43533439 |
| BCL3 | TFPI | 0.433660915 |
| BCL6B | JUP | 1.684313707 |
| BCL6B | CPSF6 | 1.52990509 |
| BCL6B | SYT8 | 1.313115073 |
| BCL6B | ROBO1 | 1.230938164 |
| BCL6B | SIPA1L1 | 1.116109212 |
| BCL6B | PLXNB2 | 0.998945822 |
| BCLAF1 | MOCS3 | 6.250988533 |
| BCLAF1 | IKBIP | 6.079029328 |
| BCLAF1 | SHISA5 | 5.401870221 |
| BCLAF1 | CFL1 | 5.057957642 |
| BCLAF1 | MEA1 | 4.822227783 |
| BCLAF1 | TNFRSF19 | 4.733012204 |
| BCLAF1 | RGS19 | 4.694196966 |
| BCLAF1 | RNF144B | 4.657167648 |
| BCLAF1 | CCDC47 | 4.64301746 |
| BCLAF1 | NXT1 | 4.469972334 |
| BCLAF1 | TIMP2 | 4.455014399 |
| BCLAF1 | WRN | 4.367115618 |
| BCLAF1 | NRAS | 4.238190721 |
| BCLAF1 | NBR1 | 4.15627295 |
| BCLAF1 | PHTF2 | 3.933273018 |
| BCLAF1 | AVPI1 | 3.781427527 |
| BCLAF1 | ATAD1 | 3.323884432 |
| BCLAF1 | EDN1 | 3.299775967 |
| BCLAF1 | COX6B1 | 3.254565308 |
| BCLAF1 | CCT2 | 3.253939575 |
| BCLAF1 | COL8A1 | 3.220074311 |
| BCLAF1 | YWHAZ | 3.149122074 |
| BCLAF1 | HNRNPU | 2.904765642 |
| BCLAF1 | UBE2D1 | 2.809596414 |
| BCLAF1 | DOCK1 | 2.787502554 |
| BCLAF1 | H1F0 | 2.679477474 |
| BCLAF1 | CRKL | 2.563370788 |
| BCLAF1 | SLC43A1 | 2.497660969 |
| BCLAF1 | NFIC | 2.32685556 |
| BCLAF1 | ITM2C | 2.201619569 |
| BCLAF1 | NID2 | 2.167178612 |
| BCLAF1 | RAB1B | 2.141625688 |
| BCLAF1 | KIFC1 | 2.12972807 |
| BCLAF1 | ATF7IP | 2.114164115 |
| BCLAF1 | CREBL2 | 2.066384109 |
| BCLAF1 | SNAP23 | 2.051798975 |
| BCLAF1 | DDOST | 2.028522479 |
| BCLAF1 | TCF4 | 1.989758366 |
| BCLAF1 | SFRP1 | 1.930740255 |
| BCLAF1 | GNAI1 | 1.926393721 |
| BCLAF1 | OSBPL11 | 1.886510718 |
| BCLAF1 | CA12 | 1.872890683 |
| BCLAF1 | UFM1 | 1.850996708 |
| BCLAF1 | TPM4 | 1.793046266 |
| BCLAF1 | S100A2 | 1.750218162 |
| BCLAF1 | HAPLN1 | 1.714034863 |
| BCLAF1 | AHR | 1.710704546 |
| BCLAF1 | MAP3K8 | 1.69546441 |
| BCLAF1 | KDELC2 | 1.682288994 |
| BCLAF1 | CDC20 | 1.667792563 |
| BCLAF1 | ARRDC3 | 1.640959835 |
| BCLAF1 | HK1 | 1.640848524 |
| BCLAF1 | GTF3A | 1.620432246 |
| BCLAF1 | ARID5B | 1.584239799 |
| BCLAF1 | YPEL5 | 1.564181571 |
| BCLAF1 | TRRAP | 1.561369577 |
| BCLAF1 | CDCA4 | 1.364490776 |
| BCLAF1 | RAB5C | 1.354743072 |
| BCLAF1 | H3F3B | 1.352926637 |
| BCLAF1 | PPIA | 1.313906338 |
| BCLAF1 | EIF1AX | 1.310644448 |
| BCLAF1 | RPL21 | 1.284862757 |
| BCLAF1 | TAGLN2 | 1.276048442 |
| BCLAF1 | KLHL35 | 1.261904498 |
| BCLAF1 | PGAP2 | 1.231401823 |
| BCLAF1 | MYL12A | 1.206844481 |
| BCLAF1 | SLC39A14 | 1.19046509 |
| BCLAF1 | ARL3 | 1.171081102 |
| BCLAF1 | SFPQ | 1.163277509 |
| BCLAF1 | TPI1 | 1.145672833 |
| BCLAF1 | NAMPT | 1.137150741 |
| BCLAF1 | LDHA | 1.13610881 |
| BCLAF1 | UBA52 | 1.118166982 |
| BCLAF1 | HNRNPA2B1 | 1.111535561 |
| BCLAF1 | RPS12 | 1.06326682 |
| BCLAF1 | NDUFB6 | 1.014647302 |
| BCLAF1 | BCLAF1 | 1 |
| BCLAF1 | TMED10 | 0.991029603 |
| BCLAF1 | CD9 | 0.984641458 |
| BCLAF1 | MYBL2 | 0.954940576 |
| BCLAF1 | FGFR1 | 0.916608741 |
| BCLAF1 | HMGB1 | 0.916023096 |
| BCLAF1 | NFKBIA | 0.892193212 |
| BCLAF1 | DAB2 | 0.878631442 |
| BCLAF1 | UBE2T | 0.875456744 |
| BCLAF1 | CALM1 | 0.863241416 |
| BCLAF1 | DDT | 0.851470055 |
| BCLAF1 | MTIF3 | 0.845339312 |
| BCLAF1 | POC1A | 0.843564365 |
| BCLAF1 | CXorf38 | 0.837724163 |
| BCLAF1 | SLK | 0.805785519 |
| BCLAF1 | DPYSL2 | 0.803235814 |
| BCLAF1 | TSC22D3 | 0.802627145 |
| BCLAF1 | PDIK1L | 0.800928345 |
| BCLAF1 | UBE2D3 | 0.798978448 |
| BCLAF1 | MORF4L1 | 0.793930173 |
| BCLAF1 | HSP90AB1 | 0.776728025 |
| BCLAF1 | NUDT1 | 0.766053839 |
| BCLAF1 | DLG4 | 0.750381786 |
| BCLAF1 | PLK1 | 0.741875593 |
| BCLAF1 | SAT1 | 0.736784919 |
| BCLAF1 | MPZL1 | 0.736660802 |
| BCLAF1 | ABCE1 | 0.736153589 |
| BCLAF1 | CCDC40 | 0.732845705 |
| BCLAF1 | SPC25 | 0.724780148 |
| BCLAF1 | RAN | 0.723717304 |
| BCLAF1 | CNNM3 | 0.717361885 |
| BCLAF1 | PNRC1 | 0.714408961 |
| BCLAF1 | TCEA3 | 0.702949727 |
| BCLAF1 | TRMT61A | 0.699677461 |
| BCLAF1 | PAK6 | 0.690468604 |
| BCLAF1 | TAF1A | 0.689003273 |
| BCLAF1 | USP45 | 0.682947005 |
| BCLAF1 | TOMM7 | 0.679060358 |
| BCLAF1 | PAQR5 | 0.675410399 |
| BCLAF1 | ARPC2 | 0.659976893 |
| BCLAF1 | SRCAP | 0.653716796 |
| BCLAF1 | MAP1B | 0.651151759 |
| BCLAF1 | STAB1 | 0.647957968 |
| BCLAF1 | RBBP7 | 0.647718333 |
| BCLAF1 | DHODH | 0.639435659 |
| BCLAF1 | AURKAIP1 | 0.634162687 |
| BCLAF1 | MBOAT1 | 0.632631441 |
| BCLAF1 | ADAM12 | 0.624272445 |
| BCLAF1 | CACNA1A | 0.60795388 |
| BCLAF1 | JUND | 0.606800864 |
| BCLAF1 | MLH3 | 0.605938046 |
| BCLAF1 | ILF2 | 0.601943282 |
| BCLAF1 | MAN2A2 | 0.600119976 |
| BCLAF1 | RPS3A | 0.590296372 |
| BCLAF1 | NR4A1 | 0.590144131 |
| BCLAF1 | THAP11 | 0.586180663 |
| BCLAF1 | SMYD2 | 0.584455374 |
| BCLAF1 | PROX1 | 0.581905828 |
| BCLAF1 | MSL2 | 0.573807658 |
| BCLAF1 | LAPTM4A | 0.566280673 |
| BCLAF1 | DNAJA1 | 0.56433883 |
| BCLAF1 | RBMS1 | 0.563673312 |
| BCLAF1 | NDUFB10 | 0.562906879 |
| BCLAF1 | SEC61G | 0.558985849 |
| BCLAF1 | SLC25A3 | 0.553844318 |
| BCLAF1 | LGI2 | 0.541546611 |
| BCLAF1 | AHNAK | 0.538730983 |
| BCLAF1 | IRF1 | 0.536344885 |
| BCLAF1 | NBEAL2 | 0.532240768 |
| BCLAF1 | PLXNA2 | 0.521477166 |
| BCLAF1 | ELMO2 | 0.519093342 |
| BCLAF1 | SNAPC4 | 0.512688514 |
| BCLAF1 | PDRG1 | 0.512235547 |
| BCLAF1 | ZNF213 | 0.510877864 |
| BCLAF1 | RWDD2A | 0.505676335 |
| BCLAF1 | ARHGEF17 | 0.492026732 |
| BCLAF1 | ZNF805 | 0.48656522 |
| BCLAF1 | MT2A | 0.486258876 |
| BCLAF1 | EMP3 | 0.482119797 |
| BCLAF1 | EEF1D | 0.478687139 |
| BCLAF1 | SPON1 | 0.476851391 |
| BCLAF1 | RIF1 | 0.476413576 |
| BCLAF1 | AXIN1 | 0.474038694 |
| BCLAF1 | HNRNPA1 | 0.473534754 |
| BCLAF1 | RPL13A | 0.472928179 |
| BCLAF1 | B3GALNT2 | 0.469268683 |
| BCLAF1 | TMED4 | 0.467084899 |
| BCLAF1 | CSNK1E | 0.466175593 |
| BCLAF1 | ADAL | 0.464344825 |
| BCLAF1 | PARP3 | 0.462332339 |
| BCLAF1 | TJAP1 | 0.459791803 |
| BCLAF1 | ELOVL2 | 0.456260063 |
| BCLAF1 | PTCH1 | 0.451708657 |
| BCLAF1 | IL6ST | 0.451075265 |
| BCLAF1 | RAD21 | 0.445413985 |
| BCLAF1 | KAT2B | 0.439255182 |
| BCLAF1 | CCNI | 0.439108177 |
| BCLAF1 | BRI3BP | 0.438854455 |
| BCLAF1 | DNAJC10 | 0.438677433 |
| BCLAF1 | TTC13 | 0.435969594 |
| BCLAF1 | DBI | 0.429334844 |
| BCLAF1 | PKP2 | 0.425970866 |
| BCLAF1 | ASB1 | 0.412620965 |
| BCLAF1 | ESRRG | 0.410768934 |
| BCLAF1 | SASH1 | 0.409079292 |
| BCLAF1 | TRIM8 | 0.408718118 |
| BCLAF1 | ZC3H6 | 0.396457228 |
| BCLAF1 | TBC1D13 | 0.396187008 |
| BCLAF1 | NR2F6 | 0.391497123 |
| BCLAF1 | STOX2 | 0.38005867 |
| BCLAF1 | ZBTB44 | 0.368617159 |
| BCLAF1 | NXPH3 | 0.365865129 |
| BCLAF1 | CCT6P3 | 0.360713247 |
| BCLAF1 | PHKA2 | 0.354964122 |
| BCLAF1 | RAI14 | 0.354358589 |
| BCLAF1 | CAMK2G | 0.353322613 |
| BCLAF1 | GTF2IRD1 | 0.344992694 |
| BCLAF1 | PATZ1 | 0.343728634 |
| BCLAF1 | ORMDL3 | 0.340707157 |
| BCLAF1 | LPAR2 | 0.335190508 |
| BCLAF1 | ADAMTS8 | 0.331616621 |
| BCLAF1 | FOXP2 | 0.32953273 |
| BCLAF1 | VGLL4 | 0.32751392 |
| BCLAF1 | NKX3-1 | 0.327279083 |
| BCLAF1 | NDUFC1 | 0.322099541 |
| BCLAF1 | PID1 | 0.316069331 |
| BCLAF1 | EYA4 | 0.31313376 |
| BCLAF1 | NTN1 | 0.30856017 |
| BCLAF1 | HOXA5 | 0.308424621 |
| BCLAF1 | MAGI2 | 0.306993556 |
| BCLAF1 | FOXP4 | 0.305221755 |
| BCLAF1 | SERPINB1 | 0.305178733 |
| BCLAF1 | NFKB1 | 0.304740955 |
| BCLAF1 | COQ10B | 0.300384214 |
| BCLAF1 | COASY | 0.298487424 |
| BCLAF1 | ARL4C | 0.297920459 |
| BCLAF1 | ZNF496 | 0.297117363 |
| BCLAF1 | CSDE1 | 0.293900801 |
| BCLAF1 | FRAT2 | 0.290473993 |
| BCLAF1 | RB1 | 0.286976065 |
| BCLAF1 | MYO1D | 0.283566608 |
| BCLAF1 | COPS7A | 0.277725694 |
| BCLAF1 | TIAM1 | 0.273591488 |
| BCLAF1 | SRC | 0.273272814 |
| BCLAF1 | HOXA9 | 0.272941079 |
| BCLAF1 | LDLRAD3 | 0.255010919 |
| BCLAF1 | SLC22A15 | 0.252687463 |
| BCLAF1 | NR3C1 | 0.252648511 |
| BCLAF1 | ZMYND19 | 0.252443035 |
| BCLAF1 | GCC1 | 0.249681576 |
| BCLAF1 | PRTG | 0.249063647 |
| BCLAF1 | TFEB | 0.247700626 |
| BCLAF1 | UFSP1 | 0.247414732 |
| BCLAF1 | AQP3 | 0.24718508 |
| BCLAF1 | TGM2 | 0.243706051 |
| BCLAF1 | SS18L1 | 0.242684059 |
| BCLAF1 | SCMH1 | 0.242588854 |
| BCLAF1 | ATN1 | 0.242351205 |
| BCLAF1 | USP24 | 0.241621528 |
| BCLAF1 | RBM12 | 0.240327303 |
| BCLAF1 | TCOF1 | 0.239693839 |
| BCLAF1 | EBAG9 | 0.238998264 |
| BCLAF1 | RFC4 | 0.23872501 |
| BCLAF1 | CLDN23 | 0.235523332 |
| BCLAF1 | CCT6P1 | 0.233254747 |
| BCLAF1 | ADCY2 | 0.229837844 |
| BCLAF1 | C12orf75 | 0.226697405 |
| BCLAF1 | C11orf95 | 0.222141692 |
| BCLAF1 | TUG1 | 0.21752558 |
| BCLAF1 | ABCB4 | 0.216895954 |
| BCLAF1 | FOXF2 | 0.211394872 |
| BCLAF1 | SLC16A3 | 0.207311964 |
| BCLAF1 | PLXNB2 | 0.202864306 |
| BCLAF1 | ETV1 | 0.202284447 |
| BCLAF1 | HIC1 | 0.201242801 |
| BCLAF1 | TLE1 | 0.200902006 |
| BCLAF1 | DDI2 | 0.199822536 |
| BCLAF1 | UBE3A | 0.199689794 |
| BCLAF1 | AP1S1 | 0.198851799 |
| BCLAF1 | MAX | 0.197252977 |
| BCLAF1 | RPRD1A | 0.196631839 |
| BCLAF1 | CD2AP | 0.196573169 |
| BCLAF1 | PDGFA | 0.194405773 |
| BCLAF1 | ANKRD40 | 0.191119777 |
| BCLAF1 | FBXW7 | 0.186279741 |
| BCLAF1 | POU3F1 | 0.182881085 |
| BCLAF1 | FRMD8 | 0.182517969 |
| BCLAF1 | SLC23A2 | 0.179316177 |
| BCLAF1 | GPD2 | 0.178596109 |
| BCLAF1 | DUSP15 | 0.173962764 |
| BCLAF1 | UHRF2 | 0.172620234 |
| BCLAF1 | RECQL4 | 0.171571234 |
| BCLAF1 | RHBDF1 | 0.170172472 |
| BCLAF1 | DEDD | 0.169283182 |
| BCLAF1 | PACS2 | 0.166409588 |
| BCLAF1 | CAMKK1 | 0.164898592 |
| BCLAF1 | CDC42BPA | 0.16168557 |
| BCLAF1 | WASL | 0.159998769 |
| BCLAF1 | CDC73 | 0.156090028 |
| BCLAF1 | BCL2L11 | 0.155141739 |
| BCLAF1 | HEG1 | 0.15428584 |
| BCLAF1 | DIP2A | 0.154126441 |
| BCLAF1 | PLXNC1 | 0.152172002 |
| BCLAF1 | ACCS | 0.149612739 |
| BCLAF1 | SCRIB | 0.135633925 |
| BCLAF1 | ZSCAN18 | 0.131207149 |
| BCLAF1 | RALGDS | 0.126885383 |
| BCLAF1 | PDE10A | 0.120847354 |
| BCLAF1 | STX17 | 0.120366495 |
| BCLAF1 | SUV39H2 | 0.115408418 |
| BCLAF1 | CISH | 0.113576045 |
| BCLAF1 | ZFYVE28 | 0.112439625 |
| BCLAF1 | PRPF40B | 0.111771532 |
| BCLAF1 | SLCO3A1 | 0.11091468 |
| BCLAF1 | ZBTB2 | 0.109356724 |
| BCLAF1 | ACSF2 | 0.105534508 |
| BCLAF1 | TPRA1 | 0.105072396 |
| BCLAF1 | FGFRL1 | 0.10484831 |
| BCLAF1 | ZNF524 | 0.102393743 |
| BCLAF1 | FXYD1 | 0.101655733 |
| BCLAF1 | PI4K2B | 0.101479416 |
| BCLAF1 | AMIGO2 | 0.100730896 |
| BCLAF1 | DNAJB5 | 0.100590841 |
| BCLAF1 | NDRG4 | 0.086242614 |
| BCLAF1 | TCIRG1 | 0.085560605 |
| BCLAF1 | TRIM39 | 0.083901946 |
| BCLAF1 | ZSWIM4 | 0.083781309 |
| BCLAF1 | CORO2B | 0.072139014 |
| BCLAF1 | CELSR2 | 0.064586345 |
| BCLAF1 | SCUBE3 | 0.060889011 |
| BCLAF1 | PTPN4 | 0.056731322 |
| BCLAF1 | PANK4 | 0.04897707 |
| BCLAF1 | USP9X | 0.047697444 |
| BCLAF1 | ITGA9 | 0.045541627 |
| BCLAF1 | HHAT | 0.039808592 |
| BCLAF1 | MSTO1 | 0.031151254 |
| BCLAF1 | PNPLA8 | 0.027372735 |
| BCLAF1 | CHST6 | 0.023224629 |
| BCLAF1 | RELA | 0.021722697 |
| BCLAF1 | RAB3GAP1 | 0.021479356 |
| BCLAF1 | MTA2 | 0.021100453 |
| BCLAF1 | CNOT8 | 0.01150263 |
| BCLAF1 | LCOR | 0.006774249 |
| BCLAF1 | GOLPH3 | 0.006717575 |
| BCLAF1 | PLEKHA3 | 0.006372944 |
| BCLAF1 | L3MBTL2 | 0.005568729 |
| BCLAF1 | MTUS1 | 0.005417742 |
| BCLAF1 | TRAP1 | 0.005326012 |
| BCLAF1 | PGP | 0.004917936 |
| BCLAF1 | KITLG | 0.002971031 |
| BCLAF1 | TIGD5 | 0.002837664 |
| BCLAF1 | MGA | 0.002436705 |
| BCLAF1 | MEX3B | 0.002178807 |
| BCLAF1 | EPS8L1 | 0.002000308 |
| BCLAF1 | DDAH1 | 0.001940635 |
| BCLAF1 | DCUN1D4 | 0.001018814 |
| BCLAF1 | ARIH1 | 0.000563156 |
| BCLAF1 | CCDC12 | 0.0003901 |
| BCLAF1 | VPS13A | 0.000261096 |
| BCLAF1 | HEY2 | 4.90E-05 |
| BCLAF1 | TAF4B | 5.97E-08 |
| BCLAF1 | ATP2C2 | 3.75E-08 |
| BCLAF1 | KLHL13 | 3.18E-09 |
| BCLAF1 | SLC47A1 | 8.69E-19 |
| BCLAF1 | POLA1 | 3.88E-19 |
| BCLAF1 | OTUD4 | 5.16E-20 |
| BCLAF1 | CST6 | 2.93E-20 |
| BHLHE40 | CDKN1A | 7.06327718 |
| BHLHE40 | GNAI1 | 6.326653997 |
| BHLHE40 | IRF1 | 5.022594628 |
| BHLHE40 | EGR1 | 4.769817314 |
| BHLHE40 | CTGF | 4.556357924 |
| BHLHE40 | KLF10 | 4.488326219 |
| BHLHE40 | GADD45A | 4.482476678 |
| BHLHE40 | JUNB | 4.323248218 |
| BHLHE40 | SUGT1 | 3.833932573 |
| BHLHE40 | KLF4 | 3.829401201 |
| BHLHE40 | CEBPB | 3.727785065 |
| BHLHE40 | BCL2 | 3.376212876 |
| BHLHE40 | ZFP36 | 3.050497561 |
| BHLHE40 | CAPZB | 3.045119895 |
| BHLHE40 | ZFAND5 | 3.015918849 |
| BHLHE40 | NR4A1 | 2.855259122 |
| BHLHE40 | ZFP36L1 | 2.825386655 |
| BHLHE40 | FOSB | 2.811151128 |
| BHLHE40 | L3MBTL3 | 2.750935768 |
| BHLHE40 | NFKBIA | 2.705518824 |
| BHLHE40 | REV3L | 2.54035102 |
| BHLHE40 | PSAP | 2.527450566 |
| BHLHE40 | TSC22D1 | 2.512294294 |
| BHLHE40 | MYC | 2.380416178 |
| BHLHE40 | PKP4 | 2.310631197 |
| BHLHE40 | SOD2 | 2.193479094 |
| BHLHE40 | HES1 | 2.043335844 |
| BHLHE40 | MCL1 | 2.01521402 |
| BHLHE40 | PDGFRB | 2.002637074 |
| BHLHE40 | MGRN1 | 1.906790697 |
| BHLHE40 | SQSTM1 | 1.879235303 |
| BHLHE40 | FOS | 1.868836886 |
| BHLHE40 | CDC42BPG | 1.630748542 |
| BHLHE40 | PRKAB2 | 1.584002628 |
| BHLHE40 | JUND | 1.429203092 |
| BHLHE40 | ZBTB5 | 1.38162349 |
| BHLHE40 | ZFR | 1.338753613 |
| BHLHE40 | NUP93 | 1.306248635 |
| BHLHE40 | FBXO30 | 1.29433365 |
| BHLHE40 | UBC | 1.272440798 |
| BHLHE40 | JUN | 1.263239592 |
| BHLHE40 | PAK1 | 1.248097353 |
| BHLHE40 | AIF1L | 1.238535684 |
| BHLHE40 | EGR2 | 1.233874002 |
| BHLHE40 | BICD2 | 1.20090002 |
| BHLHE40 | CD248 | 1.187384307 |
| BHLHE40 | PFN1 | 1.174189115 |
| BHLHE40 | ENTPD2 | 1.169610297 |
| BHLHE40 | HTRA1 | 1.159398681 |
| BHLHE40 | MPP3 | 1.152581411 |
| BHLHE40 | GADD45B | 1.138542648 |
| BHLHE40 | CDH24 | 1.122435434 |
| BHLHE40 | COL5A1 | 1.114986953 |
| BHLHE40 | LIMS2 | 1.114780284 |
| BHLHE40 | SERPING1 | 1.111575051 |
| BHLHE40 | OAZ1 | 1.105135134 |
| BHLHE40 | ZNF579 | 1.095539521 |
| BHLHE40 | BAG3 | 1.077006941 |
| BHLHE40 | CACNA2D3 | 1.070087103 |
| BHLHE40 | TUBB | 1.04730501 |
| BHLHE40 | SPRY2 | 1.038074319 |
| BHLHE40 | VARS2 | 1.034666858 |
| BHLHE40 | AVL9 | 1.024202766 |
| BHLHE40 | SET | 1.019592914 |
| BHLHE40 | KLF6 | 1.00747423 |
| BHLHE40 | MAML1 | 1.000145521 |
| BHLHE40 | BHLHE40 | 1 |
| BHLHE40 | FZD5 | 0.998935339 |
| BHLHE40 | PEPD | 0.985612911 |
| BHLHE40 | SOX18 | 0.935231376 |
| BHLHE40 | HSP90AB1 | 0.916197898 |
| BHLHE40 | AEBP1 | 0.909023804 |
| BHLHE40 | TRHDE | 0.908831782 |
| BHLHE40 | TCF7 | 0.908048392 |
| BHLHE40 | PRSS12 | 0.907939255 |
| BHLHE40 | HELLS | 0.903774081 |
| BHLHE40 | PTPN21 | 0.90089051 |
| BHLHE40 | UBE2S | 0.89394255 |
| BHLHE40 | RXRG | 0.884579755 |
| BHLHE40 | NPC2 | 0.879840617 |
| BHLHE40 | PMP22 | 0.856563154 |
| BHLHE40 | DNAJB1 | 0.854724869 |
| BHLHE40 | FOXO3 | 0.853311589 |
| BHLHE40 | TCF4 | 0.852986364 |
| BHLHE40 | C6orf48 | 0.841636242 |
| BHLHE40 | FBXW7 | 0.841140407 |
| BHLHE40 | CASK | 0.828651964 |
| BHLHE40 | BCL3 | 0.819610225 |
| BHLHE40 | CBX1 | 0.812890291 |
| BHLHE40 | MMP16 | 0.811915352 |
| BHLHE40 | SOX11 | 0.811758577 |
| BHLHE40 | PHLPP1 | 0.805432546 |
| BHLHE40 | HDLBP | 0.792092135 |
| BHLHE40 | CALM2 | 0.772690992 |
| BHLHE40 | PKN1 | 0.764871255 |
| BHLHE40 | CXCR4 | 0.758087996 |
| BHLHE40 | ARPC2 | 0.754938699 |
| BHLHE40 | ID4 | 0.752280272 |
| BHLHE40 | KLF2 | 0.750639419 |
| BHLHE40 | BTG2 | 0.735368579 |
| BHLHE40 | ZNF48 | 0.728789223 |
| BHLHE40 | IRS2 | 0.707629502 |
| BHLHE40 | ENO1 | 0.706606899 |
| BHLHE40 | MAN1A1 | 0.694278016 |
| BHLHE40 | DUSP5 | 0.690942378 |
| BHLHE40 | NSF | 0.684508717 |
| BHLHE40 | TBC1D9 | 0.681762571 |
| BHLHE40 | FAM171B | 0.66519994 |
| BHLHE40 | CANT1 | 0.661914785 |
| BHLHE40 | SOCS1 | 0.651725219 |
| BHLHE40 | PLK1 | 0.650176197 |
| BHLHE40 | ATF3 | 0.649450021 |
| BHLHE40 | HHIP | 0.642893636 |
| BHLHE40 | CDCA8 | 0.641244663 |
| BHLHE40 | LTBP1 | 0.636467933 |
| BHLHE40 | PON3 | 0.636229539 |
| BHLHE40 | H1FX | 0.632961903 |
| BHLHE40 | MAP2 | 0.632231171 |
| BHLHE40 | MAPK7 | 0.63052158 |
| BHLHE40 | LTBP4 | 0.616752471 |
| BHLHE40 | BAMBI | 0.612876784 |
| BHLHE40 | TNFAIP3 | 0.608063463 |
| BHLHE40 | SFN | 0.604945252 |
| BHLHE40 | PPM1L | 0.604573676 |
| BHLHE40 | ARHGAP26 | 0.601735977 |
| BHLHE40 | KBTBD11 | 0.594836394 |
| BHLHE40 | SH3GL1 | 0.594418268 |
| BHLHE40 | AFTPH | 0.593061549 |
| BHLHE40 | GDI2 | 0.587069634 |
| BHLHE40 | AES | 0.586940831 |
| BHLHE40 | NAMPT | 0.576848278 |
| BHLHE40 | NTN4 | 0.567927605 |
| BHLHE40 | UQCRQ | 0.563048382 |
| BHLHE40 | MDH1 | 0.559092247 |
| BHLHE40 | FAM13B | 0.557840749 |
| BHLHE40 | TMEM121 | 0.555727643 |
| BHLHE40 | DAZAP2 | 0.555241876 |
| BHLHE40 | TOX2 | 0.554265062 |
| BHLHE40 | GRB10 | 0.553695291 |
| BHLHE40 | IL6ST | 0.549522047 |
| BHLHE40 | ARC | 0.546452656 |
| BHLHE40 | MAP7 | 0.544319282 |
| BHLHE40 | OLFML2A | 0.539672229 |
| BHLHE40 | CITED2 | 0.533818429 |
| BHLHE40 | E2F6 | 0.533349978 |
| BHLHE40 | TAGLN2 | 0.532732706 |
| BHLHE40 | SOX5 | 0.532256418 |
| BHLHE40 | TMED10 | 0.531819673 |
| BHLHE40 | PSMD1 | 0.526139983 |
| BHLHE40 | HNRNPK | 0.525893002 |
| BHLHE40 | ACTR3B | 0.525715009 |
| BHLHE40 | EFEMP1 | 0.523884124 |
| BHLHE40 | DUSP4 | 0.522326082 |
| BHLHE40 | NMNAT3 | 0.521852351 |
| BHLHE40 | WNT11 | 0.517235476 |
| BHLHE40 | GATA6 | 0.51662845 |
| BHLHE40 | BCAP29 | 0.515758804 |
| BHLHE40 | MTSS1L | 0.511942862 |
| BHLHE40 | USP34 | 0.507239033 |
| BHLHE40 | HES4 | 0.505877055 |
| BHLHE40 | CELSR1 | 0.505136729 |
| BHLHE40 | NPTX2 | 0.502081661 |
| BHLHE40 | S100A11 | 0.499444505 |
| BHLHE40 | CCDC40 | 0.498645695 |
| BHLHE40 | NDFIP1 | 0.494964737 |
| BHLHE40 | NOMO1 | 0.493875116 |
| BHLHE40 | CDKN3 | 0.492940389 |
| BHLHE40 | GSN | 0.492671891 |
| BHLHE40 | MEX3A | 0.488306442 |
| BHLHE40 | BEND3 | 0.482984161 |
| BHLHE40 | B3GAT3 | 0.480964385 |
| BHLHE40 | WFS1 | 0.476996175 |
| BHLHE40 | TMBIM6 | 0.476342968 |
| BHLHE40 | BCL7A | 0.473087751 |
| BHLHE40 | CCDC149 | 0.471052155 |
| BHLHE40 | BUD31 | 0.470868664 |
| BHLHE40 | RARA | 0.470599998 |
| BHLHE40 | SRRM1 | 0.460028894 |
| BHLHE40 | EPB41 | 0.452868988 |
| BHLHE40 | MGST3 | 0.4480799 |
| BHLHE40 | CCDC36 | 0.446059193 |
| BHLHE40 | RND2 | 0.442712826 |
| BHLHE40 | STRBP | 0.438063717 |
| BHLHE40 | CCDC84 | 0.434143769 |
| BHLHE40 | SPAG5 | 0.428736964 |
| BHLHE40 | FOXP4 | 0.427293941 |
| BHLHE40 | TADA1 | 0.425954229 |
| BHLHE40 | GKAP1 | 0.423125083 |
| BHLHE40 | RAB11FIP1 | 0.422483548 |
| BHLHE40 | FAM117B | 0.421378399 |
| BHLHE40 | CHST1 | 0.420771437 |
| BHLHE40 | XYLT1 | 0.41959032 |
| BHLHE40 | BMP7 | 0.411747439 |
| BHLHE40 | HSPB8 | 0.41090187 |
| BHLHE40 | ZMIZ1 | 0.404962356 |
| BHLHE40 | CDC42BPA | 0.398567028 |
| BHLHE40 | METTL1 | 0.396719848 |
| BHLHE40 | PKD2 | 0.396169407 |
| BHLHE40 | EDN2 | 0.394463217 |
| BHLHE40 | DENND1A | 0.39165692 |
| BHLHE40 | ZADH2 | 0.388154072 |
| BHLHE40 | ZNF419 | 0.385362949 |
| BHLHE40 | TMUB1 | 0.382919407 |
| BHLHE40 | CADPS | 0.38068613 |
| BHLHE40 | TSNAXIP1 | 0.377846225 |
| BHLHE40 | TAF10 | 0.376924584 |
| BHLHE40 | GRIA4 | 0.376699779 |
| BHLHE40 | GAPDH | 0.370602868 |
| BHLHE40 | NR1D2 | 0.368271483 |
| BHLHE40 | HEY1 | 0.363882718 |
| BHLHE40 | EYA4 | 0.359070717 |
| BHLHE40 | SLC35A5 | 0.35665374 |
| BHLHE40 | ZFAND3 | 0.3536523 |
| BHLHE40 | KRBA1 | 0.351509219 |
| BHLHE40 | SPG21 | 0.350109901 |
| BHLHE40 | PTGFRN | 0.348110815 |
| BHLHE40 | TMEM184B | 0.343825027 |
| BHLHE40 | SGPP1 | 0.342431985 |
| BHLHE40 | TUG1 | 0.336862153 |
| BHLHE40 | DLG5 | 0.336518018 |
| BHLHE40 | ACAP3 | 0.334016526 |
| BHLHE40 | VASN | 0.333965354 |
| BHLHE40 | IFT81 | 0.332535922 |
| BHLHE40 | ZNF711 | 0.330777139 |
| BHLHE40 | GNB4 | 0.330512224 |
| BHLHE40 | SEC24B | 0.328706973 |
| BHLHE40 | ANKRD10 | 0.325358051 |
| BHLHE40 | FAM124A | 0.325318556 |
| BHLHE40 | MEF2D | 0.324589619 |
| BHLHE40 | SLC34A2 | 0.323810594 |
| BHLHE40 | SEMA6D | 0.323265668 |
| BHLHE40 | TMEM135 | 0.321801798 |
| BHLHE40 | RFX1 | 0.317527198 |
| BHLHE40 | RANBP10 | 0.315092085 |
| BHLHE40 | GLI1 | 0.313160258 |
| BHLHE40 | NEFH | 0.312433454 |
| BHLHE40 | DNAJB5 | 0.309938984 |
| BHLHE40 | HES6 | 0.309391873 |
| BHLHE40 | CHEK1 | 0.30802328 |
| BHLHE40 | TP53INP1 | 0.304617423 |
| BHLHE40 | BAHD1 | 0.304331046 |
| BHLHE40 | DSG2 | 0.299215568 |
| BHLHE40 | KIAA1328 | 0.298387624 |
| BHLHE40 | KATNAL1 | 0.298332154 |
| BHLHE40 | UBE2V2 | 0.295260956 |
| BHLHE40 | C15orf39 | 0.291002389 |
| BHLHE40 | ETS2 | 0.289658126 |
| BHLHE40 | USP28 | 0.289097547 |
| BHLHE40 | KCTD2 | 0.287904177 |
| BHLHE40 | MFSD3 | 0.287379077 |
| BHLHE40 | OTUD4 | 0.286686481 |
| BHLHE40 | TC2N | 0.286325878 |
| BHLHE40 | ZDHHC18 | 0.277622804 |
| BHLHE40 | SPSB2 | 0.276773941 |
| BHLHE40 | GPS1 | 0.274801306 |
| BHLHE40 | CNIH3 | 0.274206892 |
| BHLHE40 | UBE2O | 0.273547261 |
| BHLHE40 | ATP9B | 0.27282168 |
| BHLHE40 | PPP1R3D | 0.270330662 |
| BHLHE40 | OTUD1 | 0.267357806 |
| BHLHE40 | TADA2B | 0.265318132 |
| BHLHE40 | GFOD1 | 0.263827183 |
| BHLHE40 | CACNB1 | 0.263508521 |
| BHLHE40 | INHBB | 0.257993677 |
| BHLHE40 | ASTN2 | 0.257047646 |
| BHLHE40 | LRP4 | 0.25616799 |
| BHLHE40 | CDX1 | 0.251659658 |
| BHLHE40 | IER5L | 0.251205526 |
| BHLHE40 | ZNF746 | 0.248324937 |
| BHLHE40 | TBX18 | 0.245613788 |
| BHLHE40 | FAM98A | 0.245211072 |
| BHLHE40 | MYO5A | 0.244558118 |
| BHLHE40 | USP45 | 0.243980515 |
| BHLHE40 | IRF2BP2 | 0.243406274 |
| BHLHE40 | MAST2 | 0.2433252 |
| BHLHE40 | SF3A2 | 0.242363375 |
| BHLHE40 | TMEM201 | 0.241869015 |
| BHLHE40 | C21orf91 | 0.23978774 |
| BHLHE40 | SNTB2 | 0.239108112 |
| BHLHE40 | GNL3L | 0.236442721 |
| BHLHE40 | NTN1 | 0.232860181 |
| BHLHE40 | FZD10 | 0.231575933 |
| BHLHE40 | PRKCE | 0.229981832 |
| BHLHE40 | FZD8 | 0.227393729 |
| BHLHE40 | HAUS1 | 0.226626238 |
| BHLHE40 | FNTA | 0.22578357 |
| BHLHE40 | EML3 | 0.224777609 |
| BHLHE40 | ARL10 | 0.223191709 |
| BHLHE40 | POLR3E | 0.222365064 |
| BHLHE40 | NAB1 | 0.22079283 |
| BHLHE40 | ZNF358 | 0.218926611 |
| BHLHE40 | SMCHD1 | 0.218687038 |
| BHLHE40 | APBB1 | 0.217538411 |
| BHLHE40 | RNF111 | 0.214309791 |
| BHLHE40 | AKAP10 | 0.214306244 |
| BHLHE40 | SETD1B | 0.212754904 |
| BHLHE40 | ODC1 | 0.212418062 |
| BHLHE40 | ZNF664 | 0.210943233 |
| BHLHE40 | CRTC2 | 0.208756347 |
| BHLHE40 | LMF1 | 0.208233011 |
| BHLHE40 | MCM5 | 0.206681936 |
| BHLHE40 | BECN1 | 0.206646846 |
| BHLHE40 | ATE1 | 0.205698871 |
| BHLHE40 | DOCK11 | 0.204087273 |
| BHLHE40 | MMAA | 0.203943209 |
| BHLHE40 | PUF60 | 0.203666274 |
| BHLHE40 | PAG1 | 0.201369817 |
| BHLHE40 | MYH9 | 0.200836165 |
| BHLHE40 | AKAP8 | 0.200325105 |
| BHLHE40 | CLASP1 | 0.199959623 |
| BHLHE40 | TBC1D12 | 0.199728144 |
| BHLHE40 | PRKACA | 0.199226171 |
| BHLHE40 | NCAM2 | 0.197507194 |
| BHLHE40 | ANKRD13B | 0.196303411 |
| BHLHE40 | TXNDC16 | 0.195688244 |
| BHLHE40 | JDP2 | 0.194154482 |
| BHLHE40 | CREM | 0.193718184 |
| BHLHE40 | ZNF362 | 0.193099094 |
| BHLHE40 | UNC5B | 0.192174139 |
| BHLHE40 | REPS2 | 0.192027169 |
| BHLHE40 | ZNF821 | 0.191416667 |
| BHLHE40 | PELI2 | 0.190744527 |
| BHLHE40 | NFYB | 0.187479839 |
| BHLHE40 | BLMH | 0.185793988 |
| BHLHE40 | RABGAP1L | 0.185692643 |
| BHLHE40 | INTS6 | 0.185577053 |
| BHLHE40 | DCTN6 | 0.184529239 |
| BHLHE40 | DGKZ | 0.184495179 |
| BHLHE40 | ZNF764 | 0.182867402 |
| BHLHE40 | SLC25A37 | 0.182257406 |
| BHLHE40 | ANKRD28 | 0.181396847 |
| BHLHE40 | EIF2S3 | 0.18107499 |
| BHLHE40 | ZNF584 | 0.180147743 |
| BHLHE40 | SPRYD4 | 0.178637391 |
| BHLHE40 | TFAP2C | 0.178519536 |
| BHLHE40 | AGAP3 | 0.17830354 |
| BHLHE40 | EIF4ENIF1 | 0.177262537 |
| BHLHE40 | ZBED4 | 0.176538919 |
| BHLHE40 | SLITRK5 | 0.174416384 |
| BHLHE40 | HIST1H4J | 0.174302985 |
| BHLHE40 | CX3CL1 | 0.173457323 |
| BHLHE40 | CSNK1D | 0.172105158 |
| BHLHE40 | WIF1 | 0.171697669 |
| BHLHE40 | C16orf74 | 0.170878231 |
| BHLHE40 | SPEN | 0.168434247 |
| BHLHE40 | UBE2G2 | 0.167989197 |
| BHLHE40 | STAU2 | 0.167862595 |
| BHLHE40 | SLC43A3 | 0.167835174 |
| BHLHE40 | GNA12 | 0.167658559 |
| BHLHE40 | KPNA4 | 0.165510935 |
| BHLHE40 | UPF2 | 0.164551063 |
| BHLHE40 | RECQL4 | 0.162722531 |
| BHLHE40 | DNMT3A | 0.160568217 |
| BHLHE40 | BMP2K | 0.160139081 |
| BHLHE40 | BCL11B | 0.158894948 |
| BHLHE40 | C14orf37 | 0.158591035 |
| BHLHE40 | TRPC1 | 0.158449686 |
| BHLHE40 | GTDC1 | 0.157889718 |
| BHLHE40 | ARHGAP33 | 0.157727253 |
| BHLHE40 | IFT140 | 0.15723457 |
| BHLHE40 | LAMA3 | 0.15702026 |
| BHLHE40 | NHS | 0.155203786 |
| BHLHE40 | DDHD2 | 0.155139569 |
| BHLHE40 | SLC7A2 | 0.153163032 |
| BHLHE40 | DNM3 | 0.151611581 |
| BHLHE40 | MAPK6 | 0.149422379 |
| BHLHE40 | RALGAPA2 | 0.148490294 |
| BHLHE40 | SP3 | 0.147693378 |
| BHLHE40 | PRPSAP1 | 0.146082785 |
| BHLHE40 | DENND3 | 0.145941511 |
| BHLHE40 | CADM2 | 0.1458319 |
| BHLHE40 | ADAMTSL2 | 0.145601407 |
| BHLHE40 | CCNY | 0.14468994 |
| BHLHE40 | RELB | 0.143643847 |
| BHLHE40 | BAIAP2 | 0.143282875 |
| BHLHE40 | NEDD1 | 0.142742049 |
| BHLHE40 | CDC34 | 0.142643736 |
| BHLHE40 | HNRNPUL2 | 0.142482148 |
| BHLHE40 | TGM2 | 0.141496004 |
| BHLHE40 | MARK1 | 0.140662043 |
| BHLHE40 | ALS2CL | 0.139864659 |
| BHLHE40 | SIK3 | 0.139542431 |
| BHLHE40 | KDM3A | 0.137276495 |
| BHLHE40 | PTPN1 | 0.136863582 |
| BHLHE40 | NPR1 | 0.136524441 |
| BHLHE40 | PHF2 | 0.134976395 |
| BHLHE40 | TDRD3 | 0.133256925 |
| BHLHE40 | STK3 | 0.132609143 |
| BHLHE40 | ZNF524 | 0.131528942 |
| BHLHE40 | TCOF1 | 0.12922158 |
| BHLHE40 | PARN | 0.12677799 |
| BHLHE40 | SGMS2 | 0.12308876 |
| BHLHE40 | NFE2L3 | 0.121200831 |
| BHLHE40 | HAGHL | 0.120590452 |
| BHLHE40 | C8orf33 | 0.120502946 |
| BHLHE40 | ADAMTS14 | 0.120251846 |
| BHLHE40 | FGF10 | 0.119269297 |
| BHLHE40 | EDN1 | 0.114482563 |
| BHLHE40 | DCBLD2 | 0.113373232 |
| BHLHE40 | TMC6 | 0.107544826 |
| BHLHE40 | MIER3 | 0.101329141 |
| BHLHE40 | RTTN | 0.099426884 |
| BHLHE40 | USP25 | 0.096693723 |
| BHLHE40 | E2F8 | 0.088509938 |
| BHLHE40 | RASGEF1A | 0.084325486 |
| BHLHE40 | CTDSPL | 0.081504474 |
| BHLHE40 | ATP2A2 | 0.077030694 |
| BHLHE40 | RELN | 0.07238289 |
| BHLHE40 | ATOH8 | 0.068799907 |
| BHLHE40 | TMEM200B | 0.063579329 |
| BHLHE40 | ETV1 | 0.054680481 |
| BHLHE40 | CLDN11 | 0.040830341 |
| BHLHE40 | TRIM24 | 0.037326784 |
| BHLHE40 | LPGAT1 | 0.036152497 |
| BHLHE40 | LRFN3 | 0.028122829 |
| BHLHE40 | FMNL2 | 0.019219616 |
| BHLHE40 | EFHD1 | 0.013820044 |
| BHLHE40 | ZBTB10 | 0.011138308 |
| BHLHE40 | SYVN1 | 0.007645515 |
| BHLHE40 | ZC4H2 | 0.007251311 |
| BHLHE40 | SREK1 | 0.006003702 |
| BHLHE40 | ARL8B | 0.005940186 |
| BHLHE40 | DUSP7 | 0.005195444 |
| BHLHE40 | MON2 | 0.004437976 |
| BHLHE40 | ATXN1 | 0.003544581 |
| BHLHE40 | WWP2 | 0.002601404 |
| BHLHE40 | CBX7 | 0.001909234 |
| BHLHE40 | EIF2B2 | 0.000677021 |
| BHLHE40 | TYMP | 0.000175902 |
| BHLHE40 | DHCR24 | 0.000113963 |
| BHLHE40 | RCAN3 | 1.81E-05 |
| BHLHE40 | ASPHD2 | 9.40E-06 |
| BHLHE40 | RAB15 | 3.04E-06 |
| BHLHE40 | DCT | 2.34E-06 |
| BHLHE40 | MUC1 | 2.28E-07 |
| BHLHE40 | IRF8 | 1.18E-07 |
| BHLHE40 | IL17RD | 6.29E-10 |
| BHLHE40 | PAQR3 | 1.14E-10 |
| BHLHE40 | FRAS1 | 7.41E-11 |
| BHLHE40 | RHCG | 9.79E-12 |
| BHLHE40 | MYH11 | 7.00E-17 |
| BHLHE40 | STX1A | 4.38E-19 |
| BHLHE40 | SMARCAL1 | 3.71E-19 |
| BHLHE41 | EDNRA | 10.26160704 |
| BHLHE41 | SF3B3 | 7.922563442 |
| BHLHE41 | PALLD | 4.882388848 |
| BHLHE41 | WASF2 | 4.429830741 |
| BHLHE41 | HAPLN1 | 4.260980999 |
| BHLHE41 | ILF3 | 3.822741944 |
| BHLHE41 | MEF2C | 3.297842114 |
| BHLHE41 | ZHX1 | 2.141044185 |
| BHLHE41 | URM1 | 1.919593847 |
| BHLHE41 | HNRNPD | 1.775706809 |
| BHLHE41 | TNFAIP3 | 1.760805713 |
| BHLHE41 | KIF26B | 1.500693923 |
| BHLHE41 | LIMS1 | 1.294894859 |
| BHLHE41 | H1F0 | 1.258930749 |
| BHLHE41 | UBAP2L | 1.166696142 |
| BHLHE41 | TFAP2A | 1.154540021 |
| BHLHE41 | ARHGDIA | 1.104755264 |
| BHLHE41 | JAK1 | 1.082632332 |
| BHLHE41 | TMOD3 | 1.061960042 |
| BHLHE41 | NR1D1 | 1.061682039 |
| BHLHE41 | GTF3A | 1.020020522 |
| BHLHE41 | MRPL23 | 1.008289988 |
| BHLHE41 | BHLHE41 | 1 |
| BHLHE41 | CREB3L1 | 0.994546529 |
| BHLHE41 | SH3GL1 | 0.977037458 |
| BHLHE41 | SORBS2 | 0.976641337 |
| BHLHE41 | CTSB | 0.964938641 |
| BHLHE41 | PTMS | 0.944817964 |
| BHLHE41 | CDK4 | 0.91799861 |
| BHLHE41 | LMO4 | 0.831140838 |
| BHLHE41 | SLC26A2 | 0.801709721 |
| BHLHE41 | SDAD1 | 0.743514325 |
| BHLHE41 | CYB5R3 | 0.741068088 |
| BHLHE41 | UBAP2 | 0.721429627 |
| BHLHE41 | CAT | 0.702611944 |
| BHLHE41 | RNF4 | 0.667031835 |
| BHLHE41 | SCLY | 0.624442532 |
| BHLHE41 | C1QTNF4 | 0.56636814 |
| BHLHE41 | TMSB4X | 0.528588209 |
| BHLHE41 | EIF4G3 | 0.523487864 |
| BHLHE41 | C14orf93 | 0.501595036 |
| BHLHE41 | NANP | 0.489369804 |
| BHLHE41 | CRABP2 | 0.468277473 |
| BHLHE41 | STOX2 | 0.464084302 |
| BHLHE41 | BCOR | 0.463210337 |
| BHLHE41 | PTPRS | 0.449750461 |
| BHLHE41 | MTCH2 | 0.449051128 |
| BHLHE41 | ZNF273 | 0.398896713 |
| BHLHE41 | MICAL2 | 0.372258245 |
| BHLHE41 | DHX30 | 0.364433883 |
| BHLHE41 | RNF146 | 0.355433727 |
| BHLHE41 | CSDE1 | 0.354128395 |
| BHLHE41 | TRAPPC2L | 0.353932267 |
| BHLHE41 | CRLF3 | 0.352012861 |
| BHLHE41 | ZNF687 | 0.330099325 |
| BHLHE41 | NINJ2 | 0.320871529 |
| BHLHE41 | CDC42EP1 | 0.305443726 |
| BHLHE41 | SFMBT1 | 0.292638211 |
| BHLHE41 | PLA2G15 | 0.288015137 |
| BHLHE41 | VRK2 | 0.285207331 |
| BHLHE41 | PPP3CB | 0.276363747 |
| BHLHE41 | C19orf48 | 0.272540589 |
| BHLHE41 | BDP1 | 0.271301932 |
| BHLHE41 | GIGYF1 | 0.270659653 |
| BHLHE41 | ADSL | 0.270526218 |
| BHLHE41 | C1QTNF1 | 0.262598908 |
| BHLHE41 | IREB2 | 0.244048899 |
| BHLHE41 | ERBB3 | 0.231635262 |
| BHLHE41 | SEL1L3 | 0.224953478 |
| BHLHE41 | SP7 | 0.220674305 |
| BHLHE41 | METAP1D | 0.21860022 |
| BHLHE41 | AZIN1 | 0.213519631 |
| BHLHE41 | NHS | 0.190345876 |
| BHLHE41 | TNK2 | 0.189737302 |
| BHLHE41 | IL17RD | 0.187798604 |
| BHLHE41 | ALKBH8 | 0.181440942 |
| BHLHE41 | ZFAT | 0.176632976 |
| BHLHE41 | MAP3K14 | 0.169007097 |
| BHLHE41 | ATG3 | 0.163522725 |
| BHLHE41 | UCK2 | 0.159508181 |
| BHLHE41 | ECSIT | 0.151940167 |
| BHLHE41 | APBA1 | 0.148457094 |
| BHLHE41 | CDCP1 | 0.141044029 |
| BHLHE41 | IKZF4 | 0.131356852 |
| BHLHE41 | SYDE2 | 0.125264175 |
| BHLHE41 | ASPHD2 | 0.116541087 |
| BHLHE41 | THAP8 | 0.109022599 |
| BHLHE41 | PRPSAP2 | 0.106234876 |
| BHLHE41 | TNNT3 | 0.103467946 |
| BHLHE41 | SYT17 | 0.093597672 |
| BHLHE41 | PPCDC | 0.089857424 |
| BHLHE41 | RWDD3 | 0.067897941 |
| BHLHE41 | CPEB1 | 0.0355323 |
| BHLHE41 | SUMF1 | 0.027123987 |
| BHLHE41 | THNSL1 | 0.025320797 |
| BHLHE41 | FHDC1 | 0.017653345 |
| BHLHE41 | INPP5F | 0.001052509 |
| BHLHE41 | NSD1 | 0.000786495 |
| BHLHE41 | GPSM1 | 5.81E-08 |
| BHLHE41 | KLHL26 | 6.53E-09 |
| BRCA1 | CEP55 | 5.455002594 |
| BRCA1 | HMMR | 4.795408305 |
| BRCA1 | TOP2A | 4.522086164 |
| BRCA1 | SLC16A9 | 4.075947557 |
| BRCA1 | VPS72 | 3.418982763 |
| BRCA1 | PRCC | 3.366394231 |
| BRCA1 | LRP6 | 3.131781354 |
| BRCA1 | CDC45 | 2.682434386 |
| BRCA1 | TTC32 | 1.879043394 |
| BRCA1 | CDKN2C | 1.855383479 |
| BRCA1 | TXNIP | 1.837369592 |
| BRCA1 | MBTPS2 | 1.737064116 |
| BRCA1 | PPP2R3C | 1.633470861 |
| BRCA1 | TRIL | 1.620432667 |
| BRCA1 | ATAD2 | 1.602465128 |
| BRCA1 | C6orf89 | 1.418072734 |
| BRCA1 | DMTF1 | 1.358396354 |
| BRCA1 | SH3KBP1 | 1.278276131 |
| BRCA1 | RFX7 | 1.235685904 |
| BRCA1 | TLE4 | 1.209219449 |
| BRCA1 | NUDCD2 | 1.139322213 |
| BRCA1 | USP25 | 1.137997795 |
| BRCA1 | MKI67 | 1.061127816 |
| BRCA1 | BRCA1 | 1 |
| BRCA1 | RIC8B | 0.984623531 |
| BRCA1 | LIMCH1 | 0.961157111 |
| BRCA1 | FBLIM1 | 0.95442434 |
| BRCA1 | SLU7 | 0.944903012 |
| BRCA1 | RNF139 | 0.899819672 |
| BRCA1 | ERN1 | 0.893273501 |
| BRCA1 | GCA | 0.881822159 |
| BRCA1 | PCNA | 0.877478068 |
| BRCA1 | NCAPG | 0.862361686 |
| BRCA1 | MAD2L1 | 0.858597425 |
| BRCA1 | CCNB1 | 0.832805951 |
| BRCA1 | POU3F4 | 0.77706293 |
| BRCA1 | ELAVL1 | 0.591693796 |
| BRCA1 | ADAMTS5 | 0.585845203 |
| BRCA1 | TIA1 | 0.57615487 |
| BRCA1 | CDKN3 | 0.558182413 |
| BRCA1 | UHRF1 | 0.492987124 |
| BRCA1 | PPP1R2 | 0.47863542 |
| BRCA1 | C3orf58 | 0.476551889 |
| BRCA1 | RBM17 | 0.451831033 |
| BRCA1 | UPF2 | 0.439313833 |
| BRCA1 | ANKRD16 | 0.437714838 |
| BRCA1 | ITSN1 | 0.429033766 |
| BRCA1 | VPS35 | 0.399177251 |
| BRCA1 | UBE2J2 | 0.389907359 |
| BRCA1 | TMEM117 | 0.339188424 |
| BRCA1 | UBL3 | 0.335774628 |
| BRCA1 | KCTD9 | 0.316954263 |
| BRCA1 | FAM118A | 0.311069577 |
| BRCA1 | ATF7IP | 0.295670022 |
| BRCA1 | BARX2 | 0.294861093 |
| BRCA1 | MRPL35 | 0.274764697 |
| BRCA1 | KDM5A | 0.274178716 |
| BRCA1 | RBM28 | 0.267555053 |
| BRCA1 | CDC7 | 0.267184102 |
| BRCA1 | ASXL1 | 0.246935362 |
| BRCA1 | SCARB1 | 0.239309309 |
| BRCA1 | TMX1 | 0.237656574 |
| BRCA1 | THRA | 0.232994373 |
| BRCA1 | TCERG1 | 0.226436841 |
| BRCA1 | TOP1 | 0.217197045 |
| BRCA1 | EBAG9 | 0.216754539 |
| BRCA1 | BRIP1 | 0.210371214 |
| BRCA1 | KLF13 | 0.19225065 |
| BRCA1 | ZFAND6 | 0.159210568 |
| BRCA1 | SNX12 | 0.132666443 |
| BRCA1 | MAP3K7 | 0.128733378 |
| BRCA1 | POLR3A | 0.12664665 |
| BRCA1 | ATP1B1 | 0.122212665 |
| BRCA1 | CDC6 | 0.112282513 |
| BRCA1 | MAP4K3 | 0.106114287 |
| BRCA1 | DNAJB5 | 0.103589721 |
| BRCA1 | CHERP | 0.098411158 |
| BRCA1 | NAPEPLD | 0.090478001 |
| BRCA1 | RFC3 | 0.079256375 |
| BRCA1 | TIGD6 | 0.06797498 |
| BRCA1 | PITPNB | 0.00779045 |
| BRF1 | BRD9 | 3.914361503 |
| BRF1 | BASP1 | 3.828205789 |
| BRF1 | FOXN1 | 3.455478604 |
| BRF1 | BCDIN3D | 2.954227895 |
| BRF1 | MON1A | 2.242131958 |
| BRF1 | CHL1 | 1.839024513 |
| BRF1 | HEXIM1 | 1.493429167 |
| BRF1 | CCBE1 | 1.353396393 |
| BRF1 | ARMC9 | 1.340196198 |
| BRF1 | ATAD2 | 1.287661817 |
| BRF1 | ITIH5 | 1.154248858 |
| BRF1 | MASTL | 1.07636614 |
| BRF1 | VAT1 | 0.989565471 |
| BRF1 | NF2 | 0.932390429 |
| BRF1 | C1QTNF6 | 0.927479967 |
| BRF1 | LRSAM1 | 0.887917997 |
| BRF1 | RRAGD | 0.739549787 |
| BRF1 | SUCLG1 | 0.696798252 |
| BRF1 | ZER1 | 0.652344742 |
| BRF1 | METTL3 | 0.644696706 |
| BRF1 | ERLEC1 | 0.569894868 |
| BRF1 | DUSP4 | 0.529910483 |
| BRF1 | METTL6 | 0.517667883 |
| BRF1 | ANAPC2 | 0.508242436 |
| BRF1 | ZNF33B | 0.497733682 |
| BRF1 | BCCIP | 0.48242797 |
| BRF1 | TRIM25 | 0.477724561 |
| BRF1 | XRCC1 | 0.45394099 |
| BRF1 | SCMH1 | 0.447404329 |
| BRF1 | SLC35A2 | 0.447086313 |
| BRF1 | NUDCD2 | 0.441979803 |
| BRF1 | ZNF470 | 0.432650798 |
| BRF1 | KCTD10 | 0.427636217 |
| BRF1 | PGM3 | 0.420915945 |
| BRF1 | DYNLT1 | 0.416031059 |
| BRF1 | TXLNA | 0.414971283 |
| BRF1 | GPR68 | 0.413234135 |
| BRF1 | FOSL2 | 0.412122699 |
| BRF1 | ILK | 0.407039723 |
| BRF1 | MED19 | 0.351256616 |
| BRF1 | MYCN | 0.344770434 |
| BRF1 | CHAF1A | 0.326800064 |
| BRF1 | SP5 | 0.320741083 |
| BRF1 | HPS1 | 0.303661081 |
| BRF1 | LY96 | 0.300164078 |
| BRF1 | FBXO22 | 0.297826164 |
| BRF1 | GFRA1 | 0.27115122 |
| BRF1 | RPL32P3 | 0.270422704 |
| BRF1 | INPP5A | 0.255989756 |
| BRF1 | SLC25A29 | 0.249543252 |
| BRF1 | ATXN2 | 0.244082695 |
| BRF1 | RAB27B | 0.242434667 |
| BRF1 | SIDT2 | 0.239404379 |
| BRF1 | CASC4 | 0.23431714 |
| BRF1 | LIMS2 | 0.226490949 |
| BRF1 | MTMR10 | 0.224150005 |
| BRF1 | TRIM38 | 0.219746696 |
| BRF1 | IGF2R | 0.218321681 |
| BRF1 | NCSTN | 0.216854882 |
| BRF1 | PABPC5 | 0.209651339 |
| BRF1 | TMEM60 | 0.206138583 |
| BRF1 | FOXQ1 | 0.197652126 |
| BRF1 | AEBP2 | 0.19224353 |
| BRF1 | SURF6 | 0.186556111 |
| BRF1 | SLC26A2 | 0.184661501 |
| BRF1 | WASF1 | 0.166195536 |
| BRF1 | SUPT6H | 0.163474623 |
| BRF1 | ZNF280D | 0.159064082 |
| BRF1 | CCHCR1 | 0.145102231 |
| BRF1 | VMA21 | 0.130156527 |
| BRF1 | SMG5 | 0.126253349 |
| BRF1 | CMBL | 0.125371412 |
| BRF1 | ZDHHC7 | 0.118682077 |
| BRF1 | C11orf95 | 0.11708784 |
| BRF1 | ABT1 | 0.115431357 |
| BRF1 | DRG2 | 0.11177464 |
| BRF1 | PHF12 | 0.106305602 |
| BRF1 | ALG6 | 0.104275362 |
| BRF1 | NIF3L1 | 0.096977301 |
| BRF1 | GRP | 0.090813236 |
| BRF1 | N4BP2 | 0.090373888 |
| BRF1 | CIDECP | 0.087938797 |
| BRF1 | IFT140 | 0.087918356 |
| BRF1 | ACACA | 0.049221568 |
| BRF1 | PSTK | 0.04753742 |
| BRF1 | SDK2 | 0.04710828 |
| BRF1 | FOXP4 | 0.037211431 |
| BRF1 | PRORSD1P | 0.020265597 |
| BRF2 | ACTR3 | 3.391238594 |
| BRF2 | FAM32A | 2.911118839 |
| BRF2 | TCF7L2 | 2.479020613 |
| BRF2 | SDF2 | 2.455049107 |
| BRF2 | SNRPA1 | 2.40502339 |
| BRF2 | HSPA4L | 2.391043595 |
| BRF2 | TRIP10 | 2.196937542 |
| BRF2 | HMGN2 | 2.123805489 |
| BRF2 | ZRSR2 | 2.10863832 |
| BRF2 | NMRAL1 | 1.929124103 |
| BRF2 | EIF4E2 | 1.843319056 |
| BRF2 | ERRFI1 | 1.642980861 |
| BRF2 | EIF4EBP2 | 1.415880938 |
| BRF2 | CUL1 | 1.324074438 |
| BRF2 | LRRC8A | 1.216744917 |
| BRF2 | CROCC | 1.078951239 |
| BRF2 | CDK10 | 1.051225828 |
| BRF2 | FOXP1 | 1.04364918 |
| BRF2 | TMEM60 | 1.023219068 |
| BRF2 | ZCCHC17 | 1.020819143 |
| BRF2 | BRF2 | 1 |
| BRF2 | CNOT6L | 0.992162825 |
| BRF2 | TRIM38 | 0.985115924 |
| BRF2 | TIMM10 | 0.967614987 |
| BRF2 | RPL10 | 0.907693525 |
| BRF2 | THRA | 0.907375582 |
| BRF2 | TRIM8 | 0.894610462 |
| BRF2 | FOSL2 | 0.857616366 |
| BRF2 | RNF14 | 0.843465328 |
| BRF2 | GMPPB | 0.835763875 |
| BRF2 | RWDD1 | 0.835354388 |
| BRF2 | CYHR1 | 0.835237836 |
| BRF2 | TYRO3 | 0.807783113 |
| BRF2 | PTCH2 | 0.786309752 |
| BRF2 | ZNF318 | 0.772319365 |
| BRF2 | ZDHHC20 | 0.772144778 |
| BRF2 | SMUG1 | 0.751861396 |
| BRF2 | MTA1 | 0.737600859 |
| BRF2 | C9orf72 | 0.728634649 |
| BRF2 | ERN1 | 0.677839553 |
| BRF2 | ORAI1 | 0.676271546 |
| BRF2 | NOTCH3 | 0.652914601 |
| BRF2 | EIF4E | 0.652176161 |
| BRF2 | DLG4 | 0.649257652 |
| BRF2 | PHKB | 0.636791975 |
| BRF2 | TRAPPC1 | 0.631658309 |
| BRF2 | SULF1 | 0.631655896 |
| BRF2 | ZNF703 | 0.620272411 |
| BRF2 | FAM168B | 0.619614583 |
| BRF2 | URM1 | 0.594981468 |
| BRF2 | ZNF684 | 0.581739931 |
| BRF2 | SPIN1 | 0.556729738 |
| BRF2 | EPC2 | 0.552806352 |
| BRF2 | MAPRE1 | 0.537081488 |
| BRF2 | FGGY | 0.515447146 |
| BRF2 | PRDM1 | 0.513822639 |
| BRF2 | NOSIP | 0.505879162 |
| BRF2 | ZMPSTE24 | 0.497933557 |
| BRF2 | UBE3B | 0.496024091 |
| BRF2 | RUVBL2 | 0.492702853 |
| BRF2 | SPATS2 | 0.488170014 |
| BRF2 | PAFAH1B1 | 0.483668204 |
| BRF2 | CSNK2B | 0.48180459 |
| BRF2 | MAP4 | 0.467527935 |
| BRF2 | ZNF197 | 0.460043009 |
| BRF2 | MAGED2 | 0.458879915 |
| BRF2 | SMAD3 | 0.437991656 |
| BRF2 | NFKB1 | 0.435828819 |
| BRF2 | NSD1 | 0.431465176 |
| BRF2 | ITPR3 | 0.428805334 |
| BRF2 | NOD1 | 0.423509938 |
| BRF2 | YY1 | 0.422934593 |
| BRF2 | FAM20C | 0.4164071 |
| BRF2 | VAPA | 0.399935777 |
| BRF2 | CDYL | 0.391231884 |
| BRF2 | PDE4A | 0.371494125 |
| BRF2 | PHF21A | 0.36867906 |
| BRF2 | RGS10 | 0.368533983 |
| BRF2 | RIMKLB | 0.367368699 |
| BRF2 | PPP1R14B | 0.366888575 |
| BRF2 | HSPB6 | 0.366405159 |
| BRF2 | DYNLL2 | 0.366230988 |
| BRF2 | MLXIP | 0.360034072 |
| BRF2 | WASF3 | 0.358060514 |
| BRF2 | CTDSP2 | 0.328005839 |
| BRF2 | FEZ1 | 0.3077599 |
| BRF2 | GDI1 | 0.288944122 |
| BRF2 | C1QTNF7 | 0.276260197 |
| BRF2 | PSMD13 | 0.27115772 |
| BRF2 | TGFB3 | 0.266280496 |
| BRF2 | UNKL | 0.257295811 |
| BRF2 | MFSD11 | 0.252554835 |
| BRF2 | MYO1C | 0.251571903 |
| BRF2 | DEXI | 0.251255357 |
| BRF2 | USF2 | 0.245499238 |
| BRF2 | SOCS5 | 0.240023568 |
| BRF2 | CSNK1G2 | 0.23991643 |
| BRF2 | RERE | 0.23874751 |
| BRF2 | LRRC58 | 0.225265259 |
| BRF2 | ADAMTS5 | 0.216263936 |
| BRF2 | GLI1 | 0.207267383 |
| BRF2 | YWHAG | 0.148126056 |
| BRF2 | KIF1C | 0.144405664 |
| BRF2 | PIP4K2B | 0.132538911 |
| BRF2 | ZNF827 | 0.118880757 |
| BRF2 | LDB1 | 0.113279565 |
| BRF2 | SEC24B | 0.112259503 |
| BRF2 | CSK | 0.094958558 |
| BRF2 | LFNG | 0.076224625 |
| BRF2 | VPS13B | 0.07067292 |
| BRF2 | RNF219 | 0.065720471 |
| BRF2 | PTPRS | 0.062448409 |
| BRF2 | DTX2 | 0.056033845 |
| BRF2 | PDE10A | 0.050045831 |
| BRF2 | LOXL4 | 0.045682649 |
| BRF2 | ELF4 | 0.034415228 |
| BRF2 | RREB1 | 0.031534718 |
| BRF2 | RGS17 | 0.013615492 |
| CDX1 | ADRA2A | 12.54693997 |
| CDX1 | TNFSF13B | 6.912471436 |
| CDX1 | RBP5 | 5.810441445 |
| CDX1 | CD82 | 4.990789854 |
| CDX1 | CCL19 | 4.25862092 |
| CDX1 | C11orf74 | 2.544562796 |
| CDX1 | PLCXD3 | 1.838365625 |
| CDX1 | KCTD12 | 1.546333131 |
| CDX1 | VEGFA | 1.341722567 |
| CDX1 | PHYHIPL | 1.1558677 |
| CDX1 | CD200R1 | 1.140045657 |
| CDX1 | RFTN1 | 1.040010202 |
| CDX1 | CDX1 | 1 |
| CDX1 | DLEU1 | 0.955159529 |
| CDX1 | STUB1 | 0.72461712 |
| CDX1 | ZNF415 | 0.723056115 |
| CDX1 | C7 | 0.710519032 |
| CDX1 | SIDT2 | 0.624806758 |
| CDX1 | NAGLU | 0.624174597 |
| CDX1 | LGI2 | 0.448749249 |
| CDX1 | CTSS | 0.438351019 |
| CDX1 | CYGB | 0.356927092 |
| CDX1 | NID2 | 0.310934646 |
| CEBPA | LMNB1 | 5.242976094 |
| CEBPA | GLOD4 | 4.903120957 |
| CEBPA | CENPW | 3.026786581 |
| CEBPA | EEF2 | 2.952749262 |
| CEBPA | PPIG | 2.906239631 |
| CEBPA | DSG1 | 2.382801885 |
| CEBPA | MRPL2 | 2.150794203 |
| CEBPA | PACSIN3 | 1.8850469 |
| CEBPA | GUK1 | 1.883168657 |
| CEBPA | USP11 | 1.70133468 |
| CEBPA | HBP1 | 1.593588708 |
| CEBPA | SFN | 1.563632236 |
| CEBPA | SREK1 | 1.461930719 |
| CEBPA | ATG10 | 1.386832328 |
| CEBPA | FMO1 | 1.299421044 |
| CEBPA | IFNGR1 | 1.201445462 |
| CEBPA | BAX | 1.095693411 |
| CEBPA | HDAC5 | 1.088349558 |
| CEBPA | KCNN3 | 0.97713247 |
| CEBPA | NRXN3 | 0.945570916 |
| CEBPA | ZDHHC2 | 0.941068354 |
| CEBPA | GTF2F1 | 0.938280028 |
| CEBPA | TBC1D2 | 0.928786997 |
| CEBPA | CFDP1 | 0.887872962 |
| CEBPA | TRIM29 | 0.857114804 |
| CEBPA | SUPT6H | 0.850688469 |
| CEBPA | CSNK1G2 | 0.701876714 |
| CEBPA | PTPRF | 0.698533876 |
| CEBPA | NRARP | 0.663483925 |
| CEBPA | RNMT | 0.656405451 |
| CEBPA | PGAP2 | 0.550724372 |
| CEBPA | ACER1 | 0.539316607 |
| CEBPA | C9orf3 | 0.521325581 |
| CEBPA | FAM174A | 0.507097198 |
| CEBPA | HLA-C | 0.506676575 |
| CEBPA | SPRR2D | 0.433609074 |
| CEBPA | LBP | 0.323771735 |
| CEBPA | PHOSPHO2 | 0.322047759 |
| CEBPB | SCGB2A2 | 21.5512662 |
| CEBPB | CD81 | 21.08780347 |
| CEBPB | C11orf96 | 17.35083114 |
| CEBPB | APOE | 16.12196049 |
| CEBPB | JUN | 15.28639697 |
| CEBPB | SERPING1 | 14.26172956 |
| CEBPB | GPX3 | 13.10817659 |
| CEBPB | JUNB | 12.48259021 |
| CEBPB | KLHL15 | 12.28825811 |
| CEBPB | KRT10 | 10.81931716 |
| CEBPB | CDKN1C | 9.744358003 |
| CEBPB | DIP2A | 9.682789983 |
| CEBPB | ZFP36L2 | 9.311109045 |
| CEBPB | ZC3H7B | 9.050059188 |
| CEBPB | KRTDAP | 8.698277997 |
| CEBPB | CAMK2N1 | 8.352141253 |
| CEBPB | BRD2 | 8.232153768 |
| CEBPB | GLUL | 8.192776187 |
| CEBPB | IER2 | 8.163917516 |
| CEBPB | CYR61 | 7.987291437 |
| CEBPB | SQSTM1 | 7.944053945 |
| CEBPB | DMKN | 7.869315829 |
| CEBPB | TPM4 | 7.669057235 |
| CEBPB | S100A8 | 7.462408746 |
| CEBPB | KRT1 | 7.410718092 |
| CEBPB | RASD1 | 7.382290636 |
| CEBPB | TGFBR2 | 7.136675643 |
| CEBPB | PDK4 | 7.078477423 |
| CEBPB | PERP | 6.833607392 |
| CEBPB | TIMP3 | 6.209118027 |
| CEBPB | IGFBP5 | 6.088344461 |
| CEBPB | S100A9 | 5.828507043 |
| CEBPB | CYGB | 5.757535405 |
| CEBPB | SRSF5 | 5.618729783 |
| CEBPB | HEXIM1 | 5.424273932 |
| CEBPB | LY6D | 5.419195227 |
| CEBPB | SOD3 | 5.289947793 |
| CEBPB | MSX1 | 5.238167674 |
| CEBPB | BHLHE40 | 4.948144117 |
| CEBPB | APOD | 4.944818878 |
| CEBPB | SOCS3 | 4.79435362 |
| CEBPB | DUSP1 | 4.660766963 |
| CEBPB | FBLN2 | 4.442990029 |
| CEBPB | STMN2 | 4.354172964 |
| CEBPB | FBLN5 | 4.348968029 |
| CEBPB | STOM | 4.336026162 |
| CEBPB | PRRX1 | 4.233296922 |
| CEBPB | PIK3R1 | 4.227378257 |
| CEBPB | NFIL3 | 4.194672361 |
| CEBPB | FGF7 | 4.179138183 |
| CEBPB | H1FX | 3.977132232 |
| CEBPB | CNIH4 | 3.94705681 |
| CEBPB | ID2 | 3.802817317 |
| CEBPB | NR4A1 | 3.802666287 |
| CEBPB | KLF10 | 3.770307258 |
| CEBPB | CTSO | 3.550082726 |
| CEBPB | RHOBTB3 | 3.467968396 |
| CEBPB | FN1 | 3.416344489 |
| CEBPB | TCF4 | 3.341355079 |
| CEBPB | CD248 | 3.339612983 |
| CEBPB | KLF4 | 3.272130383 |
| CEBPB | PCBP2 | 3.268307281 |
| CEBPB | DAB2 | 3.261401483 |
| CEBPB | CITED2 | 3.14340818 |
| CEBPB | GADD45G | 3.094188751 |
| CEBPB | NCOA1 | 2.973893779 |
| CEBPB | BCL3 | 2.946216201 |
| CEBPB | STEAP4 | 2.914037176 |
| CEBPB | RBPJ | 2.815680845 |
| CEBPB | GEM | 2.803365796 |
| CEBPB | CCDC80 | 2.682548215 |
| CEBPB | FOS | 2.604995745 |
| CEBPB | ZFP36L1 | 2.590617593 |
| CEBPB | SERPINF1 | 2.587296379 |
| CEBPB | VIM | 2.580052503 |
| CEBPB | RUNX1 | 2.562992897 |
| CEBPB | DIO2 | 2.518676234 |
| CEBPB | REEP5 | 2.380798849 |
| CEBPB | TTC8 | 2.357878408 |
| CEBPB | HSPA5 | 2.35426936 |
| CEBPB | SPRY1 | 2.310827105 |
| CEBPB | CBX4 | 2.29715323 |
| CEBPB | RPL7 | 2.294148606 |
| CEBPB | STAT3 | 2.278650772 |
| CEBPB | HAS2 | 2.194165664 |
| CEBPB | ARID5B | 2.1823702 |
| CEBPB | ENPP2 | 2.146309552 |
| CEBPB | CD302 | 2.097204422 |
| CEBPB | PDPN | 2.046409798 |
| CEBPB | DDX6 | 2.038428412 |
| CEBPB | RRBP1 | 1.94941436 |
| CEBPB | RGS2 | 1.922281521 |
| CEBPB | CDC42EP4 | 1.900532216 |
| CEBPB | CPOX | 1.90006074 |
| CEBPB | ECSIT | 1.893792079 |
| CEBPB | HSPB6 | 1.889743304 |
| CEBPB | ID4 | 1.845475028 |
| CEBPB | CGNL1 | 1.840246105 |
| CEBPB | ID3 | 1.796960897 |
| CEBPB | BAMBI | 1.79091773 |
| CEBPB | SEC16A | 1.773128497 |
| CEBPB | CD74 | 1.744162654 |
| CEBPB | CLPX | 1.723658983 |
| CEBPB | PMP22 | 1.715213512 |
| CEBPB | GRAMD1A | 1.711493013 |
| CEBPB | PTMS | 1.704459202 |
| CEBPB | C1orf198 | 1.695697357 |
| CEBPB | NTRK2 | 1.684366618 |
| CEBPB | TMBIM6 | 1.60784188 |
| CEBPB | CCNF | 1.595862589 |
| CEBPB | CTDSPL2 | 1.550171328 |
| CEBPB | DYRK1A | 1.495748797 |
| CEBPB | KLF9 | 1.474044141 |
| CEBPB | SLIT2 | 1.404134217 |
| CEBPB | AKR1B1 | 1.386842129 |
| CEBPB | GADD45A | 1.379687571 |
| CEBPB | FRS2 | 1.342415907 |
| CEBPB | ADRB2 | 1.329953415 |
| CEBPB | BAZ1B | 1.329766656 |
| CEBPB | NUPR1 | 1.323516331 |
| CEBPB | SFPQ | 1.321945366 |
| CEBPB | TRIB3 | 1.316477788 |
| CEBPB | TGFBR3 | 1.303786948 |
| CEBPB | C3orf58 | 1.283248281 |
| CEBPB | BTF3 | 1.279176234 |
| CEBPB | HNRNPL | 1.275200704 |
| CEBPB | PRNP | 1.249423265 |
| CEBPB | ANGPTL1 | 1.236418125 |
| CEBPB | NDUFB6 | 1.222917773 |
| CEBPB | CALM2 | 1.208917878 |
| CEBPB | ITM2A | 1.197921762 |
| CEBPB | TMEM30A | 1.193029473 |
| CEBPB | DBP | 1.161596648 |
| CEBPB | C3 | 1.161447347 |
| CEBPB | KLF2 | 1.149033652 |
| CEBPB | MMP2 | 1.138211259 |
| CEBPB | THY1 | 1.127811315 |
| CEBPB | SH3BGR | 1.122199849 |
| CEBPB | NUFIP2 | 1.113717999 |
| CEBPB | MAFB | 1.111584346 |
| CEBPB | ATF3 | 1.10826889 |
| CEBPB | ANXA1 | 1.10530851 |
| CEBPB | MYO1B | 1.091306646 |
| CEBPB | ABHD6 | 1.081162453 |
| CEBPB | JUND | 1.077439363 |
| CEBPB | MGP | 1.067448863 |
| CEBPB | EXD2 | 1.066378966 |
| CEBPB | PLAGL2 | 1.065817811 |
| CEBPB | LMBRD1 | 1.062803173 |
| CEBPB | CSGALNACT1 | 1.054483408 |
| CEBPB | EEF2 | 1.050917191 |
| CEBPB | ERRFI1 | 1.039269326 |
| CEBPB | LSP1 | 1.026078192 |
| CEBPB | ZNF777 | 1.014521369 |
| CEBPB | ATP11B | 1.00705329 |
| CEBPB | CEBPB | 1 |
| CEBPB | TSC22D3 | 0.999856257 |
| CEBPB | GTF2IRD1 | 0.984648055 |
| CEBPB | DDIT4 | 0.97527598 |
| CEBPB | SOX4 | 0.96771773 |
| CEBPB | MYC | 0.949967016 |
| CEBPB | SLC23A2 | 0.930944978 |
| CEBPB | SETD1B | 0.925540202 |
| CEBPB | TPST1 | 0.900274224 |
| CEBPB | TAF7 | 0.898718578 |
| CEBPB | KDM4A | 0.882005681 |
| CEBPB | PAPD7 | 0.877485852 |
| CEBPB | IFNGR1 | 0.87689434 |
| CEBPB | SNED1 | 0.87653025 |
| CEBPB | NPR1 | 0.862536654 |
| CEBPB | LAMA4 | 0.861750991 |
| CEBPB | ARID1A | 0.85896458 |
| CEBPB | FOSB | 0.858296102 |
| CEBPB | NGFR | 0.849655374 |
| CEBPB | ING1 | 0.832015235 |
| CEBPB | DEDD | 0.823639664 |
| CEBPB | RSPO1 | 0.822539069 |
| CEBPB | CCDC47 | 0.811873404 |
| CEBPB | CNTN1 | 0.804306263 |
| CEBPB | ADIPOR2 | 0.795108299 |
| CEBPB | TMEM131 | 0.788341764 |
| CEBPB | ZIC1 | 0.78226942 |
| CEBPB | PAFAH1B1 | 0.780576721 |
| CEBPB | CCNA2 | 0.780493488 |
| CEBPB | FST | 0.776127449 |
| CEBPB | GSN | 0.770049736 |
| CEBPB | EGR1 | 0.763664459 |
| CEBPB | FAM49A | 0.763050328 |
| CEBPB | EGR2 | 0.752711284 |
| CEBPB | EMP1 | 0.749281894 |
| CEBPB | CTSB | 0.748761755 |
| CEBPB | BHLHE41 | 0.742937281 |
| CEBPB | C9orf152 | 0.730778573 |
| CEBPB | HAND1 | 0.730323693 |
| CEBPB | B3GNT2 | 0.719696069 |
| CEBPB | PDGFRA | 0.703795233 |
| CEBPB | OSBPL1A | 0.702105968 |
| CEBPB | BTG1 | 0.685706884 |
| CEBPB | DNAJA1 | 0.684374962 |
| CEBPB | GPR153 | 0.68370587 |
| CEBPB | USP11 | 0.680151316 |
| CEBPB | P4HB | 0.677449219 |
| CEBPB | KHDRBS3 | 0.675175954 |
| CEBPB | PARD3B | 0.667989582 |
| CEBPB | EMP2 | 0.66683502 |
| CEBPB | USP22 | 0.6533717 |
| CEBPB | CCNL1 | 0.652171997 |
| CEBPB | CCND1 | 0.652105797 |
| CEBPB | RNF10 | 0.651036865 |
| CEBPB | LMNB1 | 0.648986568 |
| CEBPB | PDCD1LG2 | 0.641120788 |
| CEBPB | HMGB1 | 0.629481149 |
| CEBPB | CDX1 | 0.624424493 |
| CEBPB | CPXM1 | 0.620759297 |
| CEBPB | PTDSS2 | 0.617832978 |
| CEBPB | SERPINE2 | 0.607249284 |
| CEBPB | COPS8 | 0.602418985 |
| CEBPB | PHLDA3 | 0.601615883 |
| CEBPB | H1F0 | 0.596721264 |
| CEBPB | CELF1 | 0.590227826 |
| CEBPB | LAMA2 | 0.581264072 |
| CEBPB | ANPEP | 0.57886811 |
| CEBPB | ABCA8 | 0.577365433 |
| CEBPB | SLC24A1 | 0.5757046 |
| CEBPB | COL15A1 | 0.573344452 |
| CEBPB | ASPH | 0.572291833 |
| CEBPB | HNRNPF | 0.56863347 |
| CEBPB | ZNF219 | 0.568625096 |
| CEBPB | DDIT3 | 0.568217593 |
| CEBPB | HMGB3 | 0.562573634 |
| CEBPB | HERPUD1 | 0.562572884 |
| CEBPB | SEMA6C | 0.54712077 |
| CEBPB | IL6 | 0.545492596 |
| CEBPB | SH2B3 | 0.539848977 |
| CEBPB | CYBRD1 | 0.535900048 |
| CEBPB | ZNF385B | 0.535069288 |
| CEBPB | TM9SF4 | 0.534448995 |
| CEBPB | NR2F2 | 0.533373823 |
| CEBPB | TTLL4 | 0.531053677 |
| CEBPB | SLC26A7 | 0.525974542 |
| CEBPB | HSPG2 | 0.518511034 |
| CEBPB | TCEAL4 | 0.514385539 |
| CEBPB | MTDH | 0.510496788 |
| CEBPB | COLEC12 | 0.507556229 |
| CEBPB | VDAC1 | 0.506146722 |
| CEBPB | CCDC74B | 0.504535961 |
| CEBPB | CP | 0.503748757 |
| CEBPB | PJA2 | 0.5003123 |
| CEBPB | SLC40A1 | 0.495306338 |
| CEBPB | TUBA1A | 0.490661225 |
| CEBPB | RTN4 | 0.490110505 |
| CEBPB | GUSBP1 | 0.488054989 |
| CEBPB | CSNK2A2 | 0.486183653 |
| CEBPB | FAM110A | 0.485151804 |
| CEBPB | PEBP1 | 0.482047458 |
| CEBPB | RBM39 | 0.477901784 |
| CEBPB | HVCN1 | 0.472976089 |
| CEBPB | EEPD1 | 0.471132552 |
| CEBPB | L1TD1 | 0.468863907 |
| CEBPB | ZSCAN18 | 0.466175227 |
| CEBPB | LBP | 0.465552537 |
| CEBPB | ARMC2 | 0.463638994 |
| CEBPB | ZNF217 | 0.463630048 |
| CEBPB | WFDC2 | 0.46330841 |
| CEBPB | L3MBTL3 | 0.459386825 |
| CEBPB | MAOB | 0.455819716 |
| CEBPB | HAPLN1 | 0.452171439 |
| CEBPB | LTBP1 | 0.447895296 |
| CEBPB | KCNK2 | 0.440831073 |
| CEBPB | LRP1B | 0.438139811 |
| CEBPD | MT2A | 27.13615826 |
| CEBPD | MYC | 25.16153524 |
| CEBPD | JUNB | 25.14205931 |
| CEBPD | GLUL | 16.36495772 |
| CEBPD | GADD45A | 16.07160531 |
| CEBPD | GADD45B | 13.8395169 |
| CEBPD | KLF9 | 12.00447086 |
| CEBPD | SOD3 | 11.97601827 |
| CEBPD | ISYNA1 | 11.37728737 |
| CEBPD | GPX3 | 11.31766708 |
| CEBPD | SOCS3 | 11.21597936 |
| CEBPD | NAMPT | 10.41627609 |
| CEBPD | CEBPB | 9.569855895 |
| CEBPD | EGR1 | 9.438175501 |
| CEBPD | CFD | 9.347163107 |
| CEBPD | ARID5B | 8.763886397 |
| CEBPD | SRSF5 | 8.486471008 |
| CEBPD | ENPP2 | 8.451353695 |
| CEBPD | KLF4 | 8.384365079 |
| CEBPD | TSC22D3 | 8.29655797 |
| CEBPD | PMP22 | 8.177090263 |
| CEBPD | JUN | 7.85000503 |
| CEBPD | CITED2 | 7.317299471 |
| CEBPD | SERPING1 | 6.684006895 |
| CEBPD | PIK3R1 | 6.34542185 |
| CEBPD | JUND | 6.310322215 |
| CEBPD | ZFP36L2 | 6.274580838 |
| CEBPD | CXCL2 | 6.269299205 |
| CEBPD | TIMP3 | 6.188375519 |
| CEBPD | NFKBIA | 6.158121656 |
| CEBPD | C11orf96 | 6.042821467 |
| CEBPD | SPTBN1 | 6.038934948 |
| CEBPD | TLE4 | 6.008355505 |
| CEBPD | BTG1 | 5.948650034 |
| CEBPD | SNHG8 | 5.815961707 |
| CEBPD | HTRA1 | 5.784643362 |
| CEBPD | CYBRD1 | 5.705739166 |
| CEBPD | CDKN1A | 5.67351038 |
| CEBPD | ARF1 | 5.529016491 |
| CEBPD | MDK | 5.382304363 |
| CEBPD | IER2 | 5.347377905 |
| CEBPD | CCNL1 | 5.167610635 |
| CEBPD | SPRY1 | 4.974850372 |
| CEBPD | ZFAND5 | 4.899666672 |
| CEBPD | RPL39 | 4.863506553 |
| CEBPD | CD44 | 4.737671022 |
| CEBPD | STEAP4 | 4.699480085 |
| CEBPD | PSMA5 | 4.692150252 |
| CEBPD | UBE2A | 4.691518452 |
| CEBPD | UGCG | 4.408260587 |
| CEBPD | H2AFZ | 4.293964986 |
| CEBPD | BGN | 4.181873473 |
| CEBPD | HSP90AB1 | 4.172207984 |
| CEBPD | STAT3 | 4.14399783 |
| CEBPD | HSPA1A | 3.981630472 |
| CEBPD | SVEP1 | 3.858563662 |
| CEBPD | ZFP36 | 3.817014704 |
| CEBPD | IL6ST | 3.799770586 |
| CEBPD | DDIT4 | 3.747708666 |
| CEBPD | DUSP1 | 3.69710422 |
| CEBPD | LBH | 3.629029491 |
| CEBPD | UBC | 3.429150135 |
| CEBPD | CIRBP | 3.424344191 |
| CEBPD | GPNMB | 3.258827297 |
| CEBPD | IL6 | 3.148701581 |
| CEBPD | TWIST2 | 3.069146812 |
| CEBPD | HSPB6 | 3.056248543 |
| CEBPD | CH25H | 3.03620124 |
| CEBPD | PDK4 | 2.941689364 |
| CEBPD | DDX5 | 2.866447102 |
| CEBPD | FOSB | 2.816755814 |
| CEBPD | PCF11 | 2.790859161 |
| CEBPD | APLP2 | 2.691639526 |
| CEBPD | ABHD14A | 2.682940545 |
| CEBPD | CHRDL1 | 2.676578715 |
| CEBPD | C3 | 2.657293219 |
| CEBPD | LOX | 2.581779381 |
| CEBPD | COX5B | 2.506566772 |
| CEBPD | AKAP12 | 2.492094438 |
| CEBPD | ERRFI1 | 2.459682755 |
| CEBPD | NFKBIZ | 2.439352446 |
| CEBPD | CFH | 2.436506463 |
| CEBPD | BRD2 | 2.430633764 |
| CEBPD | LPCAT1 | 2.389267148 |
| CEBPD | EIF1 | 2.313757822 |
| CEBPD | CYR61 | 2.300937276 |
| CEBPD | S100A9 | 2.287701496 |
| CEBPD | MGP | 2.28057287 |
| CEBPD | PTPRJ | 2.251512819 |
| CEBPD | ADAM12 | 2.213560557 |
| CEBPD | FKBP5 | 2.189250843 |
| CEBPD | CD9 | 2.146116626 |
| CEBPD | TSPO | 2.063323628 |
| CEBPD | KLF10 | 2.058871227 |
| CEBPD | MCL1 | 2.055173296 |
| CEBPD | CEP135 | 1.990122994 |
| CEBPD | IRF1 | 1.986219648 |
| CEBPD | ZDHHC14 | 1.963876805 |
| CEBPD | GRM7 | 1.962597247 |
| CEBPD | SFXN1 | 1.919710695 |
| CEBPD | RHOB | 1.906536703 |
| CEBPD | CCNI | 1.872283038 |
| CEBPD | UBE2D3 | 1.863964728 |
| CEBPD | ATP6V0E1 | 1.80595725 |
| CEBPD | RBM39 | 1.773138023 |
| CEBPD | MXD3 | 1.764176073 |
| CEBPD | ID2 | 1.743301622 |
| CEBPD | GPX8 | 1.705194633 |
| CEBPD | EIF4A2 | 1.678338967 |
| CEBPD | MAB21L1 | 1.647866185 |
| CEBPD | ISLR | 1.625869318 |
| CEBPD | ADAMTS2 | 1.6184507 |
| CEBPD | ANAPC11 | 1.591032316 |
| CEBPD | ADM | 1.569186902 |
| CEBPD | SYNE1 | 1.551903436 |
| CEBPD | STAC2 | 1.551789626 |
| CEBPD | FOS | 1.548900131 |
| CEBPD | ASPH | 1.526914552 |
| CEBPD | PSMB5 | 1.525176854 |
| CEBPD | PPP1R15A | 1.508602154 |
| CEBPD | RND3 | 1.455416759 |
| CEBPD | CCL8 | 1.436160802 |
| CEBPD | NR2F2 | 1.429028718 |
| CEBPD | CCDC47 | 1.410027194 |
| CEBPD | APPL1 | 1.404349122 |
| CEBPD | HAS2 | 1.402724507 |
| CEBPD | NOTCH2 | 1.365245949 |
| CEBPD | TRAM1 | 1.350211584 |
| CEBPD | FBLN2 | 1.2966177 |
| CEBPD | TXN | 1.284049463 |
| CEBPD | ANGPTL2 | 1.283020271 |
| CEBPD | HERPUD1 | 1.281285982 |
| CEBPD | IER5L | 1.278608017 |
| CEBPD | H2AFV | 1.273277081 |
| CEBPD | GSN | 1.267863251 |
| CEBPD | LPXN | 1.255911705 |
| CEBPD | ADRA2A | 1.254785521 |
| CEBPD | FBLN1 | 1.236267837 |
| CEBPD | CSGALNACT1 | 1.226048274 |
| CEBPD | SEC31A | 1.210324996 |
| CEBPD | LDHB | 1.198112489 |
| CEBPD | MSC | 1.194651052 |
| CEBPD | COL12A1 | 1.186588123 |
| CEBPD | NR4A1 | 1.160611471 |
| CEBPD | NDC80 | 1.160042659 |
| CEBPD | ANXA1 | 1.145726356 |
| CEBPD | MAPRE1 | 1.128530613 |
| CEBPD | FBLN5 | 1.11619907 |
| CEBPD | SIVA1 | 1.114866594 |
| CEBPD | PID1 | 1.11254266 |
| CEBPD | CALM2 | 1.112148506 |
| CEBPD | PLK2 | 1.08345002 |
| CEBPD | TOB2 | 1.083351873 |
| CEBPD | RPN2 | 1.077821244 |
| CEBPD | ZFP36L1 | 1.074708718 |
| CEBPD | SNED1 | 1.061177187 |
| CEBPD | WTAP | 1.050658202 |
| CEBPD | NEGR1 | 1.024212927 |
| CEBPD | LAMB1 | 1.02268566 |
| CEBPD | TUBB2A | 1.010929251 |
| CEBPD | NDUFS4 | 1.010145612 |
| CEBPD | FLNA | 1.009453643 |
| CEBPD | MEX3A | 1.00334473 |
| CEBPD | RPA2 | 0.990709266 |
| CEBPD | TCEAL7 | 0.986242852 |
| CEBPD | ASNSD1 | 0.958997152 |
| CEBPD | GNG12 | 0.955981368 |
| CEBPD | COLEC12 | 0.953887504 |
| CEBPD | OLFML3 | 0.947820704 |
| CEBPD | CCDC92 | 0.942857682 |
| CEBPD | PELI2 | 0.942741658 |
| CEBPD | HSPA14 | 0.92862639 |
| CEBPD | CP | 0.927039827 |
| CEBPD | VAMP2 | 0.926873978 |
| CEBPD | VCAN | 0.925883579 |
| CEBPD | KCTD12 | 0.921804666 |
| CEBPD | SOX4 | 0.915928967 |
| CEBPD | PHF13 | 0.91473386 |
| CEBPD | SBDS | 0.904893338 |
| CEBPD | MAGED1 | 0.896024316 |
| CEBPD | FZD10 | 0.893835332 |
| CEBPD | MTDH | 0.887374186 |
| CEBPD | PDGFRA | 0.88711144 |
| CEBPD | CALML5 | 0.878561458 |
| CEBPD | CHI3L2 | 0.866987442 |
| CEBPD | SNAI2 | 0.865886266 |
| CEBPD | FABP5 | 0.865494185 |
| CEBPD | COL15A1 | 0.865486245 |
| CEBPD | PLXDC1 | 0.857639965 |
| CEBPD | GADD45G | 0.852218751 |
| CEBPD | NFIB | 0.839441591 |
| CEBPD | ENKUR | 0.836002601 |
| CEBPD | LPAR1 | 0.810177085 |
| CEBPD | BZW1 | 0.799571306 |
| CEBPD | CMKLR1 | 0.787642309 |
| CEBPD | S100A13 | 0.78748793 |
| CEBPD | CDH24 | 0.785486472 |
| CEBPD | CPXM1 | 0.785157179 |
| CEBPD | VDAC1 | 0.784456183 |
| CEBPD | ZBTB20 | 0.781383974 |
| CEBPD | ATF3 | 0.761470453 |
| CEBPD | RNF39 | 0.760997739 |
| CEBPD | GRIK2 | 0.757866208 |
| CEBPD | RSRC2 | 0.753416505 |
| CEBPD | FOXO3 | 0.746409467 |
| CEBPD | HAS3 | 0.74409247 |
| CEBPD | CPVL | 0.739995649 |
| CEBPD | BMP1 | 0.737323837 |
| CEBPD | RPL24 | 0.73722692 |
| CEBPD | IL11 | 0.736120419 |
| CEBPD | TMBIM6 | 0.733390964 |
| CEBPD | MORF4L2 | 0.732608849 |
| CEBPD | COL4A2 | 0.731294922 |
| CEBPD | PLEKHH2 | 0.726768591 |
| CEBPD | GJA1 | 0.721703419 |
| CEBPD | CSTA | 0.716264887 |
| CEBPD | ABAT | 0.708692486 |
| CEBPD | CCDC144B | 0.704442307 |
| CEBPD | PSMG2 | 0.695030428 |
| CEBPD | NUFIP2 | 0.693635498 |
| CEBPD | SLC10A6 | 0.687736455 |
| CEBPD | ANP32A | 0.686498553 |
| CEBPD | COX6A1 | 0.684327663 |
| CEBPD | MYADM | 0.682614312 |
| CEBPD | S100A8 | 0.682236393 |
| CEBPD | MLF2 | 0.677900742 |
| CEBPD | PATZ1 | 0.676888464 |
| CEBPD | COL18A1 | 0.674974308 |
| CEBPD | SOX13 | 0.659519269 |
| CEBPD | HNRNPA2B1 | 0.657391475 |
| CEBPD | CCDC146 | 0.656912558 |
| CEBPD | SBSN | 0.649712068 |
| CEBPD | IGFBP2 | 0.647985149 |
| CEBPD | ID1 | 0.643810244 |
| CEBPD | KHDRBS3 | 0.643070888 |
| CEBPD | CREB5 | 0.639976503 |
| CEBPD | OAF | 0.639911763 |
| CEBPD | EXOC6 | 0.639856393 |
| CEBPD | GCNT1 | 0.639295983 |
| CEBPD | ALDH1A1 | 0.636273317 |
| CEBPD | SMARCA1 | 0.635136862 |
| CEBPD | GNAI1 | 0.630152675 |
| CEBPD | MIR155HG | 0.628480631 |
| CEBPD | SERBP1 | 0.622142527 |
| CEBPD | KLF2 | 0.620581006 |
| CEBPD | VIM | 0.617161478 |
| CEBPD | CCT2 | 0.61611938 |
| CEBPD | HNRNPA0 | 0.614850921 |
| CEBPD | RBMX | 0.608035794 |
| CEBPD | IFNGR1 | 0.605975291 |
| CEBPD | PHYHIPL | 0.60263017 |
| CEBPD | ALDH1A2 | 0.602560146 |
| CEBPD | HOXA4 | 0.600774453 |
| CEBPD | GYG1 | 0.600615429 |
| CEBPD | ANKRD46 | 0.597545495 |
| CEBPD | SCNN1A | 0.59401721 |
| CEBPD | NFIL3 | 0.587956254 |
| CEBPD | RPS9 | 0.586887761 |
| CEBPD | SPRR1B | 0.586849614 |
| CEBPD | INHBA | 0.584016426 |
| CEBPD | ABCA8 | 0.582687663 |
| CEBPD | FOSL2 | 0.582685222 |
| CEBPD | MCOLN3 | 0.582324099 |
| CEBPD | PROX1 | 0.578987234 |
| CEBPD | PTGS2 | 0.576943072 |
| CEBPD | PFN1 | 0.576070848 |
| CEBPD | ID4 | 0.573457903 |
| CEBPD | PTGR2 | 0.57039029 |
| CEBPD | GFRA2 | 0.570279893 |
| CEBPD | EFEMP1 | 0.56279121 |
| CEBPD | H1FX | 0.560067729 |
| CEBPD | CALCA | 0.558678964 |
| CEBPD | PPP1CC | 0.557877672 |
| CEBPD | LRP1 | 0.55617324 |
| CEBPD | HNRNPD | 0.553367799 |
| CEBPD | NRXN2 | 0.549572741 |
| CEBPD | GHITM | 0.546371332 |
| CEBPD | ADCYAP1 | 0.543784313 |
| CEBPD | ZFYVE9 | 0.540169426 |
| CEBPD | ATF4 | 0.532616571 |
| CEBPD | FXR1 | 0.532448486 |
| CEBPD | NUP188 | 0.532062298 |
| CEBPD | SURF4 | 0.530255604 |
| CEBPD | ASB1 | 0.528500299 |
| CEBPD | LRFN3 | 0.528295472 |
| CEBPD | PPDPF | 0.527430712 |
| CEBPD | TPM4 | 0.524214628 |
| CEBPD | HNRNPA3 | 0.524096883 |
| CEBPD | PDLIM2 | 0.521554291 |
| CEBPD | EDARADD | 0.521501119 |
| CEBPD | STMN2 | 0.51965402 |
| CEBPD | NFIX | 0.518523585 |
| CEBPD | RPS29 | 0.518238546 |
| CEBPD | SLIT3 | 0.513411607 |
| CEBPD | SLC25A15 | 0.510566307 |
| CEBPD | AATF | 0.509487515 |
| CEBPD | RPS27L | 0.505058525 |
| CEBPD | PTGES3 | 0.503896859 |
| CEBPD | KCNA5 | 0.503295098 |
| CEBPD | UBE2S | 0.492481229 |
| CEBPD | CDH11 | 0.492195555 |
| CEBPD | DDX3X | 0.488565969 |
| CEBPD | EDNRB | 0.488075045 |
| CEBPD | ANKRD13B | 0.484470478 |
| CEBPD | NDUFA4 | 0.484397553 |
| CEBPD | NFKBID | 0.4805514 |
| CEBPD | EIF5 | 0.478517726 |
| CEBPD | FGFR1 | 0.470451791 |
| CEBPD | C16orf58 | 0.469842719 |
| CEBPD | FNBP4 | 0.467054435 |
| CEBPD | PFKL | 0.464495311 |
| CEBPD | NDUFB4 | 0.464394955 |
| CEBPD | PALLD | 0.463702263 |
| CEBPD | KRTDAP | 0.463459474 |
| CEBPD | CDC42EP4 | 0.460399111 |
| CEBPD | WDR91 | 0.459555754 |
| CEBPD | PCMTD2 | 0.457607946 |
| CEBPD | SUMO3 | 0.456046414 |
| CEBPD | MRPS30 | 0.455455797 |
| CEBPD | SRP14 | 0.455286542 |
| CEBPD | LY6D | 0.45438505 |
| CEBPD | NACC2 | 0.454043124 |
| CEBPD | RRBP1 | 0.453679418 |
| CEBPD | MYCN | 0.451402018 |
| CEBPD | CDC42 | 0.448768593 |
| CEBPD | H1F0 | 0.447175482 |
| CEBPD | OSMR | 0.446170713 |
| CEBPD | PHIP | 0.445855879 |
| CEBPD | LTBP1 | 0.443485469 |
| CEBPD | EEF2 | 0.441229601 |
| CEBPD | PRCD | 0.435118042 |
| CEBPD | KRT10 | 0.435026978 |
| CEBPG | EIF5A | 4.980895078 |
| CEBPG | LY6D | 4.56388032 |
| CEBPG | TUBA4A | 2.447942383 |
| CEBPG | CDK1 | 2.133034429 |
| CEBPG | TRA2B | 2.103694091 |
| CEBPG | AK3 | 2.062715467 |
| CEBPG | NDRG4 | 1.764725689 |
| CEBPG | FOS | 1.619740627 |
| CEBPG | NOD2 | 1.455937344 |
| CEBPG | LY6G6C | 1.446382427 |
| CEBPG | PIM1 | 1.20768466 |
| CEBPG | HNRNPA3 | 1.048833732 |
| CEBPG | DENND2D | 1.038728165 |
| CEBPG | CFDP1 | 1.021210793 |
| CEBPG | RALGPS2 | 1.002405809 |
| CEBPG | CEBPG | 1 |
| CEBPG | ARPC5L | 0.89357817 |
| CEBPG | POMP | 0.891597217 |
| CEBPG | DGAT2 | 0.841228573 |
| CEBPG | GPC3 | 0.833105218 |
| CEBPG | C1orf21 | 0.726440219 |
| CEBPG | SUMO2 | 0.698105597 |
| CEBPG | PTK2B | 0.694022202 |
| CEBPG | PLEKHH3 | 0.678942493 |
| CEBPG | TFAP2B | 0.663127256 |
| CEBPG | TNF | 0.633057488 |
| CEBPG | TPX2 | 0.604723011 |
| CEBPG | PRKCD | 0.604702622 |
| CEBPG | RAB3A | 0.59951963 |
| CEBPG | RABEPK | 0.56574026 |
| CEBPG | OPA3 | 0.511167852 |
| CEBPG | MEF2C | 0.511043157 |
| CEBPG | PPP1R15A | 0.481192847 |
| CEBPG | CSRP2 | 0.445837224 |
| CEBPG | ZFAT | 0.437062687 |
| CEBPG | DAGLA | 0.436599936 |
| CEBPG | ITGB8 | 0.389847295 |
| CEBPG | LRFN3 | 0.386304753 |
| CEBPG | DTX1 | 0.350259852 |
| CHD1 | RPS16 | 6.884703985 |
| CHD1 | TCEAL8 | 6.239251392 |
| CHD1 | ENHO | 3.130897013 |
| CHD1 | ARHGEF6 | 2.655548311 |
| CHD1 | EHMT2 | 1.670782885 |
| CHD1 | ARHGAP21 | 1.639245713 |
| CHD1 | TGFB1 | 1.615595058 |
| CHD1 | MAML3 | 1.585642946 |
| CHD1 | SEMA4A | 1.425418558 |
| CHD1 | CTBP2 | 1.371338488 |
| CHD1 | PLCB4 | 1.348504517 |
| CHD1 | STAT6 | 1.301774267 |
| CHD1 | PSMD14 | 1.228300383 |
| CHD1 | CD93 | 1.009792297 |
| CHD1 | THNSL1 | 0.978519143 |
| CHD1 | MLLT6 | 0.907714563 |
| CHD1 | DGKZ | 0.900374226 |
| CHD1 | ATXN1L | 0.882748658 |
| CHD1 | NCAM2 | 0.866922549 |
| CHD1 | YAP1 | 0.859753245 |
| CHD1 | JARID2 | 0.67726124 |
| CHD1 | PTPRB | 0.635021345 |
| CHD1 | ZNF385B | 0.548643817 |
| CHD1 | AVPR1A | 0.343971274 |
| CIC | SYTL2 | 1.813330258 |
| CIC | GRB10 | 1.802864713 |
| CIC | SURF6 | 1.50209359 |
| CIC | MAP4 | 1.115906499 |
| CPEB1 | TLE2 | 2.408264416 |
| CPEB1 | UBE2R2 | 0.794142461 |
| CPEB1 | AXIN1 | 0.577747086 |
| CPEB1 | TCF7L2 | 0.47952198 |
| CPEB1 | PRCC | 0.448872086 |
| CPEB1 | NACC2 | 0.444297245 |
| CPEB1 | RCL1 | 0.419053831 |
| CPEB1 | ZNF598 | 0.316883308 |
| CPEB1 | RABIF | 0.206073034 |
| CPEB1 | TMEM222 | 0.181487601 |
| CPEB1 | DPAGT1 | 0.153581635 |
| CPEB1 | INPP5F | 0.153097306 |
| CPSF4 | PBRM1 | 2.207071691 |
| CPSF4 | MYC | 2.00552061 |
| CPSF4 | DUSP22 | 1.649064321 |
| CPSF4 | AMOT | 1.506584452 |
| CPSF4 | EBF1 | 1.416317121 |
| CPSF4 | ABLIM1 | 1.346378069 |
| CPSF4 | ADAMTS1 | 1.293894099 |
| CPSF4 | EMP3 | 1.110804702 |
| CPSF4 | CALD1 | 1.09260762 |
| CPSF4 | KLF10 | 0.969518128 |
| CPSF4 | MKLN1 | 0.938263236 |
| CPSF4 | NR4A3 | 0.932911144 |
| CPSF4 | ZEB2 | 0.914067316 |
| CPSF4 | ADAMTS12 | 0.698595265 |
| CPSF4 | AKAP9 | 0.548816848 |
| CPSF4 | SLC7A8 | 0.421319403 |
| CPSF4 | VGLL4 | 0.388288538 |
| CPSF4 | POU2F2 | 0.379644788 |
| CPSF4 | RPRD2 | 0.341298747 |
| CPSF4 | TCEA3 | 0.289962567 |
| CPSF4 | CACNB3 | 0.277504804 |
| CPSF4 | ASXL1 | 0.271522101 |
| CPSF4 | HOXB7 | 0.17932862 |
| CPSF4 | TP63 | 0.177657528 |
| CPSF4 | ZNF384 | 0.145718715 |
| CPSF4 | NYNRIN | 0.140641847 |
| CPSF4 | IKZF2 | 0.139207538 |
| CPSF4 | ABCD1 | 0.09364643 |
| CPSF4 | CACNB2 | 0.089362109 |
| CPSF4 | GRM7 | 0.062313656 |
| CPSF4 | CHD2 | 0.004004045 |
| CREB1 | C8orf59 | 3.74033434 |
| CREB1 | PIP4K2A | 3.24188952 |
| CREB1 | NEIL2 | 2.759869685 |
| CREB1 | NDUFB6 | 2.570331313 |
| CREB1 | PLEKHH2 | 2.004235744 |
| CREB1 | TBP | 1.873911307 |
| CREB1 | GZF1 | 1.788375673 |
| CREB1 | LSM4 | 1.781598046 |
| CREB1 | METTL5 | 1.529564205 |
| CREB1 | PKP3 | 1.416389015 |
| CREB1 | PRELID1 | 1.244667447 |
| CREB1 | CAST | 1.209203292 |
| CREB1 | PPTC7 | 1.166430808 |
| CREB1 | SNW1 | 1.159062157 |
| CREB1 | LMAN2 | 1.143517777 |
| CREB1 | OST4 | 1.100821192 |
| CREB1 | TM9SF3 | 1.088771877 |
| CREB1 | P4HA2 | 1.079946224 |
| CREB1 | CUEDC1 | 1.068529823 |
| CREB1 | CREB1 | 1 |
| CREB1 | HAS2 | 0.965040725 |
| CREB1 | POLR2J2 | 0.931027457 |
| CREB1 | SMARCA5 | 0.922365552 |
| CREB1 | SEC24B | 0.871980017 |
| CREB1 | CLTB | 0.815360742 |
| CREB1 | SLC39A13 | 0.792054465 |
| CREB1 | SLC38A10 | 0.782069699 |
| CREB1 | SNAI1 | 0.776914536 |
| CREB1 | VPS11 | 0.758330719 |
| CREB1 | RPS19BP1 | 0.756264582 |
| CREB1 | AXIN2 | 0.737427881 |
| CREB1 | BCL10 | 0.712141692 |
| CREB1 | ATG16L2 | 0.67454403 |
| CREB1 | XRN2 | 0.64374451 |
| CREB1 | LYPLA2 | 0.635258507 |
| CREB1 | DHX15 | 0.631132716 |
| CREB1 | TIAM2 | 0.62847903 |
| CREB1 | MTSS1 | 0.61873558 |
| CREB1 | DDOST | 0.610600735 |
| CREB1 | CHIC2 | 0.59712767 |
| CREB1 | RPS3A | 0.56642862 |
| CREB1 | RPL28 | 0.562786543 |
| CREB1 | ZFC3H1 | 0.55854729 |
| CREB1 | CLTC | 0.538005883 |
| CREB1 | PDSS1 | 0.493392318 |
| CREB1 | PLEC | 0.493336944 |
| CREB1 | ALG6 | 0.483887713 |
| CREB1 | HAUS5 | 0.475479593 |
| CREB1 | MYBBP1A | 0.471610528 |
| CREB1 | TMEM164 | 0.469166385 |
| CREB1 | DMTF1 | 0.446209006 |
| CREB1 | ALG10B | 0.444540412 |
| CREB1 | GNPTG | 0.443144172 |
| CREB1 | SF3B3 | 0.424315239 |
| CREB1 | SMCR8 | 0.415350238 |
| CREB1 | COL4A5 | 0.414161854 |
| CREB1 | GRPEL2 | 0.402130929 |
| CREB1 | PMS2CL | 0.397945137 |
| CREB1 | AEN | 0.351780705 |
| CREB1 | FAM120A | 0.345117632 |
| CREB1 | NUDT21 | 0.338078552 |
| CREB1 | HMOX2 | 0.333550468 |
| CREB1 | CBL | 0.31564368 |
| CREB1 | TRAPPC1 | 0.315254839 |
| CREB1 | FAM96A | 0.30946949 |
| CREB1 | SCYL2 | 0.295927356 |
| CREB1 | GCC1 | 0.295692303 |
| CREB1 | MID1 | 0.286605518 |
| CREB1 | CSNK2A1 | 0.268742758 |
| CREB1 | PAPD4 | 0.266554753 |
| CREB1 | TRAPPC2 | 0.262015992 |
| CREB1 | ADARB1 | 0.261706302 |
| CREB1 | NDST1 | 0.260951158 |
| CREB1 | TTC3P1 | 0.25982609 |
| CREB1 | MED14 | 0.255014581 |
| CREB1 | FAM173A | 0.244449948 |
| CREB1 | ABCA1 | 0.242292256 |
| CREB1 | SIRT2 | 0.241793367 |
| CREB1 | C11orf95 | 0.240496306 |
| CREB1 | FAM84A | 0.231993896 |
| CREB1 | ARPC5L | 0.231506122 |
| CREB1 | PPP1R12B | 0.230420657 |
| CREB1 | TMEM130 | 0.228248696 |
| CREB1 | STXBP5 | 0.227525221 |
| CREB1 | PIGZ | 0.224591805 |
| CREB1 | RHOBTB2 | 0.224120222 |
| CREB1 | ITPA | 0.223308447 |
| CREB1 | MAP3K7 | 0.216152189 |
| CREB1 | TMCO4 | 0.21515697 |
| CREB1 | CELF2 | 0.21094134 |
| CREB1 | SPRY2 | 0.206210449 |
| CREB1 | RARG | 0.205160444 |
| CREB1 | NCKAP5L | 0.205018715 |
| CREB1 | WDR26 | 0.203371866 |
| CREB1 | SOCS2 | 0.197184942 |
| CREB1 | DHX16 | 0.193193643 |
| CREB1 | MED6 | 0.189038827 |
| CREB1 | MLLT6 | 0.187585162 |
| CREB1 | BCLAF1 | 0.187042132 |
| CREB1 | GGA1 | 0.183059582 |
| CREB1 | ITFG2 | 0.172846721 |
| CREB1 | EFNA5 | 0.172325683 |
| CREB1 | C15orf39 | 0.170800567 |
| CREB1 | XAB2 | 0.16912912 |
| CREB1 | SHISA4 | 0.151542092 |
| CREB1 | AGPAT3 | 0.150634127 |
| CREB1 | IRF2BP1 | 0.149519665 |
| CREB1 | SGMS1 | 0.146716215 |
| CREB1 | METTL3 | 0.140791699 |
| CREB1 | ZNF426 | 0.139608367 |
| CREB1 | TMEM101 | 0.138450704 |
| CREB1 | NSUN3 | 0.138365468 |
| CREB1 | NECAP2 | 0.137742191 |
| CREB1 | CYB561D2 | 0.125399236 |
| CREB1 | PDPK1 | 0.121941121 |
| CREB1 | CPSF7 | 0.120442562 |
| CREB1 | BAZ2A | 0.108454496 |
| CREB1 | CDC34 | 0.103269806 |
| CREB1 | DLEU2 | 0.091514936 |
| CREB1 | SLC37A4 | 0.080067878 |
| CREB1 | PDE12 | 0.074994198 |
| CREB1 | DHX37 | 0.070122922 |
| CREB1 | SEMA4A | 0.067438042 |
| CREB1 | TTC30A | 0.039787862 |
| CREB1 | WNT5B | 0.014389258 |
| CREB1 | NAV1 | 0.011036187 |
| CREB1 | AP1AR | 0.006614647 |
| CREB1 | STRBP | 0.005518854 |
| CREB1 | DLL1 | 0.004282103 |
| CREB1 | LASP1 | 0.001259509 |
| CREB1 | ARFIP1 | 0.001178713 |
| CREB1 | EDEM3 | 0.001095005 |
| CREB1 | ADIPOR2 | 0.000296687 |
| CREB1 | ARHGEF4 | 1.58E-19 |
| CREB3 | GTF2H1 | 5.234144931 |
| CREB3 | FMOD | 4.852641036 |
| CREB3 | CTSA | 3.808022609 |
| CREB3 | LEPROT | 3.068204792 |
| CREB3 | UXT | 2.921970821 |
| CREB3 | PPFIBP1 | 2.833039886 |
| CREB3 | WISP2 | 2.770878166 |
| CREB3 | FRMD6 | 2.3319353 |
| CREB3 | KIAA0895L | 2.286145962 |
| CREB3 | C20orf27 | 2.153391154 |
| CREB3 | FUCA2 | 2.108017075 |
| CREB3 | MREG | 1.965915317 |
| CREB3 | FDXACB1 | 1.833530565 |
| CREB3 | SSR3 | 1.815625841 |
| CREB3 | NCK1 | 1.781985577 |
| CREB3 | TMBIM6 | 1.696091467 |
| CREB3 | ZCRB1 | 1.683187748 |
| CREB3 | PRDX4 | 1.600166771 |
| CREB3 | ARF4 | 1.556782336 |
| CREB3 | CMTM3 | 1.474738263 |
| CREB3 | ADAMTS14 | 1.40159547 |
| CREB3 | STK11 | 1.377282238 |
| CREB3 | LBH | 1.360925547 |
| CREB3 | PRC1 | 1.353163315 |
| CREB3 | DST | 1.350748546 |
| CREB3 | PRRX2 | 1.286674516 |
| CREB3 | NFX1 | 1.209347361 |
| CREB3 | NADK | 1.147023106 |
| CREB3 | SMAD7 | 1.132809226 |
| CREB3 | SYBU | 1.116209335 |
| CREB3 | SACS | 1.114651478 |
| CREB3 | ACACA | 1.087504898 |
| CREB3 | WDR35 | 1.083055879 |
| CREB3 | EP300 | 1.054470422 |
| CREB3 | HOXB2 | 1.041417887 |
| CREB3 | MCOLN1 | 1.03580391 |
| CREB3 | RRAD | 1.0146955 |
| CREB3 | IGF2R | 1.013080516 |
| CREB3 | CREB3 | 1 |
| CREB3 | STK17B | 0.992548014 |
| CREB3 | NDRG3 | 0.991536394 |
| CREB3 | MAGOHB | 0.951100675 |
| CREB3 | ATP6V1B2 | 0.912952964 |
| CREB3 | GPRC5A | 0.877093234 |
| CREB3 | MOGS | 0.857629957 |
| CREB3 | SEC23A | 0.849284231 |
| CREB3 | IGDCC4 | 0.821410221 |
| CREB3 | SIX4 | 0.803713858 |
| CREB3 | CCDC12 | 0.797142362 |
| CREB3 | PEF1 | 0.762119453 |
| CREB3 | MUC1 | 0.729210967 |
| CREB3 | SF3B1 | 0.726362224 |
| CREB3 | DAB2 | 0.712180539 |
| CREB3 | SPIB | 0.697952473 |
| CREB3 | CLASP1 | 0.682293271 |
| CREB3 | HS6ST1 | 0.672268249 |
| CREB3 | RWDD3 | 0.668175829 |
| CREB3 | MPV17 | 0.65444035 |
| CREB3 | PJA2 | 0.65390657 |
| CREB3 | KLHL7 | 0.64127265 |
| CREB3 | MAGED1 | 0.60233961 |
| CREB3 | TSC1 | 0.599425549 |
| CREB3 | ATP6V1G1 | 0.595886007 |
| CREB3 | ZFAND5 | 0.586727135 |
| CREB3 | CC2D2A | 0.586204738 |
| CREB3 | IL33 | 0.583372029 |
| CREB3 | CDC14A | 0.582270512 |
| CREB3 | MIA3 | 0.551716642 |
| CREB3 | TFG | 0.538460729 |
| CREB3 | PAPD7 | 0.527813357 |
| CREB3 | TMEM140 | 0.501692529 |
| CREB3 | KDELR2 | 0.490477269 |
| CREB3 | GOLPH3 | 0.490055502 |
| CREB3 | COX6C | 0.480579896 |
| CREB3 | RABAC1 | 0.436742297 |
| CREB3 | PITPNM3 | 0.329461829 |
| CREB3 | GAN | 0.274727947 |
| CREB3L1 | COL1A1 | 28.1809268 |
| CREB3L1 | COL11A1 | 22.92079155 |
| CREB3L1 | CLEC11A | 21.81348936 |
| CREB3L1 | SPARC | 18.05260634 |
| CREB3L1 | HAPLN1 | 17.97558065 |
| CREB3L1 | RRBP1 | 17.57012001 |
| CREB3L1 | COL1A2 | 15.71487773 |
| CREB3L1 | CALU | 15.48516726 |
| CREB3L1 | SRM | 15.19177691 |
| CREB3L1 | P4HB | 15.16106938 |
| CREB3L1 | TUBA1A | 14.37371236 |
| CREB3L1 | SEC31A | 13.99808398 |
| CREB3L1 | PRDX4 | 13.38408591 |
| CREB3L1 | CCND1 | 12.95306789 |
| CREB3L1 | COPZ2 | 12.72207352 |
| CREB3L1 | DPT | 12.50296114 |
| CREB3L1 | TPM1 | 11.93624007 |
| CREB3L1 | MORF4L2 | 11.5933478 |
| CREB3L1 | KDELR2 | 11.15573107 |
| CREB3L1 | ASPN | 11.08654432 |
| CREB3L1 | MAGED1 | 11.08215833 |
| CREB3L1 | TPI1 | 10.7247913 |
| CREB3L1 | PPP1R14A | 10.55686871 |
| CREB3L1 | SERPINH1 | 9.949043191 |
| CREB3L1 | MFAP5 | 9.911681088 |
| CREB3L1 | NME4 | 9.810814829 |
| CREB3L1 | COL5A2 | 9.081276245 |
| CREB3L1 | CERCAM | 9.07638495 |
| CREB3L1 | GPX8 | 8.799720778 |
| CREB3L1 | SSR2 | 8.751085376 |
| CREB3L1 | TMEM119 | 8.66566707 |
| CREB3L1 | YWHAH | 8.499186779 |
| CREB3L1 | PYCR1 | 8.379309083 |
| CREB3L1 | HTRA1 | 8.367822267 |
| CREB3L1 | MARCKS | 8.216012419 |
| CREB3L1 | SF3B2 | 8.188595555 |
| CREB3L1 | TNMD | 8.115559396 |
| CREB3L1 | ZNHIT1 | 7.822013945 |
| CREB3L1 | MAGED2 | 7.730592163 |
| CREB3L1 | AP2M1 | 7.530572317 |
| CREB3L1 | RCN3 | 7.434488852 |
| CREB3L1 | HSPA5 | 7.355693933 |
| CREB3L1 | ERI3 | 7.316221567 |
| CREB3L1 | EMID1 | 7.307442845 |
| CREB3L1 | TTC3 | 7.274391447 |
| CREB3L1 | C1QTNF6 | 7.145700446 |
| CREB3L1 | TMED10 | 7.144680676 |
| CREB3L1 | MEF2C | 7.134528737 |
| CREB3L1 | ANGPTL2 | 7.091299733 |
| CREB3L1 | TUBB | 7.079520511 |
| CREB3L1 | FKBP7 | 7.037847068 |
| CREB3L1 | ANAPC5 | 6.958580969 |
| CREB3L1 | CDK4 | 6.957747547 |
| CREB3L1 | FSTL1 | 6.94430976 |
| CREB3L1 | KDELR1 | 6.754365008 |
| CREB3L1 | COL6A2 | 6.549146604 |
| CREB3L1 | CHPF | 6.541912013 |
| CREB3L1 | EIF4G2 | 6.535071586 |
| CREB3L1 | SLC39A7 | 6.35440941 |
| CREB3L1 | ARF4 | 6.280419243 |
| CREB3L1 | SDF2 | 6.259312017 |
| CREB3L1 | DAP | 6.235248212 |
| CREB3L1 | LMAN1 | 6.215499061 |
| CREB3L1 | CFL1 | 6.19558017 |
| CREB3L1 | CD320 | 6.157415582 |
| CREB3L1 | TMED2 | 6.148137184 |
| CREB3L1 | SEC61A1 | 6.071983288 |
| CREB3L1 | RABAC1 | 5.954426234 |
| CREB3L1 | UFC1 | 5.88473192 |
| CREB3L1 | ANXA6 | 5.875888164 |
| CREB3L1 | COL12A1 | 5.601250582 |
| CREB3L1 | RCN1 | 5.387773906 |
| CREB3L1 | SEC13 | 5.305025332 |
| CREB3L1 | PRELP | 5.304388501 |
| CREB3L1 | LGALS1 | 5.303616172 |
| CREB3L1 | LOXL2 | 5.27404784 |
| CREB3L1 | PHPT1 | 5.249032049 |
| CREB3L1 | RBM3 | 5.229989448 |
| CREB3L1 | OSTC | 5.193391238 |
| CREB3L1 | MXRA8 | 5.163281837 |
| CREB3L1 | SH3BGRL3 | 5.156688887 |
| CREB3L1 | CPXM1 | 5.154035413 |
| CREB3L1 | SPATS2L | 5.147689248 |
| CREB3L1 | SORBS3 | 5.053427195 |
| CREB3L1 | UBE2A | 5.024996513 |
| CREB3L1 | FKBP11 | 5.020907042 |
| CREB3L1 | MFAP2 | 4.874677797 |
| CREB3L1 | PAIP2 | 4.87378294 |
| CREB3L1 | AP3S1 | 4.870297856 |
| CREB3L1 | GNAI2 | 4.854576265 |
| CREB3L1 | HSP90B1 | 4.744522613 |
| CREB3L1 | PPIB | 4.685636877 |
| CREB3L1 | CNN3 | 4.6493162 |
| CREB3L1 | UROS | 4.608681907 |
| CREB3L1 | CALR | 4.608282796 |
| CREB3L1 | FHL1 | 4.560642492 |
| CREB3L1 | FAM114A1 | 4.543198607 |
| CREB3L1 | SCMH1 | 4.510165802 |
| CREB3L1 | DPEP1 | 4.367073073 |
| CREB3L1 | EFEMP2 | 4.342202865 |
| CREB3L1 | TMEM9 | 4.307584392 |
| CREB3L1 | ERLEC1 | 4.219105142 |
| CREB3L1 | MTDH | 4.214865706 |
| CREB3L1 | SEC61G | 4.21208484 |
| CREB3L1 | PPIC | 4.165217345 |
| CREB3L1 | TCF4 | 4.165151333 |
| CREB3L1 | NDUFB2 | 4.157514499 |
| CREB3L1 | KIFAP3 | 4.128979466 |
| CREB3L1 | PDIA6 | 4.128316209 |
| CREB3L1 | TNN | 4.128125567 |
| CREB3L1 | APPL1 | 4.111154034 |
| CREB3L1 | SEC23A | 4.067643266 |
| CREB3L1 | EDIL3 | 4.012921258 |
| CREB3L1 | RBBP7 | 4.010691242 |
| CREB3L1 | VKORC1 | 4.001382066 |
| CREB3L1 | COL14A1 | 3.800429543 |
| CREB3L1 | CTHRC1 | 3.797370406 |
| CREB3L1 | TUBA1B | 3.746802169 |
| CREB3L1 | SSR4 | 3.681908065 |
| CREB3L1 | RAD23B | 3.668124285 |
| CREB3L1 | ARHGDIA | 3.64695305 |
| CREB3L1 | MFGE8 | 3.626058093 |
| CREB3L1 | COL5A1 | 3.624883334 |
| CREB3L1 | MT2A | 3.620388978 |
| CREB3L1 | TSPO | 3.615927854 |
| CREB3L1 | GOLM1 | 3.572769584 |
| CREB3L1 | ANAPC11 | 3.538390595 |
| CREB3L1 | SH3GL1 | 3.498756426 |
| CREB3L1 | CD55 | 3.482742667 |
| CREB3L1 | NDUFA4 | 3.48145925 |
| CREB3L1 | MLEC | 3.402520183 |
| CREB3L1 | DCTN3 | 3.396873843 |
| CREB3L1 | MFAP4 | 3.391548244 |
| CREB3L1 | ISOC2 | 3.348123834 |
| CREB3L1 | COX8A | 3.34309607 |
| CREB3L1 | SSR1 | 3.302007385 |
| CREB3L1 | PMEPA1 | 3.26755441 |
| CREB3L1 | ADAMTS12 | 3.177657341 |
| CREB3L1 | CUTA | 3.174260045 |
| CREB3L1 | MZT2A | 3.165376587 |
| CREB3L1 | PGRMC1 | 3.079679503 |
| CREB3L1 | SURF4 | 3.023850722 |
| CREB3L1 | DCD | 3.012839201 |
| CREB3L1 | GPX7 | 3.002530624 |
| CREB3L1 | EXT2 | 2.963860581 |
| CREB3L1 | PSMA1 | 2.944298074 |
| CREB3L1 | APOD | 2.936352387 |
| CREB3L1 | H1F0 | 2.935380507 |
| CREB3L1 | AP2S1 | 2.922776072 |
| CREB3L1 | ARPC5 | 2.900504008 |
| CREB3L1 | IDH2 | 2.874890305 |
| CREB3L1 | ST3GAL4 | 2.851093121 |
| CREB3L1 | CLIC4 | 2.830142745 |
| CREB3L1 | RRAD | 2.797833171 |
| CREB3L1 | TMED9 | 2.781927091 |
| CREB3L1 | WIPF1 | 2.780916922 |
| CREB3L1 | IDH3B | 2.763793428 |
| CREB3L1 | AES | 2.744888077 |
| CREB3L1 | OLFML3 | 2.740255437 |
| CREB3L1 | POSTN | 2.689300383 |
| CREB3L1 | PIP | 2.635186252 |
| CREB3L1 | YIF1A | 2.615287457 |
| CREB3L1 | PFN1 | 2.614663716 |
| CREB3L1 | PSAP | 2.605995628 |
| CREB3L1 | GPC3 | 2.6040911 |
| CREB3L1 | KRT1 | 2.603298434 |
| CREB3L1 | ANO10 | 2.601102386 |
| CREB3L1 | SSR3 | 2.57540373 |
| CREB3L1 | COX6C | 2.56152126 |
| CREB3L1 | LDHB | 2.560189101 |
| CREB3L1 | EMX2 | 2.541179465 |
| CREB3L1 | RUNX2 | 2.530137667 |
| CREB3L1 | LZIC | 2.512106062 |
| CREB3L1 | FKBP9 | 2.503645459 |
| CREB3L1 | PNKD | 2.494190515 |
| CREB3L1 | SLC35E1 | 2.482773857 |
| CREB3L1 | RSL1D1 | 2.475103493 |
| CREB3L1 | EIF5A | 2.472205063 |
| CREB3L1 | SH3GLB1 | 2.459056422 |
| CREB3L1 | RPN1 | 2.445891849 |
| CREB3L1 | FKBP10 | 2.439915622 |
| CREB3L1 | SERP1 | 2.423058682 |
| CREB3L1 | HMGN2 | 2.39587073 |
| CREB3L1 | GLT8D1 | 2.387010416 |
| CREB3L1 | MYL9 | 2.384039859 |
| CREB3L1 | ATP6AP2 | 2.381359616 |
| CREB3L1 | ADD1 | 2.348047557 |
| CREB3L1 | MME | 2.327155849 |
| CREB3L1 | HDLBP | 2.323054821 |
| CREB3L1 | CD248 | 2.263827659 |
| CREB3L1 | GLUD1 | 2.256625887 |
| CREB3L1 | CAP1 | 2.229374302 |
| CREB3L1 | SEC63 | 2.228061499 |
| CREB3L1 | BASP1 | 2.173808052 |
| CREB3L1 | EIF4EBP1 | 2.173806414 |
| CREB3L1 | NPM1 | 2.167572268 |
| CREB3L1 | PTGES2 | 2.158009606 |
| CREB3L1 | LOXL1 | 2.153507164 |
| CREB3L1 | MYL12A | 2.145668336 |
| CREB3L1 | RPN2 | 2.134452657 |
| CREB3L1 | AGPAT3 | 2.133976021 |
| CREB3L1 | OPTN | 2.119758841 |
| CREB3L1 | GORASP2 | 2.118289039 |
| CREB3L1 | LMAN2 | 2.10996136 |
| CREB3L1 | KDELR3 | 2.105259859 |
| CREB3L1 | YEATS4 | 2.083057381 |
| CREB3L1 | C19orf70 | 2.061158385 |
| CREB3L1 | YIPF3 | 2.056751425 |
| CREB3L1 | UQCRC1 | 2.055705395 |
| CREB3L1 | UBE2E1 | 2.023841322 |
| CREB3L1 | PPP1R14B | 1.990590726 |
| CREB3L1 | BANF1 | 1.972592044 |
| CREB3L1 | SSRP1 | 1.95108562 |
| CREB3L1 | HNRNPM | 1.928899412 |
| CREB3L1 | LRRC17 | 1.910452742 |
| CREB3L1 | PSMB5 | 1.906534591 |
| CREB3L1 | SPAG16 | 1.895623556 |
| CREB3L1 | LAMP1 | 1.88944741 |
| CREB3L1 | CTGF | 1.881921918 |
| CREB3L1 | TMED3 | 1.874491401 |
| CREB3L1 | PDPN | 1.871084818 |
| CREB3L1 | PHB | 1.865039059 |
| CREB3L1 | ARL1 | 1.842745859 |
| CREB3L1 | CTSZ | 1.831592293 |
| CREB3L1 | BUD31 | 1.805126398 |
| CREB3L1 | YBX1 | 1.801281122 |
| CREB3L1 | BSG | 1.790508246 |
| CREB3L1 | PAFAH1B3 | 1.783399293 |
| CREB3L1 | STMN1 | 1.774356818 |
| CREB3L1 | FN1 | 1.769079793 |
| CREB3L1 | PRRX1 | 1.746405706 |
| CREB3L1 | SAR1A | 1.718489673 |
| CREB3L1 | SMC4 | 1.716965321 |
| CREB3L1 | SFXN3 | 1.711933022 |
| CREB3L1 | HNRNPA3 | 1.711851299 |
| CREB3L1 | PCBP1 | 1.694604334 |
| CREB3L1 | TMEM160 | 1.692248727 |
| CREB3L1 | ACP1 | 1.691436959 |
| CREB3L1 | RIOK2 | 1.649164641 |
| CREB3L1 | RFX1 | 1.623553001 |
| CREB3L1 | LY6D | 1.614087208 |
| CREB3L1 | ZNF439 | 1.596521992 |
| CREB3L1 | ERCC1 | 1.596293289 |
| CREB3L1 | CCDC88A | 1.585853256 |
| CREB3L1 | SUPT5H | 1.577171214 |
| CREB3L1 | PALLD | 1.574068698 |
| CREB3L1 | ZYX | 1.568983821 |
| CREB3L1 | LMNA | 1.568155643 |
| CREB3L1 | WFDC1 | 1.538202619 |
| CREB3L1 | COPB1 | 1.529669318 |
| CREB3L1 | FAM124A | 1.512633668 |
| CREB3L1 | EZR | 1.509646581 |
| CREB3L1 | SERBP1 | 1.505599212 |
| CREB3L1 | ARHGAP11A | 1.489334242 |
| CREB3L1 | ROBO2 | 1.484568433 |
| CREB3L1 | DGUOK | 1.464908071 |
| CREB3L1 | SNX8 | 1.439793559 |
| CREB3L1 | PSMB7 | 1.433532107 |
| CREB3L1 | OS9 | 1.433531033 |
| CREB3L1 | HOXC8 | 1.429801406 |
| CREB3L1 | ACTG1 | 1.42854448 |
| CREB3L1 | AURKAIP1 | 1.427417668 |
| CREB3L1 | DHDDS | 1.425752673 |
| CREB3L1 | UQCRQ | 1.421178853 |
| CREB3L1 | SLC39A14 | 1.408078803 |
| CREB3L1 | MXRA5 | 1.405490176 |
| CREB3L1 | RAB2A | 1.39949706 |
| CREB3L1 | NDUFB11 | 1.393998587 |
| CREB3L1 | STIP1 | 1.382941901 |
| CREB3L1 | MXD4 | 1.367941384 |
| CREB3L1 | POLR2J | 1.352897544 |
| CREB3L1 | TXNL4A | 1.35153702 |
| CREB3L1 | IFI27 | 1.318718819 |
| CREB3L1 | SNX2 | 1.308753288 |
| CREB3L1 | ITGB1 | 1.305571269 |
| CREB3L1 | AP1M1 | 1.299641353 |
| CREB3L1 | CHCHD10 | 1.298312881 |
| CREB3L1 | CDC42EP5 | 1.28095102 |
| CREB3L1 | MARVELD1 | 1.273308242 |
| CREB3L1 | BIRC5 | 1.266225767 |
| CREB3L1 | FUS | 1.264396659 |
| CREB3L1 | PPP2R1A | 1.258806143 |
| CREB3L1 | ADCK5 | 1.25612028 |
| CREB3L1 | PDE2A | 1.255310518 |
| CREB3L1 | COL16A1 | 1.254301362 |
| CREB3L1 | MGST3 | 1.250103354 |
| CREB3L1 | MRPL51 | 1.246660444 |
| CREB3L1 | TXNIP | 1.240541128 |
| CREB3L1 | SET | 1.238212245 |
| CREB3L1 | PPP2R5A | 1.235135092 |
| CREB3L1 | TMEM158 | 1.227413983 |
| CREB3L1 | CRTAP | 1.22425134 |
| CREB3L1 | PCBP2 | 1.220164422 |
| CREB3L1 | PTMS | 1.219212767 |
| CREB3L1 | RHOC | 1.215674511 |
| CREB3L1 | ESCO1 | 1.203266574 |
| CREB3L1 | MRPL3 | 1.20120147 |
| CREB3L1 | RNF181 | 1.195011044 |
| CREB3L1 | TNFRSF12A | 1.189777509 |
| CREB3L1 | SERPINB7 | 1.179906636 |
| CREB3L1 | DMKN | 1.175726427 |
| CREB3L1 | TPM2 | 1.16973778 |
| CREB3L1 | PTOV1 | 1.167245639 |
| CREB3L1 | DDX11 | 1.166085652 |
| CREB3L1 | CLCN4 | 1.166019561 |
| CREB3L1 | ADAMTS2 | 1.159715364 |
| CREB3L1 | MRPL23 | 1.1576357 |
| CREB3L1 | ZBTB7C | 1.153981266 |
| CREB3L1 | IDH3G | 1.153483735 |
| CREB3L1 | S100A10 | 1.152559123 |
| CREB3L1 | CTSK | 1.147253891 |
| CREB3L1 | TTC1 | 1.145786719 |
| CREB3L1 | SNRPC | 1.141653577 |
| CREB3L1 | LMF2 | 1.137875541 |
| CREB3L1 | SOX4 | 1.137029527 |
| CREB3L1 | TMIE | 1.135747381 |
| CREB3L1 | LAMB1 | 1.128282697 |
| CREB3L1 | NBN | 1.122957851 |
| CREB3L1 | TPM4 | 1.108392336 |
| CREB3L1 | SUV39H1 | 1.101484411 |
| CREB3L1 | CALD1 | 1.089627067 |
| CREB3L1 | MYL12B | 1.089257743 |
| CREB3L1 | VCAN | 1.088039282 |
| CREB3L1 | ATOX1 | 1.087372141 |
| CREB3L1 | ADAM12 | 1.086292906 |
| CREB3L1 | TCF25 | 1.085572029 |
| CREB3L1 | STOML2 | 1.081318768 |
| CREB3L1 | ACTR2 | 1.078598095 |
| CREB3L1 | DSG1 | 1.069996725 |
| CREB3L1 | GLT8D2 | 1.068326042 |
| CREB3L1 | BICD2 | 1.066508136 |
| CREB3L1 | WDR45 | 1.065178157 |
| CREB3L1 | SDF4 | 1.062995492 |
| CREB3L1 | PSMC3 | 1.062889793 |
| CREB3L1 | DNAJC3 | 1.062629752 |
| CREB3L1 | SAMD4B | 1.061573744 |
| CREB3L1 | P4HA1 | 1.061550917 |
| CREB3L1 | SNCA | 1.048441393 |
| CREB3L1 | LGALS7B | 1.041606512 |
| CREB3L1 | KRT5 | 1.035977074 |
| CREB3L1 | DUSP14 | 1.023219366 |
| CREB3L1 | HEXA | 1.020782814 |
| CREB3L1 | TXLNA | 1.014645984 |
| CREB3L1 | INHBA | 1.013854819 |
| CREB3L1 | FSCN1 | 1.012977592 |
| CREB3L1 | RHOB | 1.004402803 |
| CREB3L1 | USP54 | 1.003193816 |
| CREB3L1 | CREB3L1 | 1 |
| CREB3L1 | CDCA5 | 0.999862381 |
| CREB3L1 | ANTXR1 | 0.995944114 |
| CREB3L1 | HLA-C | 0.986901669 |
| CREB3L1 | MRC2 | 0.985514615 |
| CREB3L1 | KRTDAP | 0.983119422 |
| CREB3L1 | EXOSC10 | 0.975967156 |
| CREB3L1 | NCDN | 0.975842969 |
| CREB3L1 | SCAND1 | 0.975689664 |
| CREB3L1 | MGLL | 0.968781163 |
| CREB3L1 | LOX | 0.966365104 |
| CREB3L1 | TPM3 | 0.955298538 |
| CREB3L1 | PDIA5 | 0.952187583 |
| CREB3L1 | RAB31 | 0.950740168 |
| CREB3L1 | TUBG2 | 0.947966416 |
| CREB3L1 | NDUFA1 | 0.946979258 |
| CREB3L1 | ANXA3 | 0.945522919 |
| CREB3L1 | TCEAL4 | 0.941428372 |
| CREB3L1 | PHF1 | 0.941195985 |
| CREB3L1 | CAPZB | 0.926447849 |
| CREB3L1 | PARVA | 0.923342724 |
| CREB3L1 | EMD | 0.913978906 |
| CREB3L1 | RAB1A | 0.909906632 |
| CREB3L1 | PTGER3 | 0.908559237 |
| CREB3L1 | UCHL1 | 0.908110341 |
| CREB3L1 | DCTD | 0.908106614 |
| CREB3L1 | PRKCSH | 0.902162742 |
| CREB3L1 | RHBDD3 | 0.897910347 |
| CREB3L1 | PSMB3 | 0.895118808 |
| CREB3L1 | COX6A1 | 0.893108234 |
| CREB3L1 | USP11 | 0.890584514 |
| CREB3L1 | KPNA2 | 0.884279366 |
| CREB3L1 | FAM3C | 0.880852742 |
| CREB3L1 | NAPA | 0.87637847 |
| CREB3L1 | GJA1 | 0.872646088 |
| CREB3L1 | LGALS7 | 0.869646741 |
| CREB3L1 | GAS6 | 0.85253698 |
| CREB3L1 | ZCCHC17 | 0.8516503 |
| CREB3L1 | ITGA5 | 0.851366814 |
| CREB3L1 | SNX33 | 0.851310787 |
| CREB3L1 | SFN | 0.849287223 |
| CREB3L1 | VPS35 | 0.841849516 |
| CREB3L1 | PRUNE2 | 0.838276184 |
| CREB3L1 | CDC37 | 0.837674686 |
| CREB3L1 | SLC26A2 | 0.832531624 |
| CREB3L1 | UBE2L6 | 0.82786301 |
| CREB3L1 | ANXA5 | 0.826858465 |
| CREB3L1 | PAWR | 0.826159989 |
| CREB3L1 | RPL23A | 0.819840387 |
| CREB3L1 | UBA52 | 0.803558112 |
| CREB3L1 | DAB2 | 0.792368538 |
| CREB3L1 | SLC17A5 | 0.790924667 |
| CREB3L1 | LMNB1 | 0.790623461 |
| CREB3L1 | EIF3F | 0.78761636 |
| CREB3L1 | STT3A | 0.786772132 |
| CREB3L1 | NID2 | 0.785693077 |
| CREB3L1 | SF3B1 | 0.782935684 |
| CREB3L1 | PGK1 | 0.782797637 |
| CREB3L1 | GUK1 | 0.766239078 |
| CREB3L1 | NUDC | 0.758852959 |
| CREB3L1 | GOPC | 0.756771623 |
| CREB3L1 | HDGF | 0.756175556 |
| CREB3L1 | FNIP1 | 0.756005251 |
| CREB3L1 | HM13 | 0.751295836 |
| CREB3L1 | PIN1 | 0.751168243 |
| CREB3L1 | GPX2 | 0.741370442 |
| CREB3L1 | XBP1 | 0.737756967 |
| CREB3L1 | BANP | 0.736730247 |
| CREB3L1 | HNRNPA1 | 0.733456429 |
| CREB3L1 | GSDMB | 0.73171126 |
| CREB3L1 | CKAP4 | 0.720872821 |
| CREB3L1 | MORF4L1 | 0.718385378 |
| CREB3L1 | PSMD14 | 0.709168928 |
| CREB3L1 | UBE2Q2 | 0.708214557 |
| CREB3L1 | PAPSS1 | 0.707851488 |
| CREB3L1 | CRABP2 | 0.706441902 |
| CREB3L1 | CTAGE5 | 0.703566076 |
| CREB3L1 | NUPL2 | 0.700333365 |
| CREB3L1 | PRCP | 0.700106703 |
| CREB3L1 | JAGN1 | 0.69987119 |
| CREB3L1 | KIF2C | 0.699840162 |
| CREB3L1 | PRDX5 | 0.699628845 |
| CREB3L1 | SPON2 | 0.694483813 |
| CREB3L1 | GOLGA5 | 0.686560022 |
| CREB3L1 | DENND2D | 0.68349797 |
| CREB3L1 | ACBD3 | 0.676285335 |
| CREB3L1 | SCP2 | 0.674348435 |
| CREB3L1 | PNRC1 | 0.671158946 |
| CREB3L1 | PPP2R5C | 0.665330872 |
| CREB3L1 | PTP4A2 | 0.660725472 |
| CREB3L1 | SUCLG2 | 0.659900464 |
| CREB3L1 | KATNAL2 | 0.658721068 |
| CREB3L1 | NEGR1 | 0.656447023 |
| CREB3L1 | FBN1 | 0.6525861 |
| CREB3L1 | FBXL15 | 0.652198129 |
| CREB3L1 | C22orf31 | 0.65175254 |
| CREB3L1 | AHSA1 | 0.647991629 |
| CREB3L1 | LRP2BP | 0.644439239 |
| CREB3L1 | REST | 0.640606709 |
| CREB3L1 | PPP1R3E | 0.628247399 |
| CREB3L1 | CDH11 | 0.626707836 |
| CREB3L1 | GPN2 | 0.621599666 |
| CREB3L1 | SND1 | 0.619200692 |
| CREB3L1 | IMPDH1 | 0.619186555 |
| CREB3L1 | ERN1 | 0.612078619 |
| CREB3L1 | ABAT | 0.602031371 |
| CREB3L1 | COPS6 | 0.601303851 |
| CREB3L1 | GTF2A2 | 0.60105314 |
| CREB3L1 | RRP1 | 0.598275051 |
| CREB3L1 | NUCB2 | 0.59686832 |
| CREB3L1 | PCDHB7 | 0.586983338 |
| CREB3L1 | UQCRC2 | 0.586135179 |
| CREB3L1 | NFKBIZ | 0.57824015 |
| CREB3L1 | PTN | 0.575216028 |
| CREB3L1 | TMOD3 | 0.573190923 |
| CREB3L1 | DHRS7 | 0.572686255 |
| CREB3L1 | VPS26B | 0.572013977 |
| CREB3L1 | GINS4 | 0.570845318 |
| CREB3L1 | ELOVL5 | 0.563021476 |
| CREB3L1 | RBFOX1 | 0.5569048 |
| CREB3L1 | MPHOSPH6 | 0.555886031 |
| CREB3L1 | GRB2 | 0.55573737 |
| CREB3L1 | COX5A | 0.55328715 |
| CREB3L1 | TMEM143 | 0.55044686 |
| CREB3L1 | LRRC59 | 0.543352885 |
| CREB3L1 | EPHA2 | 0.542322939 |
| CREB3L1 | YWHAG | 0.535542245 |
| CREB3L1 | POLDIP2 | 0.535135491 |
| CREB3L1 | RANBP1 | 0.534332661 |
| CREB3L1 | ZDHHC18 | 0.533408629 |
| CREB3L1 | B3GNTL1 | 0.533370991 |
| CREB3L1 | DBN1 | 0.533178101 |
| CREB3L1 | ADPRHL1 | 0.532887902 |
| CREB3L1 | CTCF | 0.532733186 |
| CREB3L1 | SLC2A6 | 0.532321237 |
| CREB3L1 | TMEM201 | 0.53099118 |
| CREB3L1 | RDH11 | 0.530442494 |
| CREB3L1 | LMO4 | 0.530350777 |
| CREB3L1 | CITED2 | 0.529269506 |
| CREB3L1 | MMP3 | 0.525608178 |
| CREB3L1 | PRDX3 | 0.520167865 |
| CREB3L1 | P4HA2 | 0.51993125 |
| CREB3L1 | HSBP1 | 0.519731003 |
| CREB3L1 | LYPD6B | 0.518026936 |
| CREB3L1 | STX17 | 0.517237027 |
| CREB3L1 | TMTC2 | 0.514418526 |
| CREB3L1 | FNDC3B | 0.513461716 |
| CREB3L1 | SRRM1 | 0.512453279 |
| CREB3L1 | PRELID2 | 0.511677522 |
| CREB3L1 | ARL2 | 0.507782301 |
| CREB3L1 | UBE2E3 | 0.506596283 |
| CREB3L1 | GK | 0.506004777 |
| CREB3L1 | PERP | 0.502575597 |
| CREB3L1 | NHLRC1 | 0.502193212 |
| CREB3L1 | DCBLD2 | 0.501480446 |
| CREB3L1 | DDA1 | 0.495655172 |
| CREB3L1 | ZFAND6 | 0.494433897 |
| CREB3L1 | KLF8 | 0.492373831 |
| CREB3L1 | AACS | 0.489677753 |
| CREB3L1 | AKR1A1 | 0.487758605 |
| CREB3L1 | SLC35F5 | 0.485741036 |
| CREB3L1 | GNAO1 | 0.485167007 |
| CREB3L1 | TMEM167B | 0.484610743 |
| CREB3L1 | LIPA | 0.482344296 |
| CREB3L1 | KDM5A | 0.482080778 |
| CREB3L1 | SENP3 | 0.481792837 |
| CREB3L1 | CDKN2A | 0.480193992 |
| CREB3L1 | USP49 | 0.478634001 |
| CREB3L1 | CALML5 | 0.478261099 |
| CREB3L1 | ANAPC4 | 0.475290283 |
| CREB3L1 | SCFD1 | 0.473007996 |
| CREB3L1 | SNRPA | 0.471510563 |
| CREB3L1 | SPATA7 | 0.466557955 |
| CREB3L1 | PFKFB2 | 0.465046723 |
| CREB3L1 | BMP1 | 0.464384108 |
| CREB3L1 | APCDD1 | 0.46266919 |
| CREB3L1 | STX18 | 0.460636493 |
| CREB3L1 | GPRC5C | 0.460085578 |
| CREB3L1 | RAN | 0.459694397 |
| CREB3L1 | MYH9 | 0.456483889 |
| CREB3L1 | UNK | 0.45592699 |
| CREB3L1 | DNAJB11 | 0.452190575 |
| CREB3L1 | CA13 | 0.451569108 |
| CREB3L1 | WNK2 | 0.450643993 |
| CREB3L1 | ACTR1A | 0.450228559 |
| CREB3L1 | RAD23A | 0.449420593 |
| CREB3L1 | EFEMP1 | 0.449419236 |
| CREB3L1 | ARCN1 | 0.446388599 |
| CREB3L1 | MAP1A | 0.44598372 |
| CREB3L1 | POLR2H | 0.444761881 |
| CREB3L1 | PLD3 | 0.443491248 |
| CREB3L1 | MLF2 | 0.442240357 |
| CREB3L1 | P2RX7 | 0.440957592 |
| CREB3L1 | REXO1 | 0.440791329 |
| CREB3L1 | DPM2 | 0.440649149 |
| CREB3L1 | CTBP2 | 0.439843928 |
| CREB3L1 | RSU1 | 0.437902684 |
| CREB3L1 | CHID1 | 0.435468566 |
| CREB3L1 | PRIM2 | 0.435091411 |
| CREB3L1 | MTHFSD | 0.434441075 |
| CREB3L1 | ZNF367 | 0.431813281 |
| CREB3L1 | SMTN | 0.429975602 |
| CREB3L1 | NRAS | 0.426662324 |
| CREB3L1 | PSMF1 | 0.424745948 |
| CREB3L1 | ITPR2 | 0.422686833 |
| CREB3L1 | ZNF670 | 0.422195075 |
| CREB3L1 | GRP | 0.421535175 |
| CREB3L1 | QSOX1 | 0.417701545 |
| CREB3L1 | CDT1 | 0.417661882 |
| CREB3L1 | FDXR | 0.416515708 |
| CREB3L1 | RALGPS2 | 0.415303135 |
| CREB3L1 | RAD54L2 | 0.411500629 |
| CREB3L1 | RAB10 | 0.409218014 |
| CREB3L1 | PREB | 0.408672283 |
| CREB3L1 | RIPPLY2 | 0.406401935 |
| CREB3L1 | GMDS | 0.401535443 |
| CREB3L1 | OPCML | 0.400091101 |
| CREB3L1 | SHC3 | 0.399475927 |
| CREB3L1 | CHAC2 | 0.389636926 |
| CREB3L1 | GANAB | 0.386537534 |
| CREB3L1 | FAM20C | 0.379459246 |
| CREB3L1 | E4F1 | 0.37940336 |
| CREB3L1 | VMA21 | 0.377878278 |
| CREB3L1 | SFTA1P | 0.377462848 |
| CREB3L1 | APEX1 | 0.376221334 |
| CREB3L1 | NEIL2 | 0.372625641 |
| CREB3L1 | LMO7 | 0.370380107 |
| CREB3L1 | CORO7 | 0.369877263 |
| CREB3L1 | KSR1 | 0.368860012 |
| CREB3L1 | LGR6 | 0.367001854 |
| CREB3L1 | PPP4R1 | 0.363557433 |
| CREB3L1 | MAOB | 0.363260615 |
| CREB3L1 | SMARCA2 | 0.362268508 |
| CREB3L1 | FOXC1 | 0.358400322 |
| CREB3L1 | NEK6 | 0.358201949 |
| CREB3L1 | FAM160A2 | 0.357586401 |
| CREB3L1 | STK10 | 0.35560821 |
| CREB3L1 | TBC1D10A | 0.351987666 |
| CREB3L1 | GCNT1 | 0.351142759 |
| CREB3L1 | MAPKBP1 | 0.350202175 |
| CREB3L1 | GPLD1 | 0.349723405 |
| CREB3L1 | VWA5A | 0.349136418 |
| CREB3L1 | JDP2 | 0.347262832 |
| CREB3L1 | PFAS | 0.346603838 |
| CREB3L1 | ZNF83 | 0.345952311 |
| CREB3L1 | KCNA5 | 0.338400173 |
| CREB3L1 | MAP3K1 | 0.332246239 |
| CREB3L1 | F7 | 0.332051494 |
| CREB3L1 | NPL | 0.329234638 |
| CREB3L1 | ARFGEF1 | 0.328705739 |
| CREB3L1 | ROPN1B | 0.328074865 |
| CREB3L1 | OXNAD1 | 0.325919775 |
| CREB3L1 | CCDC51 | 0.32557863 |
| CREB3L1 | GRIK2 | 0.324746679 |
| CREB3L1 | NT5DC2 | 0.321964104 |
| CREB3L1 | ZRANB3 | 0.320560692 |
| CREB3L1 | SLC29A4 | 0.32001668 |
| CREB3L1 | COQ10A | 0.31787617 |
| CREB3L1 | CDC16 | 0.316614308 |
| CREB3L1 | TSG101 | 0.316527659 |
| CREB3L1 | EXTL1 | 0.316503374 |
| CREB3L1 | SECISBP2L | 0.313245626 |
| CREB3L1 | GMFB | 0.31308739 |
| CREB3L1 | ERBB3 | 0.312397681 |
| CREB3L1 | GBF1 | 0.311758824 |
| CREB3L1 | BUB3 | 0.30893459 |
| CREB3L1 | ZFPL1 | 0.307729757 |
| CREB3L1 | CASP8AP2 | 0.307530365 |
| CREB3L1 | MACROD1 | 0.305596113 |
| CREB3L1 | NCKIPSD | 0.303213533 |
| CREB3L1 | NDRG4 | 0.302392042 |
| CREB3L1 | PCIF1 | 0.301973682 |
| CREB3L1 | WDR77 | 0.300925456 |
| CREB3L1 | ARHGEF9 | 0.300874792 |
| CREB3L1 | LRRC26 | 0.299231372 |
| CREB3L1 | RGL1 | 0.298582587 |
| CREB3L1 | SREK1IP1 | 0.297971695 |
| CREB3L1 | COPA | 0.297652737 |
| CREB3L1 | GLI2 | 0.297354722 |
| CREB3L1 | ZNF543 | 0.297164951 |
| CREB3L1 | ZNF876P | 0.296814239 |
| CREB3L1 | BPTF | 0.295657263 |
| CREB3L1 | PODXL2 | 0.294247862 |
| CREB3L1 | RBL1 | 0.290737102 |
| CREB3L1 | FBXL13 | 0.289260158 |
| CREB3L1 | NXPH3 | 0.286894042 |
| CREB3L1 | SCRN1 | 0.285602142 |
| CREB3L1 | GRPEL1 | 0.28537181 |
| CREB3L1 | GPBAR1 | 0.283912799 |
| CREB3L1 | RBM4B | 0.283422976 |
| CREB3L1 | HMGN4 | 0.281825941 |
| CREB3L1 | PRICKLE3 | 0.278668501 |
| CREB3L1 | PCMT1 | 0.277499102 |
| CREB3L1 | DLST | 0.277368041 |
| CREB3L1 | VPS41 | 0.277142904 |
| CREB3L1 | C14orf28 | 0.276737651 |
| CREB3L1 | SSBP3 | 0.275924828 |
| CREB3L1 | IGF2R | 0.274926178 |
| CREB3L1 | ROCK2 | 0.273399136 |
| CREB3L1 | C17orf51 | 0.267713267 |
| CREB3L1 | GRIPAP1 | 0.265497254 |
| CREB3L1 | TRIM26 | 0.264561195 |
| CREB3L1 | RPS6KB2 | 0.264230667 |
| CREB3L1 | LEPROTL1 | 0.263579839 |
| CREB3L1 | CDC42BPA | 0.263233919 |
| CREB3L1 | NANS | 0.263098399 |
| CREB3L1 | PTDSS1 | 0.260856473 |
| CREB3L1 | IPP | 0.260133413 |
| CREB3L1 | KIAA0355 | 0.258845868 |
| CREB3L1 | RGPD3 | 0.257258291 |
| CREB3L1 | IL20RB | 0.256628215 |
| CREB3L1 | NIPSNAP3A | 0.256365071 |
| CREB3L1 | ARMC1 | 0.254628289 |
| CREB3L1 | BAHD1 | 0.254327377 |
| CREB3L1 | ZMYM6 | 0.253683236 |
| CREB3L1 | PLOD1 | 0.252539435 |
| CREB3L1 | SRP54 | 0.249670513 |
| CREB3L1 | BMPR1A | 0.249403551 |
| CREB3L1 | POLR3D | 0.249053564 |
| CREB3L1 | TCHH | 0.248355114 |
| CREB3L1 | DHX32 | 0.248005144 |
| CREB3L1 | STX12 | 0.247591252 |
| CREB3L1 | ABCG2 | 0.247320595 |
| CREB3L1 | RARG | 0.246887798 |
| CREB3L1 | TSPAN13 | 0.246179395 |
| CREB3L1 | PXDN | 0.246101103 |
| CREB3L1 | ABCC4 | 0.243366782 |
| CREB3L1 | L3MBTL3 | 0.241702396 |
| CREB3L1 | TRAIP | 0.237675844 |
| CREB3L1 | DMXL1 | 0.237159909 |
| CREB3L1 | PTBP1 | 0.237103148 |
| CREB3L1 | ABCF1 | 0.237039436 |
| CREB3L1 | AP1S1 | 0.236317362 |
| CREB3L1 | PKP4 | 0.235281553 |
| CREB3L1 | TRIP12 | 0.235047828 |
| CREB3L1 | MDN1 | 0.234881668 |
| CREB3L1 | HOXB5 | 0.234416746 |
| CREB3L1 | USP48 | 0.233700247 |
| CREB3L1 | THAP8 | 0.232766228 |
| CREB3L1 | FADS1 | 0.2325071 |
| CREB3L1 | ZNF264 | 0.230985132 |
| CREB3L1 | C15orf39 | 0.23093012 |
| CREB3L1 | TAF8 | 0.228224028 |
| CREB3L1 | PBRM1 | 0.226964771 |
| CREB3L1 | NGLY1 | 0.222759288 |
| CREB3L1 | STK16 | 0.222326966 |
| CREB3L1 | CILP | 0.22210653 |
| CREB3L1 | ATF7 | 0.221082527 |
| CREB3L1 | CEP135 | 0.218487985 |
| CREB3L1 | UHRF2 | 0.216563488 |
| CREB3L1 | PSMC6 | 0.216388311 |
| CREB3L1 | GXYLT2 | 0.215911969 |
| CREB3L1 | ATP2A2 | 0.215256532 |
| CREB3L1 | TMEM17 | 0.215117721 |
| CREB3L1 | HYAL2 | 0.213228195 |
| CREB3L1 | SOX11 | 0.212830052 |
| CREB3L1 | FAM50A | 0.2125709 |
| CREB3L1 | PDCD7 | 0.212555486 |
| CREB3L1 | CDK5 | 0.211786621 |
| CREB3L1 | TSKU | 0.209534366 |
| CREB3L1 | SLC5A6 | 0.20841804 |
| CREB3L1 | RTF1 | 0.207660347 |
| CREB3L1 | CDC42EP1 | 0.20709061 |
| CREB3L1 | AP4B1 | 0.205891016 |
| CREB3L1 | CRLS1 | 0.205043539 |
| CREB3L1 | UBXN6 | 0.203010615 |
| CREB3L1 | CANT1 | 0.202995567 |
| CREB3L1 | MTPAP | 0.201491087 |
| CREB3L1 | ICMT | 0.199667239 |
| CREB3L1 | ARL2BP | 0.199120575 |
| CREB3L1 | CPVL | 0.198036208 |
| CREB3L1 | SLC43A3 | 0.196447973 |
| CREB3L1 | PLP1 | 0.192335892 |
| CREB3L1 | RECQL4 | 0.190673625 |
| CREB3L1 | TLE3 | 0.189051768 |
| CREB3L1 | GART | 0.188278583 |
| CREB3L1 | SORD | 0.185759043 |
| CREB3L1 | ARSA | 0.1857239 |
| CREB3L1 | KLHDC3 | 0.185320757 |
| CREB3L1 | BIRC6 | 0.185309241 |
| CREB3L1 | THUMPD3 | 0.183103597 |
| CREB3L1 | RNF126 | 0.18250591 |
| CREB3L1 | RAMP2 | 0.181543848 |
| CREB3L1 | SEMA3B | 0.178948243 |
| CREB3L1 | BAZ1B | 0.178281181 |
| CREB3L1 | KIF7 | 0.177267805 |
| CREB3L1 | CAPN7 | 0.176686197 |
| CREB3L1 | ZNF461 | 0.17544859 |
| CREB3L1 | CD2AP | 0.175188193 |
| CREB3L1 | TTC39C | 0.174933748 |
| CREB3L1 | LAGE3 | 0.174875263 |
| CREB3L1 | PLCXD3 | 0.174487643 |
| CREB3L1 | TBC1D2 | 0.17354807 |
| CREB3L1 | CDK14 | 0.172569751 |
| CREB3L1 | FZD5 | 0.172361635 |
| CREB3L1 | ALS2 | 0.170883562 |
| CREB3L1 | MAN2B2 | 0.170329602 |
| CREB3L1 | EGFL6 | 0.169615542 |
| CREB3L1 | FMR1 | 0.167219325 |
| CREB3L1 | TELO2 | 0.166342367 |
| CREB3L1 | TMEM52 | 0.166298751 |
| CREB3L1 | SCYL1 | 0.165456885 |
| CREB3L1 | SNX18 | 0.164490076 |
| CREB3L1 | MRPS9 | 0.161293454 |
| CREB3L1 | SDC1 | 0.160202064 |
| CREB3L1 | TBL2 | 0.159588664 |
| CREB3L1 | GREM1 | 0.159071379 |
| CREB3L1 | GSK3B | 0.158833939 |
| CREB3L1 | SPG7 | 0.158644102 |
| CREB3L1 | TAOK2 | 0.156482617 |
| CREB3L1 | TEP1 | 0.153201488 |
| CREB3L1 | POLRMT | 0.150539329 |
| CREB3L1 | LARP1 | 0.149699158 |
| CREB3L1 | ZNF362 | 0.148348836 |
| CREB3L1 | THAP4 | 0.147131965 |
| CREB3L1 | CASP7 | 0.146554572 |
| CREB3L1 | TYRO3 | 0.145423568 |
| CREB3L1 | ZNF12 | 0.145366095 |
| CREB3L1 | ABHD10 | 0.14290235 |
| CREB3L1 | PCSK6 | 0.14137584 |
| CREB3L1 | SPHK1 | 0.140671963 |
| CREB3L1 | INTS4 | 0.139568288 |
| CREB3L1 | NUP153 | 0.135729292 |
| CREB3L1 | SSH1 | 0.135492865 |
| CREB3L1 | AXIN1 | 0.134881763 |
| CREB3L1 | ADSS | 0.13399473 |
| CREB3L1 | CRNDE | 0.131072797 |
| CREB3L1 | ESRP1 | 0.130159548 |
| CREB3L1 | TTBK2 | 0.129660735 |
| CREB3L1 | NIF3L1 | 0.12915333 |
| CREB3L1 | AMN1 | 0.127565861 |
| CREB3L1 | PLEKHA6 | 0.127263247 |
| CREB3L1 | ZNRD1 | 0.125926319 |
| CREB3L1 | SEC11C | 0.124571597 |
| CREB3L1 | AGBL3 | 0.124530543 |
| CREB3L1 | STAG3L3 | 0.123907883 |
| CREB3L1 | ARAP2 | 0.118953627 |
| CREB3L1 | DGKH | 0.115676808 |
| CREB3L1 | B9D1 | 0.114098849 |
| CREB3L1 | SMARCAL1 | 0.11328917 |
| CREB3L1 | ALKBH5 | 0.11049237 |
| CREB3L1 | HOMER2 | 0.109846189 |
| CREB3L1 | TMEM171 | 0.109537947 |
| CREB3L1 | ZNF35 | 0.108729306 |
| CREB3L1 | IFRD2 | 0.106652283 |
| CREB3L1 | LTC4S | 0.106391361 |
| CREB3L1 | NEFL | 0.103784691 |
| CREB3L1 | SPPL2B | 0.102810002 |
| CREB3L1 | ROPN1L | 0.102318993 |
| CREB3L1 | ITGA3 | 0.10189188 |
| CREB3L1 | OMA1 | 0.101627477 |
| CREB3L1 | GNA13 | 0.097414976 |
| CREB3L1 | SLC38A6 | 0.094097942 |
| CREB3L1 | KLRD1 | 0.09382479 |
| CREB3L1 | GPR180 | 0.090316224 |
| CREB3L1 | MYO1E | 0.090090519 |
| CREB3L1 | PRSS35 | 0.089137674 |
| CREB3L1 | CAMK4 | 0.086595079 |
| CREB3L1 | SLC39A9 | 0.083148737 |
| CREB3L1 | TRIM36 | 0.080828844 |
| CREB3L1 | MTHFD1 | 0.077475172 |
| CREB3L1 | AMPH | 0.075704032 |
| CREB3L1 | MAP2K1 | 0.073879449 |
| CREB3L1 | PRSS36 | 0.072831392 |
| CREB3L1 | RB1CC1 | 0.072426967 |
| CREB3L1 | LRRC37BP1 | 0.070147202 |
| CREB3L1 | TAF15 | 0.069713961 |
| CREB3L1 | HELQ | 0.069349092 |
| CREB3L1 | OXSR1 | 0.066903497 |
| CREB3L1 | ZBTB47 | 0.038130891 |
| CREB3L1 | PODXL | 0.035795721 |
| CREB3L1 | SLC6A4 | 0.033905446 |
| CREB3L1 | ZNF304 | 0.033123201 |
| CREB3L1 | NEURL2 | 0.027905424 |
| CREB3L1 | RXFP1 | 0.023961591 |
| CREB3L1 | TPSB2 | 0.020695172 |
| CREB3L1 | TRUB1 | 0.018942138 |
| CREB3L1 | SLC25A39 | 0.015281166 |
| CREB3L1 | USP15 | 0.014425415 |
| CREB3L1 | NOTCH2 | 0.014046217 |
| CREB3L1 | CCDC74B | 0.013072158 |
| CREB3L1 | PRKAB1 | 0.012591646 |
| CREB3L1 | STARD7 | 0.005541526 |
| CREB3L1 | USP9X | 0.005264863 |
| CREB3L1 | WWP2 | 0.001854736 |
| CREB3L1 | RBM18 | 0.001730834 |
| CREB3L1 | POP7 | 0.001704661 |
| CREB3L1 | WASF3 | 0.00067515 |
| CREB3L1 | AUTS2 | 0.000302685 |
| CREB3L1 | IMPA2 | 0.000111802 |
| CREB3L1 | LIN52 | 4.93E-05 |
| CREB3L1 | SCARF1 | 4.72E-05 |
| CREB3L1 | PC | 1.00E-06 |
| CREB3L1 | OAZ3 | 3.31E-07 |
| CREB3L1 | IL4I1 | 6.87E-08 |
| CREB3L1 | B3GALNT2 | 5.15E-08 |
| CREB3L1 | GDPD5 | 2.95E-09 |
| CREB3L1 | C15orf48 | 6.20E-18 |
| CREB3L1 | HIP1R | 4.24E-19 |
| CREB3L1 | SPIN3 | 2.30E-19 |
| CREB3L1 | SSTR2 | 6.07E-20 |
| CREB3L2 | ARRDC3 | 3.542610622 |
| CREB3L2 | RBMS3 | 2.962219605 |
| CREB3L2 | RECQL5 | 2.689081457 |
| CREB3L2 | ZNF277 | 2.630700196 |
| CREB3L2 | HSP90B1 | 2.293148712 |
| CREB3L2 | P4HA2 | 2.287971157 |
| CREB3L2 | SSBP4 | 2.220513175 |
| CREB3L2 | PLXNB1 | 2.164804126 |
| CREB3L2 | SEC24A | 2.144749046 |
| CREB3L2 | USP53 | 2.092387962 |
| CREB3L2 | POLD2 | 2.059998931 |
| CREB3L2 | KDM5B | 1.880356197 |
| CREB3L2 | B4GALT3 | 1.875580429 |
| CREB3L2 | GHR | 1.818751404 |
| CREB3L2 | CLK4 | 1.77114944 |
| CREB3L2 | CSTF3 | 1.680196689 |
| CREB3L2 | GSN | 1.62020892 |
| CREB3L2 | TIMP2 | 1.508935612 |
| CREB3L2 | SLC27A4 | 1.504838676 |
| CREB3L2 | ATXN2 | 1.50252093 |
| CREB3L2 | MDFIC | 1.438810667 |
| CREB3L2 | PDE5A | 1.41159219 |
| CREB3L2 | HPCAL1 | 1.322279757 |
| CREB3L2 | SPTBN1 | 1.249770248 |
| CREB3L2 | TAB3 | 1.244516384 |
| CREB3L2 | PERP | 1.186572564 |
| CREB3L2 | PTCH1 | 1.123321058 |
| CREB3L2 | ATF5 | 1.122957155 |
| CREB3L2 | TPM2 | 1.104097358 |
| CREB3L2 | COL18A1 | 1.098636986 |
| CREB3L2 | H1FX | 1.091709635 |
| CREB3L2 | ID4 | 1.035209437 |
| CREB3L2 | MIER2 | 1.02477568 |
| CREB3L2 | CREB3L2 | 1 |
| CREB3L2 | RAP1GAP | 0.674811904 |
| CREB3L2 | GRIK2 | 0.674007158 |
| CREB3L4 | TMEM106B | 3.488158022 |
| CREB3L4 | CLEC14A | 3.34982075 |
| CREB3L4 | TOMM34 | 2.646412943 |
| CREB3L4 | G2E3 | 2.317175451 |
| CREB3L4 | PRICKLE1 | 2.263628612 |
| CREB3L4 | DRAM1 | 2.154570824 |
| CREB3L4 | STXBP3 | 2.065465723 |
| CREB3L4 | FYTTD1 | 2.039199922 |
| CREB3L4 | UBE2D1 | 1.983374494 |
| CREB3L4 | HIPK1 | 1.858355258 |
| CREB3L4 | CBWD5 | 1.85202341 |
| CREB3L4 | UTP18 | 1.818046294 |
| CREB3L4 | ESRRA | 1.789184907 |
| CREB3L4 | FAM200A | 1.677396344 |
| CREB3L4 | PCNP | 1.61966028 |
| CREB3L4 | FHL1 | 1.529099854 |
| CREB3L4 | NOL9 | 1.474735091 |
| CREB3L4 | PARP11 | 1.44675121 |
| CREB3L4 | SLC35E1 | 1.428300536 |
| CREB3L4 | KIAA0232 | 1.426944824 |
| CREB3L4 | RSBN1 | 1.335059611 |
| CREB3L4 | ACADVL | 1.196871838 |
| CREB3L4 | SUMF1 | 1.166025378 |
| CREB3L4 | ANK3 | 1.15973667 |
| CREB3L4 | RAB10 | 1.138081461 |
| CREB3L4 | ATXN1 | 1.075046314 |
| CREB3L4 | IKBKB | 0.955777706 |
| CREB3L4 | PTGIR | 0.949421827 |
| CREB3L4 | FECH | 0.943311484 |
| CREB3L4 | XAF1 | 0.845450018 |
| CREB3L4 | MAPK1 | 0.820701715 |
| CREB3L4 | NISCH | 0.811239207 |
| CREB3L4 | RAPGEF2 | 0.779498669 |
| CREB3L4 | ENTPD5 | 0.777243199 |
| CREB3L4 | NEK1 | 0.75171835 |
| CREB3L4 | TSR2 | 0.668241972 |
| CREB3L4 | CNOT2 | 0.663654275 |
| CREB3L4 | EHBP1 | 0.612600755 |
| CREB3L4 | TRIP12 | 0.599730785 |
| CREB3L4 | CDIPT | 0.599421752 |
| CREB3L4 | PHC1 | 0.586042298 |
| CREB3L4 | USP21 | 0.578901726 |
| CREB3L4 | CFLAR | 0.550332038 |
| CREB3L4 | SYNRG | 0.540003061 |
| CREB3L4 | CERK | 0.537946878 |
| CREB3L4 | TCF7L2 | 0.532781871 |
| CREB3L4 | CNOT7 | 0.525534847 |
| CREB3L4 | CADM1 | 0.525424763 |
| CREB3L4 | OLA1 | 0.522225645 |
| CREB3L4 | HLA-DQA1 | 0.514964267 |
| CREB3L4 | EHD3 | 0.448080535 |
| CREB3L4 | RNF20 | 0.443279379 |
| CREB3L4 | LARP7 | 0.441221974 |
| CREB3L4 | LRRFIP1 | 0.439206182 |
| CREB3L4 | MEF2A | 0.437802904 |
| CREB3L4 | SLC35F2 | 0.427264651 |
| CREB3L4 | RBM25 | 0.42554212 |
| CREB3L4 | PLEKHH2 | 0.395216833 |
| CREB3L4 | MAP1B | 0.385903333 |
| CREB3L4 | RBBP4 | 0.371577791 |
| CREB3L4 | ZNF93 | 0.358211681 |
| CREB3L4 | SAMD4B | 0.32114252 |
| CREB3L4 | SMAD3 | 0.318239547 |
| CREB3L4 | PARP8 | 0.275761884 |
| CREB3L4 | BLCAP | 0.271217093 |
| CREB3L4 | IRF9 | 0.264741832 |
| CREB3L4 | C11orf70 | 0.262459766 |
| CREB3L4 | FOXRED2 | 0.259233282 |
| CREB3L4 | NUDT9 | 0.258343552 |
| CREB3L4 | LPP | 0.243454541 |
| CREB3L4 | PDCD6IP | 0.242241516 |
| CREB3L4 | HDX | 0.238275749 |
| CREB3L4 | SYAP1 | 0.236953411 |
| CREB3L4 | USP1 | 0.230442581 |
| CREB3L4 | ZNF644 | 0.223743836 |
| CREB3L4 | INTS10 | 0.220544064 |
| CREB3L4 | F3 | 0.208201027 |
| CREB3L4 | LDB2 | 0.19447772 |
| CREB3L4 | TBX3 | 0.193677736 |
| CREB3L4 | TSPAN31 | 0.187604055 |
| CREB3L4 | RFT1 | 0.183473602 |
| CREB3L4 | MAP1LC3A | 0.180457486 |
| CREB3L4 | ESR1 | 0.173509744 |
| CREB3L4 | NAP1L4 | 0.172766 |
| CREB3L4 | ZNF446 | 0.160658246 |
| CREB3L4 | ELAVL1 | 0.143379611 |
| CREB3L4 | ESCO1 | 0.142550504 |
| CREB3L4 | YIPF1 | 0.141457292 |
| CREB3L4 | MITF | 0.134766961 |
| CREB3L4 | CDS1 | 0.134369208 |
| CREB3L4 | MED28 | 0.13383877 |
| CREB3L4 | AUH | 0.12360375 |
| CREB3L4 | CRIM1 | 0.122467897 |
| CREB3L4 | RHOBTB1 | 0.118555821 |
| CREB3L4 | CD63 | 0.108514581 |
| CREB3L4 | UBE2W | 0.097229533 |
| CREB3L4 | IFT172 | 0.08980052 |
| CREB3L4 | HIST1H4H | 0.083072587 |
| CREB3L4 | PHACTR3 | 0.070096621 |
| CREB3L4 | HOXA9 | 0.068115129 |
| CREB3L4 | ALDH3B1 | 0.06722768 |
| CREB3L4 | HIST1H2AL | 0.059081636 |
| CREB3L4 | ZNHIT2 | 0.058827409 |
| CREB3L4 | HERC5 | 0.053794281 |
| CREB3L4 | RAB40C | 0.044794192 |
| CREB5 | PDPN | 2.356609726 |
| CREB5 | PAF1 | 2.262625225 |
| CREB5 | NUP98 | 2.114661242 |
| CREB5 | AKAP12 | 2.0005206 |
| CREB5 | TPP2 | 1.969028134 |
| CREB5 | MAPK8 | 1.813192437 |
| CREB5 | LMO7 | 1.521203799 |
| CREB5 | CDKN2AIP | 1.487217935 |
| CREB5 | XRN2 | 1.329971386 |
| CREB5 | KLF9 | 1.233227202 |
| CREB5 | CD2AP | 1.208362806 |
| CREB5 | TNFAIP6 | 1.007400716 |
| CREB5 | S100A9 | 1.002966755 |
| CREB5 | CREB5 | 1 |
| CREB5 | NFKBIZ | 0.985593252 |
| CREB5 | RCE1 | 0.969679013 |
| CREB5 | CRYAB | 0.958693315 |
| CREB5 | CA12 | 0.935174715 |
| CREB5 | SLC25A40 | 0.910178273 |
| CREB5 | MYADM | 0.794684212 |
| CREB5 | GEM | 0.760800104 |
| CREB5 | RORA | 0.702480997 |
| CREB5 | CDC42BPB | 0.6578879 |
| CREB5 | MAF | 0.655153693 |
| CREB5 | WNT11 | 0.523606348 |
| CREB5 | ENPP2 | 0.522956447 |
| CREB5 | ZBTB1 | 0.516645843 |
| CREB5 | PTP4A1 | 0.471505331 |
| CREB5 | ID4 | 0.45137168 |
| CREB5 | FAM20C | 0.434818856 |
| CREB5 | DAAM1 | 0.248013292 |
| CREB5 | FOXP1 | 0.218871087 |
| CREB5 | IARS | 0.183805399 |
| CREB5 | CSNK1D | 0.181748103 |
| CREB5 | ISG20 | 0.171503325 |
| CREB5 | PHACTR3 | 0.095069822 |
| CREB5 | LYRM1 | 0.061795944 |
| CREBL2 | RPL21 | 5.778663609 |
| CREBL2 | TMEM165 | 3.59474417 |
| CREBL2 | CHRDL1 | 2.536154267 |
| CREBL2 | NEGR1 | 2.388085567 |
| CREBL2 | CYBRD1 | 2.028749913 |
| CREBL2 | MOCS3 | 1.563360625 |
| CREBL2 | CXCR4 | 1.562958198 |
| CREBL2 | CRKL | 1.47753687 |
| CREBL2 | GALNT13 | 1.117375778 |
| CREBL2 | NRAS | 1.017630646 |
| CREBL2 | ISOC2 | 0.826178502 |
| CREBL2 | ALDH1A1 | 0.81718249 |
| CREBL2 | AVPI1 | 0.779642172 |
| CREBL2 | ATRX | 0.67845277 |
| CREBL2 | EDN1 | 0.646558753 |
| CREBL2 | SYNE1 | 0.644980701 |
| CREBL2 | RNF144B | 0.574421321 |
| CREBL2 | NFKBIA | 0.538997011 |
| CREBL2 | ANGPTL1 | 0.519508352 |
| CREBL2 | TP53I11 | 0.494665691 |
| CREBL2 | TAOK3 | 0.485177353 |
| CREBL2 | NBR1 | 0.418037977 |
| CREBL2 | TGFBR3 | 0.380902464 |
| CREBL2 | COL8A1 | 0.369296013 |
| CREBL2 | GPD2 | 0.366049666 |
| CREBL2 | BCL6 | 0.318340658 |
| CREBL2 | ATF7IP | 0.301363278 |
| CREBL2 | USP37 | 0.289326645 |
| CREBL2 | ARRDC3 | 0.28660845 |
| CREBL2 | SPOP | 0.236666413 |
| CREBL2 | UAP1 | 0.230729972 |
| CREBL2 | ARL4C | 0.210829919 |
| CREBL2 | AZIN1 | 0.199356495 |
| CREBL2 | PACRGL | 0.125552933 |
| CREBL2 | LMO2 | 0.113906979 |
| CREBL2 | CYP3A5 | 0.053915181 |
| CREM | TPM3 | 5.354640104 |
| CREM | METRNL | 2.317599031 |
| CREM | CDKN1B | 2.246954915 |
| CREM | ANGPTL1 | 1.829749267 |
| CREM | NCALD | 1.640435892 |
| CREM | C7orf50 | 1.424925056 |
| CREM | STX5 | 1.401256225 |
| CREM | CTNNA1 | 1.210168267 |
| CREM | CREM | 1 |
| CREM | PAFAH1B1 | 0.989421187 |
| CREM | XRN2 | 0.868881095 |
| CREM | NUFIP2 | 0.847056546 |
| CREM | RARA | 0.832013864 |
| CREM | ID3 | 0.796900922 |
| CREM | KLHL2 | 0.781169774 |
| CREM | RPN1 | 0.728805175 |
| CREM | RAB11FIP1 | 0.685807734 |
| CREM | NAMPT | 0.613465058 |
| CREM | PAPOLA | 0.437903895 |
| CREM | FAM107B | 0.436115355 |
| CREM | VCP | 0.418243934 |
| CREM | TSG101 | 0.418235631 |
| CREM | TXNL1 | 0.369631799 |
| CREM | DDX51 | 0.325631357 |
| CREM | SESTD1 | 0.324130426 |
| CREM | CTNNB1 | 0.323955581 |
| CREM | OTUD1 | 0.30217294 |
| CREM | WISP1 | 0.290328988 |
| CREM | LGR5 | 0.285545842 |
| CREM | GPBP1 | 0.270788142 |
| CREM | C6orf106 | 0.270516413 |
| CREM | PAF1 | 0.264549413 |
| CREM | ZBTB2 | 0.259852849 |
| CREM | SERF2 | 0.244998404 |
| CREM | PDZD8 | 0.239742618 |
| CREM | GPR183 | 0.237981168 |
| CREM | PAK3 | 0.229194456 |
| CREM | FOSL1 | 0.225745006 |
| CREM | MARK4 | 0.220103635 |
| CREM | UBQLN1 | 0.218598509 |
| CREM | TTLL7 | 0.20119265 |
| CREM | YWHAG | 0.19123723 |
| CREM | TRAP1 | 0.190118688 |
| CREM | HOXA13 | 0.168930137 |
| CREM | NCAM1 | 0.159979808 |
| CREM | C2orf49 | 0.137759844 |
| CREM | CYB5D2 | 0.134162558 |
| CREM | SLC25A40 | 0.098829937 |
| CREM | KIF17 | 0.046689085 |
| CREM | TAF5 | 0.041906981 |
| CREM | MYOM2 | 0.040148609 |
| CREM | ZNF365 | 0.023527046 |
| CREM | KCNT2 | 0.020438037 |
| CREM | CDS1 | 0.012280198 |
| CREM | SORCS1 | 0.007654608 |
| CREM | SLC4A3 | 2.89E-06 |
| CTCF | MAP4K4 | 3.59354583 |
| CTCF | COL10A1 | 3.252201545 |
| CTCF | TMEM147 | 3.111272716 |
| CTCF | TMEM40 | 3.03872401 |
| CTCF | XYLT1 | 2.802774337 |
| CTCF | ITGAE | 2.772662789 |
| CTCF | CCDC71 | 2.609898397 |
| CTCF | NUP62 | 2.577986331 |
| CTCF | ZNF469 | 2.574624616 |
| CTCF | TNPO1 | 2.454875414 |
| CTCF | COX16 | 2.421939766 |
| CTCF | PDLIM2 | 2.389946162 |
| CTCF | TRNT1 | 2.26940392 |
| CTCF | MRPL46 | 2.242109475 |
| CTCF | EEF2K | 2.23812071 |
| CTCF | CDCA8 | 2.176489888 |
| CTCF | TNFRSF19 | 2.14391032 |
| CTCF | CXXC5 | 2.080720829 |
| CTCF | NEK2 | 2.014659778 |
| CTCF | MMADHC | 1.937535252 |
| CTCF | S1PR1 | 1.911616275 |
| CTCF | MELK | 1.88350219 |
| CTCF | GNPDA2 | 1.807045214 |
| CTCF | TGFB1I1 | 1.753749391 |
| CTCF | GADD45GIP1 | 1.740499371 |
| CTCF | MIS12 | 1.730533057 |
| CTCF | RUNX1 | 1.699247861 |
| CTCF | FAM3A | 1.671613682 |
| CTCF | CENPJ | 1.643407804 |
| CTCF | SLC39A3 | 1.603975108 |
| CTCF | ZNF146 | 1.552630154 |
| CTCF | PARP16 | 1.542830385 |
| CTCF | NUMA1 | 1.542425674 |
| CTCF | ISCU | 1.519955485 |
| CTCF | RPL24 | 1.51678904 |
| CTCF | MXI1 | 1.492199436 |
| CTCF | HCFC1 | 1.480074854 |
| CTCF | PDZRN3 | 1.457467418 |
| CTCF | FAM57A | 1.415435236 |
| CTCF | ORMDL2 | 1.377334603 |
| CTCF | TXN | 1.340419435 |
| CTCF | RAC1 | 1.339702046 |
| CTCF | CHCHD3 | 1.338223726 |
| CTCF | ASXL1 | 1.337075992 |
| CTCF | AFF4 | 1.332727223 |
| CTCF | DCAF8 | 1.310737531 |
| CTCF | LMAN1 | 1.308998582 |
| CTCF | H2AFV | 1.279913079 |
| CTCF | STAM2 | 1.241097435 |
| CTCF | STIM2 | 1.232185021 |
| CTCF | CDC20 | 1.230507305 |
| CTCF | SDHAP1 | 1.229216638 |
| CTCF | OLFML3 | 1.21330149 |
| CTCF | ELL | 1.18868383 |
| CTCF | NANS | 1.114832883 |
| CTCF | PHTF2 | 1.090801359 |
| CTCF | TMCO1 | 1.086268791 |
| CTCF | DDAH2 | 1.079283146 |
| CTCF | C1orf123 | 1.07109503 |
| CTCF | MALAT1 | 1.067729477 |
| CTCF | TSEN54 | 1.057552803 |
| CTCF | MDH2 | 1.046516718 |
| CTCF | ATXN1 | 1.03239162 |
| CTCF | EMILIN1 | 1.02729198 |
| CTCF | TMUB1 | 1.027251313 |
| CTCF | CIRBP | 1.011097766 |
| CTCF | SCUBE3 | 1.004086207 |
| CTCF | CTCF | 1 |
| CTCF | HIF1A | 0.992942411 |
| CTCF | CEP55 | 0.974661655 |
| CTCF | GCLM | 0.973467304 |
| CTCF | CWC15 | 0.940064327 |
| CTCF | G6PC3 | 0.925169807 |
| CTCF | TMEM9B | 0.871887631 |
| CTCF | ACTR2 | 0.869195528 |
| CTCF | ABCB8 | 0.868097127 |
| CTCF | PLTP | 0.850331489 |
| CTCF | UXT | 0.847354145 |
| CTCF | COMMD4 | 0.835211698 |
| CTCF | NTAN1 | 0.82577541 |
| CTCF | ATXN10 | 0.813293897 |
| CTCF | RPL41 | 0.804683493 |
| CTCF | EFEMP2 | 0.797044031 |
| CTCF | GOLGA3 | 0.790823793 |
| CTCF | PRIM1 | 0.776391916 |
| CTCF | CRTC2 | 0.774276873 |
| CTCF | GNB2 | 0.771987214 |
| CTCF | RPL19 | 0.76499423 |
| CTCF | ZNF384 | 0.747871058 |
| CTCF | MARS2 | 0.718843212 |
| CTCF | NPHP3 | 0.718609093 |
| CTCF | CHCHD5 | 0.709466182 |
| CTCF | LSM2 | 0.70261703 |
| CTCF | FKBP7 | 0.679920186 |
| CTCF | C1QTNF6 | 0.667071693 |
| CTCF | COMP | 0.658710472 |
| CTCF | AP1M1 | 0.637959057 |
| CTCF | MMP14 | 0.620079788 |
| CTCF | MFN2 | 0.613733063 |
| CTCF | SPON2 | 0.602246017 |
| CTCF | GABARAPL1 | 0.565520552 |
| CTCF | SLC16A5 | 0.564317604 |
| CTCF | SLC26A7 | 0.562167821 |
| CTCF | PDIA6 | 0.560610741 |
| CTCF | ANGPTL2 | 0.550653144 |
| CTCF | MORN1 | 0.547265104 |
| CTCF | MSN | 0.547032213 |
| CTCF | STMN3 | 0.5422156 |
| CTCF | RPS13 | 0.527606437 |
| CTCF | PHF19 | 0.518768489 |
| CTCF | SET | 0.51561915 |
| CTCF | PCGF3 | 0.513472325 |
| CTCF | EEF1A1 | 0.510091449 |
| CTCF | ZFYVE1 | 0.499444117 |
| CTCF | CPT2 | 0.497667965 |
| CTCF | ZYX | 0.4957137 |
| CTCF | NOLC1 | 0.49120768 |
| CTCF | RPL5 | 0.482080501 |
| CTCF | LGR4 | 0.480390864 |
| CTCF | THRAP3 | 0.469393562 |
| CTCF | RPLP1 | 0.465449234 |
| CTCF | CLEC11A | 0.4611332 |
| CTCF | TRAPPC6A | 0.456922178 |
| CTCF | SLC37A3 | 0.453050855 |
| CTCF | COPB2 | 0.440032296 |
| CTCF | C17orf58 | 0.436459338 |
| CTCF | RET | 0.433948079 |
| CTCF | TACC3 | 0.427520682 |
| CTCF | CEP63 | 0.426951562 |
| CTCF | RAB15 | 0.413412473 |
| CTCF | SLC30A6 | 0.392315402 |
| CTCF | TM9SF2 | 0.388534974 |
| CTCF | AURKA | 0.370019134 |
| CTCF | MDH1B | 0.368774235 |
| CTCF | FAM24B | 0.366210124 |
| CTCF | GATA3 | 0.365095755 |
| CTCF | NARF | 0.360033427 |
| CTCF | ALMS1 | 0.353882462 |
| CTCF | ZNF85 | 0.353592468 |
| CTCF | FZD1 | 0.347942481 |
| CTCF | TSPAN6 | 0.34516507 |
| CTCF | FAT1 | 0.340678468 |
| CTCF | FOXL2 | 0.34018339 |
| CTCF | LIN54 | 0.338203118 |
| CTCF | FAM181B | 0.337868487 |
| CTCF | ZNF691 | 0.337028826 |
| CTCF | ITGA3 | 0.326689988 |
| CTCF | INCENP | 0.305975834 |
| CTCF | C2CD3 | 0.304270837 |
| CTCF | TOR1B | 0.302782952 |
| CTCF | ANKRD26 | 0.302502303 |
| CTCF | PTER | 0.300508209 |
| CTCF | ZNF148 | 0.297469685 |
| CTCF | CCNK | 0.291294013 |
| CTCF | NPRL3 | 0.290895352 |
| CTCF | CAMKK2 | 0.290724059 |
| CTCF | ANO1 | 0.275080098 |
| CTCF | THAP2 | 0.262725536 |
| CTCF | GPR161 | 0.26129146 |
| CTCF | SPIN1 | 0.25819863 |
| CTCF | ZC3H10 | 0.256332894 |
| CTCF | COQ6 | 0.255843372 |
| CTCF | RAB6B | 0.254179812 |
| CTCF | SULT2B1 | 0.24443392 |
| CTCF | ZDHHC4 | 0.242212239 |
| CTCF | MRPS12 | 0.239700496 |
| CTCF | ZNF292 | 0.234451838 |
| CTCF | OFD1 | 0.231288645 |
| CTCF | ZNF506 | 0.228737068 |
| CTCF | DDX19A | 0.226879742 |
| CTCF | NR2F6 | 0.224788547 |
| CTCF | ERGIC1 | 0.22443174 |
| CTCF | DDX17 | 0.218065031 |
| CTCF | INTS7 | 0.217030015 |
| CTCF | MTA2 | 0.210686785 |
| CTCF | NR1H2 | 0.206397366 |
| CTCF | SPOP | 0.202992723 |
| CTCF | RAP2B | 0.19862695 |
| CTCF | CCNL2 | 0.19476109 |
| CTCF | C1orf52 | 0.193061581 |
| CTCF | ZFP90 | 0.192227245 |
| CTCF | HMG20A | 0.189468307 |
| CTCF | CRISPLD1 | 0.188059323 |
| CTCF | TTPAL | 0.187331769 |
| CTCF | TRPT1 | 0.185076496 |
| CTCF | CDC34 | 0.183839261 |
| CTCF | THAP7 | 0.182861407 |
| CTCF | GMPPB | 0.17845411 |
| CTCF | PPP2CB | 0.177131482 |
| CTCF | ANXA11 | 0.174042911 |
| CTCF | SLC30A5 | 0.173986119 |
| CTCF | TTC31 | 0.172054136 |
| CTCF | CTU1 | 0.171924852 |
| CTCF | MRPS22 | 0.1701643 |
| CTCF | MRPS34 | 0.151835685 |
| CTCF | FCHSD2 | 0.148843956 |
| CTCF | PPFIA1 | 0.14764378 |
| CTCF | RARS | 0.146725749 |
| CTCF | TUFT1 | 0.140482895 |
| CTCF | ZNF12 | 0.139278392 |
| CTCF | DBP | 0.127727076 |
| CTCF | CLOCK | 0.118344423 |
| CTCF | MALT1 | 0.099534245 |
| CTCF | RNF123 | 0.087505675 |
| CTCF | TSPAN2 | 0.081888906 |
| CTCF | AP1S2 | 0.077680966 |
| CTCF | NOL6 | 0.073793354 |
| CTCF | GCSH | 0.071721986 |
| CTCF | VPS8 | 0.070545846 |
| CTCF | BEND3 | 0.070273277 |
| CTCF | CCDC43 | 0.044485311 |
| CTCF | CDK20 | 0.041499485 |
| CTCF | RWDD4 | 0.022863785 |
| CTCF | LHPP | 0.014389059 |
| CTCF | NDUFV1 | 0.012618497 |
| CTCF | MYO1D | 0.011347229 |
| CTCF | TTC30B | 0.010265563 |
| CTCF | LIPG | 0.005953026 |
| CTCF | MAVS | 0.004828035 |
| CTCF | PCSK5 | 2.79E-05 |
| CTCF | PDCD2L | 2.45E-06 |
| CTCF | TMEM106A | 5.67E-08 |
| CTCF | KCTD1 | 2.91E-09 |
| CTCF | ZNF827 | 9.33E-19 |
| CTCF | TRIM11 | 2.85E-19 |
| CUX1 | S100A14 | 5.051121027 |
| CUX1 | KIF20B | 4.556441738 |
| CUX1 | SFPQ | 2.558240602 |
| CUX1 | KIF2A | 2.414512815 |
| CUX1 | EAPP | 2.402377696 |
| CUX1 | TXNDC16 | 2.366375691 |
| CUX1 | CYB5A | 2.322758393 |
| CUX1 | UNG | 2.268538218 |
| CUX1 | FAM117B | 2.044239668 |
| CUX1 | GMCL1 | 1.883536437 |
| CUX1 | FDXR | 1.815791195 |
| CUX1 | CDKN2D | 1.739415203 |
| CUX1 | NTRK1 | 1.483741602 |
| CUX1 | SNHG6 | 1.443369615 |
| CUX1 | FARS2 | 1.392900884 |
| CUX1 | FIS1 | 1.359506123 |
| CUX1 | CEP70 | 1.330080112 |
| CUX1 | IRAK4 | 1.264681594 |
| CUX1 | LIN52 | 1.255003313 |
| CUX1 | FCHSD1 | 1.250301337 |
| CUX1 | ERGIC3 | 1.229553967 |
| CUX1 | MTOR | 1.14757293 |
| CUX1 | DDX42 | 1.126806133 |
| CUX1 | LNX2 | 1.091210583 |
| CUX1 | RPS16 | 1.090886559 |
| CUX1 | POLR2G | 1.074587192 |
| CUX1 | NDUFS8 | 1.017777492 |
| CUX1 | CUX1 | 1 |
| CUX1 | ITFG2 | 0.990585441 |
| CUX1 | ABCD1 | 0.976284965 |
| CUX1 | PRDX4 | 0.945050831 |
| CUX1 | ELL | 0.880974434 |
| CUX1 | MOSPD3 | 0.832783962 |
| CUX1 | USP36 | 0.792972313 |
| CUX1 | HNRNPA2B1 | 0.764619148 |
| CUX1 | COMMD6 | 0.756851938 |
| CUX1 | HES2 | 0.756841849 |
| CUX1 | UBR4 | 0.70536229 |
| CUX1 | CDC45 | 0.650752451 |
| CUX1 | TLCD1 | 0.637673016 |
| CUX1 | EIF4EBP2 | 0.581681471 |
| CUX1 | FLAD1 | 0.578071947 |
| CUX1 | SMARCA5 | 0.558911068 |
| CUX1 | BEX5 | 0.493215515 |
| CUX1 | MEX3C | 0.481576617 |
| CUX1 | RNFT1 | 0.461904663 |
| CUX1 | PHF12 | 0.453111842 |
| CUX1 | C11orf65 | 0.450821674 |
| CUX1 | PRKCB | 0.435050484 |
| CUX1 | DUSP3 | 0.397864071 |
| CUX1 | RPE | 0.394816559 |
| CUX1 | MYH11 | 0.391057744 |
| CUX1 | CRNDE | 0.376217737 |
| CUX1 | ARAP1 | 0.37561587 |
| CUX1 | TPRA1 | 0.370125638 |
| CUX1 | ELP3 | 0.363868736 |
| CUX1 | TAP1 | 0.344323118 |
| CUX1 | PTK7 | 0.334848633 |
| CUX1 | SP2 | 0.332215745 |
| CUX1 | SLC1A4 | 0.328581247 |
| CUX1 | ORAOV1 | 0.327259296 |
| CUX1 | TMEM102 | 0.320466461 |
| CUX1 | NBEAL2 | 0.310561168 |
| CUX1 | DLG3 | 0.298467565 |
| CUX1 | REM2 | 0.289497331 |
| CUX1 | AP1G1 | 0.285896584 |
| CUX1 | GINS4 | 0.284982503 |
| CUX1 | KIF26A | 0.279421871 |
| CUX1 | CEP57L1 | 0.273177569 |
| CUX1 | ROBO3 | 0.272908589 |
| CUX1 | KCTD18 | 0.270262836 |
| CUX1 | DHRS4L2 | 0.270224693 |
| CUX1 | FAM13B | 0.263480862 |
| CUX1 | RBM15 | 0.246233243 |
| CUX1 | SMAD2 | 0.245576934 |
| CUX1 | PDRG1 | 0.241172011 |
| CUX1 | FKBP4 | 0.235292652 |
| CUX1 | PRPF4B | 0.22149524 |
| CUX1 | TRIM62 | 0.220861878 |
| CUX1 | STRN | 0.209022424 |
| CUX1 | SNX24 | 0.205541378 |
| CUX1 | PSMC5 | 0.204717336 |
| CUX1 | DNAL4 | 0.204016613 |
| CUX1 | LZTFL1 | 0.202189639 |
| CUX1 | THBD | 0.193115867 |
| CUX1 | TFAP2C | 0.1913847 |
| CUX1 | EFNA1 | 0.185990446 |
| CUX1 | EIF2AK1 | 0.185125757 |
| CUX1 | ASB1 | 0.181297854 |
| CUX1 | ACTR5 | 0.180405738 |
| CUX1 | ASCC2 | 0.179743425 |
| CUX1 | FMN1 | 0.179345176 |
| CUX1 | MTR | 0.179030193 |
| CUX1 | TOR1B | 0.16950902 |
| CUX1 | IMPA2 | 0.168846389 |
| CUX1 | MMD | 0.161782773 |
| CUX1 | CNOT3 | 0.1597982 |
| CUX1 | HAND1 | 0.155363909 |
| CUX1 | ZSCAN29 | 0.14990467 |
| CUX1 | CYP2R1 | 0.144864081 |
| CUX1 | RRS1 | 0.143448378 |
| CUX1 | SNX11 | 0.137227163 |
| CUX1 | ANKRD27 | 0.122970639 |
| CUX1 | ARMCX6 | 0.114602207 |
| CUX1 | ICA1 | 0.113933936 |
| CUX1 | KIAA0895L | 0.112720367 |
| CUX1 | E2F8 | 0.095091404 |
| CUX1 | FAM86DP | 0.091050637 |
| CUX1 | TAF10 | 0.089816978 |
| CUX1 | BIN3 | 0.088861078 |
| CUX1 | FGFR1OP | 0.081632617 |
| CUX1 | PSMB9 | 0.049993406 |
| CUX1 | NAA40 | 0.041734711 |
| CUX1 | ITPKC | 5.27E-05 |
| CYB5R1 | AQP3 | 4.357889285 |
| CYB5R1 | PRKCE | 2.410865522 |
| CYB5R1 | SRSF7 | 2.039195001 |
| CYB5R1 | HLA-F | 1.989572601 |
| CYB5R1 | GLIS3 | 1.818507574 |
| DAB2 | CCDC80 | 34.66423454 |
| DAB2 | C1R | 29.90641933 |
| DAB2 | THY1 | 29.75883648 |
| DAB2 | SOD3 | 29.33027126 |
| DAB2 | CXCL12 | 26.76969341 |
| DAB2 | CLU | 25.3276649 |
| DAB2 | DCN | 23.71274656 |
| DAB2 | SERPING1 | 21.54175817 |
| DAB2 | PMP22 | 21.3013855 |
| DAB2 | FGL2 | 20.68818676 |
| DAB2 | ABI3BP | 19.27785112 |
| DAB2 | APOE | 17.16503439 |
| DAB2 | IGFBP4 | 16.80554094 |
| DAB2 | IL6ST | 14.70217434 |
| DAB2 | SLIT3 | 14.37161753 |
| DAB2 | LAMA4 | 13.61352187 |
| DAB2 | ADD3 | 12.50167278 |
| DAB2 | FBN1 | 11.85216437 |
| DAB2 | STMN2 | 10.09190712 |
| DAB2 | FAM198B | 8.233779077 |
| DAB2 | PRNP | 8.028546015 |
| DAB2 | ANXA1 | 7.945549356 |
| DAB2 | ARL6IP5 | 7.839144365 |
| DAB2 | FHL1 | 7.350878751 |
| DAB2 | CSGALNACT1 | 7.073407447 |
| DAB2 | SF3B1 | 7.065102989 |
| DAB2 | CALD1 | 6.526516677 |
| DAB2 | PODN | 6.475259964 |
| DAB2 | NTRK2 | 5.638865139 |
| DAB2 | SCPEP1 | 5.436747193 |
| DAB2 | FRMD6 | 5.425455523 |
| DAB2 | PRRX1 | 5.422728104 |
| DAB2 | WASF2 | 5.332652709 |
| DAB2 | DHRS7 | 5.063434231 |
| DAB2 | ASPH | 4.809741087 |
| DAB2 | EMILIN1 | 4.641109827 |
| DAB2 | GOLIM4 | 4.380426926 |
| DAB2 | SPTBN1 | 4.06409696 |
| DAB2 | RTN4 | 3.989356989 |
| DAB2 | SRRM1 | 3.937739341 |
| DAB2 | GPNMB | 3.91523418 |
| DAB2 | MYCBP2 | 3.903652083 |
| DAB2 | DIO2 | 3.574173661 |
| DAB2 | BCAP29 | 3.53641461 |
| DAB2 | IL11RA | 3.482598438 |
| DAB2 | PEA15 | 3.439080987 |
| DAB2 | ECM1 | 3.345508062 |
| DAB2 | CNN3 | 3.275744189 |
| DAB2 | VCAM1 | 3.174620536 |
| DAB2 | HSPB6 | 3.023406797 |
| DAB2 | PDGFRA | 2.939124296 |
| DAB2 | KRT14 | 2.891985398 |
| DAB2 | SPARCL1 | 2.634646876 |
| DAB2 | RUNX1 | 2.494614563 |
| DAB2 | KRTDAP | 2.440845842 |
| DAB2 | SUMO2 | 2.185273272 |
| DAB2 | MFGE8 | 2.177983566 |
| DAB2 | OLFML3 | 2.11855761 |
| DAB2 | CALM3 | 2.07712215 |
| DAB2 | DPYSL2 | 2.031565416 |
| DAB2 | SFN | 1.983345226 |
| DAB2 | GPX4 | 1.979821943 |
| DAB2 | NR3C1 | 1.960624956 |
| DAB2 | PTN | 1.91507244 |
| DAB2 | DMKN | 1.866148337 |
| DAB2 | PPP1CC | 1.865181907 |
| DAB2 | TXNIP | 1.861485546 |
| DAB2 | UROD | 1.851500857 |
| DAB2 | MRPL24 | 1.802617862 |
| DAB2 | CCL2 | 1.782612236 |
| DAB2 | CP | 1.720120243 |
| DAB2 | ICAM1 | 1.702164657 |
| DAB2 | PNN | 1.624137712 |
| DAB2 | RRBP1 | 1.570286203 |
| DAB2 | NFIA | 1.53108494 |
| DAB2 | RAPGEF1 | 1.491806942 |
| DAB2 | RBM26 | 1.477408381 |
| DAB2 | ADAMTS5 | 1.4409991 |
| DAB2 | TRIP10 | 1.439624182 |
| DAB2 | REXO2 | 1.405939561 |
| DAB2 | VEGFA | 1.380351705 |
| DAB2 | ZFP36L2 | 1.366518783 |
| DAB2 | TMOD3 | 1.356677657 |
| DAB2 | ALKBH8 | 1.354444979 |
| DAB2 | ISG15 | 1.351235636 |
| DAB2 | PDIA6 | 1.298715587 |
| DAB2 | CKMT1A | 1.279552112 |
| DAB2 | GNA11 | 1.265852927 |
| DAB2 | SLC27A3 | 1.260684833 |
| DAB2 | KCNJ15 | 1.229988811 |
| DAB2 | TCEAL4 | 1.219407587 |
| DAB2 | C5orf51 | 1.206504603 |
| DAB2 | TCF3 | 1.170741386 |
| DAB2 | S100A13 | 1.170006023 |
| DAB2 | DST | 1.153665755 |
| DAB2 | RUNX1T1 | 1.097853189 |
| DAB2 | SYNCRIP | 1.097187926 |
| DAB2 | CITED2 | 1.074710223 |
| DAB2 | ADAMTS12 | 1.05209337 |
| DAB2 | PRDX3 | 1.029171863 |
| DAB2 | DAB2 | 1 |
| DBP | H1FX | 2.376012246 |
| DBP | ZNF165 | 2.225796787 |
| DBP | P4HB | 2.174758699 |
| DBP | RIPK1 | 2.166369734 |
| DBP | PARVA | 2.12366637 |
| DBP | PHOSPHO2 | 1.763941671 |
| DBP | RTN4 | 1.738301987 |
| DBP | CDH13 | 1.712159878 |
| DBP | CD55 | 1.693472419 |
| DBP | TMUB2 | 1.650369351 |
| DBP | RNASEH2B | 1.614368296 |
| DBP | SDK2 | 1.492935726 |
| DBP | AK4 | 1.455337089 |
| DBP | RB1 | 1.378797709 |
| DBP | GMPPA | 1.253385132 |
| DBP | CCNK | 1.231382773 |
| DBP | DCLK1 | 1.163346598 |
| DBP | DYRK1A | 1.135249622 |
| DBP | IL1R2 | 1.043660257 |
| DBP | UBE2N | 0.828311097 |
| DBP | SGMS1 | 0.817711043 |
| DBP | RIN2 | 0.806155971 |
| DBP | TAF7 | 0.751093153 |
| DBP | CDKN1C | 0.742224148 |
| DBP | NARS2 | 0.73915159 |
| DBP | ZNHIT1 | 0.728262169 |
| DBP | NCOA5 | 0.716480735 |
| DBP | MDH1 | 0.67447356 |
| DBP | HNRNPF | 0.56569779 |
| DBP | PTPRD | 0.5500697 |
| DBP | CBX7 | 0.539603698 |
| DBP | BMPR1A | 0.474171859 |
| DBP | MEST | 0.4143248 |
| DBP | VDAC3 | 0.392642132 |
| DBP | CREBZF | 0.39072457 |
| DBP | RAMP1 | 0.377689763 |
| DBP | APBB2 | 0.365616773 |
| DBP | ADAM23 | 0.354226869 |
| DBP | MRRF | 0.343659004 |
| DBP | HOXC4 | 0.340022119 |
| DBP | JDP2 | 0.327242765 |
| DBP | TOPORS | 0.322660366 |
| DBP | SPATA2L | 0.322622349 |
| DBP | VPS18 | 0.303067458 |
| DBP | KLHL15 | 0.290430165 |
| DBP | EPS8 | 0.285918312 |
| DBP | CCL13 | 0.261413544 |
| DBP | RGS3 | 0.25966642 |
| DBP | PLD1 | 0.254650025 |
| DBP | CTTNBP2 | 0.253316329 |
| DBP | PELI1 | 0.251087507 |
| DBP | FBXO3 | 0.250620923 |
| DBP | DCTN4 | 0.246659997 |
| DBP | TBRG1 | 0.239548327 |
| DBP | CRBN | 0.2347645 |
| DBP | SLC25A14 | 0.222463897 |
| DBP | EIF4A1 | 0.205506014 |
| DBP | HOPX | 0.196703868 |
| DBP | PDHA1 | 0.195812955 |
| DBP | CAMTA1 | 0.195331551 |
| DBP | UNC50 | 0.192121842 |
| DBP | PLCE1 | 0.191353815 |
| DBP | EFR3A | 0.186967725 |
| DBP | LIPC | 0.172962949 |
| DBP | SH3BGR | 0.141731662 |
| DBP | ADIPOR2 | 0.128353538 |
| DBP | MAGEF1 | 0.124655199 |
| DBP | RGS17 | 0.116538822 |
| DBP | MFSD5 | 0.113770176 |
| DBP | MDFI | 0.10950905 |
| DBP | EPHB6 | 0.095770882 |
| DBP | HS6ST2 | 0.073974664 |
| DBP | GLCE | 0.073246509 |
| DDIT3 | BTG1 | 5.352740728 |
| DDIT3 | RND3 | 4.232770124 |
| DDIT3 | HSPA8 | 3.878815372 |
| DDIT3 | LOX | 3.464006848 |
| DDIT3 | MMP17 | 2.186246758 |
| DDIT3 | JUN | 2.167709736 |
| DDIT3 | ESR1 | 2.121482836 |
| DDIT3 | DPT | 2.089121991 |
| DDIT3 | NFKBIZ | 1.828842902 |
| DDIT3 | ZBTB10 | 1.822230466 |
| DDIT3 | SPRED2 | 1.819071203 |
| DDIT3 | SLC19A2 | 1.564622997 |
| DDIT3 | TSHZ2 | 1.536968347 |
| DDIT3 | HERPUD1 | 1.479653778 |
| DDIT3 | GLRX2 | 1.305716462 |
| DDIT3 | CSRNP3 | 1.27102791 |
| DDIT3 | CEBPB | 1.190534927 |
| DDIT3 | BDNF | 1.122986593 |
| DDIT3 | TEF | 1.064602181 |
| DDIT3 | TMEM30A | 1.02373679 |
| DDIT3 | DDIT3 | 1 |
| DDIT3 | CTH | 0.969827403 |
| DDIT3 | AEBP1 | 0.930364301 |
| DDIT3 | FAM53C | 0.873698939 |
| DDIT3 | CDKN1A | 0.768572811 |
| DDIT3 | SDCBP | 0.685245271 |
| DDIT3 | ACOT4 | 0.684150506 |
| DDIT3 | COL25A1 | 0.669717951 |
| DDIT3 | STOM | 0.667575376 |
| DDIT3 | FOSB | 0.666727584 |
| DDIT3 | ACSL5 | 0.640783058 |
| DDIT3 | FHL1 | 0.508274522 |
| DDIT3 | KCNAB1 | 0.501242058 |
| DDIT3 | TRAM1L1 | 0.474111881 |
| DDIT3 | NUPR1 | 0.461577978 |
| DDIT3 | KLHL15 | 0.451129798 |
| DDIT3 | EIF1 | 0.434847837 |
| DEAF1 | CSGALNACT1 | 10.52765582 |
| DEAF1 | LSM5 | 7.359335739 |
| DEAF1 | DPYSL3 | 3.128020634 |
| DEAF1 | EPSTI1 | 2.947197777 |
| DEAF1 | EDEM2 | 2.808027332 |
| DEAF1 | TSC22D1 | 2.787823142 |
| DEAF1 | CCDC102A | 2.478129001 |
| DEAF1 | MICALL2 | 2.421324056 |
| DEAF1 | C12orf29 | 2.255864329 |
| DEAF1 | FAM13A | 2.21127875 |
| DEAF1 | LPCAT3 | 2.164770837 |
| DEAF1 | NEDD4L | 1.997933684 |
| DEAF1 | TXLNG | 1.953895118 |
| DEAF1 | FAM84B | 1.896329051 |
| DEAF1 | DNAJC1 | 1.880996607 |
| DEAF1 | SERTAD2 | 1.840422975 |
| DEAF1 | PRDM8 | 1.800176132 |
| DEAF1 | CEP350 | 1.714591697 |
| DEAF1 | ATF2 | 1.7115536 |
| DEAF1 | RAI2 | 1.684653331 |
| DEAF1 | CDC42EP2 | 1.667821778 |
| DEAF1 | INTS8 | 1.652168526 |
| DEAF1 | ZNF521 | 1.576713183 |
| DEAF1 | TMEM47 | 1.548713913 |
| DEAF1 | VKORC1L1 | 1.542607416 |
| DEAF1 | GSK3A | 1.513091417 |
| DEAF1 | CXorf38 | 1.484132173 |
| DEAF1 | PDZRN3 | 1.420244758 |
| DEAF1 | FAM109B | 1.399425222 |
| DEAF1 | SBNO2 | 1.380922271 |
| DEAF1 | ASS1 | 1.368097166 |
| DEAF1 | RRAGA | 1.298314092 |
| DEAF1 | RRAGC | 1.291146491 |
| DEAF1 | EIF4ENIF1 | 1.267986903 |
| DEAF1 | ZMYND8 | 1.265547526 |
| DEAF1 | MAP2K7 | 1.237003771 |
| DEAF1 | MBD3 | 1.220515643 |
| DEAF1 | TPD52L2 | 1.112798054 |
| DEAF1 | TDG | 1.053713308 |
| DEAF1 | AR | 1.036528779 |
| DEAF1 | SNRPA1 | 1.032301784 |
| DEAF1 | SMAP2 | 1.025229551 |
| DEAF1 | NET1 | 1.005552954 |
| DEAF1 | DEAF1 | 1 |
| DEAF1 | LUC7L2 | 0.969814007 |
| DEAF1 | TMEM100 | 0.951896197 |
| DEAF1 | FBXO3 | 0.951695894 |
| DEAF1 | ODF2 | 0.923157348 |
| DEAF1 | CDC42EP3 | 0.909516792 |
| DEAF1 | FAM200B | 0.888517647 |
| DEAF1 | PITX2 | 0.872254604 |
| DEAF1 | VGLL4 | 0.861144693 |
| DEAF1 | ABHD12 | 0.82433169 |
| DEAF1 | TRIP12 | 0.820371091 |
| DEAF1 | SULT1A1 | 0.799540862 |
| DEAF1 | USP15 | 0.69809682 |
| DEAF1 | HIBCH | 0.685115818 |
| DEAF1 | ZKSCAN5 | 0.62570185 |
| DEAF1 | PHC2 | 0.619274839 |
| DEAF1 | ACVRL1 | 0.572901498 |
| DLX3 | HOXD1 | 3.545150443 |
| DLX3 | ZMIZ1 | 3.037489445 |
| DLX3 | KIF26A | 2.962023472 |
| DLX3 | CYP26B1 | 2.495227916 |
| DLX3 | RALY | 1.057770875 |
| DLX3 | DLX3 | 1 |
| DLX3 | MEIS2 | 0.976521785 |
| DLX3 | FERMT2 | 0.935679224 |
| DLX3 | MAP2K7 | 0.738253547 |
| DLX3 | INHBA | 0.586638738 |
| DLX3 | PURA | 0.512699302 |
| DLX3 | LSM14B | 0.494233179 |
| DLX3 | SAMD11 | 0.46079339 |
| DLX3 | ATP11B | 0.280905266 |
| DLX3 | GLI2 | 0.241686891 |
| DLX3 | DUSP6 | 0.224257875 |
| DLX3 | JAG1 | 0.203172676 |
| E2F1 | DTL | 7.510567129 |
| E2F1 | FEN1 | 5.258547847 |
| E2F1 | KIAA1586 | 3.678189574 |
| E2F1 | UROS | 3.615535671 |
| E2F1 | ATAD5 | 2.575337272 |
| E2F1 | KIF11 | 2.495370377 |
| E2F1 | CDT1 | 2.392593675 |
| E2F1 | TK1 | 1.971700845 |
| E2F1 | SHCBP1 | 1.887501491 |
| E2F1 | RRM2 | 1.780357098 |
| E2F1 | KIFC1 | 1.519478152 |
| E2F1 | DIAPH3 | 1.45148362 |
| E2F1 | KIF18B | 1.446946904 |
| E2F1 | PAFAH1B3 | 1.358787428 |
| E2F1 | FOXM1 | 1.339563692 |
| E2F1 | CDCA7 | 1.338153504 |
| E2F1 | HJURP | 1.238663283 |
| E2F1 | ZBTB17 | 1.063839672 |
| E2F1 | E2F1 | 1 |
| E2F1 | TMLHE | 0.977741812 |
| E2F1 | PRDM11 | 0.777256284 |
| E2F1 | GTSE1 | 0.752068128 |
| E2F1 | ZNF260 | 0.74986157 |
| E2F1 | PTN | 0.717053315 |
| E2F1 | LMNB2 | 0.703541522 |
| E2F1 | ASF1B | 0.643796073 |
| E2F1 | MSH2 | 0.612552166 |
| E2F1 | CKAP2L | 0.576871161 |
| E2F1 | PARP1 | 0.545739345 |
| E2F1 | DUT | 0.539438268 |
| E2F1 | ZNF384 | 0.537317006 |
| E2F1 | HELLS | 0.518517447 |
| E2F1 | CLSPN | 0.492597142 |
| E2F1 | CENPO | 0.380012537 |
| E2F1 | PCNA | 0.330411389 |
| E2F1 | RNF157 | 0.297267867 |
| E2F1 | CDK4 | 0.294147295 |
| E2F1 | TCF19 | 0.271264882 |
| E2F1 | NCLN | 0.264534804 |
| E2F1 | EZH2 | 0.239381294 |
| E2F1 | CAPN5 | 0.229722139 |
| E2F1 | ZNF143 | 0.222445368 |
| E2F1 | BEND6 | 0.217822799 |
| E2F1 | DSN1 | 0.217777548 |
| E2F1 | FEZ1 | 0.213850144 |
| E2F1 | C17orf62 | 0.193673077 |
| E2F1 | SMC3 | 0.190464377 |
| E2F1 | SIRT6 | 0.185309847 |
| E2F1 | TMEM199 | 0.183869688 |
| E2F1 | LRRC42 | 0.140780334 |
| E2F1 | ARG2 | 0.131143331 |
| E2F1 | BRIP1 | 0.105921443 |
| E2F1 | ZNF205 | 0.076822702 |
| E2F1 | ZNF468 | 0.072750421 |
| E2F1 | ZNF550 | 0.062583502 |
| E2F1 | BACH2 | 0.045479788 |
| E2F1 | GPHN | 0.039527942 |
| E2F2 | CDC45 | 30.51059106 |
| E2F2 | DIAPH3 | 21.01626938 |
| E2F2 | ASF1B | 17.35923227 |
| E2F2 | RRM2 | 9.359791501 |
| E2F2 | PSMC3IP | 5.779009126 |
| E2F2 | UHRF1 | 5.718275927 |
| E2F2 | DBF4 | 4.954041927 |
| E2F2 | CKAP2L | 4.137999428 |
| E2F2 | LSM4 | 3.842907617 |
| E2F2 | SPAG5 | 3.712913138 |
| E2F2 | ESCO2 | 3.563829249 |
| E2F2 | UBE2T | 3.134415605 |
| E2F2 | PSD | 3.013356635 |
| E2F2 | MCM7 | 2.648821381 |
| E2F2 | KIF11 | 2.027644721 |
| E2F2 | ENHO | 1.937400914 |
| E2F2 | KIF4A | 1.905070392 |
| E2F2 | COMMD4 | 1.802907137 |
| E2F2 | DYNLT1 | 1.773064844 |
| E2F2 | ALG10B | 1.618645899 |
| E2F2 | CDC6 | 1.401454207 |
| E2F2 | AP3M2 | 1.255966674 |
| E2F2 | CDK4 | 1.216167506 |
| E2F2 | PLK1 | 1.15583182 |
| E2F2 | BARD1 | 1.148757284 |
| E2F2 | XRCC4 | 1.084941034 |
| E2F2 | E2F2 | 1 |
| E2F2 | CD320 | 0.969374616 |
| E2F2 | PTPN23 | 0.902691852 |
| E2F2 | FNBP1L | 0.871559955 |
| E2F2 | PGP | 0.792794616 |
| E2F2 | ASH2L | 0.741185934 |
| E2F2 | MTCH2 | 0.722808524 |
| E2F2 | PA2G4 | 0.693546969 |
| E2F2 | MZT1 | 0.665310632 |
| E2F2 | MZT2A | 0.65504218 |
| E2F2 | NR2C2AP | 0.613773046 |
| E2F2 | RECQL4 | 0.613646562 |
| E2F2 | FANCD2 | 0.586779068 |
| E2F2 | PCNA | 0.577789135 |
| E2F2 | HUS1 | 0.549455009 |
| E2F2 | TOMM40L | 0.516679019 |
| E2F2 | OIP5 | 0.499215768 |
| E2F2 | NXN | 0.498048601 |
| E2F2 | HIST1H4C | 0.466354069 |
| E2F2 | PCDH19 | 0.449745776 |
| E2F2 | ZNF446 | 0.444929759 |
| E2F2 | COQ2 | 0.411434086 |
| E2F2 | MLLT6 | 0.408243654 |
| E2F2 | TBC1D5 | 0.391245741 |
| E2F2 | TTK | 0.388844248 |
| E2F2 | LSM2 | 0.384441831 |
| E2F2 | APOO | 0.355467776 |
| E2F2 | SRR | 0.34897861 |
| E2F2 | CDKN2C | 0.341218224 |
| E2F2 | SDR39U1 | 0.309162825 |
| E2F2 | NR2C2 | 0.300252391 |
| E2F2 | NUDT16L1 | 0.294651861 |
| E2F2 | TGFBRAP1 | 0.291924202 |
| E2F2 | BID | 0.284810667 |
| E2F2 | AGPAT3 | 0.26742413 |
| E2F2 | SAP30 | 0.240873912 |
| E2F2 | TOMM40 | 0.232741816 |
| E2F2 | SAC3D1 | 0.225659395 |
| E2F2 | AMZ2 | 0.222420378 |
| E2F2 | DOCK10 | 0.215648425 |
| E2F2 | C2CD4B | 0.211806593 |
| E2F2 | FAM53B | 0.210912473 |
| E2F2 | MTAP | 0.181242354 |
| E2F2 | PTPDC1 | 0.176867345 |
| E2F2 | ZNF669 | 0.175994075 |
| E2F2 | PFKP | 0.174776295 |
| E2F2 | WDR34 | 0.167646764 |
| E2F2 | HYAL2 | 0.163235271 |
| E2F2 | NKIRAS2 | 0.161918657 |
| E2F2 | TFDP1 | 0.161086562 |
| E2F2 | MBD1 | 0.160712231 |
| E2F2 | GNB5 | 0.157006372 |
| E2F2 | GMNN | 0.149200969 |
| E2F2 | ISY1 | 0.147965584 |
| E2F2 | VPS37B | 0.143988431 |
| E2F2 | PUSL1 | 0.143024813 |
| E2F2 | TOMM5 | 0.139558316 |
| E2F2 | SHMT1 | 0.111520567 |
| E2F2 | TDP1 | 0.111395467 |
| E2F2 | TMEM100 | 0.105124677 |
| E2F2 | ORC5 | 0.1050172 |
| E2F2 | NNT | 0.089263955 |
| E2F2 | GTPBP8 | 0.067189211 |
| E2F2 | RALGAPA2 | 0.022304498 |
| E2F3 | PGRMC1 | 7.219486058 |
| E2F3 | PDLIM7 | 5.560243856 |
| E2F3 | MAGT1 | 4.937140955 |
| E2F3 | LIMCH1 | 4.336190396 |
| E2F3 | PTTG1 | 3.612039854 |
| E2F3 | POLR2J | 2.675351842 |
| E2F3 | GLRX3 | 1.813194053 |
| E2F3 | NDUFAF3 | 1.797080274 |
| E2F3 | SREK1 | 1.68830768 |
| E2F3 | YIF1A | 1.614122416 |
| E2F3 | VDAC2 | 1.499878905 |
| E2F3 | NUDT11 | 1.497234861 |
| E2F3 | TMTC2 | 1.442085291 |
| E2F3 | CTSS | 1.405741244 |
| E2F3 | MTMR10 | 1.380949004 |
| E2F3 | DLAT | 1.313344223 |
| E2F3 | DGUOK | 1.250544194 |
| E2F3 | NPTX2 | 1.247124672 |
| E2F3 | EDEM1 | 1.182916035 |
| E2F3 | LDLRAP1 | 1.179944057 |
| E2F3 | TAF11 | 1.165557399 |
| E2F3 | RCCD1 | 1.142820802 |
| E2F3 | CLSPN | 1.076014642 |
| E2F3 | PJA1 | 1.071396527 |
| E2F3 | BRWD1 | 1.051050252 |
| E2F3 | ATIC | 1.035435315 |
| E2F3 | RHOBTB1 | 1.003993264 |
| E2F3 | E2F3 | 1 |
| E2F3 | AHCY | 0.996790652 |
| E2F3 | KIAA0355 | 0.951851796 |
| E2F3 | SENP6 | 0.931673243 |
| E2F3 | PPM1A | 0.903265601 |
| E2F3 | CORO7 | 0.884442036 |
| E2F3 | IFFO2 | 0.87505098 |
| E2F3 | PLOD3 | 0.863980705 |
| E2F3 | CNPY4 | 0.845566022 |
| E2F3 | PELP1 | 0.704595192 |
| E2F3 | TPD52L1 | 0.699855765 |
| E2F3 | FAM122B | 0.674792834 |
| E2F3 | WTAP | 0.621013845 |
| E2F3 | EMCN | 0.538875107 |
| E2F3 | IKBIP | 0.514547145 |
| E2F3 | HOXB7 | 0.508617017 |
| E2F4 | CWC15 | 5.017994342 |
| E2F4 | DHX34 | 3.43011168 |
| E2F4 | ZC3H7B | 2.427289617 |
| E2F4 | EXD2 | 2.34519215 |
| E2F4 | KLHL15 | 2.224104047 |
| E2F4 | ZNF83 | 2.203714686 |
| E2F4 | ADCY4 | 2.144604307 |
| E2F4 | ZNF217 | 2.103023459 |
| E2F4 | GPX1 | 1.923596196 |
| E2F4 | EXTL2 | 1.858446322 |
| E2F4 | SMAD4 | 1.835321782 |
| E2F4 | DLC1 | 1.82145479 |
| E2F4 | NSUN6 | 1.697972968 |
| E2F4 | IPO8 | 1.624089696 |
| E2F4 | GOLGA8A | 1.58458544 |
| E2F4 | MLF2 | 1.540770895 |
| E2F4 | GPRASP1 | 1.511901885 |
| E2F4 | ZNF865 | 1.510043138 |
| E2F4 | NOVA1 | 1.473877882 |
| E2F4 | DCAF16 | 1.389253351 |
| E2F4 | PARD3B | 1.381101125 |
| E2F4 | SETBP1 | 1.368844001 |
| E2F4 | TBP | 1.355489073 |
| E2F4 | GOLGA8B | 1.322551844 |
| E2F4 | POFUT2 | 1.318752027 |
| E2F4 | DZIP1L | 1.304249394 |
| E2F4 | PTDSS2 | 1.282521819 |
| E2F4 | CCNI | 1.258618834 |
| E2F4 | TNPO3 | 1.234251443 |
| E2F4 | DEDD | 1.166204114 |
| E2F4 | SQSTM1 | 1.084845142 |
| E2F4 | CLPTM1 | 1.062789 |
| E2F4 | ZNF436 | 1.056504539 |
| E2F4 | HSPA4 | 1.054825087 |
| E2F4 | TBX18 | 1.012725568 |
| E2F4 | STOML1 | 1.003434693 |
| E2F4 | E2F4 | 1 |
| E2F4 | TSC22D4 | 0.962516915 |
| E2F4 | PAPD7 | 0.960171456 |
| E2F4 | GTF2A2 | 0.956208784 |
| E2F4 | CNOT1 | 0.915203084 |
| E2F4 | ZNF462 | 0.883899147 |
| E2F4 | TRIM4 | 0.848613624 |
| E2F4 | CDC14B | 0.806089157 |
| E2F4 | SH3BGRL | 0.801661805 |
| E2F4 | TSGA10 | 0.787477211 |
| E2F4 | KLF10 | 0.77109548 |
| E2F4 | SEMA3A | 0.721697528 |
| E2F4 | POLR1B | 0.717349466 |
| E2F4 | RPL37 | 0.715006997 |
| E2F4 | PDGFRA | 0.699146236 |
| E2F4 | RPS27A | 0.681176079 |
| E2F4 | AUTS2 | 0.669526187 |
| E2F4 | SRP54 | 0.666818016 |
| E2F4 | TNRC6B | 0.653048141 |
| E2F4 | EIF3I | 0.639601251 |
| E2F4 | RPL37A | 0.632681658 |
| E2F4 | MPZL1 | 0.632070415 |
| E2F4 | CDK5RAP1 | 0.625438253 |
| E2F4 | CLDND1 | 0.59948396 |
| E2F4 | NCAPD2 | 0.594727797 |
| E2F4 | DCP2 | 0.594453919 |
| E2F4 | DCTN3 | 0.588762564 |
| E2F4 | POLR2J2 | 0.587054145 |
| E2F4 | JMJD6 | 0.556529874 |
| E2F4 | YWHAB | 0.555147748 |
| E2F4 | NAA38 | 0.542608854 |
| E2F4 | HIF1AN | 0.542594976 |
| E2F4 | WDR61 | 0.534835451 |
| E2F4 | PHC2 | 0.530969908 |
| E2F4 | THOC5 | 0.530932972 |
| E2F4 | IFNGR1 | 0.529114409 |
| E2F4 | TSPAN14 | 0.528529656 |
| E2F4 | PIGT | 0.518460002 |
| E2F4 | ADAM17 | 0.513452389 |
| E2F4 | SNX17 | 0.506061258 |
| E2F4 | CDK13 | 0.501973191 |
| E2F4 | GTF3C1 | 0.4987188 |
| E2F4 | PCBP2 | 0.480804197 |
| E2F4 | TNFRSF1A | 0.478193726 |
| E2F4 | ISCU | 0.458629635 |
| E2F4 | BLZF1 | 0.455835763 |
| E2F4 | GTPBP2 | 0.455099052 |
| E2F4 | LEPROT | 0.454388065 |
| E2F4 | DCAF10 | 0.451474848 |
| E2F4 | ADARB1 | 0.449225693 |
| E2F4 | FBXO38 | 0.442394842 |
| E2F6 | CRY1 | 5.737246509 |
| E2F6 | SLC1A4 | 5.302048425 |
| E2F6 | MAN2B2 | 4.068205631 |
| E2F6 | CALCOCO1 | 3.839923981 |
| E2F6 | PRPF6 | 3.674711956 |
| E2F6 | CNTN1 | 3.532131133 |
| E2F6 | TMEM64 | 3.471842977 |
| E2F6 | MAGI3 | 3.224318742 |
| E2F6 | PRKAR1A | 3.218374565 |
| E2F6 | AKIRIN2 | 3.183340083 |
| E2F6 | FBXL14 | 3.100797695 |
| E2F6 | ANXA7 | 3.068406721 |
| E2F6 | FBXW11 | 3.0323019 |
| E2F6 | RALY | 2.87899328 |
| E2F6 | PRDX4 | 2.594717243 |
| E2F6 | WDR75 | 2.502137898 |
| E2F6 | BMP1 | 2.484194766 |
| E2F6 | MALAT1 | 2.281539623 |
| E2F6 | HSPB6 | 2.221458876 |
| E2F6 | GPX2 | 2.160751022 |
| E2F6 | NADSYN1 | 1.962108061 |
| E2F6 | SRRT | 1.920172574 |
| E2F6 | QARS | 1.880247326 |
| E2F6 | SNAI1 | 1.85565009 |
| E2F6 | FNBP1L | 1.822066731 |
| E2F6 | ZNF260 | 1.818680538 |
| E2F6 | ORC4 | 1.768144894 |
| E2F6 | SEC24C | 1.734385729 |
| E2F6 | ORMDL2 | 1.721307664 |
| E2F6 | UGDH | 1.668908413 |
| E2F6 | NFASC | 1.645472854 |
| E2F6 | PSMG2 | 1.620168573 |
| E2F6 | PAK3 | 1.5962838 |
| E2F6 | STK25 | 1.58830379 |
| E2F6 | LANCL1 | 1.482883816 |
| E2F6 | IVNS1ABP | 1.469315454 |
| E2F6 | STIM2 | 1.435268372 |
| E2F6 | ARMC1 | 1.428652996 |
| E2F6 | SDF4 | 1.424850597 |
| E2F6 | SPOCK1 | 1.353401239 |
| E2F6 | NRXN2 | 1.251325092 |
| E2F6 | FRMD6 | 1.244625706 |
| E2F6 | IGFBP3 | 1.232063496 |
| E2F6 | CITED2 | 1.231876275 |
| E2F6 | GSR | 1.21294493 |
| E2F6 | C18orf32 | 1.16664632 |
| E2F6 | NTRK2 | 1.108188023 |
| E2F6 | ADIPOR2 | 1.048762081 |
| E2F6 | E2F6 | 1 |
| E2F6 | RBM7 | 0.979143232 |
| E2F6 | KDELR1 | 0.97886322 |
| E2F6 | PPP2R5C | 0.977089735 |
| E2F6 | SF3B2 | 0.928867033 |
| E2F6 | DLST | 0.916416685 |
| E2F6 | DOHH | 0.883522743 |
| E2F6 | RPL23A | 0.856012981 |
| E2F6 | RGS10 | 0.848145124 |
| E2F6 | SPAST | 0.836527144 |
| E2F6 | CD2BP2 | 0.824856758 |
| E2F6 | LMBR1 | 0.776359088 |
| E2F6 | HSPA13 | 0.774197077 |
| E2F6 | SMAD1 | 0.768580236 |
| E2F6 | TMEM167B | 0.767294712 |
| E2F6 | BZW1 | 0.714982978 |
| E2F6 | NBPF1 | 0.677873683 |
| E2F6 | RP9P | 0.673308251 |
| E2F6 | RRBP1 | 0.655923961 |
| E2F6 | FBXO9 | 0.612085267 |
| E2F6 | CITED4 | 0.599865127 |
| E2F6 | CAST | 0.571602347 |
| E2F6 | PTOV1 | 0.5629863 |
| E2F6 | ID4 | 0.553909867 |
| E2F6 | ITM2A | 0.550181245 |
| E2F6 | TMEM30A | 0.506057475 |
| E2F6 | RPN1 | 0.503828916 |
| E2F6 | ARHGEF40 | 0.479791784 |
| E2F6 | NCKAP1 | 0.475617021 |
| E2F6 | EFNA1 | 0.473801456 |
| E2F6 | DYRK3 | 0.46856922 |
| E2F6 | ALG5 | 0.46620239 |
| E2F6 | DNAJC1 | 0.461063995 |
| E2F7 | TACC3 | 24.68564469 |
| E2F7 | CEP55 | 20.0607552 |
| E2F7 | NUSAP1 | 17.81056874 |
| E2F7 | CDK1 | 16.80756144 |
| E2F7 | RAD51AP1 | 14.79164644 |
| E2F7 | RRM2 | 14.18107969 |
| E2F7 | CDCA4 | 13.76306426 |
| E2F7 | TYMS | 12.80773974 |
| E2F7 | UBE2T | 12.38843801 |
| E2F7 | CKAP2L | 10.73202335 |
| E2F7 | NUF2 | 10.14204574 |
| E2F7 | NCAPD2 | 9.411310574 |
| E2F7 | ESCO2 | 9.094774787 |
| E2F7 | TROAP | 7.995807715 |
| E2F7 | TOP2A | 7.851694559 |
| E2F7 | HMGB2 | 7.801674482 |
| E2F7 | CDC20 | 7.660331939 |
| E2F7 | ATAD5 | 7.016843015 |
| E2F7 | CCNA2 | 6.906825217 |
| E2F7 | MSH2 | 6.84934134 |
| E2F7 | HIST1H4C | 6.488889994 |
| E2F7 | CENPN | 5.638412027 |
| E2F7 | CENPF | 5.497023286 |
| E2F7 | ARHGAP11A | 5.163110244 |
| E2F7 | C21orf58 | 5.124321921 |
| E2F7 | PKMYT1 | 4.98533895 |
| E2F7 | PBK | 4.916569973 |
| E2F7 | LMNB1 | 4.82880395 |
| E2F7 | FAM111A | 4.157025217 |
| E2F7 | PRC1 | 4.123709298 |
| E2F7 | DIAPH3 | 3.92316732 |
| E2F7 | CDCA2 | 3.521437359 |
| E2F7 | PSRC1 | 3.40670794 |
| E2F7 | ANLN | 3.254781719 |
| E2F7 | MCM10 | 3.172588547 |
| E2F7 | POLD3 | 3.161435903 |
| E2F7 | TMEM106C | 3.111794451 |
| E2F7 | MND1 | 3.109349673 |
| E2F7 | FAM111B | 3.060812482 |
| E2F7 | RRM1 | 2.938700249 |
| E2F7 | CLSPN | 2.893591097 |
| E2F7 | PEG10 | 2.888036865 |
| E2F7 | OLFM1 | 2.881114265 |
| E2F7 | AURKB | 2.845053412 |
| E2F7 | CENPA | 2.762478211 |
| E2F7 | CDC45 | 2.751509461 |
| E2F7 | MYBL2 | 2.688195066 |
| E2F7 | PRKAR1B | 2.647485615 |
| E2F7 | HJURP | 2.597792902 |
| E2F7 | CENPO | 2.541352214 |
| E2F7 | HIST1H1A | 2.532927784 |
| E2F7 | UBE2S | 2.514119071 |
| E2F7 | RACGAP1 | 2.389295409 |
| E2F7 | HMMR | 2.340075664 |
| E2F7 | MELK | 2.321367917 |
| E2F7 | FAM83D | 2.271883909 |
| E2F7 | NDC80 | 2.204345027 |
| E2F7 | SHCBP1 | 2.169146751 |
| E2F7 | DTYMK | 2.163130462 |
| E2F7 | CCDC34 | 2.071685709 |
| E2F7 | FBXO5 | 1.88569034 |
| E2F7 | TK1 | 1.880284451 |
| E2F7 | MZT1 | 1.870706767 |
| E2F7 | NCAPH2 | 1.824984017 |
| E2F7 | SMC4 | 1.818269646 |
| E2F7 | BUB3 | 1.675350943 |
| E2F7 | TCF19 | 1.648510896 |
| E2F7 | OIP5 | 1.586987948 |
| E2F7 | SAC3D1 | 1.582201308 |
| E2F7 | CKAP5 | 1.560111654 |
| E2F7 | R3HCC1 | 1.518009673 |
| E2F7 | FEN1 | 1.460537783 |
| E2F7 | TIMELESS | 1.341649251 |
| E2F7 | SMC1A | 1.047769568 |
| E2F7 | E2F7 | 1 |
| E2F7 | DNMT1 | 0.97906234 |
| E2F7 | CDC6 | 0.969164985 |
| E2F7 | SFRP5 | 0.967457132 |
| E2F7 | GINS1 | 0.963989449 |
| E2F7 | H2AFV | 0.95230021 |
| E2F7 | TTK | 0.944930484 |
| E2F7 | E2F8 | 0.921234862 |
| E2F7 | MBNL3 | 0.91933101 |
| E2F7 | CCNB1 | 0.912612218 |
| E2F7 | GNG2 | 0.901718411 |
| E2F7 | PIN1 | 0.878528456 |
| E2F7 | CYB5R2 | 0.85955489 |
| E2F7 | CHAC2 | 0.82777945 |
| E2F7 | DCAF17 | 0.79228362 |
| E2F7 | LSM4 | 0.770357539 |
| E2F7 | CALM3 | 0.76069249 |
| E2F7 | SMC2 | 0.749192402 |
| E2F7 | SLC25A1 | 0.737470282 |
| E2F7 | PSMC3IP | 0.728090381 |
| E2F7 | PHF19 | 0.721131191 |
| E2F7 | ARL6IP6 | 0.704309904 |
| E2F7 | KIF4A | 0.652841309 |
| E2F7 | SAP30 | 0.61193993 |
| E2F7 | ASRGL1 | 0.589046394 |
| E2F7 | PLK4 | 0.581601556 |
| E2F7 | SSRP1 | 0.566086965 |
| E2F7 | CDK2 | 0.559398481 |
| E2F7 | POP4 | 0.555891574 |
| E2F7 | ZNF714 | 0.547835417 |
| E2F7 | ACADM | 0.546594177 |
| E2F7 | TMSB15A | 0.539859521 |
| E2F7 | COX10 | 0.520339582 |
| E2F7 | YEATS4 | 0.506268126 |
| E2F7 | HDGF | 0.481556769 |
| E2F7 | NIPA2 | 0.480495276 |
| E2F7 | HAUS3 | 0.475455775 |
| E2F7 | ZNF548 | 0.467432627 |
| E2F7 | HIST1H3B | 0.466924687 |
| E2F7 | SLX4 | 0.466418379 |
| E2F7 | MEIS3 | 0.456965522 |
| E2F7 | ANKS1A | 0.430074301 |
| E2F7 | KCNA5 | 0.424835583 |
| E2F7 | GMNN | 0.417664419 |
| E2F7 | LIN9 | 0.391850106 |
| E2F7 | USP13 | 0.383383282 |
| E2F7 | KIF18A | 0.375703345 |
| E2F7 | NUP35 | 0.368100232 |
| E2F7 | TLK2 | 0.356005374 |
| E2F7 | RANGAP1 | 0.348442757 |
| E2F7 | DHX37 | 0.337352858 |
| E2F7 | TMPO | 0.334384255 |
| E2F7 | PHC1 | 0.329373466 |
| E2F7 | HIST1H1B | 0.32853108 |
| E2F7 | CNKSR3 | 0.32540661 |
| E2F7 | HIST1H3C | 0.312425885 |
| E2F7 | CDT1 | 0.306985713 |
| E2F7 | RRAGD | 0.298203868 |
| E2F7 | FUZ | 0.295566029 |
| E2F7 | HIST1H4B | 0.293154219 |
| E2F7 | USF1 | 0.272481309 |
| E2F7 | APLP1 | 0.261106387 |
| E2F7 | GNL1 | 0.247578072 |
| E2F7 | CDKN2C | 0.244594704 |
| E2F7 | THOC6 | 0.221647951 |
| E2F7 | TRIM59 | 0.217997199 |
| E2F7 | PSD3 | 0.21429146 |
| E2F7 | GMCL1 | 0.206782084 |
| E2F7 | MAPRE1 | 0.20390258 |
| E2F7 | SYNE2 | 0.201853892 |
| E2F7 | MRPL47 | 0.199568166 |
| E2F7 | FANCD2 | 0.186696831 |
| E2F7 | HIP1 | 0.183053904 |
| E2F7 | PHGDH | 0.176067513 |
| E2F7 | HUS1 | 0.168673599 |
| E2F7 | RBBP8 | 0.158195432 |
| E2F7 | KDELC1 | 0.156121705 |
| E2F7 | RECQL4 | 0.145147094 |
| E2F7 | POC5 | 0.141832858 |
| E2F7 | NRM | 0.138460448 |
| E2F7 | INCENP | 0.137496175 |
| E2F7 | EZH1 | 0.137172001 |
| E2F7 | LPGAT1 | 0.124603003 |
| E2F7 | TYRO3 | 0.121602305 |
| E2F7 | NUP188 | 0.119907849 |
| E2F7 | UMPS | 0.118461397 |
| E2F7 | DGCR8 | 0.115065088 |
| E2F7 | SKA1 | 0.111866281 |
| E2F7 | RFC4 | 0.111143254 |
| E2F7 | CFHR1 | 0.099730411 |
| E2F7 | ATP11A | 0.082674754 |
| E2F7 | PAK4 | 0.081708642 |
| E2F7 | IKBKB | 0.077950407 |
| E2F7 | SYT11 | 0.077332027 |
| E2F7 | GJC1 | 0.068990992 |
| E2F7 | TMEM116 | 0.058364479 |
| E2F7 | CIT | 0.056787731 |
| E2F7 | FANCG | 0.054744004 |
| E2F7 | NEURL1B | 0.051344826 |
| E2F7 | LIG3 | 0.049700388 |
| E2F7 | HYLS1 | 0.028719117 |
| E2F8 | CDCA5 | 18.69414857 |
| E2F8 | MKI67 | 15.16489519 |
| E2F8 | FAM111B | 14.10960001 |
| E2F8 | MYBL2 | 10.68243321 |
| E2F8 | MND1 | 9.642222142 |
| E2F8 | PAQR4 | 8.648306408 |
| E2F8 | LMNB1 | 8.146543702 |
| E2F8 | PKMYT1 | 7.543483519 |
| E2F8 | KIFC1 | 6.985018382 |
| E2F8 | ZWINT | 6.095340217 |
| E2F8 | CDK4 | 6.083535823 |
| E2F8 | TYMS | 5.998659974 |
| E2F8 | CDC45 | 5.788542028 |
| E2F8 | CENPN | 5.565227191 |
| E2F8 | EZH2 | 5.319233606 |
| E2F8 | SPTLC3 | 4.967339437 |
| E2F8 | MELK | 4.858925008 |
| E2F8 | HECTD1 | 4.728045002 |
| E2F8 | RRM1 | 4.606909002 |
| E2F8 | NUSAP1 | 3.894155832 |
| E2F8 | FBXO5 | 3.822381848 |
| E2F8 | HIST1H4C | 3.57616251 |
| E2F8 | HDGF | 3.527923319 |
| E2F8 | TLK2 | 3.254159344 |
| E2F8 | GLRX5 | 3.002997741 |
| E2F8 | E2F7 | 2.989963355 |
| E2F8 | CCNA2 | 2.837154511 |
| E2F8 | ATAD5 | 2.820776647 |
| E2F8 | NCAPG | 2.589440752 |
| E2F8 | PRKDC | 2.552755537 |
| E2F8 | MCM10 | 2.526024132 |
| E2F8 | CENPH | 2.465355932 |
| E2F8 | MORN2 | 2.390640557 |
| E2F8 | GINS4 | 2.352301252 |
| E2F8 | RNASEH2C | 2.286366971 |
| E2F8 | HDDC3 | 2.26603954 |
| E2F8 | PALLD | 2.257284143 |
| E2F8 | NCAPH | 2.245164399 |
| E2F8 | SERPINE1 | 2.192020891 |
| E2F8 | SNRPA1 | 2.089486334 |
| E2F8 | HIST1H3B | 2.033342351 |
| E2F8 | ANAPC7 | 2.032793447 |
| E2F8 | NUF2 | 2.030459918 |
| E2F8 | PTS | 2.018900563 |
| E2F8 | MRPS21 | 2.011916467 |
| E2F8 | SMC2 | 1.974310918 |
| E2F8 | KIF15 | 1.833379383 |
| E2F8 | COQ10B | 1.774491706 |
| E2F8 | LIG1 | 1.771980141 |
| E2F8 | ASF1B | 1.751001302 |
| E2F8 | HIST1H1B | 1.739988845 |
| E2F8 | UBE2S | 1.733491595 |
| E2F8 | ORC1 | 1.726828304 |
| E2F8 | VDAC1 | 1.71839595 |
| E2F8 | FXYD6 | 1.622381171 |
| E2F8 | DHFR | 1.579292512 |
| E2F8 | MAD2L2 | 1.561831624 |
| E2F8 | GGH | 1.561340493 |
| E2F8 | CDC6 | 1.531228295 |
| E2F8 | PRIM1 | 1.520533314 |
| E2F8 | H2AFZ | 1.476943681 |
| E2F8 | LAGE3 | 1.400588038 |
| E2F8 | MRPS33 | 1.379250921 |
| E2F8 | PBK | 1.336833373 |
| E2F8 | PSMA1 | 1.321982374 |
| E2F8 | RANBP1 | 1.287815442 |
| E2F8 | ZC3HAV1L | 1.228910755 |
| E2F8 | SP100 | 1.222619944 |
| E2F8 | KPNA2 | 1.213208376 |
| E2F8 | CHD3 | 1.18303383 |
| E2F8 | KIF26A | 1.155874067 |
| E2F8 | HMMR | 1.121572933 |
| E2F8 | KDELR3 | 1.119073651 |
| E2F8 | CDK7 | 1.117828832 |
| E2F8 | POC1A | 1.087494589 |
| E2F8 | NAA50 | 1.030649364 |
| E2F8 | E2F8 | 1 |
| E2F8 | YKT6 | 0.983246373 |
| E2F8 | PSIP1 | 0.970629509 |
| E2F8 | UQCRC1 | 0.961536627 |
| E2F8 | RBM17 | 0.946071656 |
| E2F8 | DEK | 0.922003583 |
| E2F8 | OTUD5 | 0.902704209 |
| E2F8 | CKAP2L | 0.89631713 |
| E2F8 | ACOT7 | 0.886358273 |
| E2F8 | ANP32B | 0.87888964 |
| E2F8 | KIF4A | 0.878405504 |
| E2F8 | MOB2 | 0.857748237 |
| E2F8 | DNAJC9 | 0.741523469 |
| E2F8 | FOXM1 | 0.735480676 |
| E2F8 | MYH9 | 0.73491518 |
| E2F8 | MESP1 | 0.725453265 |
| E2F8 | PRC1 | 0.690823594 |
| E2F8 | TNIP1 | 0.665094923 |
| E2F8 | ICMT | 0.663247176 |
| E2F8 | TFB1M | 0.644815896 |
| E2F8 | COL11A1 | 0.642963744 |
| E2F8 | FANCG | 0.641128059 |
| E2F8 | FUS | 0.620323705 |
| E2F8 | POLA2 | 0.608042262 |
| E2F8 | HJURP | 0.595810588 |
| E2F8 | PHKB | 0.588227228 |
| E2F8 | NRM | 0.548751902 |
| E2F8 | RBBP7 | 0.545832887 |
| E2F8 | TCF19 | 0.527246654 |
| E2F8 | CENPM | 0.52121837 |
| E2F8 | ABHD3 | 0.50631376 |
| E2F8 | AMZ2 | 0.503954599 |
| E2F8 | RER1 | 0.481260156 |
| E2F8 | BUB3 | 0.476032136 |
| E2F8 | GNAO1 | 0.475095624 |
| E2F8 | RPL22L1 | 0.441950556 |
| E2F8 | SAC3D1 | 0.40924763 |
| E2F8 | C21orf58 | 0.401106343 |
| E2F8 | PPP2R5A | 0.364508099 |
| E2F8 | GRPEL2 | 0.362448176 |
| E2F8 | CEP70 | 0.333944987 |
| E2F8 | N4BP2 | 0.274932743 |
| E2F8 | DDX55 | 0.262373451 |
| E2F8 | PDIK1L | 0.251022255 |
| E2F8 | GMNN | 0.249220466 |
| E2F8 | ECT2 | 0.245201287 |
| E2F8 | TYRO3 | 0.24278558 |
| E2F8 | POLD3 | 0.230202492 |
| E2F8 | C1QTNF2 | 0.229224518 |
| E2F8 | ANKS1A | 0.225036878 |
| E2F8 | MCM2 | 0.221557285 |
| E2F8 | MCM3 | 0.221275136 |
| E2F8 | GALNT7 | 0.217095423 |
| E2F8 | MFAP1 | 0.21408453 |
| E2F8 | LIN9 | 0.21282054 |
| E2F8 | CCND2 | 0.203649322 |
| E2F8 | TRIM8 | 0.188625852 |
| E2F8 | RPP25 | 0.177793482 |
| E2F8 | PCNA | 0.176197063 |
| E2F8 | GMCL1 | 0.175305214 |
| E2F8 | APIP | 0.170797246 |
| E2F8 | BRD7 | 0.166260773 |
| E2F8 | RMI1 | 0.160042884 |
| E2F8 | SEPSECS | 0.159414935 |
| E2F8 | CHAC2 | 0.141444866 |
| E2F8 | UCKL1 | 0.140339046 |
| E2F8 | ADAMTS9 | 0.135809037 |
| E2F8 | ASRGL1 | 0.130186689 |
| E2F8 | AKAP10 | 0.126598146 |
| E2F8 | MEIS3 | 0.126404112 |
| E2F8 | CSE1L | 0.125295659 |
| E2F8 | TTLL7 | 0.120951451 |
| E2F8 | MYOCD | 0.111800999 |
| E2F8 | UNG | 0.110968382 |
| E2F8 | TIMELESS | 0.107118497 |
| E2F8 | SRR | 0.103593498 |
| E2F8 | KCNA5 | 0.102621661 |
| E2F8 | GRK6 | 0.102258706 |
| E2F8 | CENPO | 0.093238641 |
| E2F8 | ARL6IP6 | 0.084310474 |
| E2F8 | IKBKB | 0.079825719 |
| E2F8 | SKA1 | 0.055249713 |
| E2F8 | L3MBTL2 | 0.050761453 |
| E2F8 | C1QL1 | 0.047139679 |
| E4F1 | ADM | 1.930927341 |
| E4F1 | KCNK15 | 0.957606996 |
| E4F1 | EMX2 | 0.919439467 |
| E4F1 | AHCYL2 | 0.816386697 |
| E4F1 | LYPLAL1 | 0.774504493 |
| E4F1 | TRIB1 | 0.643896145 |
| E4F1 | NFKB2 | 0.546737808 |
| E4F1 | NAA50 | 0.54098542 |
| E4F1 | CYCS | 0.538055236 |
| EBF1 | CXCL12 | 29.6945343 |
| EBF1 | FBLN2 | 11.79104901 |
| EBF1 | SNED1 | 10.98289568 |
| EBF1 | IL6ST | 6.923907832 |
| EBF1 | DCLK1 | 5.952798873 |
| EBF1 | ZEB2 | 4.470049302 |
| EBF1 | CACNB4 | 2.684151049 |
| EBF1 | SMOC1 | 2.432716919 |
| EBF1 | SACS | 2.193646453 |
| EBF1 | DCAF12 | 2.159452653 |
| EBF1 | FBXW4 | 2.153520634 |
| EBF1 | RAD54L2 | 1.834317999 |
| EBF1 | GRIA3 | 1.69690884 |
| EBF1 | HS6ST1 | 1.624049304 |
| EBF1 | PAPD7 | 1.570050246 |
| EBF1 | NMB | 1.498316153 |
| EBF1 | ADCY3 | 1.49440779 |
| EBF1 | SYBU | 1.476514153 |
| EBF1 | PER2 | 1.253394744 |
| EBF1 | CD34 | 0.991181497 |
| EBF1 | RARA | 0.876469969 |
| EBF1 | FOXP4 | 0.833038336 |
| ECSIT | KLHL15 | 5.057519636 |
| ECSIT | NCOA1 | 2.83261733 |
| ECSIT | ERCC3 | 2.643070105 |
| ECSIT | DOK6 | 2.208523811 |
| ECSIT | CGNL1 | 1.89519067 |
| ECSIT | NAALADL2 | 1.451462981 |
| ECSIT | TRIO | 1.218544227 |
| ECSIT | MYO1B | 1.20324838 |
| ECSIT | RPL23P8 | 0.824259184 |
| EGR2 | TIMP3 | 12.19963567 |
| EGR2 | OLFML2A | 6.1390712 |
| EGR2 | EGR1 | 5.550539663 |
| EGR2 | ITM2A | 5.315675006 |
| EGR2 | GSN | 3.023508356 |
| EGR2 | RPL29 | 2.85681084 |
| EGR2 | COL12A1 | 2.464066139 |
| EGR2 | PFN1 | 2.291077091 |
| EGR2 | CTGF | 2.237900002 |
| EGR2 | PPP1R15A | 2.078346439 |
| EGR2 | FAM20C | 1.80351217 |
| EGR2 | RSF1 | 1.789910876 |
| EGR2 | ZFHX3 | 1.77554516 |
| EGR2 | SLC39A13 | 1.62658466 |
| EGR2 | SULF2 | 1.580572078 |
| EGR2 | IER2 | 1.572769469 |
| EGR2 | TWIST1 | 1.445358055 |
| EGR2 | BRD2 | 1.180362239 |
| EGR2 | JUN | 1.178056163 |
| EGR2 | VWA1 | 1.172682488 |
| EGR2 | GADD45B | 1.165798402 |
| EGR2 | ZMYND11 | 1.131511553 |
| EGR2 | GTF2E2 | 1.104387232 |
| EGR2 | NOB1 | 1.047579701 |
| EGR2 | EGR2 | 1 |
| EGR2 | DYRK1A | 0.789159441 |
| EGR2 | DKKL1 | 0.784827097 |
| EGR2 | CITED2 | 0.775173233 |
| EGR2 | APBB2 | 0.770179769 |
| EGR2 | SH3BP2 | 0.760690047 |
| EGR2 | MYL12B | 0.745844696 |
| EGR2 | RIMKLB | 0.723703159 |
| EGR2 | SOX4 | 0.692244888 |
| EGR2 | ATF3 | 0.690927406 |
| EGR2 | JUNB | 0.669059308 |
| EGR2 | HEXIM1 | 0.660470161 |
| EGR2 | PIK3R1 | 0.639194646 |
| EGR2 | USPL1 | 0.636426512 |
| EGR2 | AEBP1 | 0.627972838 |
| EGR2 | ID2 | 0.587557168 |
| EGR2 | BEND3 | 0.536928669 |
| EGR2 | CIRBP | 0.501941724 |
| EGR2 | TGFBR3 | 0.489560454 |
| EGR2 | SSPN | 0.464196425 |
| EGR2 | BCL9L | 0.459159709 |
| EGR2 | TIMP2 | 0.457003209 |
| EGR2 | GRIA3 | 0.445293968 |
| EGR2 | FOXO3 | 0.436610785 |
| EGR3 | CHURC1 | 6.133579698 |
| EGR3 | HES1 | 5.060623179 |
| EGR3 | PPP2R5D | 4.349054749 |
| EGR3 | NR4A2 | 3.507932109 |
| EGR3 | FOSB | 3.271584404 |
| EGR3 | BHLHE40 | 3.195059725 |
| EGR3 | EVI5 | 3.185813546 |
| EGR3 | SSH1 | 2.903542014 |
| EGR3 | MXD4 | 2.404274888 |
| EGR3 | PTPN14 | 2.280425337 |
| EGR3 | LYST | 2.245878844 |
| EGR3 | ZNF302 | 2.242670156 |
| EGR3 | PNP | 2.11439582 |
| EGR3 | DNAJA4 | 2.086215828 |
| EGR3 | TMEM19 | 1.940753063 |
| EGR3 | HEXIM1 | 1.86962154 |
| EGR3 | ELL | 1.83122717 |
| EGR3 | SEMA4C | 1.802359151 |
| EGR3 | FAM69A | 1.711590172 |
| EGR3 | STX7 | 1.663305547 |
| EGR3 | BZW2 | 1.643670332 |
| EGR3 | ZNF12 | 1.627128504 |
| EGR3 | TUBA1B | 1.389848333 |
| EGR3 | ZNF146 | 1.338381758 |
| EGR3 | GPR155 | 1.306563741 |
| EGR3 | MAN1C1 | 1.284476943 |
| EGR3 | CYGB | 1.158072767 |
| EGR3 | SYNGR1 | 1.140780206 |
| EGR3 | ETF1 | 1.124532956 |
| EGR3 | SMAD7 | 1.115631906 |
| EGR3 | GEM | 1.114714421 |
| EGR3 | EGR3 | 1 |
| EGR3 | SPATA9 | 0.936684453 |
| EGR3 | ZNF565 | 0.921877493 |
| EGR3 | FAM124A | 0.8047057 |
| EGR3 | ZNF665 | 0.754731435 |
| EGR3 | GMFG | 0.736558354 |
| EGR3 | RAB36 | 0.678542267 |
| EGR3 | RPP40 | 0.495196831 |
| EGR4 | SFN | 4.187383512 |
| EGR4 | PLOD1 | 2.846999945 |
| EGR4 | TRIM7 | 2.111512454 |
| EGR4 | CAMTA1 | 2.065293867 |
| EGR4 | TNIP2 | 1.995747475 |
| EGR4 | EIF4EBP2 | 1.515193597 |
| EGR4 | SUPT3H | 1.477046813 |
| EGR4 | DNAJA2 | 1.472412303 |
| EGR4 | TFAP2C | 1.443629654 |
| EGR4 | ARL4C | 1.421809529 |
| EGR4 | YWHAB | 1.260776772 |
| EGR4 | SERF2 | 1.084784381 |
| EGR4 | DUSP5 | 1.077392789 |
| EGR4 | YES1 | 1.027908038 |
| EGR4 | RNU4ATAC | 0.984155985 |
| EGR4 | KIAA0100 | 0.932556105 |
| EGR4 | VPS4A | 0.852173292 |
| EGR4 | ATOX1 | 0.813224367 |
| EGR4 | TPBG | 0.670371232 |
| EGR4 | GRINA | 0.571473846 |
| EGR4 | POLR1D | 0.558623299 |
| EGR4 | F2RL1 | 0.536893217 |
| EGR4 | PHTF2 | 0.533515338 |
| EGR4 | RPRD1A | 0.527895154 |
| EGR4 | SNX6 | 0.525029304 |
| EGR4 | ATP11B | 0.419086639 |
| EGR4 | MYO10 | 0.353275289 |
| EGR4 | THOP1 | 0.316135903 |
| EGR4 | ING2 | 0.289017544 |
| EGR4 | MORC4 | 0.286017815 |
| EGR4 | BAIAP2 | 0.274310754 |
| EGR4 | CNOT4 | 0.272971506 |
| EGR4 | DIP2A | 0.264815978 |
| EGR4 | GMPS | 0.260047798 |
| EGR4 | DEGS2 | 0.242035368 |
| EGR4 | SPINT2 | 0.237089559 |
| EGR4 | KIAA1328 | 0.223422017 |
| EGR4 | TTF2 | 0.208047589 |
| EGR4 | NPM3 | 0.186726342 |
| EGR4 | TMEM8B | 0.147281025 |
| EGR4 | ZNF48 | 0.094460247 |
| EGR4 | GM2A | 0.093622564 |
| EGR4 | FAM83G | 0.055937306 |
| EGR4 | SPESP1 | 0.032637996 |
| EGR4 | RND2 | 0.032426414 |
| EGR4 | MST1R | 0.023022332 |
| EGR4 | ALDH1L2 | 0.014406228 |
| EGR4 | STX17 | 0.007317846 |
| ELF1 | DCTN3 | 6.712147419 |
| ELF1 | HKR1 | 4.718281835 |
| ELF1 | POLG | 4.159721361 |
| ELF1 | ZNF623 | 3.709794287 |
| ELF1 | C7 | 3.381700553 |
| ELF1 | DENND1B | 3.374688315 |
| ELF1 | FHL1 | 3.335344467 |
| ELF1 | LMO2 | 3.330621132 |
| ELF1 | RPL15 | 2.920673525 |
| ELF1 | RPS27A | 2.679997843 |
| ELF1 | NUAK1 | 2.573479671 |
| ELF1 | SCARA5 | 2.516022494 |
| ELF1 | PPP2R5A | 2.382668809 |
| ELF1 | ADAMTS10 | 2.351660987 |
| ELF1 | POT1 | 2.290386553 |
| ELF1 | PHPT1 | 2.274059073 |
| ELF1 | TINF2 | 2.215257607 |
| ELF1 | RPL29 | 2.148446393 |
| ELF1 | CYR61 | 2.074201122 |
| ELF1 | FEZ1 | 2.019698934 |
| ELF1 | AVIL | 1.978003105 |
| ELF1 | HM13 | 1.953034116 |
| ELF1 | PCM1 | 1.950798413 |
| ELF1 | MYC | 1.932506369 |
| ELF1 | ZZEF1 | 1.914142149 |
| ELF1 | PPP1R3E | 1.912370187 |
| ELF1 | ATG16L1 | 1.897518929 |
| ELF1 | ARHGEF3 | 1.804286855 |
| ELF1 | ANXA1 | 1.790363904 |
| ELF1 | SON | 1.782506195 |
| ELF1 | NCOA2 | 1.771489291 |
| ELF1 | CSRNP1 | 1.76347862 |
| ELF1 | PPP2R3C | 1.697547177 |
| ELF1 | EED | 1.684791963 |
| ELF1 | C11orf96 | 1.678663427 |
| ELF1 | CWF19L2 | 1.645240894 |
| ELF1 | TIMM10 | 1.635179101 |
| ELF1 | CHCHD3 | 1.631044296 |
| ELF1 | ATP6V1E2 | 1.577816234 |
| ELF1 | SLC25A5 | 1.572836387 |
| ELF1 | DHX8 | 1.558349149 |
| ELF1 | TNKS1BP1 | 1.52821821 |
| ELF1 | PRR16 | 1.522022256 |
| ELF1 | AACS | 1.4956688 |
| ELF1 | NRXN2 | 1.492175749 |
| ELF1 | FAM3C | 1.491326517 |
| ELF1 | DDI2 | 1.49001712 |
| ELF1 | TMEM120A | 1.483227598 |
| ELF1 | SLC38A2 | 1.476419792 |
| ELF1 | CTDSPL | 1.455198439 |
| ELF1 | CDC34 | 1.441078978 |
| ELF1 | LPIN3 | 1.413294941 |
| ELF1 | CEP57L1 | 1.411272111 |
| ELF1 | AGPAT1 | 1.411075525 |
| ELF1 | AHCY | 1.4025161 |
| ELF1 | KIF18B | 1.394233925 |
| ELF1 | DAB2IP | 1.378422956 |
| ELF1 | BCL2L2 | 1.371852232 |
| ELF1 | HNRNPUL1 | 1.371015449 |
| ELF1 | CLCN4 | 1.35834741 |
| ELF1 | RASL11A | 1.354616582 |
| ELF1 | CCDC91 | 1.346329122 |
| ELF1 | NR1D2 | 1.342842797 |
| ELF1 | FOSL2 | 1.31542282 |
| ELF1 | SDCBP | 1.307771023 |
| ELF1 | HIVEP3 | 1.296421675 |
| ELF1 | GTF2F1 | 1.278664773 |
| ELF1 | RPL21 | 1.236865469 |
| ELF1 | INSIG2 | 1.234087231 |
| ELF1 | PRAF2 | 1.212752485 |
| ELF1 | SF1 | 1.200682104 |
| ELF1 | ZNF445 | 1.153971698 |
| ELF1 | STX12 | 1.151425663 |
| ELF1 | RSPRY1 | 1.150605811 |
| ELF1 | SVIL | 1.149698253 |
| ELF1 | KCTD5 | 1.131530835 |
| ELF1 | ITGA5 | 1.105335509 |
| ELF1 | RGMA | 1.09698516 |
| ELF1 | RPS20 | 1.091179406 |
| ELF1 | NBN | 1.056535577 |
| ELF1 | SREBF1 | 1.055293477 |
| ELF1 | MEGF9 | 1.053716713 |
| ELF1 | KCTD12 | 1.052790595 |
| ELF1 | GMPR2 | 1.017091384 |
| ELF1 | C1orf52 | 1.009386147 |
| ELF1 | ZFP36L1 | 1.008233018 |
| ELF1 | ELF1 | 1 |
| ELF1 | ORAI1 | 0.978985611 |
| ELF1 | ING2 | 0.976763892 |
| ELF1 | CDK1 | 0.974154865 |
| ELF1 | PCGF3 | 0.973581599 |
| ELF1 | TPM3 | 0.919351855 |
| ELF1 | RSL1D1 | 0.899995221 |
| ELF1 | IPO4 | 0.88034678 |
| ELF1 | TRPC6 | 0.879672493 |
| ELF1 | PIGT | 0.830567884 |
| ELF1 | GDF5 | 0.829338557 |
| ELF1 | LYRM7 | 0.828514881 |
| ELF1 | TAPBP | 0.818871998 |
| ELF1 | CTNNA1 | 0.818410107 |
| ELF1 | SSBP1 | 0.81058478 |
| ELF1 | FKBP5 | 0.808511392 |
| ELF1 | CAPZB | 0.795384876 |
| ELF1 | SPATA6 | 0.785873028 |
| ELF1 | SDF2 | 0.778319316 |
| ELF1 | RPL10 | 0.775960091 |
| ELF1 | OXR1 | 0.769318835 |
| ELF1 | FBLN2 | 0.758715709 |
| ELF1 | FTSJ1 | 0.756050736 |
| ELF1 | PLD3 | 0.742670887 |
| ELF1 | RPS8 | 0.725380206 |
| ELF1 | ANKRD28 | 0.724233304 |
| ELF1 | SH3KBP1 | 0.717733224 |
| ELF1 | RTF1 | 0.698927122 |
| ELF1 | HDX | 0.697179224 |
| ELF1 | ZNF680 | 0.691518342 |
| ELF1 | UGGT1 | 0.687559613 |
| ELF1 | ZNF696 | 0.686250353 |
| ELF1 | UBL3 | 0.686118605 |
| ELF1 | GTF2A2 | 0.684333616 |
| ELF1 | CYP7B1 | 0.677208571 |
| ELF1 | PDPN | 0.661547011 |
| ELF1 | NUDT17 | 0.656033936 |
| ELF1 | DDX5 | 0.652528449 |
| ELF1 | CTDSP1 | 0.646456091 |
| ELF1 | PQLC3 | 0.645681752 |
| ELF1 | MTCH1 | 0.642248321 |
| ELF1 | MT1X | 0.63785104 |
| ELF1 | SRRM2 | 0.636362281 |
| ELF1 | IER3 | 0.625238821 |
| ELF1 | EMP2 | 0.620689863 |
| ELF1 | KLF3 | 0.619798127 |
| ELF1 | SMUG1 | 0.598103317 |
| ELF1 | RPL27A | 0.596195113 |
| ELF1 | SH3GLB1 | 0.596097167 |
| ELF1 | PCDHB4 | 0.595929963 |
| ELF1 | TIMP2 | 0.590860311 |
| ELF1 | FSTL1 | 0.587553958 |
| ELF1 | GIGYF1 | 0.58704456 |
| ELF1 | CREB3L1 | 0.586528218 |
| ELF1 | NDUFA1 | 0.584219408 |
| ELF1 | HECTD1 | 0.581511333 |
| ELF1 | MRPL9 | 0.579190307 |
| ELF1 | TMX2 | 0.577546226 |
| ELF1 | POC1A | 0.576749831 |
| ELF1 | RAB10 | 0.572706652 |
| ELF1 | FOXL1 | 0.567320189 |
| ELF1 | GSTCD | 0.564274316 |
| ELF1 | MTPN | 0.551614056 |
| ELF1 | C14orf28 | 0.539447572 |
| ELF1 | GYPC | 0.5371168 |
| ELF1 | COPE | 0.534789496 |
| ELF1 | THRAP3 | 0.532115847 |
| ELF1 | ARHGAP10 | 0.532107253 |
| ELF1 | ENY2 | 0.529705451 |
| ELF1 | MACROD2 | 0.5168268 |
| ELF1 | UBE2D3 | 0.511114008 |
| ELF1 | CCDC82 | 0.498318914 |
| ELF1 | RPL12 | 0.491824337 |
| ELF1 | UBE2E2 | 0.490458487 |
| ELF1 | HSPA8 | 0.488376945 |
| ELF1 | CXCR4 | 0.482635593 |
| ELF1 | BHLHE40 | 0.481204994 |
| ELF1 | KAZALD1 | 0.480303656 |
| ELF1 | SLC1A3 | 0.474949461 |
| ELF1 | BOC | 0.470883037 |
| ELF1 | COL4A1 | 0.462551502 |
| ELF1 | CD70 | 0.462501804 |
| ELF1 | ELMO3 | 0.461851353 |
| ELF1 | PRKACA | 0.460797761 |
| ELF1 | PROM2 | 0.460781897 |
| ELF1 | TM9SF3 | 0.454466608 |
| ELF1 | IGFBP5 | 0.452884667 |
| ELF1 | TNFAIP2 | 0.447391975 |
| ELF1 | DNAJC25 | 0.442365675 |
| ELF1 | PLXNA2 | 0.442313816 |
| ELF1 | SHC3 | 0.441170387 |
| ELF1 | BRI3 | 0.44019228 |
| ELF1 | AEBP1 | 0.437529506 |
| ELF1 | POU3F4 | 0.427127528 |
| ELF1 | SERP1 | 0.421845633 |
| ELF1 | GSC | 0.40934236 |
| ELF1 | FAM114A2 | 0.405829857 |
| ELF1 | TRIM9 | 0.405592449 |
| ELF1 | FANK1 | 0.38933901 |
| ELF1 | PITX3 | 0.387471484 |
| ELF1 | ASPHD2 | 0.386843486 |
| ELF1 | ROR1 | 0.386131876 |
| ELF1 | SHE | 0.383546691 |
| ELF1 | ZNF18 | 0.382142732 |
| ELF1 | LIG1 | 0.37628264 |
| ELF1 | MTSS1L | 0.37020423 |
| ELF1 | NHSL1 | 0.367806845 |
| ELF1 | PDE4A | 0.360845934 |
| ELF1 | BCL9 | 0.358819604 |
| ELF1 | ST8SIA2 | 0.356735554 |
| ELF1 | UBR7 | 0.351326186 |
| ELF1 | HOXB8 | 0.350571942 |
| ELF1 | CNST | 0.34579712 |
| ELF1 | RAX | 0.332894489 |
| ELF1 | RPL36A | 0.331536006 |
| ELF1 | LMBR1 | 0.330183815 |
| ELF1 | MGAT1 | 0.328178541 |
| ELF1 | PDE4B | 0.327106037 |
| ELF1 | RBMS2 | 0.321600456 |
| ELF1 | ZKSCAN5 | 0.319253053 |
| ELF1 | ZNF438 | 0.318047591 |
| ELF1 | IL1R2 | 0.317082228 |
| ELF1 | ING4 | 0.310811459 |
| ELF1 | PCDH7 | 0.309445866 |
| ELF1 | PIH1D1 | 0.306639012 |
| ELF1 | ZNF585A | 0.30586309 |
| ELF1 | AMBRA1 | 0.304580578 |
| ELF1 | ZFHX3 | 0.303551826 |
| ELF1 | SOCS6 | 0.29998351 |
| ELF1 | TSPAN17 | 0.294740715 |
| ELF1 | DIAPH2 | 0.292072033 |
| ELF1 | NFIA | 0.290018304 |
| ELF1 | MSX1 | 0.288494329 |
| ELF1 | TRIM8 | 0.28590868 |
| ELF1 | TBCEL | 0.278613357 |
| ELF1 | E2F1 | 0.277264772 |
| ELF1 | EPAS1 | 0.276238511 |
| ELF1 | SLC30A7 | 0.274496546 |
| ELF1 | ZNF641 | 0.270197666 |
| ELF1 | ZNF77 | 0.269173201 |
| ELF1 | KBTBD4 | 0.266346013 |
| ELF1 | SDC1 | 0.263136209 |
| ELF1 | PKIA | 0.262767039 |
| ELF1 | ICA1 | 0.257381894 |
| ELF1 | GNL1 | 0.256943111 |
| ELF1 | PCYT2 | 0.255370151 |
| ELF1 | SRGAP3 | 0.255085981 |
| ELF1 | UQCRC2 | 0.24798935 |
| ELF1 | PSMC5 | 0.247314416 |
| ELF1 | C19orf25 | 0.244793549 |
| ELF1 | ZNF121 | 0.243369502 |
| ELF1 | NUDT14 | 0.243305275 |
| ELF1 | GREM1 | 0.242314421 |
| ELF1 | OLFM1 | 0.240418345 |
| ELF1 | POLR2H | 0.239060645 |
| ELF1 | DLX3 | 0.237210668 |
| ELF1 | ZDHHC4 | 0.237100395 |
| ELF1 | PACRGL | 0.236600416 |
| ELF1 | TRIP12 | 0.23459423 |
| ELF1 | FAM107B | 0.234587317 |
| ELF1 | EGLN3 | 0.233857486 |
| ELF1 | LARS | 0.232748735 |
| ELF1 | SFT2D1 | 0.230687348 |
| ELF1 | AHCYL2 | 0.228543528 |
| ELF1 | INSIG1 | 0.227817717 |
| ELF1 | NIPA2 | 0.22405147 |
| ELF1 | LIPA | 0.221911407 |
| ELF1 | ZNF574 | 0.220948096 |
| ELF1 | ZNF644 | 0.220599002 |
| ELF1 | DCBLD2 | 0.219937534 |
| ELF1 | BET1L | 0.218184238 |
| ELF1 | CREB1 | 0.216922302 |
| ELF1 | RAPGEF4 | 0.215598406 |
| ELF1 | CLK3 | 0.214608353 |
| ELF1 | SLCO3A1 | 0.213595976 |
| ELF1 | ELOVL5 | 0.213216633 |
| ELF1 | ATG7 | 0.212844944 |
| ELF1 | RBAK | 0.212299612 |
| ELF1 | NAALAD2 | 0.211842391 |
| ELF1 | CDK13 | 0.21148792 |
| ELF1 | PSMA6 | 0.211417063 |
| ELF1 | ARRDC1 | 0.210254952 |
| ELF1 | COQ4 | 0.205278639 |
| ELF1 | BAZ1A | 0.204726001 |
| ELF1 | WWP2 | 0.204103796 |
| ELF1 | TBX3 | 0.202993911 |
| ELF1 | LAMA5 | 0.20187698 |
| ELF1 | TRIO | 0.199047332 |
| ELF1 | WDR18 | 0.198433622 |
| ELF1 | CCDC6 | 0.197831602 |
| ELF1 | AATF | 0.19681423 |
| ELF1 | HCCS | 0.194323495 |
| ELF1 | TEAD2 | 0.192165008 |
| ELF1 | UBR5 | 0.188721284 |
| ELF1 | RGS19 | 0.185430383 |
| ELF1 | METRNL | 0.18278597 |
| ELF1 | VEZF1 | 0.181000746 |
| ELF1 | SNRPN | 0.178882237 |
| ELF1 | ANKRD34A | 0.178110639 |
| ELF1 | GMPPB | 0.177828953 |
| ELF1 | FAM91A1 | 0.177716572 |
| ELF1 | MYH10 | 0.177091567 |
| ELF1 | PHTF2 | 0.175277211 |
| ELF1 | TMEM41B | 0.173676535 |
| ELF1 | BRPF1 | 0.173328525 |
| ELF1 | TUBE1 | 0.173063841 |
| ELF1 | ZNF28 | 0.17270899 |
| ELF1 | CHMP1B | 0.172604905 |
| ELF1 | GALNT13 | 0.16940375 |
| ELF1 | TMOD2 | 0.16837397 |
| ELF1 | ALCAM | 0.1673874 |
| ELF1 | TM2D3 | 0.166136641 |
| ELF1 | CCDC71 | 0.164317864 |
| ELF1 | NCOR2 | 0.163301193 |
| ELF1 | LPCAT4 | 0.160488753 |
| ELF1 | MYL3 | 0.160402189 |
| ELF1 | CIAPIN1 | 0.159969412 |
| ELF1 | PTGIR | 0.159960115 |
| ELF1 | NAB1 | 0.157023309 |
| ELF1 | ETFDH | 0.156612657 |
| ELF1 | ZNF263 | 0.155209046 |
| ELF1 | ACLY | 0.153115337 |
| ELF1 | ELMOD2 | 0.148916713 |
| ELF1 | RPRD1B | 0.147674695 |
| ELF1 | HOXC9 | 0.147139235 |
| ELF1 | DPY19L3 | 0.14702284 |
| ELF1 | FOXP1 | 0.144871734 |
| ELF1 | EBF3 | 0.13645725 |
| ELF1 | FAM180A | 0.135156921 |
| ELF1 | PAPPA2 | 0.131388568 |
| ELF1 | CLEC1A | 0.130462763 |
| ELF1 | EMILIN2 | 0.130325815 |
| ELF1 | MPRIP | 0.130097987 |
| ELF1 | NUDT3 | 0.129593127 |
| ELF1 | NXF1 | 0.127867846 |
| ELF1 | GMEB1 | 0.124378934 |
| ELF1 | TBX2 | 0.124027799 |
| ELF1 | MPHOSPH9 | 0.122616208 |
| ELF1 | SAP30L | 0.121464588 |
| ELF1 | ANGPTL7 | 0.118168647 |
| ELF1 | ECT2 | 0.117160531 |
| ELF1 | PDE4D | 0.112549324 |
| ELF1 | NAA35 | 0.111507544 |
| ELF1 | NUP155 | 0.111210018 |
| ELF1 | ACVR2B | 0.105830777 |
| ELF1 | ZFPM2 | 0.103091649 |
| ELF1 | ABCB7 | 0.102298693 |
| ELF1 | GNL2 | 0.099960448 |
| ELF1 | ATP2C1 | 0.097745869 |
| ELF1 | DZIP1 | 0.091231021 |
| ELF1 | HOXC10 | 0.08836045 |
| ELF1 | SH3RF3 | 0.085585843 |
| ELF1 | PGAP3 | 0.076377166 |
| ELF1 | ZNF746 | 0.073946266 |
| ELF1 | TAF10 | 0.073189202 |
| ELF1 | ZNF70 | 0.069393464 |
| ELF1 | ALOX5AP | 0.058860835 |
| ELF1 | GLS | 0.053275113 |
| ELF1 | FBRS | 0.051272641 |
| ELF1 | HLA-DMB | 0.049274605 |
| ELF1 | MICALL2 | 0.048001952 |
| ELF1 | ZNF653 | 0.040065197 |
| ELF1 | PTPN6 | 0.038378364 |
| ELF1 | INHBB | 0.03120646 |
| ELF1 | GRIK2 | 0.029563836 |
| ELF1 | ESPN | 0.027808232 |
| ELF1 | EVI5 | 0.025616355 |
| ELF1 | TCHH | 0.021967009 |
| ELF1 | ATP6V1C1 | 0.020757618 |
| ELF1 | ACO1 | 0.020171993 |
| ELF1 | ABHD13 | 0.016427148 |
| ELF1 | HELZ | 0.008295676 |
| ELF1 | SLC20A1 | 0.007526145 |
| ELF1 | PPP1R12C | 0.002023188 |
| ELF1 | CBWD2 | 0.000948868 |
| ELF1 | ZC3HAV1 | 0.000478951 |
| ELF1 | SART1 | 9.99E-05 |
| ELF1 | KCNAB2 | 2.05E-05 |
| ELF1 | ZNF365 | 3.62E-06 |
| ELF1 | SORT1 | 2.45E-06 |
| ELF1 | PTPN3 | 9.40E-07 |
| ELF1 | ALDH3B1 | 1.19E-18 |
| ELF2 | NFIX | 8.079325475 |
| ELF2 | OLFML3 | 4.655913512 |
| ELF2 | SEC24A | 3.968989505 |
| ELF2 | RPS12 | 3.947245438 |
| ELF2 | RPSA | 3.90655336 |
| ELF2 | RPL27 | 3.831720715 |
| ELF2 | SIGMAR1 | 3.3344796 |
| ELF2 | IMPAD1 | 3.29850342 |
| ELF2 | NUCB1 | 3.123618008 |
| ELF2 | SERPING1 | 2.936917144 |
| ELF2 | C9orf78 | 2.887018238 |
| ELF2 | ITGB1 | 2.820508335 |
| ELF2 | SREK1IP1 | 2.796010201 |
| ELF2 | RHEB | 2.653834718 |
| ELF2 | DNTTIP1 | 2.483710688 |
| ELF2 | E2F4 | 2.433611463 |
| ELF2 | ANAPC16 | 2.400659482 |
| ELF2 | RNH1 | 2.32379898 |
| ELF2 | CTSK | 2.301895267 |
| ELF2 | CCDC80 | 2.293957541 |
| ELF2 | IGFBP5 | 2.277897267 |
| ELF2 | C1R | 2.208284981 |
| ELF2 | GPNMB | 2.15931464 |
| ELF2 | CENPB | 2.138430868 |
| ELF2 | SGCB | 2.12672118 |
| ELF2 | RORA | 2.101890632 |
| ELF2 | PLAC9 | 2.077361973 |
| ELF2 | IFNGR1 | 2.067760996 |
| ELF2 | CCDC90B | 2.063005517 |
| ELF2 | TMBIM6 | 2.007285069 |
| ELF2 | CTBP2 | 1.991289501 |
| ELF2 | FBLN2 | 1.972645518 |
| ELF2 | TMEM30A | 1.967067977 |
| ELF2 | CBX3 | 1.860698832 |
| ELF2 | F10 | 1.789049495 |
| ELF2 | SNW1 | 1.738472345 |
| ELF2 | RPL37 | 1.730400251 |
| ELF2 | PPP1R3E | 1.724769079 |
| ELF2 | ABCA8 | 1.718817764 |
| ELF2 | IFNGR2 | 1.69679991 |
| ELF2 | PSMA4 | 1.681370287 |
| ELF2 | DMAP1 | 1.628328176 |
| ELF2 | SMARCE1 | 1.623140393 |
| ELF2 | SPATA5L1 | 1.614148879 |
| ELF2 | ARHGAP31 | 1.604612064 |
| ELF2 | FEM1B | 1.600899297 |
| ELF2 | OSR1 | 1.587050338 |
| ELF2 | SPATS2L | 1.572343181 |
| ELF2 | TRABD | 1.567073066 |
| ELF2 | SRP9 | 1.565006154 |
| ELF2 | TYROBP | 1.556606214 |
| ELF2 | H1FX | 1.528229355 |
| ELF2 | BSG | 1.485281807 |
| ELF2 | RHOG | 1.48371864 |
| ELF2 | SUMO2 | 1.47501435 |
| ELF2 | ARL6IP5 | 1.450612925 |
| ELF2 | RPP14 | 1.444521017 |
| ELF2 | SATB1 | 1.429190056 |
| ELF2 | NUDC | 1.428948436 |
| ELF2 | MAGEH1 | 1.408105623 |
| ELF2 | CNTFR | 1.395412357 |
| ELF2 | GPC4 | 1.391913863 |
| ELF2 | CD81 | 1.390803833 |
| ELF2 | NIPAL1 | 1.37297215 |
| ELF2 | FGF7 | 1.363750721 |
| ELF2 | MFHAS1 | 1.353212263 |
| ELF2 | IFT88 | 1.347142385 |
| ELF2 | CTSZ | 1.332980217 |
| ELF2 | PTPN6 | 1.331273418 |
| ELF2 | PRLR | 1.320193965 |
| ELF2 | RBM43 | 1.314921425 |
| ELF2 | DNASE1L1 | 1.309782125 |
| ELF2 | PTPRC | 1.309201041 |
| ELF2 | RPL28 | 1.301959038 |
| ELF2 | PTCH1 | 1.282828507 |
| ELF2 | DIO3OS | 1.277888973 |
| ELF2 | THOC3 | 1.27339815 |
| ELF2 | NELL1 | 1.265563587 |
| ELF2 | MDK | 1.255444121 |
| ELF2 | PRIM1 | 1.255034285 |
| ELF2 | DDX10 | 1.244931177 |
| ELF2 | APOE | 1.239704008 |
| ELF2 | VCPIP1 | 1.232451387 |
| ELF2 | PLEKHB2 | 1.214841862 |
| ELF2 | EMID1 | 1.207294328 |
| ELF2 | PPIL2 | 1.205232287 |
| ELF2 | DACT1 | 1.192648241 |
| ELF2 | LMBR1L | 1.186627216 |
| ELF2 | TESK1 | 1.180285608 |
| ELF2 | P2RY1 | 1.15232966 |
| ELF2 | CDR2L | 1.147169322 |
| ELF2 | LLPH | 1.129908876 |
| ELF2 | TBK1 | 1.121197875 |
| ELF2 | EPHB2 | 1.120328537 |
| ELF2 | NOSIP | 1.117584749 |
| ELF2 | C11orf68 | 1.110603361 |
| ELF2 | SERPINF1 | 1.109906863 |
| ELF2 | RPL6 | 1.094711876 |
| ELF2 | TMEM8B | 1.086609344 |
| ELF2 | C11orf74 | 1.079578387 |
| ELF2 | HSP90B1 | 1.075976106 |
| ELF2 | PHLDA3 | 1.067442019 |
| ELF2 | PPP1R16A | 1.066549076 |
| ELF2 | PSTPIP2 | 1.065484272 |
| ELF2 | BARD1 | 1.060251089 |
| ELF2 | JRKL | 1.056852938 |
| ELF2 | ERCC4 | 1.042353486 |
| ELF2 | PYGB | 1.042319643 |
| ELF2 | EPB41L4A | 1.035186777 |
| ELF2 | TMEM129 | 1.030104868 |
| ELF2 | TBC1D5 | 1.028900916 |
| ELF2 | RARRES3 | 1.024986735 |
| ELF2 | ALPL | 1.023217576 |
| ELF2 | ACTR2 | 1.021970224 |
| ELF2 | HMGB1 | 1.012024012 |
| ELF2 | FHL1 | 1.009308261 |
| ELF2 | ELF2 | 1 |
| ELF2 | RPS15A | 0.99533101 |
| ELF2 | STMN3 | 0.994606677 |
| ELF2 | AMOTL1 | 0.989223606 |
| ELF2 | GBE1 | 0.988456 |
| ELF2 | MORC3 | 0.978643907 |
| ELF2 | THNSL2 | 0.975787647 |
| ELF2 | SNRPD2 | 0.97254497 |
| ELF2 | PROS1 | 0.965718611 |
| ELF2 | RPL11 | 0.963166141 |
| ELF2 | XPNPEP3 | 0.959589917 |
| ELF2 | ATP6V1C2 | 0.959330172 |
| ELF2 | RINT1 | 0.953063681 |
| ELF2 | DNAJB1 | 0.951756069 |
| ELF2 | PTK6 | 0.949636303 |
| ELF2 | ITPK1 | 0.94940869 |
| ELF2 | P2RX4 | 0.947395119 |
| ELF2 | EXOSC1 | 0.938807156 |
| ELF2 | UBN1 | 0.937352393 |
| ELF2 | RPL3 | 0.937152259 |
| ELF2 | PRDX4 | 0.928883767 |
| ELF2 | ARHGEF10L | 0.926555186 |
| ELF2 | COX6B1 | 0.906283234 |
| ELF2 | LRRC1 | 0.906038018 |
| ELF2 | HTT | 0.902068528 |
| ELF2 | GFOD1 | 0.900499969 |
| ELF2 | SLC39A7 | 0.89669718 |
| ELF2 | KATNAL1 | 0.895777194 |
| ELF2 | SLITRK6 | 0.889027695 |
| ELF2 | FAM45A | 0.887703163 |
| ELF2 | RPL37A | 0.885917208 |
| ELF2 | TAX1BP3 | 0.871360071 |
| ELF2 | GNA12 | 0.867153909 |
| ELF2 | FBXO4 | 0.864352054 |
| ELF2 | TSPAN4 | 0.860489498 |
| ELF2 | RPL30 | 0.859841073 |
| ELF2 | NDRG3 | 0.857977439 |
| ELF2 | ABCA9 | 0.852228401 |
| ELF2 | PNPLA2 | 0.852209562 |
| ELF2 | U2AF1L4 | 0.851894695 |
| ELF2 | GPX1 | 0.850627615 |
| ELF2 | SETD6 | 0.838780344 |
| ELF2 | PSME1 | 0.835638063 |
| ELF2 | MFSD9 | 0.826368403 |
| ELF2 | SLC35B2 | 0.824526241 |
| ELF2 | NAP1L4 | 0.819427392 |
| ELF2 | MBOAT7 | 0.818752036 |
| ELF2 | SLC22A5 | 0.817997082 |
| ELF2 | IQCH | 0.812715872 |
| ELF2 | COL23A1 | 0.807802461 |
| ELF2 | FADS2 | 0.797540267 |
| ELF2 | CREB3L2 | 0.797281246 |
| ELF2 | ZNF780A | 0.795786755 |
| ELF2 | EMP3 | 0.794935143 |
| ELF2 | BAZ2A | 0.793467388 |
| ELF2 | MEA1 | 0.791756136 |
| ELF2 | RPL12 | 0.791655777 |
| ELF2 | RPL10A | 0.783643523 |
| ELF2 | ZNF25 | 0.773965648 |
| ELF2 | HTRA3 | 0.768137531 |
| ELF2 | SAMHD1 | 0.759322161 |
| ELF2 | TMED7 | 0.752333954 |
| ELF2 | TFPI | 0.750626444 |
| ELF2 | SDHAF2 | 0.748919338 |
| ELF2 | CPXM2 | 0.747644769 |
| ELF2 | RPL24 | 0.74567803 |
| ELF2 | CYBRD1 | 0.741346909 |
| ELF2 | RPL5 | 0.741031225 |
| ELF2 | PRPS2 | 0.73900523 |
| ELF2 | DUT | 0.738327361 |
| ELF2 | CAP1 | 0.737386187 |
| ELF2 | DCAF7 | 0.734497266 |
| ELF2 | MID1IP1 | 0.733867209 |
| ELF2 | KIAA1328 | 0.729996654 |
| ELF2 | ALDH1A1 | 0.727757274 |
| ELF2 | PIGA | 0.723523228 |
| ELF2 | IDH3A | 0.722915596 |
| ELF2 | COPS7A | 0.719779153 |
| ELF2 | ZNF436 | 0.717800599 |
| ELF2 | SLC33A1 | 0.717230797 |
| ELF2 | NSFL1C | 0.716819146 |
| ELF2 | TRIM37 | 0.716751396 |
| ELF2 | TEX264 | 0.713483108 |
| ELF2 | SLC30A7 | 0.711350051 |
| ELF2 | FAM13C | 0.708760724 |
| ELF2 | TRMT6 | 0.706651377 |
| ELF2 | TARSL2 | 0.699118023 |
| ELF2 | SH3BP5L | 0.697178634 |
| ELF2 | PRR16 | 0.697028102 |
| ELF2 | HSCB | 0.696888359 |
| ELF2 | AP1AR | 0.696035965 |
| ELF2 | KITLG | 0.687597178 |
| ELF2 | RPL41 | 0.686283475 |
| ELF2 | POGZ | 0.684740786 |
| ELF2 | PICALM | 0.681023662 |
| ELF2 | RTN4RL2 | 0.67811504 |
| ELF2 | GADD45GIP1 | 0.67778767 |
| ELF2 | GAS6 | 0.669281191 |
| ELF2 | COX16 | 0.666319778 |
| ELF2 | NDUFA3 | 0.66424282 |
| ELF2 | TRIM2 | 0.661501761 |
| ELF2 | ALKBH2 | 0.658004707 |
| ELF2 | BMF | 0.651082709 |
| ELF2 | PPIC | 0.648479886 |
| ELF2 | RPL36AL | 0.647245772 |
| ELF2 | RBM3 | 0.645880508 |
| ELF2 | RPLP2 | 0.64528152 |
| ELF2 | YWHAB | 0.644768125 |
| ELF2 | TOMM7 | 0.641688162 |
| ELF2 | MYC | 0.638275238 |
| ELF2 | GPX4 | 0.634368222 |
| ELF2 | MLLT1 | 0.633256349 |
| ELF2 | KLF10 | 0.631960819 |
| ELF2 | SDF2 | 0.627704971 |
| ELF2 | PAIP2 | 0.627212452 |
| ELF2 | TNRC6B | 0.626373231 |
| ELF2 | C1D | 0.622935506 |
| ELF2 | MORF4L2 | 0.622697218 |
| ELF2 | POP5 | 0.619599669 |
| ELF2 | PDK4 | 0.61334205 |
| ELF2 | GIGYF2 | 0.612475403 |
| ELF2 | XPNPEP2 | 0.611693469 |
| ELF2 | ZNF155 | 0.610536685 |
| ELF2 | SEC16A | 0.602787683 |
| ELF2 | CYFIP1 | 0.602005967 |
| ELF2 | TMEM128 | 0.600619583 |
| ELF2 | CCT7 | 0.59464381 |
| ELF2 | LY6K | 0.593629402 |
| ELF2 | SIRT2 | 0.593163327 |
| ELF2 | RAB3GAP2 | 0.587980064 |
| ELF2 | CREBBP | 0.585755625 |
| ELF2 | PELO | 0.585280656 |
| ELF2 | CBR4 | 0.585036273 |
| ELF2 | HCG27 | 0.584977217 |
| ELF2 | HNRNPK | 0.583947555 |
| ELF2 | INF2 | 0.580349078 |
| ELF2 | PDIA5 | 0.579472233 |
| ELF2 | HMOX2 | 0.569011018 |
| ELF2 | MSX1 | 0.568820401 |
| ELF2 | TEX10 | 0.565571704 |
| ELF2 | SSR2 | 0.565352555 |
| ELF2 | ZRANB2 | 0.565156662 |
| ELF2 | MEOX2 | 0.564273903 |
| ELF2 | DHFR | 0.559627266 |
| ELF2 | LZIC | 0.558999791 |
| ELF2 | LRIG1 | 0.558213056 |
| ELF2 | FAM198B | 0.556876225 |
| ELF2 | GDE1 | 0.55669261 |
| ELF2 | MAP7D3 | 0.553967671 |
| ELF2 | VPS37C | 0.553849601 |
| ELF2 | RPL7 | 0.553168248 |
| ELF2 | BST2 | 0.552911294 |
| ELF2 | KDSR | 0.552650496 |
| ELF2 | SSR3 | 0.552548624 |
| ELF2 | ANGPTL5 | 0.550213025 |
| ELF2 | WDR45 | 0.550181218 |
| ELF2 | GSDMD | 0.544854657 |
| ELF2 | CDCA5 | 0.544629579 |
| ELF2 | NR1D2 | 0.540648715 |
| ELF2 | WNT3 | 0.539209945 |
| ELF2 | NEXN | 0.539174746 |
| ELF2 | SH3PXD2B | 0.53552839 |
| ELF2 | FAM120AOS | 0.530219447 |
| ELF2 | DPYSL2 | 0.528363079 |
| ELF2 | POLR2E | 0.528310388 |
| ELF2 | ITM2B | 0.527009283 |
| ELF2 | ALDH3A1 | 0.522520728 |
| ELF2 | NOL6 | 0.521261546 |
| ELF2 | RBM34 | 0.518163162 |
| ELF2 | CAMK4 | 0.517926565 |
| ELF2 | SMOC2 | 0.517249162 |
| ELF2 | RCL1 | 0.516702552 |
| ELF2 | CSRP2 | 0.516247455 |
| ELF2 | IGFBP2 | 0.513388086 |
| ELF2 | PLAGL1 | 0.513134204 |
| ELF2 | IQSEC3 | 0.511619734 |
| ELF2 | RAB21 | 0.510119862 |
| ELF2 | ZNF672 | 0.509093498 |
| ELF2 | SUPT16H | 0.508319989 |
| ELF2 | RXRG | 0.507712681 |
| ELF2 | SF3B2 | 0.507321587 |
| ELF2 | NPC2 | 0.506696936 |
| ELF2 | RPL23 | 0.506647044 |
| ELF2 | NCAPD2 | 0.505943676 |
| ELF2 | PPCS | 0.503301179 |
| ELF2 | BAIAP2 | 0.502655003 |
| ELF2 | TCTA | 0.499637861 |
| ELF2 | TMEM176B | 0.498210754 |
| ELF2 | SIK3 | 0.4964442 |
| ELF2 | MAGED1 | 0.49443468 |
| ELF2 | NDUFB6 | 0.493045054 |
| ELF2 | HSPH1 | 0.492767961 |
| ELF2 | SPATA24 | 0.490949217 |
| ELF2 | DDX6 | 0.490735623 |
| ELF2 | DMXL2 | 0.489098404 |
| ELF2 | CYB561 | 0.488273262 |
| ELF2 | HIPK3 | 0.48524865 |
| ELF2 | NACC2 | 0.485072021 |
| ELF2 | RPL15 | 0.483904137 |
| ELF2 | UBL5 | 0.481691548 |
| ELF2 | BCCIP | 0.481689343 |
| ELF2 | PKN2 | 0.474199031 |
| ELF2 | IZUMO4 | 0.472264734 |
| ELF2 | RPS5 | 0.4721304 |
| ELF2 | JMJD1C | 0.469621363 |
| ELF2 | RAP2C | 0.467673636 |
| ELF2 | PRDX2 | 0.467347185 |
| ELF2 | TIMM44 | 0.467100653 |
| ELF2 | MAGI3 | 0.46538061 |
| ELF2 | SCAI | 0.464511254 |
| ELF2 | UBA3 | 0.462174073 |
| ELF2 | IAH1 | 0.460936392 |
| ELF2 | LYSMD2 | 0.460178615 |
| ELF2 | CISD3 | 0.458412451 |
| ELF2 | ANXA6 | 0.457690187 |
| ELF2 | TXNIP | 0.455237789 |
| ELF2 | ASTE1 | 0.454904507 |
| ELF2 | FUT10 | 0.452033944 |
| ELF2 | TMEM184B | 0.451576679 |
| ELF2 | CD44 | 0.450784595 |
| ELF2 | ETV4 | 0.449742478 |
| ELF2 | FGL2 | 0.448794949 |
| ELF2 | TOP2B | 0.44319604 |
| ELF2 | COG2 | 0.442580322 |
| ELF2 | PTPRU | 0.442156149 |
| ELF2 | MBNL2 | 0.441528203 |
| ELF2 | ATAD5 | 0.440523991 |
| ELF2 | GJB2 | 0.439080176 |
| ELF2 | LANCL1 | 0.438497087 |
| ELF2 | NDUFAF4 | 0.437245512 |
| ELF2 | HP1BP3 | 0.434899494 |
| ELF2 | SCARF1 | 0.434891372 |
| ELF2 | DNAH6 | 0.433911091 |
| ELF2 | RPL35 | 0.433737927 |
| ELF2 | ZNF85 | 0.432988212 |
| ELF2 | EPM2AIP1 | 0.429985565 |
| ELF2 | KCTD11 | 0.42840714 |
| ELF2 | ARPP19 | 0.42828063 |
| ELF2 | CORO2B | 0.427804588 |
| ELF2 | ZNF595 | 0.427198137 |
| ELF2 | FAM109A | 0.424777885 |
| ELF2 | PRR13 | 0.422303173 |
| ELF2 | CGGBP1 | 0.421730287 |
| ELF2 | TANK | 0.421308953 |
| ELF2 | CUL7 | 0.420119552 |
| ELF2 | ANGPT1 | 0.419081524 |
| ELF2 | SMARCA2 | 0.417358633 |
| ELF2 | LPXN | 0.417314037 |
| ELF2 | LUC7L2 | 0.416811994 |
| ELF2 | THUMPD3 | 0.415837058 |
| ELF2 | ADPRHL1 | 0.414487796 |
| ELF2 | PCOLCE | 0.413716623 |
| ELF2 | ZCWPW2 | 0.413467814 |
| ELF2 | WWC1 | 0.411553038 |
| ELF2 | MTHFR | 0.410863063 |
| ELF2 | GPATCH1 | 0.410196463 |
| ELF2 | ZNF329 | 0.392444448 |
| ELF2 | TM2D2 | 0.390937463 |
| ELF2 | CALCA | 0.388900151 |
| ELF2 | TSPAN8 | 0.386599813 |
| ELF2 | DCTN2 | 0.385056333 |
| ELF2 | ZNF738 | 0.38468414 |
| ELF2 | WRN | 0.382804735 |
| ELF2 | NIPBL | 0.379372194 |
| ELF2 | CRY1 | 0.37814646 |
| ELF2 | C19orf12 | 0.373215926 |
| ELF2 | SETMAR | 0.372191267 |
| ELF2 | PCDH17 | 0.371079854 |
| ELF2 | LRP1B | 0.366082976 |
| ELF2 | PLA2G15 | 0.3652717 |
| ELF2 | ARHGAP23 | 0.36491091 |
| ELF2 | B3GALT4 | 0.361118482 |
| ELF2 | UBB | 0.359998108 |
| ELF2 | SLC34A2 | 0.359444182 |
| ELF2 | SLC44A2 | 0.35404861 |
| ELF2 | MAP1A | 0.352824799 |
| ELF2 | HADHB | 0.352719991 |
| ELF2 | L3MBTL3 | 0.35188302 |
| ELF2 | SMURF2 | 0.349116377 |
| ELF2 | ZNF334 | 0.347313768 |
| ELF2 | FGFR2 | 0.34573977 |
| ELF2 | MFSD1 | 0.34499394 |
| ELF2 | AGTR1 | 0.344164432 |
| ELF2 | ZNF521 | 0.342356812 |
| ELF2 | ZNF100 | 0.341512622 |
| ELF2 | IGSF3 | 0.33979211 |
| ELF2 | ARIH1 | 0.338761413 |
| ELF2 | SORL1 | 0.338677797 |
| ELF2 | ZNF609 | 0.337718133 |
| ELF2 | IL7R | 0.337095785 |
| ELF2 | GOPC | 0.337050397 |
| ELF2 | ATG16L1 | 0.33576953 |
| ELF2 | ZNF578 | 0.3340621 |
| ELF2 | SLAIN2 | 0.333112249 |
| ELF2 | KCNMA1 | 0.332387013 |
| ELF2 | CMTM8 | 0.331711998 |
| ELF2 | ARFIP2 | 0.3312082 |
| ELF2 | RPE | 0.331150906 |
| ELF2 | PLCB2 | 0.329700532 |
| ELF2 | E2F1 | 0.329316737 |
| ELF2 | ANKS3 | 0.326524669 |
| ELF2 | CAMSAP1 | 0.324622421 |
| ELF2 | AP3M1 | 0.324544052 |
| ELF2 | ZBTB33 | 0.323764749 |
| ELF2 | FAM107A | 0.320327169 |
| ELF2 | NOC2L | 0.316771539 |
| ELF2 | ZSCAN5A | 0.31587069 |
| ELF2 | EIF5AL1 | 0.314156908 |
| ELF2 | FAM13A | 0.313560563 |
| ELF2 | ORC4 | 0.313549682 |
| ELF2 | ZDHHC16 | 0.310663988 |
| ELF2 | SCRN1 | 0.306876228 |
| ELF2 | FABP7 | 0.306645267 |
| ELF2 | PGM2L1 | 0.305921677 |
| ELF2 | GMPR | 0.30514968 |
| ELF2 | PLTP | 0.304884569 |
| ELF2 | TSTA3 | 0.303899184 |
| ELF2 | AMIGO2 | 0.303573207 |
| ELF2 | E4F1 | 0.301138277 |
| ELF2 | CA6 | 0.300602319 |
| ELF2 | TRIM7 | 0.300318746 |
| ELF2 | CLCN2 | 0.299961988 |
| ELF2 | ATP10D | 0.299128055 |
| ELF2 | CRISPLD1 | 0.298582049 |
| ELF2 | PHLDB1 | 0.297915927 |
| ELF2 | MKRN1 | 0.297197099 |
| ELF2 | SEC14L1 | 0.297035082 |
| ELF2 | PGF | 0.296374697 |
| ELF2 | SRD5A3 | 0.296273094 |
| ELF2 | HEY2 | 0.294737524 |
| ELF2 | RPA2 | 0.292797346 |
| ELF2 | UBE2J2 | 0.291919583 |
| ELF2 | RNF149 | 0.291098981 |
| ELF2 | FGF18 | 0.290163148 |
| ELF2 | ZNF598 | 0.289267513 |
| ELF2 | TRAF2 | 0.288324761 |
| ELF2 | ZNF740 | 0.288256398 |
| ELF2 | TCF7L2 | 0.28796224 |
| ELF2 | LIMCH1 | 0.285806533 |
| ELF2 | PIH1D1 | 0.284932008 |
| ELF2 | WDR37 | 0.283247674 |
| ELF2 | TIMM23 | 0.282145586 |
| ELF2 | H19 | 0.28152857 |
| ELF2 | PPP6R1 | 0.280566135 |
| ELF2 | TMEM204 | 0.280225742 |
| ELF2 | SLC4A1AP | 0.27856146 |
| ELF2 | RAB22A | 0.277816654 |
| ELF2 | BMP4 | 0.277366713 |
| ELF2 | GGA1 | 0.275376812 |
| ELF2 | CAMTA1 | 0.274578512 |
| ELF2 | PIP4K2B | 0.274488626 |
| ELF2 | SFT2D1 | 0.274154398 |
| ELF2 | IDS | 0.273190006 |
| ELF2 | CD2BP2 | 0.272308011 |
| ELF2 | SLC25A23 | 0.271814373 |
| ELF2 | PRX | 0.27107777 |
| ELF2 | PGM2 | 0.270585576 |
| ELF2 | LRRC32 | 0.268377015 |
| ELF2 | CADPS2 | 0.268116879 |
| ELF2 | NAV1 | 0.267637676 |
| ELF2 | GTPBP3 | 0.267370485 |
| ELF2 | RELL2 | 0.267110759 |
| ELF2 | ZBTB10 | 0.266740809 |
| ELF2 | TRIM3 | 0.266374993 |
| ELF2 | WDR5B | 0.263927194 |
| ELF2 | WDR4 | 0.263407123 |
| ELF2 | ACOT9 | 0.263122753 |
| ELF2 | ZNF501 | 0.263069982 |
| ELF2 | STK4 | 0.262622632 |
| ELF2 | GGPS1 | 0.259096845 |
| ELF2 | DYRK3 | 0.256206214 |
| ELF2 | ASAP2 | 0.255800532 |
| ELF2 | ZNF225 | 0.255785104 |
| ELF2 | TSNAXIP1 | 0.255745005 |
| ELF2 | UTRN | 0.253876007 |
| ELF2 | ARHGAP19 | 0.253713322 |
| ELF2 | CILP2 | 0.253552365 |
| ELF2 | NDUFV3 | 0.252081451 |
| ELF2 | ZNF500 | 0.252057697 |
| ELF2 | UCP2 | 0.249741452 |
| ELF2 | HOXC9 | 0.249604352 |
| ELF2 | BMPR2 | 0.248102002 |
| ELF2 | FIBIN | 0.247044162 |
| ELF2 | CLIC2 | 0.246118461 |
| ELF2 | TBX3 | 0.24531057 |
| ELF2 | FGD4 | 0.245161598 |
| ELF2 | LTC4S | 0.24446086 |
| ELF2 | NMNAT1 | 0.243140506 |
| ELF2 | URB1 | 0.242480376 |
| ELF2 | CCDC12 | 0.241889589 |
| ELF2 | PINX1 | 0.24140194 |
| ELF2 | SBNO2 | 0.240542001 |
| ELF2 | SLC29A3 | 0.240016828 |
| ELF2 | PUM2 | 0.23985135 |
| ELF2 | RASSF2 | 0.2393699 |
| ELF2 | ZNF805 | 0.239163573 |
| ELF2 | CSTF2 | 0.236866841 |
| ELF2 | LST1 | 0.235557634 |
| ELF2 | ATRX | 0.234305425 |
| ELF2 | SYT17 | 0.232852249 |
| ELF2 | ISOC2 | 0.232561864 |
| ELF2 | TDG | 0.232551186 |
| ELF2 | EBPL | 0.232260436 |
| ELF2 | FBXO22 | 0.229972003 |
| ELF2 | SHOX2 | 0.229714716 |
| ELF2 | CPSF1 | 0.228905925 |
| ELF2 | MICAL2 | 0.228393026 |
| ELF2 | NIP7 | 0.228266349 |
| ELF2 | ARPC4 | 0.225785211 |
| ELF2 | PMS2CL | 0.225341082 |
| ELF2 | NUDT3 | 0.224920688 |
| ELF2 | ATG4A | 0.224646609 |
| ELF2 | ARSI | 0.223821241 |
| ELF2 | TRIM28 | 0.222684078 |
| ELF2 | TOP3B | 0.22185991 |
| ELF2 | ARHGAP5 | 0.221431936 |
| ELF2 | PPP1CB | 0.221257557 |
| ELF2 | CLTC | 0.221138296 |
| ELF2 | PNPLA8 | 0.220249298 |
| ELF2 | CUL9 | 0.21984131 |
| ELF2 | SNRNP27 | 0.219793255 |
| ELF2 | ANKRD10 | 0.219237017 |
| ELF2 | C3orf38 | 0.21857973 |
| ELF2 | PTER | 0.216170272 |
| ELF2 | NALCN | 0.215668216 |
| ELF2 | ZNF239 | 0.214498884 |
| ELF2 | CA5B | 0.213077192 |
| ELF2 | DDX20 | 0.213026709 |
| ELF2 | LZTFL1 | 0.212860637 |
| ELF2 | ADCYAP1 | 0.210848441 |
| ELF2 | ZNF326 | 0.210433631 |
| ELF2 | PER1 | 0.210057532 |
| ELF2 | ACBD6 | 0.208779461 |
| ELF2 | VTI1A | 0.20873595 |
| ELF2 | CRISPLD2 | 0.208726669 |
| ELF2 | CKMT1B | 0.208663576 |
| ELF2 | SLC35A5 | 0.206547401 |
| ELF2 | CDK13 | 0.206444312 |
| ELF2 | NUS1 | 0.204224771 |
| ELF2 | VPS29 | 0.202868744 |
| ELF2 | YARS2 | 0.201828784 |
| ELF2 | RBM27 | 0.201760872 |
| ELF2 | POLRMT | 0.200934473 |
| ELF2 | HAGHL | 0.199585813 |
| ELF2 | C16orf70 | 0.198971926 |
| ELF2 | RNF4 | 0.198501359 |
| ELF2 | C16orf86 | 0.198179043 |
| ELF2 | PRKG1 | 0.197889983 |
| ELF2 | ESR1 | 0.197281667 |
| ELF2 | RARS | 0.196466985 |
| ELF2 | ALX4 | 0.196108615 |
| ELF2 | MYO9A | 0.195565388 |
| ELF2 | RPP40 | 0.195352302 |
| ELF2 | DSE | 0.192407954 |
| ELF2 | TAF5 | 0.191937993 |
| ELF2 | PPP2R2D | 0.191375561 |
| ELF2 | EIF2B5 | 0.18948603 |
| ELF2 | TMEM219 | 0.188841377 |
| ELF2 | PLA1A | 0.188706053 |
| ELF2 | SLC2A13 | 0.187425046 |
| ELF2 | TSG101 | 0.187265842 |
| ELF2 | NMB | 0.186596489 |
| ELF2 | RBM26 | 0.185330396 |
| ELF2 | IKZF5 | 0.183689517 |
| ELF2 | IL2RG | 0.182360897 |
| ELF2 | ANG | 0.177932147 |
| ELF2 | ERAP1 | 0.177450592 |
| ELF2 | CORO7 | 0.176997734 |
| ELF2 | EPB41L5 | 0.176572092 |
| ELF2 | CHM | 0.174745554 |
| ELF2 | TBC1D8 | 0.173706272 |
| ELF2 | WDR5 | 0.173375372 |
| ELF2 | CHMP1B | 0.172428256 |
| ELF2 | AGAP3 | 0.171223065 |
| ELF2 | ITPR2 | 0.169071968 |
| ELF2 | SCRIB | 0.168735194 |
| ELF2 | THEM4 | 0.168234081 |
| ELF2 | DCLRE1A | 0.167122024 |
| ELF2 | TM7SF3 | 0.166670376 |
| ELF2 | CPT1A | 0.165619426 |
| ELF2 | TSSC4 | 0.162811575 |
| ELF2 | ATP13A1 | 0.162531858 |
| ELF2 | DLGAP4 | 0.161011165 |
| ELF2 | ADAMTS9 | 0.157469343 |
| ELF2 | TBC1D2 | 0.156577122 |
| ELF2 | BAP1 | 0.15649774 |
| ELF2 | MOCS1 | 0.156326941 |
| ELF2 | SF3B4 | 0.155783422 |
| ELF2 | HAAO | 0.155687562 |
| ELF2 | NEDD4L | 0.155165082 |
| ELF2 | MGST2 | 0.153687903 |
| ELF2 | ZNF558 | 0.152747266 |
| ELF2 | STX7 | 0.150766888 |
| ELF2 | C10orf88 | 0.149924426 |
| ELF2 | SLC9A3R2 | 0.149849549 |
| ELF2 | SS18 | 0.148214187 |
| ELF2 | ZNHIT3 | 0.146961905 |
| ELF2 | SLC7A2 | 0.146154884 |
| ELF2 | ANKRD9 | 0.146103207 |
| ELF2 | MDM2 | 0.145658058 |
| ELF2 | WDR74 | 0.143850343 |
| ELF2 | CEP120 | 0.143028085 |
| ELF2 | CDK5RAP1 | 0.142032384 |
| ELF2 | MYO5A | 0.13909205 |
| ELF2 | LRRC8A | 0.136254902 |
| ELF2 | ATP10A | 0.135514156 |
| ELF2 | ZNF462 | 0.133584047 |
| ELF2 | NUDT4 | 0.132189371 |
| ELF2 | RNPS1 | 0.131697376 |
| ELF2 | CYP7B1 | 0.129875525 |
| ELF2 | DENND2D | 0.129601049 |
| ELF2 | BCL7A | 0.12628639 |
| ELF2 | OSGIN2 | 0.124984614 |
| ELF2 | MIDN | 0.123912961 |
| ELF2 | CHCHD4 | 0.123560779 |
| ELF2 | CX3CL1 | 0.123499695 |
| ELF2 | CST6 | 0.12278219 |
| ELF2 | SCG5 | 0.122743548 |
| ELF2 | ANKRD13B | 0.12214217 |
| ELF2 | ZNF701 | 0.118466937 |
| ELF2 | APOL3 | 0.112941493 |
| ELF2 | ETFA | 0.112364626 |
| ELF2 | EIF4E | 0.105638329 |
| ELF2 | ATXN7L3 | 0.104402986 |
| ELF2 | NUP37 | 0.103462127 |
| ELF2 | DDI2 | 0.103421057 |
| ELF2 | ZNF496 | 0.094437839 |
| ELF2 | STK24 | 0.092482378 |
| ELF2 | MLH1 | 0.090788505 |
| ELF2 | NUP98 | 0.089532625 |
| ELF2 | GSPT1 | 0.088185652 |
| ELF2 | ZNF524 | 0.087272833 |
| ELF2 | MLH3 | 0.084861013 |
| ELF2 | MRPL21 | 0.083112206 |
| ELF2 | KCND2 | 0.081884298 |
| ELF2 | ARHGAP6 | 0.080650979 |
| ELF2 | TRIM11 | 0.07807002 |
| ELF2 | IGF2BP2 | 0.076671542 |
| ELF2 | NEBL | 0.07500837 |
| ELF2 | PCDHGA6 | 0.074862828 |
| ELF2 | TMEM209 | 0.069524796 |
| ELF2 | HGF | 0.064429652 |
| ELF2 | SERP2 | 0.06430197 |
| ELF2 | ZNF200 | 0.058357743 |
| ELF2 | PLCH2 | 0.057925361 |
| ELF2 | CCDC94 | 0.055482571 |
| ELF2 | GPR1 | 0.054116461 |
| ELF2 | ITGA3 | 0.051009967 |
| ELF2 | PCDHB15 | 0.05044367 |
| ELF2 | BTBD3 | 0.050341882 |
| ELF2 | ZNF222 | 0.046586721 |
| ELF2 | IGFL2 | 0.043161367 |
| ELF2 | TP53BP2 | 0.042046348 |
| ELF2 | GDF15 | 0.031369757 |
| ELF2 | MGEA5 | 0.030092457 |
| ELF2 | PABPC1L | 0.024801028 |
| ELF2 | GK | 0.020682478 |
| ELF2 | ZBTB38 | 0.015624378 |
| ELF2 | NQO1 | 0.013216327 |
| ELF2 | ATG2A | 0.012935991 |
| ELF2 | DEDD | 0.010938207 |
| ELF2 | MYOM2 | 0.002594716 |
| ELF2 | DNAJC14 | 0.002271948 |
| ELF2 | STK39 | 0.001179515 |
| ELF2 | TRADD | 0.000824476 |
| ELF2 | ZYG11B | 0.000551831 |
| ELF2 | FAM192A | 0.000433512 |
| ELF2 | RAB2B | 0.000427902 |
| ELF2 | EDEM3 | 0.000349841 |
| ELF2 | C1orf52 | 0.000325762 |
| ELF2 | MFN2 | 0.000122429 |
| ELF2 | GLA | 7.99E-05 |
| ELF2 | ZW10 | 2.24E-06 |
| ELF2 | FRS3 | 7.74E-07 |
| ELF2 | ANKRD2 | 2.26E-07 |
| ELF2 | GRB7 | 9.10E-08 |
| ELF2 | SUSD4 | 7.66E-08 |
| ELF2 | MTHFS | 8.51E-11 |
| ELF2 | FANCE | 2.92E-11 |
| ELF2 | THUMPD2 | 1.76E-18 |
| ELF2 | SPECC1 | 1.20E-18 |
| ELF2 | LIN52 | 1.11E-18 |
| ELF2 | CRYBB2 | 3.81E-19 |
| ELF3 | SCARA5 | 3.492406812 |
| ELF3 | CSTF2T | 3.373311491 |
| ELF3 | GNG11 | 3.004699953 |
| ELF3 | IFT74 | 2.855566516 |
| ELF3 | CIRBP | 2.429657492 |
| ELF3 | DYRK2 | 2.036911271 |
| ELF3 | IL6ST | 2.0208682 |
| ELF3 | SNRPN | 1.885185516 |
| ELF3 | LDLRAD3 | 1.869933958 |
| ELF3 | SYT11 | 1.845090826 |
| ELF3 | NEAT1 | 1.837179742 |
| ELF3 | TUBB2B | 1.798795597 |
| ELF3 | LMAN2 | 1.792485472 |
| ELF3 | EGR2 | 1.775488593 |
| ELF3 | CRLF1 | 1.750217067 |
| ELF3 | ATF3 | 1.737415475 |
| ELF3 | IGSF10 | 1.689461171 |
| ELF3 | PRELID1 | 1.688564555 |
| ELF3 | JAG2 | 1.686593882 |
| ELF3 | RPL39 | 1.663883638 |
| ELF3 | TXNIP | 1.58722424 |
| ELF3 | GNB2 | 1.580805021 |
| ELF3 | NUF2 | 1.523052468 |
| ELF3 | SLC46A3 | 1.496888162 |
| ELF3 | SOX13 | 1.492702345 |
| ELF3 | PIP4K2A | 1.445418256 |
| ELF3 | UBE4B | 1.404524997 |
| ELF3 | NTRK2 | 1.372912874 |
| ELF3 | DENND5B | 1.301146301 |
| ELF3 | TOB1 | 1.271345962 |
| ELF3 | TRIM4 | 1.266413745 |
| ELF3 | SCN4B | 1.251037092 |
| ELF3 | TEX264 | 1.235248312 |
| ELF3 | GRB10 | 1.219897758 |
| ELF3 | PTN | 1.218229047 |
| ELF3 | ATP6V0A1 | 1.209646233 |
| ELF3 | RB1CC1 | 1.190224823 |
| ELF3 | BAHCC1 | 1.187515967 |
| ELF3 | POLH | 1.141533079 |
| ELF3 | NCOA7 | 1.114421728 |
| ELF3 | TSHZ1 | 1.113324399 |
| ELF3 | CDKN1A | 1.113181299 |
| ELF3 | RNF150 | 1.089179952 |
| ELF3 | PHF6 | 1.082246583 |
| ELF3 | PDCD10 | 1.076567544 |
| ELF3 | LRRCC1 | 1.076358992 |
| ELF3 | AFAP1L2 | 1.043276803 |
| ELF3 | OSR1 | 1.035564349 |
| ELF3 | QSER1 | 1.034172889 |
| ELF3 | ANKRD11 | 1.033524636 |
| ELF3 | CD14 | 1.030551332 |
| ELF3 | RPS20 | 1.027844038 |
| ELF3 | ARL6IP5 | 1.01065793 |
| ELF3 | ELF3 | 1 |
| ELF3 | SLC20A1 | 0.997653758 |
| ELF3 | MCM7 | 0.993879955 |
| ELF3 | RAB7A | 0.973871558 |
| ELF3 | CAMTA1 | 0.963158919 |
| ELF3 | ZNF354A | 0.961603656 |
| ELF3 | TMEM223 | 0.938394415 |
| ELF3 | SRSF4 | 0.924570032 |
| ELF3 | UBR7 | 0.918916331 |
| ELF3 | C14orf93 | 0.877165564 |
| ELF3 | TBK1 | 0.848329402 |
| ELF3 | NFKBID | 0.844004022 |
| ELF3 | DAZAP2 | 0.834312502 |
| ELF3 | TRAF7 | 0.822696239 |
| ELF3 | HLA-DRA | 0.80951808 |
| ELF3 | LIMS1 | 0.800689711 |
| ELF3 | BPTF | 0.789621377 |
| ELF3 | EXOC3 | 0.782243389 |
| ELF3 | YIPF5 | 0.782160642 |
| ELF3 | MTG1 | 0.765706466 |
| ELF3 | TOP2B | 0.745517792 |
| ELF3 | ARCN1 | 0.732463629 |
| ELF3 | STAG1 | 0.726841937 |
| ELF3 | ACTR6 | 0.714869092 |
| ELF3 | MLLT10 | 0.69779933 |
| ELF3 | SLC1A5 | 0.691823258 |
| ELF3 | CASK | 0.682188027 |
| ELF3 | WDR36 | 0.674691722 |
| ELF3 | PLEKHG5 | 0.671964115 |
| ELF3 | SCARB1 | 0.666142503 |
| ELF3 | LTBP4 | 0.660468561 |
| ELF3 | ARF4 | 0.650794142 |
| ELF3 | LAMB1 | 0.645959076 |
| ELF3 | SNED1 | 0.638420468 |
| ELF3 | OSBPL5 | 0.6286223 |
| ELF3 | BMS1 | 0.624518948 |
| ELF3 | USP3 | 0.618247307 |
| ELF3 | SS18 | 0.615890911 |
| ELF3 | RNASEH1 | 0.605378082 |
| ELF3 | PDCD11 | 0.590881576 |
| ELF3 | AIP | 0.53791519 |
| ELF3 | RALGDS | 0.526743423 |
| ELF3 | TNFRSF1A | 0.506852859 |
| ELF3 | PRKAR1A | 0.488809913 |
| ELF3 | LSM14A | 0.482100912 |
| ELF3 | ITIH5 | 0.479429157 |
| ELF3 | ARL2BP | 0.470159985 |
| ELF3 | RBMS1 | 0.442904561 |
| ELF3 | CTTN | 0.423392035 |
| ELF3 | NFE2L2 | 0.401727841 |
| ELF3 | ESAM | 0.392329953 |
| ELF3 | FAM120B | 0.388065513 |
| ELF3 | GLCCI1 | 0.380113626 |
| ELF3 | MAP2 | 0.376715631 |
| ELF3 | CEP350 | 0.367234413 |
| ELF3 | NFKB2 | 0.344364127 |
| ELF3 | NDUFB3 | 0.322287194 |
| ELF3 | TSPAN14 | 0.311026375 |
| ELF3 | CHIC2 | 0.301537435 |
| ELF3 | PHLDB1 | 0.294489313 |
| ELF3 | PNP | 0.290256886 |
| ELF3 | TNIP2 | 0.288389011 |
| ELF3 | NSUN6 | 0.282465236 |
| ELF3 | TMEM199 | 0.27315397 |
| ELF3 | LRG1 | 0.271043048 |
| ELF3 | ZDHHC17 | 0.270252906 |
| ELF3 | MFAP1 | 0.269922425 |
| ELF3 | DCAF7 | 0.269007561 |
| ELF3 | BCL2L11 | 0.268782772 |
| ELF3 | PFDN6 | 0.264253451 |
| ELF3 | PRRG2 | 0.263718556 |
| ELF3 | OSMR | 0.25747143 |
| ELF3 | BTN3A3 | 0.253940828 |
| ELF3 | PHC2 | 0.253689188 |
| ELF3 | RP2 | 0.249500608 |
| ELF3 | TM4SF1 | 0.243501575 |
| ELF3 | SYTL2 | 0.237108786 |
| ELF3 | ROCK2 | 0.224193534 |
| ELF3 | MYH10 | 0.208566153 |
| ELF3 | ZC3H13 | 0.208368986 |
| ELF3 | ARHGEF7 | 0.202594689 |
| ELF3 | PLXNA1 | 0.198390111 |
| ELF3 | QPCT | 0.189380498 |
| ELF3 | SLC30A1 | 0.187109532 |
| ELF3 | STK40 | 0.186892007 |
| ELF3 | NCK2 | 0.182307807 |
| ELF3 | ZNF501 | 0.181997814 |
| ELF3 | POM121C | 0.181262538 |
| ELF3 | KTI12 | 0.180168786 |
| ELF3 | CSNK1A1 | 0.17787976 |
| ELF3 | ATP6V1A | 0.162225859 |
| ELF3 | PLCE1 | 0.160998337 |
| ELF3 | U2AF2 | 0.156441905 |
| ELF3 | DAXX | 0.156143435 |
| ELF3 | BCL9L | 0.142551322 |
| ELF3 | RARA | 0.142029927 |
| ELF3 | NFS1 | 0.14035279 |
| ELF3 | LAMC2 | 0.131410149 |
| ELF3 | LPIN3 | 0.126799799 |
| ELF3 | ST20 | 0.123325681 |
| ELF3 | ANKRD42 | 0.122455109 |
| ELF3 | ESR1 | 0.120469687 |
| ELF3 | BCL2L13 | 0.119734642 |
| ELF3 | LYRM1 | 0.106473988 |
| ELF3 | ERP27 | 0.104603565 |
| ELF3 | METTL6 | 0.089256962 |
| ELF3 | TGFBRAP1 | 0.070598972 |
| ELF3 | TPCN2 | 0.065304834 |
| ELF3 | ADSL | 0.048423266 |
| ELF3 | RIN2 | 1.26E-16 |
| ELF4 | SF1 | 6.596006827 |
| ELF4 | PHF21A | 4.147550018 |
| ELF4 | CCNL1 | 1.853226759 |
| ELF4 | POSTN | 1.269569355 |
| ELF4 | TCP1 | 1.184274811 |
| ELF4 | POLR2J | 0.988250393 |
| ELF4 | VPS13B | 0.762037429 |
| ELF4 | DARS | 0.688302027 |
| ELF4 | RPF2 | 0.670794121 |
| ELF4 | SF3B5 | 0.614426811 |
| ELF4 | USP38 | 0.518294337 |
| ELF4 | WDR1 | 0.509250636 |
| ELF4 | SIN3A | 0.503010451 |
| ELF4 | FAM49B | 0.487003375 |
| ELF4 | PPM1A | 0.48208077 |
| ELF4 | PHTF1 | 0.479120686 |
| ELF4 | TJAP1 | 0.301787509 |
| ELF4 | TRPT1 | 0.296768617 |
| ELF4 | TMEM33 | 0.286100911 |
| ELF4 | FAM19A5 | 0.246065387 |
| ELF4 | MYH10 | 0.243720122 |
| ELF4 | SOCS4 | 0.219487122 |
| ELF4 | AP1M1 | 0.182312876 |
| ELF4 | MTX3 | 0.164598781 |
| ELF4 | GALNT5 | 0.157013639 |
| ELF4 | TTF1 | 0.111723502 |
| ELF4 | PRKCH | 0.060785845 |
| ELK1 | ESYT1 | 8.044281667 |
| ELK1 | PRCC | 5.170045777 |
| ELK1 | TMEM200A | 3.992961194 |
| ELK1 | CTTN | 3.734917045 |
| ELK1 | KIF11 | 2.666741595 |
| ELK1 | UTP20 | 2.573596513 |
| ELK1 | NUP155 | 2.455834405 |
| ELK1 | NFKBIZ | 2.393513272 |
| ELK1 | ALCAM | 2.39065034 |
| ELK1 | PSEN1 | 2.333939936 |
| ELK1 | LMAN2L | 2.285874101 |
| ELK1 | SLC25A12 | 2.101829121 |
| ELK1 | SFSWAP | 2.09120946 |
| ELK1 | NCOA2 | 1.935725706 |
| ELK1 | HBEGF | 1.853038418 |
| ELK1 | SLC2A13 | 1.64654065 |
| ELK1 | ABCB7 | 1.621928199 |
| ELK1 | TMEM209 | 1.614484558 |
| ELK1 | SUPT3H | 1.574318018 |
| ELK1 | RNF114 | 1.523011089 |
| ELK1 | TIPARP | 1.496288043 |
| ELK1 | UBE2A | 1.493254017 |
| ELK1 | SAA1 | 1.448152652 |
| ELK1 | LCMT1 | 1.430239145 |
| ELK1 | DDX23 | 1.394944836 |
| ELK1 | HNRNPAB | 1.394412037 |
| ELK1 | BEX5 | 1.391546679 |
| ELK1 | MRPS22 | 1.376337406 |
| ELK1 | INO80E | 1.348326632 |
| ELK1 | PHF21A | 1.305690887 |
| ELK1 | ABCF3 | 1.238274954 |
| ELK1 | CDH5 | 1.232456406 |
| ELK1 | HDAC11 | 1.225212867 |
| ELK1 | NADK | 1.156396727 |
| ELK1 | AP4B1 | 1.144194145 |
| ELK1 | ANAPC7 | 1.120920215 |
| ELK1 | ABCC1 | 1.117282527 |
| ELK1 | XYLB | 1.08462559 |
| ELK1 | CNKSR2 | 1.074821791 |
| ELK1 | SATB2 | 1.046694945 |
| ELK1 | ELK1 | 1 |
| ELK1 | SLC20A2 | 0.969596401 |
| ELK1 | UBIAD1 | 0.966113534 |
| ELK1 | FKBP14 | 0.958161561 |
| ELK1 | CLSPN | 0.93832962 |
| ELK1 | CPNE5 | 0.935759555 |
| ELK1 | UBE2J2 | 0.929867589 |
| ELK1 | SMC2 | 0.908743202 |
| ELK1 | TTC28 | 0.897811828 |
| ELK1 | ZFAND5 | 0.884101606 |
| ELK1 | CAMK1 | 0.865610214 |
| ELK1 | COPS5 | 0.855926616 |
| ELK1 | ARSA | 0.851123817 |
| ELK1 | KIF26B | 0.82441712 |
| ELK1 | PRPS2 | 0.800154832 |
| ELK1 | RNF187 | 0.796667607 |
| ELK1 | NOC3L | 0.775442328 |
| ELK1 | SAP30L | 0.769410587 |
| ELK1 | ZNF561 | 0.748934671 |
| ELK1 | EDIL3 | 0.730549361 |
| ELK1 | ALG2 | 0.713740516 |
| ELK1 | HOXD3 | 0.689259411 |
| ELK1 | AZIN1 | 0.681577319 |
| ELK1 | CD47 | 0.679063996 |
| ELK1 | F3 | 0.66066528 |
| ELK1 | JRKL | 0.660575591 |
| ELK1 | FBXL20 | 0.659588066 |
| ELK1 | CCDC51 | 0.652507608 |
| ELK1 | OTUD4 | 0.649221631 |
| ELK1 | JUND | 0.635385114 |
| ELK1 | PDE4D | 0.619940524 |
| ELK1 | ZNRF3 | 0.617547056 |
| ELK1 | ZNF296 | 0.61566179 |
| ELK1 | CX3CL1 | 0.611361622 |
| ELK1 | LENG9 | 0.610231916 |
| ELK1 | CDK7 | 0.609972492 |
| ELK1 | PCNA | 0.60399158 |
| ELK1 | GOLGA5 | 0.60346309 |
| ELK1 | FCF1 | 0.579339058 |
| ELK1 | FAM109B | 0.567932726 |
| ELK1 | BRCA1 | 0.544994528 |
| ELK1 | TNKS | 0.536355921 |
| ELK1 | JMJD1C | 0.526101793 |
| ELK1 | GTF3C2 | 0.526093345 |
| ELK1 | ZFHX3 | 0.525398821 |
| ELK1 | CHCHD6 | 0.524123335 |
| ELK1 | CTDNEP1 | 0.515455984 |
| ELK1 | SH3KBP1 | 0.50845993 |
| ELK1 | UTP23 | 0.505013446 |
| ELK1 | EIF4A2 | 0.504465765 |
| ELK1 | RPRM | 0.504113553 |
| ELK1 | LOXL2 | 0.501875711 |
| ELK1 | VAPB | 0.497928047 |
| ELK1 | ATP6V0B | 0.471245901 |
| ELK1 | ASCC1 | 0.471056905 |
| ELK1 | SRRM2 | 0.468853786 |
| ELK1 | GTSE1 | 0.467651995 |
| ELK1 | PSMD6 | 0.465987509 |
| ELK1 | TRIM7 | 0.460136211 |
| ELK1 | NCAPH2 | 0.45605244 |
| ELK1 | ROBO1 | 0.454846853 |
| ELK1 | TRAPPC6B | 0.444117874 |
| ELK1 | YEATS4 | 0.438141581 |
| ELK1 | BUB3 | 0.426137885 |
| ELK1 | MRPS31 | 0.419094138 |
| ELK1 | PER2 | 0.418973975 |
| ELK1 | IQGAP1 | 0.418852792 |
| ELK1 | KITLG | 0.418054581 |
| ELK1 | DENND4C | 0.417126496 |
| ELK1 | FEN1 | 0.413115005 |
| ELK1 | SAMD8 | 0.412475936 |
| ELK1 | AP1G1 | 0.408849789 |
| ELK1 | THRB | 0.40774111 |
| ELK1 | CKAP2L | 0.404047461 |
| ELK1 | NKAP | 0.394263125 |
| ELK1 | GOSR2 | 0.372567135 |
| ELK1 | M6PR | 0.364832154 |
| ELK1 | SYAP1 | 0.348259807 |
| ELK1 | FBL | 0.337728334 |
| ELK1 | SLC30A9 | 0.334093237 |
| ELK1 | PDZD8 | 0.330207064 |
| ELK1 | DERL3 | 0.328506927 |
| ELK1 | PIGF | 0.315933921 |
| ELK1 | NGRN | 0.31430187 |
| ELK1 | XPO1 | 0.310895509 |
| ELK1 | PSD3 | 0.308717313 |
| ELK1 | LPCAT1 | 0.308655114 |
| ELK1 | NUP107 | 0.290648014 |
| ELK1 | SPPL3 | 0.288318968 |
| ELK1 | ABI2 | 0.274180553 |
| ELK1 | WAC | 0.270771381 |
| ELK1 | DAP3 | 0.267203955 |
| ELK1 | IPO5 | 0.266532972 |
| ELK1 | RAB18 | 0.266469212 |
| ELK1 | LRRC47 | 0.264458322 |
| ELK1 | PTER | 0.247602102 |
| ELK1 | N4BP2L1 | 0.242431665 |
| ELK1 | MAU2 | 0.241566312 |
| ELK1 | CDV3 | 0.238768384 |
| ELK1 | CDKL3 | 0.227089432 |
| ELK1 | ZMYM1 | 0.22330276 |
| ELK1 | TMEM187 | 0.222917372 |
| ELK1 | BBS2 | 0.220951538 |
| ELK1 | PFKP | 0.219119766 |
| ELK1 | NXT1 | 0.218836478 |
| ELK1 | CHST11 | 0.216552537 |
| ELK1 | ARHGEF6 | 0.21528649 |
| ELK1 | SLC19A2 | 0.201644589 |
| ELK1 | GNB4 | 0.201570441 |
| ELK1 | EGOT | 0.198104979 |
| ELK1 | C3orf38 | 0.195193117 |
| ELK1 | FMR1 | 0.190807608 |
| ELK1 | GRIK1 | 0.190218742 |
| ELK1 | NR1H2 | 0.188819517 |
| ELK1 | GCHFR | 0.185447535 |
| ELK1 | H6PD | 0.184759595 |
| ELK1 | KLF13 | 0.182839893 |
| ELK1 | NUAK1 | 0.176977433 |
| ELK1 | KLHDC10 | 0.175500686 |
| ELK1 | EIF3B | 0.173341248 |
| ELK1 | DHX8 | 0.172607216 |
| ELK1 | CUL4A | 0.168862169 |
| ELK1 | C21orf58 | 0.167228434 |
| ELK1 | THRA | 0.162315493 |
| ELK1 | UBR7 | 0.158457091 |
| ELK1 | PIGK | 0.156328506 |
| ELK1 | LYPLA2 | 0.154407979 |
| ELK1 | GFPT2 | 0.153364037 |
| ELK1 | MYO1E | 0.145233059 |
| ELK1 | C2orf69 | 0.142719038 |
| ELK1 | UBA1 | 0.140320026 |
| ELK1 | MAGOHB | 0.13966099 |
| ELK1 | ETFA | 0.136464229 |
| ELK1 | ESYT2 | 0.12843583 |
| ELK1 | MRPL44 | 0.124632497 |
| ELK1 | B3GALT4 | 0.122665921 |
| ELK1 | NUBP1 | 0.111508035 |
| ELK1 | USP38 | 0.110819678 |
| ELK1 | POLR2D | 0.109686636 |
| ELK1 | PLAT | 0.109020625 |
| ELK1 | PORCN | 0.106741466 |
| ELK1 | FBXW4 | 0.102990941 |
| ELK1 | ZFP37 | 0.097382251 |
| ELK1 | NACC1 | 0.092531588 |
| ELK1 | SLC9A1 | 0.080868122 |
| ELK1 | FASN | 0.080555759 |
| ELK1 | GPR155 | 0.060218295 |
| ELK1 | RNF25 | 0.05786152 |
| ELK1 | ZC3H3 | 0.054965563 |
| ELK1 | MTOR | 0.049361369 |
| ELK1 | PANK4 | 0.048131433 |
| ELK1 | STK32B | 0.043473865 |
| ELK1 | C8orf44 | 0.042352006 |
| ELK1 | TMEM62 | 0.017639698 |
| ELK1 | FAM133A | 0.016881294 |
| ELK3 | NDUFB11 | 3.258223705 |
| ELK3 | SPATS2 | 2.84888014 |
| ELK3 | PPA1 | 2.847843399 |
| ELK3 | TBPL1 | 2.121578066 |
| ELK3 | HOXC10 | 1.529886154 |
| ELK3 | GJA4 | 1.316828346 |
| ELK3 | ZNF787 | 1.262108649 |
| ELK3 | PPP1R12C | 1.256444472 |
| ELK3 | PTPN12 | 1.166868409 |
| ELK3 | USP38 | 1.160861046 |
| ELK3 | ZNF41 | 1.148998883 |
| ELK3 | MRPL33 | 1.096919723 |
| ELK3 | SNRPB2 | 1.096440559 |
| ELK3 | WISP2 | 1.033886714 |
| ELK3 | FBLN2 | 1.027611581 |
| ELK3 | PALM2 | 1.022034354 |
| ELK3 | NHSL1 | 1.016396323 |
| ELK3 | PDPN | 1.013267699 |
| ELK3 | ZRANB3 | 1.00756916 |
| ELK3 | RPL23 | 1.004672749 |
| ELK3 | ELK3 | 1 |
| ELK3 | FBXL20 | 0.984192642 |
| ELK3 | NDUFS8 | 0.978343666 |
| ELK3 | ORC5 | 0.976930993 |
| ELK3 | RPS6KC1 | 0.925001515 |
| ELK3 | MRRF | 0.901633585 |
| ELK3 | ERCC6 | 0.868851843 |
| ELK3 | GTF2F1 | 0.848660295 |
| ELK3 | CTTNBP2NL | 0.801322097 |
| ELK3 | MYL12A | 0.799581369 |
| ELK3 | ABHD2 | 0.794491998 |
| ELK3 | CALD1 | 0.78401614 |
| ELK3 | ERH | 0.769910627 |
| ELK3 | EMP1 | 0.763521042 |
| ELK3 | STK17A | 0.741064395 |
| ELK3 | SDCBP | 0.727851916 |
| ELK3 | TMEM150C | 0.723481123 |
| ELK3 | APLP2 | 0.70246947 |
| ELK3 | RPS5 | 0.696173764 |
| ELK3 | TNIP1 | 0.693327093 |
| ELK3 | SLCO3A1 | 0.691224843 |
| ELK3 | ZNFX1 | 0.676711922 |
| ELK3 | ATG7 | 0.67342407 |
| ELK3 | CBFA2T3 | 0.671358223 |
| ELK3 | SDC3 | 0.65691156 |
| ELK3 | MYH11 | 0.655004136 |
| ELK3 | IGFBP4 | 0.650988273 |
| ELK3 | GORASP2 | 0.650520482 |
| ELK3 | XPO1 | 0.615004026 |
| ELK3 | STC1 | 0.611807614 |
| ELK3 | WRB | 0.594487452 |
| ELK3 | MTUS1 | 0.591915012 |
| ELK3 | RPS7 | 0.588052736 |
| ELK3 | C9orf40 | 0.577876257 |
| ELK3 | RNMT | 0.568922948 |
| ELK3 | RPS16 | 0.558380639 |
| ELK3 | PDAP1 | 0.558003776 |
| ELK3 | RPS14 | 0.557590729 |
| ELK3 | CDKL3 | 0.556279671 |
| ELK3 | HSP90AB1 | 0.543174099 |
| ELK3 | C8orf44 | 0.543044577 |
| ELK3 | GNAI2 | 0.542935406 |
| ELK3 | ACTG1 | 0.531306851 |
| ELK3 | PEA15 | 0.529061089 |
| ELK3 | PALMD | 0.520275922 |
| ELK3 | ZDHHC1 | 0.519566584 |
| ELK3 | GDPD5 | 0.518449231 |
| ELK3 | ARL6IP5 | 0.508143287 |
| ELK3 | PACRG | 0.506248913 |
| ELK3 | CXorf57 | 0.497682659 |
| ELK3 | RPL10A | 0.492983444 |
| ELK3 | ARL6 | 0.47621264 |
| ELK3 | GBAP1 | 0.471198733 |
| ELK3 | ABCE1 | 0.469191775 |
| ELK3 | QARS | 0.461983789 |
| ELK3 | TSC22D1 | 0.444042012 |
| ELK3 | RPS21 | 0.441985462 |
| ELK3 | PIK3R1 | 0.434156548 |
| ELK4 | HNRNPD | 4.09043536 |
| ELK4 | TIAL1 | 4.010619199 |
| ELK4 | C17orf97 | 3.749339454 |
| ELK4 | RBMS1 | 3.422612185 |
| ELK4 | MAU2 | 2.385416561 |
| ELK4 | ZNF766 | 2.369719792 |
| ELK4 | ZNF444 | 2.119397544 |
| ELK4 | ARRDC2 | 1.846777397 |
| ELK4 | HOXB6 | 1.786050606 |
| ELK4 | PRKG1 | 1.725691063 |
| ELK4 | MAGED1 | 1.700729002 |
| ELK4 | ZRANB1 | 1.626931287 |
| ELK4 | LMF2 | 1.610501321 |
| ELK4 | EIF2B2 | 1.502014485 |
| ELK4 | CALM3 | 1.438526146 |
| ELK4 | SLC40A1 | 1.434328071 |
| ELK4 | RGMB | 1.406987452 |
| ELK4 | ULK2 | 1.401184278 |
| ELK4 | NFYC | 1.388266836 |
| ELK4 | NFE2L2 | 1.345219702 |
| ELK4 | ZFX | 1.331715698 |
| ELK4 | PRPF39 | 1.31918823 |
| ELK4 | H2AFY | 1.317122176 |
| ELK4 | PRPF6 | 1.249347804 |
| ELK4 | TBCB | 1.057931755 |
| ELK4 | ADAMTS9 | 1.002345026 |
| ELK4 | ELK4 | 1 |
| ELK4 | TMED2 | 0.998409847 |
| ELK4 | LAMC1 | 0.919851695 |
| ELK4 | JUND | 0.906975307 |
| ELK4 | JMJD8 | 0.904738554 |
| ELK4 | PLCG1 | 0.88240904 |
| ELK4 | SEC11C | 0.808704008 |
| ELK4 | UBE2D3 | 0.805158944 |
| ELK4 | EXT2 | 0.795743171 |
| ELK4 | SLC12A2 | 0.795264669 |
| ELK4 | RREB1 | 0.794896656 |
| ELK4 | SLC39A14 | 0.763701962 |
| ELK4 | UBE4B | 0.737182947 |
| ELK4 | MICAL2 | 0.707808932 |
| ELK4 | WASL | 0.694743072 |
| ELK4 | RALGDS | 0.658964419 |
| ELK4 | OGT | 0.641469413 |
| ELK4 | SMG1 | 0.59333188 |
| ELK4 | G0S2 | 0.59033078 |
| ELK4 | SPSB3 | 0.586547679 |
| ELK4 | PFN2 | 0.584340119 |
| ELK4 | PRR5 | 0.579723606 |
| ELK4 | TTPAL | 0.569694264 |
| ELK4 | SMAD1 | 0.563603093 |
| ELK4 | MGLL | 0.51317898 |
| ELK4 | SDF4 | 0.502524797 |
| ELK4 | TFIP11 | 0.497485058 |
| ELK4 | FLYWCH2 | 0.474922551 |
| ELK4 | GPX4 | 0.470968635 |
| ELK4 | TOR1AIP2 | 0.450961191 |
| ELK4 | PPP3R1 | 0.448138123 |
| ELK4 | PAX1 | 0.436029659 |
| ELK4 | SH3KBP1 | 0.434686549 |
| ELK4 | STK4 | 0.422277066 |
| ELK4 | CAPN7 | 0.391882621 |
| ELK4 | SH2B3 | 0.321838064 |
| ELK4 | SLC39A8 | 0.317198563 |
| ELK4 | SLITRK4 | 0.288321302 |
| ELK4 | NT5DC2 | 0.263382746 |
| ELK4 | ATXN2L | 0.239792769 |
| ELK4 | KLF11 | 0.227099068 |
| ELK4 | RELB | 0.205968725 |
| ELK4 | B3GALT6 | 0.135937845 |
| ELK4 | CLCN7 | 0.127566438 |
| ELK4 | AKAP11 | 0.123859953 |
| ELK4 | TEF | 0.118227299 |
| ELK4 | UVRAG | 0.113241034 |
| ELK4 | HOXA5 | 0.107251012 |
| ELK4 | RNASEK | 0.056439834 |
| ELK4 | CNTNAP1 | 0.054180534 |
| ELK4 | RFESD | 0.034081182 |
| ELK4 | ESF1 | 0.000133731 |
| EP300 | ITM2A | 6.183162398 |
| EP300 | TTC1 | 4.868649438 |
| EP300 | PPP1R16A | 3.234107374 |
| EP300 | NFX1 | 3.202176812 |
| EP300 | C20orf27 | 3.135236807 |
| EP300 | CCDC25 | 3.114674217 |
| EP300 | IDH3G | 2.946768118 |
| EP300 | LSM4 | 2.694519869 |
| EP300 | HMGN5 | 2.553764799 |
| EP300 | PRKACA | 2.544147497 |
| EP300 | AHDC1 | 2.142040488 |
| EP300 | ISY1 | 2.062057991 |
| EP300 | MAGOHB | 1.934556531 |
| EP300 | PEF1 | 1.907742637 |
| EP300 | MFGE8 | 1.903750237 |
| EP300 | STOML2 | 1.897239848 |
| EP300 | ACBD3 | 1.859564271 |
| EP300 | NOV | 1.832994354 |
| EP300 | CREB3 | 1.815106318 |
| EP300 | PRELID1 | 1.812698721 |
| EP300 | ABHD5 | 1.78151101 |
| EP300 | CEP63 | 1.772088054 |
| EP300 | MOGS | 1.759436519 |
| EP300 | CLASP1 | 1.684523055 |
| EP300 | DGKZ | 1.672473383 |
| EP300 | DYNC2LI1 | 1.582004024 |
| EP300 | YWHAE | 1.581154255 |
| EP300 | CCDC12 | 1.550707299 |
| EP300 | LTB4R | 1.540232423 |
| EP300 | MICALL1 | 1.538409417 |
| EP300 | IVL | 1.517852597 |
| EP300 | BMI1 | 1.512907464 |
| EP300 | ANKS3 | 1.488899542 |
| EP300 | SUPV3L1 | 1.465786762 |
| EP300 | STK17B | 1.458253426 |
| EP300 | SPRED1 | 1.447063658 |
| EP300 | CPA3 | 1.426336247 |
| EP300 | GNA15 | 1.396454462 |
| EP300 | IGF2R | 1.393729806 |
| EP300 | BAG3 | 1.360050361 |
| EP300 | SULF2 | 1.323898178 |
| EP300 | WDR35 | 1.305708708 |
| EP300 | CDKN2A | 1.260619196 |
| EP300 | TBC1D22B | 1.258601296 |
| EP300 | MCOLN1 | 1.255598609 |
| EP300 | PNKD | 1.2507916 |
| EP300 | IGFBP4 | 1.230559008 |
| EP300 | GOLGA7 | 1.168878472 |
| EP300 | ANPEP | 1.14568939 |
| EP300 | CHST3 | 1.135929875 |
| EP300 | NIPBL | 1.132622415 |
| EP300 | DCTN3 | 1.080147154 |
| EP300 | PTGFR | 1.074557541 |
| EP300 | IKBKE | 1.063196005 |
| EP300 | VCAN | 1.03766958 |
| EP300 | RPN2 | 1.034897549 |
| EP300 | RPL34 | 1.034464348 |
| EP300 | BRPF3 | 1.034395887 |
| EP300 | PSMC3 | 1.027512015 |
| EP300 | ITGB5 | 1.027482324 |
| EP300 | P2RY1 | 1.024764692 |
| EP300 | SRSF5 | 0.968688438 |
| EP300 | TMBIM6 | 0.916777557 |
| EP300 | TMEM69 | 0.585481168 |
| EP300 | TRAPPC8 | 0.573294575 |
| EP300 | ELF2 | 0.571268362 |
| EP300 | LRRC1 | 0.537613004 |
| EP300 | TFG | 0.534520007 |
| EP300 | HNRNPA0 | 0.511077789 |
| EP300 | PAQR5 | 0.493114947 |
| EP300 | POGZ | 0.486006407 |
| EP300 | SAMD8 | 0.472134332 |
| EP300 | PSMA5 | 0.452544504 |
| EP300 | TLE3 | 0.441162567 |
| EP300 | COL24A1 | 0.359904209 |
| EP300 | UBN2 | 0.343550565 |
| EP300 | RARB | 0.335271342 |
| EP300 | SH3BP5L | 0.320246106 |
| EP300 | KITLG | 0.318652557 |
| EP300 | LZTR1 | 0.30534163 |
| EP300 | SCYL3 | 0.300133275 |
| EP300 | C6orf120 | 0.286013385 |
| EP300 | TMEM128 | 0.282496137 |
| EP300 | MGAT1 | 0.270528075 |
| EP300 | WDR45 | 0.241210435 |
| EP300 | TXNL4B | 0.213044995 |
| EP300 | CSNK1A1 | 0.203529766 |
| EP300 | TFB2M | 0.199688711 |
| EP300 | PPP1R10 | 0.199105654 |
| EP300 | CHRNB1 | 0.198733745 |
| EP300 | ELP2 | 0.173281225 |
| EP300 | RAP2C | 0.154250032 |
| EP300 | ANKS1A | 0.143266641 |
| EP300 | CHUK | 0.062765696 |
| EP300 | SLCO4A1 | 0.020835775 |
| EP300 | TAOK2 | 0.011167175 |
| EP300 | TOR1B | 1.82E-19 |
| ERF | CKAP2L | 14.37824169 |
| ERF | TTC37 | 9.271231395 |
| ERF | DLGAP5 | 5.662433502 |
| ERF | KLF10 | 3.135683893 |
| ERF | TOP2A | 3.085973627 |
| ERF | UBE2N | 2.262888112 |
| ERF | JTB | 2.232044103 |
| ERF | GAST | 2.225015724 |
| ERF | ETV7 | 2.092362702 |
| ERF | CHCHD2 | 1.954672823 |
| ERF | CDC42EP3 | 1.877568257 |
| ERF | ATP11A | 1.860161972 |
| ERF | CERCAM | 1.791127996 |
| ERF | C5orf46 | 1.786324347 |
| ERF | RABEP2 | 1.734824023 |
| ERF | RPSA | 1.728238674 |
| ERF | XRCC2 | 1.610625597 |
| ERF | TECPR2 | 1.599083543 |
| ERF | MRPL51 | 1.589989155 |
| ERF | NR4A1 | 1.472354904 |
| ERF | DCK | 1.451839875 |
| ERF | CCND1 | 1.428880762 |
| ERF | MARS | 1.406314983 |
| ERF | TRAM1 | 1.401433819 |
| ERF | GFM2 | 1.377401315 |
| ERF | HSPBP1 | 1.30452113 |
| ERF | RPL15 | 1.288704285 |
| ERF | CNN3 | 1.281726645 |
| ERF | ITM2B | 1.265198622 |
| ERF | C1QTNF6 | 1.24192085 |
| ERF | ITIH5 | 1.239874294 |
| ERF | GLI2 | 1.234621847 |
| ERF | IMPA2 | 1.227439117 |
| ERF | HSPE1 | 1.197328219 |
| ERF | SPCS3 | 1.097919897 |
| ERF | TLE1 | 1.090292193 |
| ERF | CWF19L2 | 1.085746644 |
| ERF | PUSL1 | 1.062930121 |
| ERF | GTF2B | 1.048254585 |
| ERF | IRF1 | 1.026562349 |
| ERF | SUCLG1 | 1.024352977 |
| ERF | RTN4RL2 | 1.020797191 |
| ERF | GFOD2 | 1.01975072 |
| ERF | TAPBP | 1.010232101 |
| ERF | ERF | 1 |
| ERF | HSP90AA1 | 0.965547744 |
| ERF | PTGES3 | 0.963510445 |
| ERF | CTNNA1 | 0.93763705 |
| ERF | CYP1B1 | 0.932397092 |
| ERF | MRPL54 | 0.931709657 |
| ERF | SPTBN2 | 0.917240196 |
| ERF | PHF21A | 0.904227464 |
| ERF | MYL6B | 0.898533114 |
| ERF | G3BP1 | 0.890557189 |
| ERF | RRP15 | 0.887057069 |
| ERF | HLA-B | 0.880071972 |
| ERF | NIPA2 | 0.866328211 |
| ERF | MRPL16 | 0.845471624 |
| ERF | PLK2 | 0.840010955 |
| ERF | PPIC | 0.83848155 |
| ERF | ETV4 | 0.828568692 |
| ERF | PITPNA | 0.818910978 |
| ERF | HBP1 | 0.818659457 |
| ERF | LLGL2 | 0.79320703 |
| ERF | ERRFI1 | 0.787980517 |
| ERF | ZNF143 | 0.776507693 |
| ERF | PSMB7 | 0.766800916 |
| ERF | ZNF546 | 0.731623839 |
| ERF | BCL3 | 0.724670453 |
| ERF | HEXIM1 | 0.716552786 |
| ERF | CCDC47 | 0.712144941 |
| ERF | MED27 | 0.704874124 |
| ERF | EXOSC1 | 0.699222264 |
| ERF | RSRC2 | 0.695290059 |
| ERF | NAAA | 0.682539092 |
| ERF | RPL19 | 0.681180992 |
| ERF | RPS7 | 0.678147663 |
| ERF | GLYR1 | 0.665528136 |
| ERF | UBL5 | 0.655921801 |
| ERF | DCP2 | 0.654201504 |
| ERF | TP63 | 0.644562976 |
| ERF | ZDHHC18 | 0.642822811 |
| ERF | SLC39A14 | 0.641737167 |
| ERF | MTF1 | 0.637177449 |
| ERF | COX6A1 | 0.622840608 |
| ERF | MAD2L2 | 0.615327686 |
| ERF | LHPP | 0.615194916 |
| ERF | VPS11 | 0.604310711 |
| ERF | NUP107 | 0.592122461 |
| ERF | ZNF594 | 0.587441463 |
| ERF | AKT1S1 | 0.584010243 |
| ERF | POLR2G | 0.581166604 |
| ERF | TPRKB | 0.573792503 |
| ERF | MTDH | 0.559379088 |
| ERF | SGTA | 0.534608824 |
| ERF | ERH | 0.529851661 |
| ERF | POU2F2 | 0.515972011 |
| ERF | FGD5 | 0.510514195 |
| ERF | COCH | 0.509020286 |
| ERF | CCNH | 0.508914888 |
| ERF | SRSF10 | 0.494541775 |
| ERF | NDUFS3 | 0.490864355 |
| ERF | TPM1 | 0.484345736 |
| ERF | CHEK1 | 0.474883495 |
| ERF | IL1R1 | 0.474841517 |
| ERF | KLHL2 | 0.459930172 |
| ERF | DNAJC10 | 0.457856929 |
| ERF | EZR | 0.455801421 |
| ERF | KLHL29 | 0.448505486 |
| ERF | CFL1 | 0.443687736 |
| ERF | VPS26A | 0.441211233 |
| ERF | IDH3B | 0.424367983 |
| ERF | CGGBP1 | 0.423765026 |
| ERF | KIF14 | 0.423142713 |
| ERF | SH2B2 | 0.421604159 |
| ERF | ARL6 | 0.409653144 |
| ERF | ADCY3 | 0.408958237 |
| ERF | CLSPN | 0.406795638 |
| ERF | UNC50 | 0.400019323 |
| ERF | KDELC1 | 0.398906547 |
| ERF | EFNA4 | 0.391853711 |
| ERF | OLFM2 | 0.379519668 |
| ERF | SAP30 | 0.377520115 |
| ERF | COL6A3 | 0.367761036 |
| ERF | DALRD3 | 0.365908426 |
| ERF | ZC3HAV1 | 0.364393066 |
| ERF | CREM | 0.351742583 |
| ERF | PAPD7 | 0.34850331 |
| ERF | STK4 | 0.34360594 |
| ERF | PIP5K1A | 0.343211509 |
| ERF | STEAP2 | 0.341018705 |
| ERF | FEM1A | 0.33983531 |
| ERF | ADAMTS8 | 0.338349782 |
| ERF | CCDC102A | 0.334264063 |
| ERF | SEMA6B | 0.327205708 |
| ERF | HIST1H1A | 0.326277779 |
| ERF | IL1RN | 0.324769223 |
| ERF | PLD2 | 0.319449805 |
| ERF | TSEN34 | 0.315443311 |
| ERF | TXNDC16 | 0.312670476 |
| ERF | ENTPD4 | 0.311194054 |
| ERF | MBNL3 | 0.311171837 |
| ERF | GPN1 | 0.309636469 |
| ERF | TAF11 | 0.309544549 |
| ERF | SHROOM1 | 0.306595589 |
| ERF | WDR4 | 0.304375544 |
| ERF | WDR83 | 0.304078025 |
| ERF | NFKBIB | 0.303115529 |
| ERF | RCC2 | 0.300502702 |
| ERF | FBXW4 | 0.297434613 |
| ERF | JOSD1 | 0.296693941 |
| ERF | LRRC58 | 0.29627368 |
| ERF | MCTP1 | 0.295532571 |
| ERF | FBXO18 | 0.295529357 |
| ERF | CCL23 | 0.292327029 |
| ERF | HIST2H2AB | 0.291510067 |
| ERF | DAZAP1 | 0.291482886 |
| ERF | STX18 | 0.284265947 |
| ERF | MAN2A2 | 0.281561254 |
| ERF | CACNB1 | 0.275709739 |
| ERF | ZFC3H1 | 0.273042513 |
| ERF | USP53 | 0.27224053 |
| ERF | NUDT5 | 0.269831841 |
| ERF | LAGE3 | 0.269427767 |
| ERF | GAPVD1 | 0.266366341 |
| ERF | MAPKAPK2 | 0.265556273 |
| ERF | DIDO1 | 0.262214574 |
| ERF | PELI1 | 0.261855676 |
| ERF | DLC1 | 0.261572352 |
| ERF | RANBP2 | 0.258242915 |
| ERF | RNGTT | 0.255518931 |
| ERF | PARN | 0.252755022 |
| ERF | AEN | 0.249779656 |
| ERF | GMCL1 | 0.249117015 |
| ERF | MBD4 | 0.248077897 |
| ERF | RSPO1 | 0.247977321 |
| ERF | RFC4 | 0.243572095 |
| ERF | MAB21L2 | 0.241721915 |
| ERF | C11orf49 | 0.24100111 |
| ERF | OTUD7B | 0.23896229 |
| ERF | ENOSF1 | 0.237804629 |
| ERF | SPG21 | 0.236483621 |
| ERF | UACA | 0.236200883 |
| ERF | UNC5B | 0.234688582 |
| ERF | AP3D1 | 0.234057693 |
| ERF | CEP70 | 0.230901382 |
| ERF | GEN1 | 0.228172212 |
| ERF | DGKE | 0.227220335 |
| ERF | BDKRB2 | 0.227000202 |
| ERF | PIP4K2B | 0.225881469 |
| ERF | TRAM2 | 0.223849137 |
| ERF | ANP32E | 0.220833022 |
| ERF | NPC1 | 0.219747032 |
| ERF | ZNF704 | 0.219515453 |
| ERF | VTRNA1-1 | 0.218983617 |
| ERF | CHD2 | 0.218036089 |
| ERF | CD163L1 | 0.212899806 |
| ERF | LTV1 | 0.211796421 |
| ERF | CMAS | 0.207216981 |
| ERF | PI4KA | 0.207013385 |
| ERF | CRYL1 | 0.207002872 |
| ERF | GLG1 | 0.206096614 |
| ERF | UBL7 | 0.205445957 |
| ERF | BBX | 0.204518994 |
| ERF | INTS9 | 0.204084262 |
| ERF | SEC23IP | 0.203246733 |
| ERF | BBS1 | 0.20144662 |
| ERF | ESYT1 | 0.198365857 |
| ERF | FUT8 | 0.196613265 |
| ERF | SNX19 | 0.194904737 |
| ERF | SNN | 0.194809583 |
| ERF | DERA | 0.194222485 |
| ERF | BCL6 | 0.193868582 |
| ERF | TBCE | 0.193177995 |
| ERF | COPS5 | 0.189427838 |
| ERF | SHOX2 | 0.189316018 |
| ERF | CCDC6 | 0.187201314 |
| ERF | ETFB | 0.186188343 |
| ERF | MED7 | 0.184915614 |
| ERF | M6PR | 0.183419552 |
| ERF | MAP2K5 | 0.182528687 |
| ERF | UBR4 | 0.182138683 |
| ERF | SASH1 | 0.18027191 |
| ERF | HLCS | 0.180093131 |
| ERF | COMMD10 | 0.179011453 |
| ERF | DNAJC1 | 0.178858199 |
| ERF | IKZF5 | 0.178730495 |
| ERF | HAAO | 0.178384563 |
| ERF | SAP30L | 0.173111718 |
| ERF | AK4 | 0.170630438 |
| ERF | ESRRA | 0.170334149 |
| ERF | F12 | 0.168284427 |
| ERF | ANKRD37 | 0.166349021 |
| ERF | DAXX | 0.165518536 |
| ERF | PYGO1 | 0.160984479 |
| ERF | HOPX | 0.153641312 |
| ERF | PDLIM5 | 0.153241783 |
| ERF | MTOR | 0.152325333 |
| ERF | PTPN9 | 0.150680207 |
| ERF | OGFOD1 | 0.149256952 |
| ERF | ADAR | 0.147409181 |
| ERF | CENPM | 0.146671352 |
| ERF | RHOH | 0.145834265 |
| ERF | SCLY | 0.14191514 |
| ERF | NBEAL2 | 0.139826599 |
| ERF | DNAL1 | 0.139765297 |
| ERF | GIT1 | 0.135986051 |
| ERF | ILKAP | 0.135066107 |
| ERF | PITX2 | 0.131748601 |
| ERF | F11R | 0.130896233 |
| ERF | SMAP2 | 0.123084753 |
| ERF | ZNF544 | 0.122454064 |
| ERF | SENP8 | 0.112522719 |
| ERF | BLOC1S3 | 0.111925416 |
| ERF | WDR25 | 0.110494264 |
| ERF | THUMPD2 | 0.107386414 |
| ERF | RNF20 | 0.104773531 |
| ERF | B2M | 0.101348342 |
| ERF | EIF4ENIF1 | 0.090970378 |
| ERF | PPIL1 | 0.089174986 |
| ERF | ZNF394 | 0.088407681 |
| ERF | LASP1 | 0.086550478 |
| ERF | ABCB7 | 0.079404096 |
| ERF | FAM105A | 0.077005938 |
| ERF | F8 | 0.075780924 |
| ERF | LLPH | 0.064621094 |
| ERF | ZNF579 | 0.064379568 |
| ERF | SLC36A1 | 0.06252506 |
| ERF | KCNAB2 | 0.062182785 |
| ERF | GALNT9 | 0.055854246 |
| ERF | PLEK2 | 0.055079896 |
| ERF | PABPC5 | 0.048244336 |
| ERF | CIC | 0.047183462 |
| ERF | TOR1AIP1 | 0.040983872 |
| ERF | ZNF141 | 0.040469353 |
| ERF | WNT16 | 0.0313267 |
| ERF | SLC35A3 | 0.024074197 |
| ERF | CDH6 | 0.020386698 |
| ERF | SCARF1 | 0.016285213 |
| ERF | PALM2 | 0.015548951 |
| ERF | FOXO1 | 0.007880878 |
| ERF | CLPTM1L | 0.002886423 |
| ERF | PAK1IP1 | 0.002731546 |
| ERF | ATP6V1D | 0.001324944 |
| ERF | BRWD1 | 0.00065245 |
| ERF | ZNF253 | 7.97E-06 |
| ERF | ZNF429 | 5.20E-08 |
| ERG | NUB1 | 3.897178725 |
| ERG | ZC3H13 | 3.435316125 |
| ERG | A2M | 3.287763287 |
| ERG | CAPG | 3.076144504 |
| ERG | MPRIP | 2.691943008 |
| ERG | BEND6 | 2.448543829 |
| ERG | WWC2 | 2.435617499 |
| ERG | MRPS18C | 2.220906315 |
| ERG | SAT1 | 2.10398265 |
| ERG | C20orf24 | 1.988853532 |
| ERG | CBFB | 1.972660904 |
| ERG | RARRES1 | 1.510930121 |
| ERG | ZNF84 | 1.403599466 |
| ERG | PDE4DIP | 1.278622743 |
| ERG | RAD23A | 1.233649785 |
| ERG | DLEU2 | 1.203538392 |
| ERG | ARHGAP12 | 1.166214554 |
| ERG | TMEM91 | 1.081503535 |
| ERG | STIM1 | 1.067585008 |
| ERG | ERG | 1 |
| ERG | AP1B1 | 0.998074152 |
| ERG | NT5C | 0.995255145 |
| ERG | MRPS34 | 0.978347936 |
| ERG | MTCH2 | 0.969517897 |
| ERG | HES4 | 0.865603169 |
| ERG | NDUFS4 | 0.851635 |
| ERG | CLCN3 | 0.8482684 |
| ERG | C3orf14 | 0.845009119 |
| ERG | APOE | 0.834682058 |
| ERG | DNM2 | 0.823633693 |
| ERG | PAIP2 | 0.80291882 |
| ERG | CRIPT | 0.798104446 |
| ERG | CD163L1 | 0.785202675 |
| ERG | PSMB4 | 0.758487315 |
| ERG | TBC1D23 | 0.750680971 |
| ERG | BOC | 0.743432606 |
| ERG | LYRM1 | 0.69350709 |
| ERG | POLR2F | 0.684045779 |
| ERG | EIF2AK4 | 0.682952091 |
| ERG | CTNNBL1 | 0.664248908 |
| ERG | TRNAU1AP | 0.636188624 |
| ERG | CNRIP1 | 0.623317158 |
| ERG | ABCA5 | 0.613968534 |
| ERG | CYC1 | 0.60592296 |
| ERG | KCNK2 | 0.600289226 |
| ERG | JMJD8 | 0.590404284 |
| ERG | YBX1 | 0.574116253 |
| ERG | FARP1 | 0.556212802 |
| ERG | PFDN6 | 0.553028807 |
| ERG | ARPC3 | 0.540247459 |
| ERG | POLR1D | 0.534451091 |
| ERG | CDV3 | 0.531175258 |
| ERG | RANGRF | 0.520035671 |
| ERG | AR | 0.517440464 |
| ERG | SRP72 | 0.488451325 |
| ERG | B3GNT5 | 0.483317414 |
| ERG | CAMK2N1 | 0.482552833 |
| ERG | CYB5A | 0.480611409 |
| ERG | UBE2V2 | 0.459148112 |
| ERG | TSPAN15 | 0.443673054 |
| ERG | ITGA1 | 0.419913658 |
| ERG | EEA1 | 0.412043926 |
| ERG | SH3PXD2A | 0.370291091 |
| ERG | CNP | 0.363130642 |
| ERG | ATP6V1H | 0.341479392 |
| ERG | CLIP1 | 0.339395899 |
| ERG | RWDD2A | 0.318522981 |
| ERG | ARHGEF7 | 0.315066096 |
| ERG | ZFAND2B | 0.313615599 |
| ERG | YIPF2 | 0.30484236 |
| ERG | SMCHD1 | 0.286270157 |
| ERG | PLEKHA1 | 0.280218019 |
| ERG | SAE1 | 0.277739289 |
| ERG | CHMP1A | 0.274307765 |
| ERG | UBE2Q1 | 0.272147496 |
| ERG | ARL8B | 0.271107333 |
| ERG | POP7 | 0.263142751 |
| ERG | RTF1 | 0.262016115 |
| ERG | ZC3H12A | 0.257049356 |
| ERG | CDK2AP2 | 0.256806834 |
| ERG | FDFT1 | 0.248010591 |
| ERG | CYP26B1 | 0.247335344 |
| ERG | MARS2 | 0.245667491 |
| ERG | ROBO4 | 0.243233158 |
| ERG | ORMDL1 | 0.236882073 |
| ERG | EIF2A | 0.234836742 |
| ERG | TGFB3 | 0.229567111 |
| ERG | ZNF608 | 0.227592602 |
| ERG | RPL22L1 | 0.227205693 |
| ERG | PPP4C | 0.227160092 |
| ERG | ABLIM1 | 0.222228628 |
| ERG | ALG13 | 0.215186092 |
| ERG | CYCS | 0.212015576 |
| ERG | TRIM44 | 0.206580777 |
| ERG | GRSF1 | 0.203591746 |
| ERG | METAP2 | 0.199841572 |
| ERG | IFT27 | 0.191420858 |
| ERG | SHOX2 | 0.16104901 |
| ERG | PRR14 | 0.159394239 |
| ERG | NETO2 | 0.159144524 |
| ERG | SLC22A17 | 0.138816734 |
| ERG | STAMBPL1 | 0.137518463 |
| ERG | CFI | 0.13649287 |
| ERG | BAG1 | 0.131922876 |
| ERG | TMEM59L | 0.130857002 |
| ERG | KALRN | 0.125257605 |
| ERG | KDR | 0.115643725 |
| ERG | TBC1D22A | 0.109597055 |
| ERG | SNIP1 | 0.107697597 |
| ERG | IDH3A | 0.100773096 |
| ERG | IGSF8 | 0.098531548 |
| ERG | MYO19 | 0.09035062 |
| ERG | SHQ1 | 0.088220381 |
| ERG | PCDH17 | 0.085987847 |
| ERG | PDE12 | 0.083765939 |
| ERG | DGKI | 0.081548433 |
| ERG | RFK | 0.075163856 |
| ERG | WDR37 | 0.071338255 |
| ERG | TMEM102 | 0.07086582 |
| ERG | TEC | 0.031409241 |
| ERG | F11R | 0.025045381 |
| ERG | CHMP7 | 0.000436698 |
| ESRRA | OGFR | 3.924658585 |
| ESRRA | RUNX1 | 3.846128136 |
| ESRRA | TMEM158 | 3.367611663 |
| ESRRA | BICD2 | 3.089387703 |
| ESRRA | CAMK2N1 | 2.810559634 |
| ESRRA | SNAI1 | 2.647396097 |
| ESRRA | ATAD5 | 2.59987448 |
| ESRRA | MBTPS1 | 2.414703319 |
| ESRRA | CYP1B1 | 2.390517696 |
| ESRRA | ZSCAN29 | 1.9534353 |
| ESRRA | TMEM115 | 1.858736637 |
| ESRRA | FAM50A | 1.618013861 |
| ESRRA | SFSWAP | 1.542594363 |
| ESRRA | PIP5K1A | 1.446958729 |
| ESRRA | CLN8 | 1.420922165 |
| ESRRA | CDKL5 | 1.41859499 |
| ESRRA | C16orf70 | 1.381311742 |
| ESRRA | HOXB2 | 1.379994855 |
| ESRRA | CTPS2 | 1.350201167 |
| ESRRA | GIMAP7 | 1.319329742 |
| ESRRA | LEPROT | 1.296517712 |
| ESRRA | MEA1 | 1.282729554 |
| ESRRA | PRKRIP1 | 1.266765743 |
| ESRRA | CHD9 | 1.255377934 |
| ESRRA | KCTD12 | 1.252299311 |
| ESRRA | ARMCX1 | 1.196290558 |
| ESRRA | DIAPH1 | 1.196049293 |
| ESRRA | FGFBP3 | 1.163335981 |
| ESRRA | RIT1 | 1.160437852 |
| ESRRA | ATF1 | 1.159557442 |
| ESRRA | INTS8 | 1.155604368 |
| ESRRA | SMARCAD1 | 1.132456297 |
| ESRRA | PSMG2 | 1.120544278 |
| ESRRA | CD99L2 | 1.091309533 |
| ESRRA | NUP50 | 1.080112296 |
| ESRRA | CLOCK | 1.069924568 |
| ESRRA | CRTAP | 1.055755166 |
| ESRRA | DOK6 | 1.008059146 |
| ESRRA | ESRRA | 1 |
| ESRRA | GTPBP1 | 0.972684944 |
| ESRRA | MAP1B | 0.947702677 |
| ESRRA | NRAS | 0.920719239 |
| ESRRA | RAB22A | 0.91754714 |
| ESRRA | TMX1 | 0.909288499 |
| ESRRA | PARP11 | 0.90119592 |
| ESRRA | UBXN11 | 0.874700051 |
| ESRRA | RAB18 | 0.856622023 |
| ESRRA | COQ3 | 0.85354435 |
| ESRRA | TWISTNB | 0.850461359 |
| ESRRA | ACVR1B | 0.844524943 |
| ESRRA | PDGFRB | 0.829636133 |
| ESRRA | MYBBP1A | 0.828398816 |
| ESRRA | TSPAN17 | 0.81714694 |
| ESRRA | ZNHIT2 | 0.811538786 |
| ESRRA | FMNL3 | 0.783183512 |
| ESRRA | PARP8 | 0.781049572 |
| ESRRA | PDRG1 | 0.772848814 |
| ESRRA | RBBP6 | 0.76295827 |
| ESRRA | ANGEL1 | 0.75809637 |
| ESRRA | ARL5B | 0.705052597 |
| ESRRA | DMC1 | 0.703024132 |
| ESRRA | TPD52L2 | 0.700320665 |
| ESRRA | CFL2 | 0.684640498 |
| ESRRA | CSK | 0.668637767 |
| ESRRA | HSPB7 | 0.651417432 |
| ESRRA | NUP88 | 0.648033133 |
| ESRRA | KBTBD11 | 0.64417856 |
| ESRRA | WDR31 | 0.640206027 |
| ESRRA | IFT140 | 0.637295937 |
| ESRRA | NEAT1 | 0.633463732 |
| ESRRA | NAP1L3 | 0.620603325 |
| ESRRA | HECTD1 | 0.612995554 |
| ESRRA | LYST | 0.597459773 |
| ESRRA | STAT5A | 0.595289386 |
| ESRRA | MAFB | 0.593655968 |
| ESRRA | TPM1 | 0.589148307 |
| ESRRA | MAP3K11 | 0.585381229 |
| ESRRA | HNRNPC | 0.579906044 |
| ESRRA | SLC30A5 | 0.568770122 |
| ESRRA | MGA | 0.559717014 |
| ESRRA | OPLAH | 0.551237102 |
| ESRRA | RABGAP1L | 0.546226521 |
| ESRRA | RPP38 | 0.541295098 |
| ESRRA | DDX42 | 0.53086455 |
| ESRRA | NOP58 | 0.525694142 |
| ESRRA | E4F1 | 0.525316502 |
| ESRRA | RPL13A | 0.516782632 |
| ESRRA | PCTP | 0.516677014 |
| ESRRA | SEC14L1 | 0.511509115 |
| ESRRA | SH2D2A | 0.501094575 |
| ESRRA | TUBB2A | 0.49193878 |
| ESRRA | TOMM7 | 0.484460174 |
| ESRRA | SENP2 | 0.479944439 |
| ESRRA | SLC25A46 | 0.47651777 |
| ESRRA | SLC3A2 | 0.466760758 |
| ESRRA | RAB31 | 0.465089842 |
| ESRRA | FBXW4 | 0.464080522 |
| ESRRA | LEF1 | 0.45655426 |
| ESRRA | RPS3A | 0.449558641 |
| ESRRA | PSKH1 | 0.447987345 |
| ESRRA | GPBP1 | 0.437198934 |
| ESRRA | ATP10A | 0.429442759 |
| ESRRA | KLHL18 | 0.383024844 |
| ESRRA | CRTAC1 | 0.380740943 |
| ESRRA | ANKRD13D | 0.380338279 |
| ESRRA | SGPP2 | 0.378043154 |
| ESRRA | THRA | 0.373707 |
| ESRRA | MKLN1 | 0.355914736 |
| ESRRA | ITGB8 | 0.352726726 |
| ESRRA | KIF9 | 0.333530021 |
| ESRRA | DOK5 | 0.328206223 |
| ESRRA | RBM15B | 0.327397725 |
| ESRRA | SQLE | 0.309104455 |
| ESRRA | TADA2A | 0.302119076 |
| ESRRA | DNMBP | 0.293585345 |
| ESRRA | INO80C | 0.291240862 |
| ESRRA | ISYNA1 | 0.285952607 |
| ESRRA | PMM2 | 0.284248754 |
| ESRRA | RICTOR | 0.283991554 |
| ESRRA | MUM1 | 0.276460348 |
| ESRRA | PRR3 | 0.272730837 |
| ESRRA | TMEM175 | 0.27185967 |
| ESRRA | MAST2 | 0.271643664 |
| ESRRA | TFAP4 | 0.269028831 |
| ESRRA | BCL9L | 0.267921175 |
| ESRRA | ATXN1 | 0.264258819 |
| ESRRA | RAD9A | 0.262656466 |
| ESRRA | NPTXR | 0.25983913 |
| ESRRA | FADS2 | 0.256599792 |
| ESRRA | SSBP2 | 0.25291877 |
| ESRRA | PTGIR | 0.252548492 |
| ESRRA | CLINT1 | 0.250584135 |
| ESRRA | FAM117A | 0.250196267 |
| ESRRA | GJB6 | 0.247153503 |
| ESRRA | PPT2 | 0.243375606 |
| ESRRA | AHCY | 0.240394959 |
| ESRRA | ST8SIA1 | 0.240135848 |
| ESRRA | SYCE1L | 0.237222175 |
| ESRRA | CADM1 | 0.236459054 |
| ESRRA | STXBP3 | 0.234310293 |
| ESRRA | BIRC3 | 0.234279061 |
| ESRRA | SH3PXD2A | 0.231548978 |
| ESRRA | ARHGAP5 | 0.230009531 |
| ESRRA | FMR1 | 0.224441705 |
| ESRRA | SREK1 | 0.216313432 |
| ESRRA | SLC31A2 | 0.215215232 |
| ESRRA | TCF23 | 0.215043427 |
| ESRRA | ENOPH1 | 0.212988099 |
| ESRRA | RAP2A | 0.212925891 |
| ESRRA | HMBOX1 | 0.211205368 |
| ESRRA | ZC3H3 | 0.209490375 |
| ESRRA | WTIP | 0.204060607 |
| ESRRA | KCTD1 | 0.201331827 |
| ESRRA | RNF38 | 0.199794324 |
| ESRRA | EPC1 | 0.198251539 |
| ESRRA | BCL2L11 | 0.197051646 |
| ESRRA | ZHX2 | 0.196177522 |
| ESRRA | HLX | 0.194840342 |
| ESRRA | IQGAP2 | 0.190169566 |
| ESRRA | SMU1 | 0.190149732 |
| ESRRA | PEX2 | 0.190129712 |
| ESRRA | CTCF | 0.186225289 |
| ESRRA | SCAMP4 | 0.182293901 |
| ESRRA | CHD6 | 0.180417243 |
| ESRRA | BDP1 | 0.180061544 |
| ESRRA | HEY2 | 0.178318822 |
| ESRRA | PDHA1 | 0.17789056 |
| ESRRA | NUDT3 | 0.17690625 |
| ESRRA | PTP4A3 | 0.176176548 |
| ESRRA | GNA12 | 0.17470862 |
| ESRRA | UTP18 | 0.169080573 |
| ESRRA | RSPO1 | 0.168016008 |
| ESRRA | GALNT3 | 0.155451956 |
| ESRRA | PDCD6IP | 0.150087068 |
| ESRRA | NEDD4L | 0.149834936 |
| ESRRA | RCC1 | 0.140011864 |
| ESRRA | RGMB | 0.135946346 |
| ESRRA | SMARCE1 | 0.135722862 |
| ESRRA | KDR | 0.133170569 |
| ESRRA | ZNF24 | 0.120552416 |
| ESRRA | CPSF1 | 0.116475279 |
| ESRRA | ZNF35 | 0.102164771 |
| ESRRA | AMMECR1 | 0.095246899 |
| ESRRA | ZNF213 | 0.093112762 |
| ESRRA | NOC4L | 0.091642411 |
| ESRRA | ITPR3 | 0.09050816 |
| ESRRA | MESP1 | 0.077212272 |
| ESRRA | ANO1 | 0.076645232 |
| ESRRA | MYO18A | 0.071706093 |
| ESRRA | ZNF384 | 0.062665778 |
| ESRRA | CEP76 | 0.049909856 |
| ESRRA | VAV2 | 0.047046689 |
| ESRRA | DHX35 | 0.04435294 |
| ESRRA | ZCCHC6 | 0.038773574 |
| ESRRA | FAM69B | 0.037695957 |
| ESRRA | POU2F3 | 0.035929612 |
| ESRRA | CDK5 | 0.032287833 |
| ESRRA | TPD52 | 0.032107319 |
| ESRRA | SLC26A10 | 0.024442528 |
| ESRRA | EFS | 0.015920855 |
| ESRRA | CGNL1 | 0.013101409 |
| ESRRA | MYH11 | 0.011065294 |
| ESRRA | PPIF | 0.007609714 |
| ESRRA | STX12 | 0.005432758 |
| ESRRA | CPNE1 | 0.005425055 |
| ESRRA | SOCS6 | 5.68E-05 |
| ESRRA | TBC1D20 | 4.69E-17 |
| ESRRA | HIST1H2BB | 1.65E-18 |
| ESRRA | GPR162 | 2.89E-19 |
| ETS1 | UXT | 4.425192147 |
| ETS1 | HAS2 | 3.749008799 |
| ETS1 | EIF4A2 | 3.734256826 |
| ETS1 | GSTP1 | 2.785186959 |
| ETS1 | CREBZF | 2.622923829 |
| ETS1 | WASF2 | 2.523576152 |
| ETS1 | TNKS2 | 2.450755939 |
| ETS1 | MDH1 | 2.166377762 |
| ETS1 | PDE4B | 2.110944172 |
| ETS1 | ACSL3 | 2.084595846 |
| ETS1 | PSMD2 | 2.081790304 |
| ETS1 | MAGED2 | 1.986027034 |
| ETS1 | COPE | 1.954914389 |
| ETS1 | CUX1 | 1.926247129 |
| ETS1 | TADA3 | 1.872032995 |
| ETS1 | ZFP91 | 1.831084405 |
| ETS1 | TRIM24 | 1.819071472 |
| ETS1 | IL1R1 | 1.749090541 |
| ETS1 | OLFML3 | 1.676781795 |
| ETS1 | CDCA5 | 1.647300213 |
| ETS1 | ABHD2 | 1.618584617 |
| ETS1 | EAPP | 1.538995338 |
| ETS1 | UQCRC2 | 1.52917239 |
| ETS1 | TP63 | 1.523439375 |
| ETS1 | PALMD | 1.514281826 |
| ETS1 | STOM | 1.45945926 |
| ETS1 | NFE2L3 | 1.456441301 |
| ETS1 | RND3 | 1.329044762 |
| ETS1 | PRDX5 | 1.241112562 |
| ETS1 | HNRNPD | 1.225510074 |
| ETS1 | KIAA1468 | 1.20016616 |
| ETS1 | FEZ1 | 1.196975224 |
| ETS1 | STEAP4 | 1.176611751 |
| ETS1 | SDAD1 | 1.142916309 |
| ETS1 | CD82 | 1.03156372 |
| ETS1 | PLCB3 | 1.02592812 |
| ETS1 | ETS1 | 1 |
| ETS1 | SRM | 0.96734157 |
| ETS1 | HLA-C | 0.963263785 |
| ETS1 | RBM17 | 0.936369477 |
| ETS1 | KLF10 | 0.921346485 |
| ETS1 | ATP6V0E1 | 0.886081175 |
| ETS1 | MRPS14 | 0.864232788 |
| ETS1 | MAP2K2 | 0.802205481 |
| ETS1 | TLCD1 | 0.781986424 |
| ETS1 | SCRG1 | 0.757829079 |
| ETS1 | TOX2 | 0.753083996 |
| ETS1 | MPRIP | 0.731936777 |
| ETS1 | AP1G1 | 0.712082598 |
| ETS1 | PITPNC1 | 0.694812385 |
| ETS1 | LYPD5 | 0.673170286 |
| ETS1 | TMEM107 | 0.670438818 |
| ETS1 | DSG3 | 0.667718999 |
| ETS1 | LSAMP | 0.653874225 |
| ETS1 | GNAS | 0.650257391 |
| ETS1 | TMEM92 | 0.636522731 |
| ETS1 | PRDX4 | 0.6204982 |
| ETS1 | RHOBTB1 | 0.59883631 |
| ETS1 | PPM1G | 0.594831171 |
| ETS1 | CDH11 | 0.580691706 |
| ETS1 | TIMP3 | 0.566613816 |
| ETS1 | COL6A5 | 0.556228955 |
| ETS1 | MESP1 | 0.547607518 |
| ETS1 | SERPINB6 | 0.540367928 |
| ETS1 | TAX1BP1 | 0.532874469 |
| ETS1 | DAB2 | 0.508390145 |
| ETS1 | FAM169B | 0.492717879 |
| ETS1 | FYN | 0.486646617 |
| ETS1 | GADD45A | 0.485150003 |
| ETS1 | VRK3 | 0.483439714 |
| ETS1 | AP2S1 | 0.481190968 |
| ETS1 | COX5B | 0.469295347 |
| ETS1 | SLC41A1 | 0.456225094 |
| ETS1 | TSG101 | 0.455286632 |
| ETS1 | PIN4 | 0.453601942 |
| ETS1 | SERBP1 | 0.452240629 |
| ETS1 | EMILIN1 | 0.450124242 |
| ETS1 | KIFC2 | 0.447848219 |
| ETS1 | PACSIN2 | 0.445947038 |
| ETS1 | CACNA1D | 0.440961594 |
| ETS1 | GADD45B | 0.438726667 |
| ETS1 | PDGFRB | 0.434156554 |
| ETS2 | ZFAND5 | 11.98655492 |
| ETS2 | MT2A | 5.757943507 |
| ETS2 | UBC | 5.173753239 |
| ETS2 | ITPRIP | 4.932329786 |
| ETS2 | AKAP12 | 4.620554446 |
| ETS2 | PERP | 4.082522352 |
| ETS2 | RPL26 | 3.651169 |
| ETS2 | NEAT1 | 3.557701484 |
| ETS2 | CDKN1A | 3.269440984 |
| ETS2 | HSPD1 | 3.100712389 |
| ETS2 | RPL19 | 2.912271729 |
| ETS2 | FSTL1 | 2.636813483 |
| ETS2 | ICAM1 | 2.487174166 |
| ETS2 | RAB38 | 2.40101176 |
| ETS2 | TIMP2 | 2.320178193 |
| ETS2 | RND3 | 2.22635181 |
| ETS2 | ACTB | 2.036123071 |
| ETS2 | HSPH1 | 2.025272504 |
| ETS2 | RPS21 | 1.952122226 |
| ETS2 | SFN | 1.742486737 |
| ETS2 | RPL34 | 1.613131551 |
| ETS2 | PPIC | 1.608356494 |
| ETS2 | EPHB6 | 1.571006576 |
| ETS2 | PPP2R5C | 1.538097659 |
| ETS2 | RPS27A | 1.515537215 |
| ETS2 | ZFP36 | 1.512486417 |
| ETS2 | DDX5 | 1.437224108 |
| ETS2 | UPF2 | 1.39473208 |
| ETS2 | TES | 1.383543167 |
| ETS2 | EIF1 | 1.256643194 |
| ETS2 | AR | 1.25244742 |
| ETS2 | STEAP4 | 1.234854604 |
| ETS2 | CDK9 | 1.233109168 |
| ETS2 | NASP | 1.233071315 |
| ETS2 | KLF6 | 1.18727654 |
| ETS2 | PLAC9 | 1.117780831 |
| ETS2 | KANK3 | 1.093300986 |
| ETS2 | RPS26 | 1.084000216 |
| ETS2 | BTF3 | 1.026580882 |
| ETS2 | CCL20 | 1.002855168 |
| ETS2 | ETS2 | 1 |
| ETS2 | PFKFB3 | 0.993970308 |
| ETS2 | ATP1B1 | 0.990050972 |
| ETS2 | RGS16 | 0.987977368 |
| ETS2 | IRF1 | 0.972929986 |
| ETS2 | CDC42 | 0.958264027 |
| ETS2 | INCENP | 0.954671628 |
| ETS2 | CLN5 | 0.939079162 |
| ETS2 | RPL38 | 0.938725147 |
| ETS2 | CTSK | 0.903085774 |
| ETS2 | MGST3 | 0.898836138 |
| ETS2 | HNMT | 0.896085391 |
| ETS2 | SGK1 | 0.857905382 |
| ETS2 | CD81 | 0.856181774 |
| ETS2 | LGALS3 | 0.847840518 |
| ETS2 | FAM49B | 0.847347469 |
| ETS2 | BAZ2B | 0.846967881 |
| ETS2 | NOC3L | 0.826987393 |
| ETS2 | CXorf36 | 0.814668981 |
| ETS2 | RPL32 | 0.807172797 |
| ETS2 | RPL27A | 0.790141923 |
| ETS2 | TTC39C | 0.778314812 |
| ETS2 | LTN1 | 0.775036162 |
| ETS2 | ZNF496 | 0.761979222 |
| ETS2 | GAS5 | 0.758440529 |
| ETS2 | STC1 | 0.745014962 |
| ETS2 | LRP1 | 0.73056234 |
| ETS2 | IL1RN | 0.718326159 |
| ETS2 | MICALL1 | 0.715543782 |
| ETS2 | OSTF1 | 0.685016611 |
| ETS2 | HSPA8 | 0.670421551 |
| ETS2 | JTB | 0.666385832 |
| ETS2 | PFN1 | 0.655808903 |
| ETS2 | GJB2 | 0.645715734 |
| ETS2 | TTC17 | 0.643711373 |
| ETS2 | HBEGF | 0.636278135 |
| ETS2 | TMBIM6 | 0.634019631 |
| ETS2 | CYR61 | 0.629608652 |
| ETS2 | FOXN3 | 0.624036547 |
| ETS2 | EIF4A2 | 0.621529533 |
| ETS2 | BCL11A | 0.619404341 |
| ETS2 | CD248 | 0.616698053 |
| ETS2 | EEF1E1 | 0.59168663 |
| ETS2 | NFKBIZ | 0.590001817 |
| ETS2 | GUCY1A2 | 0.589057553 |
| ETS2 | DYNC1I2 | 0.581642885 |
| ETS2 | C10orf55 | 0.579551632 |
| ETS2 | NBEAL2 | 0.56604579 |
| ETS2 | LIMS1 | 0.559361739 |
| ETS2 | PCOLCE | 0.555698864 |
| ETS2 | ANKRD11 | 0.554884684 |
| ETS2 | PSMB1 | 0.553077455 |
| ETS2 | EFNA1 | 0.552490667 |
| ETS2 | AHSA1 | 0.54042348 |
| ETS2 | ABI1 | 0.532896061 |
| ETS2 | S100A9 | 0.531512395 |
| ETS2 | ORMDL3 | 0.530052518 |
| ETS2 | LMNB1 | 0.520108212 |
| ETS2 | THBS2 | 0.513592949 |
| ETS2 | YTHDC1 | 0.511504018 |
| ETS2 | FAM32A | 0.50986892 |
| ETS2 | RER1 | 0.505660135 |
| ETS2 | VIM | 0.497545725 |
| ETS2 | TMEM40 | 0.495922213 |
| ETS2 | CSF2RB | 0.493921013 |
| ETS2 | TAF5 | 0.491516175 |
| ETS2 | GPX7 | 0.486591114 |
| ETS2 | RPS5 | 0.485263744 |
| ETS2 | EFNB3 | 0.484260171 |
| ETS2 | F11R | 0.475809499 |
| ETS2 | RAI14 | 0.474485378 |
| ETS2 | MAX | 0.474225758 |
| ETS2 | DSP | 0.46305574 |
| ETS2 | GNAS | 0.458829703 |
| ETS2 | PPIA | 0.454294745 |
| ETS2 | IL7R | 0.444235248 |
| ETS2 | DGCR8 | 0.443333346 |
| ETS2 | PTPN21 | 0.439286466 |
| ETS2 | NOTCH1 | 0.439056988 |
| ETS2 | MOB2 | 0.438294437 |
| ETS2 | RPL35 | 0.436682427 |
| ETS2 | TIGD6 | 0.372012149 |
| ETS2 | HNRNPH2 | 0.319913025 |
| ETS2 | B4GALT1 | 0.290964998 |
| ETS2 | KIAA0040 | 0.261745471 |
| ETS2 | WDR37 | 0.238746662 |
| ETS2 | NR1H2 | 0.221129004 |
| ETS2 | DALRD3 | 0.212938305 |
| ETS2 | FZR1 | 0.209095176 |
| ETS2 | ZBTB7B | 0.204822444 |
| ETS2 | ATG2B | 0.197893816 |
| ETS2 | TPRG1L | 0.171515237 |
| ETS2 | RPF1 | 0.161008412 |
| ETS2 | ZNF513 | 0.15378471 |
| ETS2 | CUTC | 0.139557059 |
| ETS2 | PER2 | 0.136524796 |
| ETS2 | MARK2 | 0.118189991 |
| ETS2 | ECE1 | 0.109565512 |
| ETS2 | NLRX1 | 0.059633467 |
| ETS2 | C19orf25 | 0.022407513 |
| ETS2 | RNPEPL1 | 0.002639228 |
| ETS2 | KIAA1468 | 1.24E-06 |
| ETS2 | NCF2 | 1.08E-07 |
| ETS2 | CCL23 | 7.15E-19 |
| ETS2 | NAV3 | 5.28E-19 |
| ETS2 | C7orf43 | 3.40E-19 |
| ETV1 | A2M | 5.993910456 |
| ETV1 | NINJ1 | 4.59436902 |
| ETV1 | WFDC1 | 4.471337044 |
| ETV1 | MR1 | 4.377067534 |
| ETV1 | RGS6 | 4.347790053 |
| ETV1 | SLC35A2 | 4.195891106 |
| ETV1 | ZNF562 | 3.316944072 |
| ETV1 | TMF1 | 3.245670811 |
| ETV1 | GTF3C5 | 3.223554138 |
| ETV1 | COL18A1 | 3.131909767 |
| ETV1 | SFT2D2 | 3.107688582 |
| ETV1 | DIDO1 | 3.074793326 |
| ETV1 | CDK19 | 3.019562811 |
| ETV1 | NKAPL | 2.573223929 |
| ETV1 | UBE2F | 2.552958228 |
| ETV1 | MAPKAP1 | 2.48977407 |
| ETV1 | NOV | 2.438482189 |
| ETV1 | ARL5B | 2.405073775 |
| ETV1 | NUDT19 | 2.393559265 |
| ETV1 | ERH | 2.19853355 |
| ETV1 | ZNF624 | 2.194193347 |
| ETV1 | HSCB | 2.152693056 |
| ETV1 | GON4L | 2.147768632 |
| ETV1 | STIM2 | 2.1372523 |
| ETV1 | ZCCHC4 | 2.048263526 |
| ETV1 | NPM3 | 2.037686702 |
| ETV1 | TRIP11 | 2.007248576 |
| ETV1 | PLCD1 | 2.000571311 |
| ETV1 | ITGA6 | 1.917657253 |
| ETV1 | GRPEL1 | 1.899591623 |
| ETV1 | CNIH4 | 1.878591039 |
| ETV1 | RNF216 | 1.825994484 |
| ETV1 | IFITM2 | 1.822843573 |
| ETV1 | PURB | 1.818911055 |
| ETV1 | FGFR2 | 1.789671871 |
| ETV1 | NENF | 1.770022995 |
| ETV1 | SDCBP | 1.765559634 |
| ETV1 | DIS3 | 1.689671724 |
| ETV1 | SDF2 | 1.675012685 |
| ETV1 | ARAP1 | 1.623412684 |
| ETV1 | PPCDC | 1.574605935 |
| ETV1 | GRIPAP1 | 1.523838967 |
| ETV1 | WDR46 | 1.516864114 |
| ETV1 | OLA1 | 1.478409245 |
| ETV1 | PELI2 | 1.459979598 |
| ETV1 | SUPT5H | 1.367322434 |
| ETV1 | TGFBR2 | 1.365888393 |
| ETV1 | RELL1 | 1.314334679 |
| ETV1 | TMBIM1 | 1.293390262 |
| ETV1 | ADAMTS8 | 1.281185755 |
| ETV1 | MRPS26 | 1.280678529 |
| ETV1 | HEBP2 | 1.273756193 |
| ETV1 | DCP2 | 1.25492182 |
| ETV1 | SEPHS1 | 1.252276731 |
| ETV1 | DHRS1 | 1.249923859 |
| ETV1 | C10orf76 | 1.249516821 |
| ETV1 | IRF7 | 1.208171034 |
| ETV1 | FAM96B | 1.208072127 |
| ETV1 | DNAJC25 | 1.196371534 |
| ETV1 | GOLGA5 | 1.190550273 |
| ETV1 | TIMM8B | 1.185368234 |
| ETV1 | STK17B | 1.114023424 |
| ETV1 | HEXIM2 | 1.084107679 |
| ETV1 | APOD | 1.078193501 |
| ETV1 | USP39 | 1.056800866 |
| ETV1 | CSRP1 | 1.030474756 |
| ETV1 | PDZD8 | 1.027156737 |
| ETV1 | ETV1 | 1 |
| ETV1 | TPK1 | 0.967741767 |
| ETV1 | PARVA | 0.927485384 |
| ETV1 | P2RY14 | 0.903175951 |
| ETV1 | PRSS3 | 0.876919173 |
| ETV1 | DOK5 | 0.859051522 |
| ETV1 | POMP | 0.853427162 |
| ETV1 | NSD1 | 0.851363332 |
| ETV1 | ATG2B | 0.836335197 |
| ETV1 | KDM3B | 0.821628777 |
| ETV1 | SEC31A | 0.800104414 |
| ETV1 | RAP2B | 0.738549359 |
| ETV1 | ADAMTS5 | 0.725503222 |
| ETV1 | AKAP12 | 0.719271651 |
| ETV1 | ICAM1 | 0.708409673 |
| ETV1 | YBX1 | 0.691606372 |
| ETV1 | CDKN1C | 0.69150826 |
| ETV1 | ZC3HC1 | 0.675847291 |
| ETV1 | OAF | 0.670282072 |
| ETV1 | ASH2L | 0.652069822 |
| ETV1 | PDLIM3 | 0.644752602 |
| ETV1 | SLC25A17 | 0.639127773 |
| ETV1 | ADAMTSL2 | 0.630885508 |
| ETV1 | ENY2 | 0.623779233 |
| ETV1 | RAPGEF6 | 0.606412118 |
| ETV1 | POLR1E | 0.604109086 |
| ETV1 | NDUFAF3 | 0.598689311 |
| ETV1 | GJB2 | 0.595938037 |
| ETV1 | SDHC | 0.584076275 |
| ETV1 | NOL8 | 0.563957919 |
| ETV1 | CHMP5 | 0.560684217 |
| ETV1 | ALKBH2 | 0.559551475 |
| ETV1 | JAG1 | 0.534691621 |
| ETV1 | WDR70 | 0.527959505 |
| ETV1 | RAB10 | 0.517306654 |
| ETV1 | REL | 0.514214293 |
| ETV1 | HAGHL | 0.508619722 |
| ETV1 | TOR1B | 0.506218665 |
| ETV1 | PEX12 | 0.506146688 |
| ETV1 | TJP1 | 0.505710597 |
| ETV1 | KLHL5 | 0.498978295 |
| ETV1 | FAM120AOS | 0.495868271 |
| ETV1 | EGFR | 0.495796123 |
| ETV1 | C9orf85 | 0.480104677 |
| ETV1 | CTSC | 0.47235463 |
| ETV1 | SELL | 0.46298046 |
| ETV1 | RPL6 | 0.457648719 |
| ETV1 | GLT8D2 | 0.453782646 |
| ETV1 | KRT7 | 0.447203542 |
| ETV1 | CEP63 | 0.436368385 |
| ETV1 | ZNF780A | 0.426567649 |
| ETV1 | F8 | 0.410623498 |
| ETV1 | SSFA2 | 0.409231528 |
| ETV1 | PTPN21 | 0.403265367 |
| ETV1 | GOLGA2 | 0.399526535 |
| ETV1 | NUFIP2 | 0.396324788 |
| ETV1 | SERP2 | 0.392121392 |
| ETV1 | N4BP2L2 | 0.37349135 |
| ETV1 | DAZL | 0.358818054 |
| ETV1 | NGFR | 0.356313966 |
| ETV1 | MED10 | 0.319496258 |
| ETV1 | MCM3 | 0.319484999 |
| ETV1 | PPP1R3E | 0.31113285 |
| ETV1 | DLG4 | 0.299077611 |
| ETV1 | IGFBP6 | 0.282522943 |
| ETV1 | VASH1 | 0.27853148 |
| ETV1 | ERLIN2 | 0.269400803 |
| ETV1 | SRPK2 | 0.265841073 |
| ETV1 | NXPH3 | 0.263480082 |
| ETV1 | C12orf75 | 0.255947072 |
| ETV1 | RBMS3 | 0.251428735 |
| ETV1 | RNF14 | 0.251173307 |
| ETV1 | KDM5A | 0.249650551 |
| ETV1 | PSMD6 | 0.248945876 |
| ETV1 | ITPRIPL2 | 0.247146843 |
| ETV1 | FOXN3 | 0.246513018 |
| ETV1 | FCHO2 | 0.243423185 |
| ETV1 | TRAM2 | 0.2391965 |
| ETV1 | NAA10 | 0.237136719 |
| ETV1 | TRAF4 | 0.235899975 |
| ETV1 | C19orf48 | 0.233281914 |
| ETV1 | PAK1IP1 | 0.230233641 |
| ETV1 | ARCN1 | 0.219449371 |
| ETV1 | ZADH2 | 0.218327072 |
| ETV1 | ACTN4 | 0.217364583 |
| ETV1 | ELP3 | 0.216355038 |
| ETV1 | SPTAN1 | 0.213203024 |
| ETV1 | NOM1 | 0.213005913 |
| ETV1 | PAAF1 | 0.211850904 |
| ETV1 | SLC25A24 | 0.210098513 |
| ETV1 | PTPN1 | 0.199593104 |
| ETV1 | BHLHE22 | 0.196190247 |
| ETV1 | ZMAT2 | 0.190330484 |
| ETV1 | SPRY4 | 0.181751869 |
| ETV1 | TSC1 | 0.181612658 |
| ETV1 | AP4B1 | 0.178411422 |
| ETV1 | MARK2 | 0.177952398 |
| ETV1 | SETD1A | 0.173343386 |
| ETV1 | MTF2 | 0.16906384 |
| ETV1 | FBXO32 | 0.163379186 |
| ETV1 | ITGA1 | 0.157650976 |
| ETV1 | TMEM222 | 0.150032516 |
| ETV1 | KIF5C | 0.141779597 |
| ETV1 | SPRY2 | 0.13841785 |
| ETV1 | KDM1B | 0.132872633 |
| ETV1 | TLCD2 | 0.124480267 |
| ETV1 | SYNPO | 0.122929081 |
| ETV1 | MIPOL1 | 0.111780706 |
| ETV1 | MTMR10 | 0.111565438 |
| ETV1 | PCSK7 | 0.10883787 |
| ETV1 | ZBTB40 | 0.103355172 |
| ETV1 | EIF2S3 | 0.101592925 |
| ETV1 | STAG3L4 | 0.096695649 |
| ETV1 | SEPSECS | 0.096673383 |
| ETV1 | TMEM67 | 0.09498476 |
| ETV1 | FCHSD2 | 0.093886748 |
| ETV1 | LIG3 | 0.093671412 |
| ETV1 | MGAT5 | 0.092454843 |
| ETV1 | N4BP2 | 0.092024239 |
| ETV1 | HIST1H4H | 0.091296175 |
| ETV1 | ADAM22 | 0.090436011 |
| ETV1 | ABCD3 | 0.088210465 |
| ETV1 | HAUS8 | 0.085508452 |
| ETV1 | KIF22 | 0.082686671 |
| ETV1 | CAMK4 | 0.079646821 |
| ETV1 | NAT6 | 0.039315044 |
| ETV1 | LRRC69 | 0.037068246 |
| ETV1 | GPS2 | 0.034546343 |
| ETV1 | IL11 | 0.026969578 |
| ETV1 | MOCS3 | 0.019448497 |
| ETV1 | CLPTM1L | 0.003440206 |
| ETV2 | PPP2R1A | 5.409253665 |
| ETV2 | ZNF226 | 3.137630959 |
| ETV2 | ARMC1 | 2.994875788 |
| ETV2 | AKR1C3 | 2.774633062 |
| ETV2 | SLC26A7 | 2.708509503 |
| ETV2 | TMEM80 | 2.625574822 |
| ETV2 | TMEM176A | 2.422575913 |
| ETV2 | MFAP2 | 2.420938244 |
| ETV2 | KDELR1 | 2.388592866 |
| ETV2 | C21orf59 | 2.292961188 |
| ETV2 | SYS1 | 2.282369128 |
| ETV2 | SPG7 | 2.272909019 |
| ETV2 | ALKBH5 | 2.212368495 |
| ETV2 | EIF4G1 | 2.127460743 |
| ETV2 | MSH6 | 2.069820645 |
| ETV2 | FKBP1A | 2.027917853 |
| ETV2 | PPM1L | 1.961816524 |
| ETV2 | ANGPTL2 | 1.956169556 |
| ETV2 | POLB | 1.837708837 |
| ETV2 | GLUD1 | 1.784722077 |
| ETV2 | GRB10 | 1.772585577 |
| ETV2 | MARK2 | 1.770764752 |
| ETV2 | PEX26 | 1.753167587 |
| ETV2 | PBRM1 | 1.733997445 |
| ETV2 | CCDC90B | 1.670543062 |
| ETV2 | FREM1 | 1.658073164 |
| ETV2 | SDR16C5 | 1.595152038 |
| ETV2 | IPO9 | 1.56890071 |
| ETV2 | DDX60L | 1.556131546 |
| ETV2 | NUP107 | 1.555397994 |
| ETV2 | STAG2 | 1.490728823 |
| ETV2 | FTSJ3 | 1.375262508 |
| ETV2 | RBBP4 | 1.346370202 |
| ETV2 | YKT6 | 1.316536189 |
| ETV2 | FBXO38 | 1.316465925 |
| ETV2 | EPHB3 | 1.26930113 |
| ETV2 | TARSL2 | 1.25639457 |
| ETV2 | LSM3 | 1.230444512 |
| ETV2 | GNG12 | 1.194617577 |
| ETV2 | NUBPL | 1.163584122 |
| ETV2 | SEC22C | 1.130809376 |
| ETV2 | PPRC1 | 1.109584833 |
| ETV2 | CDC16 | 1.104766505 |
| ETV2 | FAM96A | 1.087419461 |
| ETV2 | RWDD1 | 1.060515736 |
| ETV2 | BUB1B | 1.060441787 |
| ETV2 | DPF2 | 1.034216533 |
| ETV2 | GSK3B | 1.014634773 |
| ETV2 | ETV2 | 1 |
| ETV2 | PYCR2 | 0.904355078 |
| ETV2 | ALDOC | 0.878493359 |
| ETV2 | C3orf14 | 0.869350598 |
| ETV2 | CLDN4 | 0.850816942 |
| ETV2 | EHD4 | 0.80633305 |
| ETV2 | DTYMK | 0.779418148 |
| ETV2 | APLP1 | 0.751493324 |
| ETV2 | CALHM2 | 0.744296463 |
| ETV2 | LONRF1 | 0.728476165 |
| ETV2 | MPZL2 | 0.713191104 |
| ETV2 | ERH | 0.693113574 |
| ETV2 | PNKD | 0.659680877 |
| ETV2 | SLC16A1 | 0.648160627 |
| ETV2 | ITGB1 | 0.640634186 |
| ETV2 | PDCD6 | 0.638719255 |
| ETV2 | DCLK1 | 0.611601279 |
| ETV2 | ELN | 0.608643361 |
| ETV2 | WFDC1 | 0.60672651 |
| ETV2 | PAK2 | 0.601498236 |
| ETV2 | TMX2 | 0.582301931 |
| ETV2 | IRF2 | 0.579777195 |
| ETV2 | ARL1 | 0.569710947 |
| ETV2 | RPL22 | 0.568347391 |
| ETV2 | CENPP | 0.557171298 |
| ETV2 | RTN4 | 0.548821676 |
| ETV2 | CAV1 | 0.521726702 |
| ETV2 | SRCAP | 0.517432357 |
| ETV2 | SART3 | 0.504076702 |
| ETV2 | SRSF5 | 0.502295849 |
| ETV2 | DCTN2 | 0.491975596 |
| ETV2 | RAB32 | 0.490689061 |
| ETV2 | IER5 | 0.489466201 |
| ETV2 | TPM2 | 0.488897248 |
| ETV2 | ATP8B2 | 0.476699428 |
| ETV2 | SPPL2A | 0.467744201 |
| ETV2 | PRIM2 | 0.462555021 |
| ETV2 | COMMD1 | 0.45882019 |
| ETV2 | KRT16 | 0.455205011 |
| ETV2 | UQCRC2 | 0.447447513 |
| ETV2 | ANP32E | 0.447345499 |
| ETV2 | ECH1 | 0.443651908 |
| ETV2 | CDKN1C | 0.440136154 |
| ETV2 | EI24 | 0.435233148 |
| ETV2 | LRRC59 | 0.434591415 |
| ETV2 | C20orf24 | 0.426721719 |
| ETV2 | SKIV2L | 0.42396139 |
| ETV2 | CDC14B | 0.39834949 |
| ETV2 | MMADHC | 0.397923874 |
| ETV2 | MED25 | 0.38511423 |
| ETV2 | BCL6 | 0.384788575 |
| ETV2 | YPEL5 | 0.376365818 |
| ETV2 | ZNF529 | 0.373130512 |
| ETV2 | PUF60 | 0.372548939 |
| ETV2 | CAMSAP1 | 0.358363412 |
| ETV2 | RPL17 | 0.34315251 |
| ETV2 | RNASEH1 | 0.342025206 |
| ETV2 | ETF1 | 0.339469679 |
| ETV2 | PEX7 | 0.338720438 |
| ETV2 | GALE | 0.332983163 |
| ETV2 | FGF13 | 0.321486604 |
| ETV2 | ZNF687 | 0.321447727 |
| ETV2 | ATP6V0D1 | 0.29876751 |
| ETV2 | AGPAT2 | 0.284379397 |
| ETV2 | DNAJC14 | 0.281534066 |
| ETV2 | SLC39A6 | 0.273299453 |
| ETV2 | SLC29A1 | 0.271615742 |
| ETV2 | BFAR | 0.262168337 |
| ETV2 | CLTC | 0.260126986 |
| ETV2 | WDR1 | 0.258111704 |
| ETV2 | GRAMD1C | 0.254706004 |
| ETV2 | SRP54 | 0.249526022 |
| ETV2 | CD34 | 0.248031229 |
| ETV2 | PTGES | 0.238043124 |
| ETV2 | ENPP1 | 0.237235406 |
| ETV2 | SYP | 0.237105968 |
| ETV2 | EPOR | 0.234602733 |
| ETV2 | ZNF416 | 0.232846227 |
| ETV2 | MDFI | 0.230600423 |
| ETV2 | CCDC157 | 0.226761332 |
| ETV2 | EIF4B | 0.211305365 |
| ETV2 | HMOX2 | 0.21127041 |
| ETV2 | ATF6 | 0.20487356 |
| ETV2 | ATF7 | 0.187934304 |
| ETV2 | GLYR1 | 0.179617014 |
| ETV2 | CD2BP2 | 0.176086139 |
| ETV2 | LUC7L2 | 0.171837008 |
| ETV2 | RAB6A | 0.17074258 |
| ETV2 | PHACTR4 | 0.166183071 |
| ETV2 | SP2 | 0.152015446 |
| ETV2 | GALNT5 | 0.147195086 |
| ETV2 | SNAPC2 | 0.14684459 |
| ETV2 | RAB9A | 0.144685444 |
| ETV2 | PRKAB1 | 0.139522611 |
| ETV2 | ABCB7 | 0.136799968 |
| ETV2 | FAM122B | 0.136728379 |
| ETV2 | NRAS | 0.132161802 |
| ETV2 | ANXA11 | 0.131016122 |
| ETV2 | MEIS3 | 0.121818275 |
| ETV2 | LYSMD2 | 0.121015213 |
| ETV2 | POLR2J3 | 0.114497029 |
| ETV2 | ZNF502 | 0.103165252 |
| ETV2 | SENP5 | 0.094017495 |
| ETV2 | PARM1 | 0.089402616 |
| ETV2 | SPA17 | 0.086044864 |
| ETV2 | CENPJ | 0.085131241 |
| ETV2 | RNF25 | 0.076496877 |
| ETV2 | GFRA2 | 0.072743323 |
| ETV2 | METAP1D | 0.071026119 |
| ETV2 | HIST1H4A | 0.066565793 |
| ETV2 | OPHN1 | 0.056927532 |
| ETV2 | ZNF569 | 0.04896696 |
| ETV2 | DONSON | 0.047631614 |
| ETV2 | IZUMO4 | 0.037597822 |
| ETV2 | LRRK1 | 0.036319324 |
| ETV2 | LRP5L | 0.031501115 |
| ETV2 | C1orf112 | 0.02429973 |
| ETV3 | PRPF40A | 6.701111926 |
| ETV3 | KRT15 | 4.303450634 |
| ETV3 | ATP6V1E1 | 3.679993995 |
| ETV3 | SCMH1 | 3.621479266 |
| ETV3 | RPSA | 3.181169309 |
| ETV3 | UBN1 | 2.454013898 |
| ETV3 | TXNIP | 2.342070819 |
| ETV3 | TMEM144 | 2.18923204 |
| ETV3 | RAB13 | 2.048299009 |
| ETV3 | PTTG1IP | 2.005032097 |
| ETV3 | OST4 | 2.003070763 |
| ETV3 | TBX1 | 1.754227026 |
| ETV3 | GLCCI1 | 1.693311256 |
| ETV3 | LMAN2 | 1.675735803 |
| ETV3 | PNP | 1.620837523 |
| ETV3 | EEF1B2 | 1.554464429 |
| ETV3 | NUCKS1 | 1.552052802 |
| ETV3 | CAMK1 | 1.543687982 |
| ETV3 | ZNF267 | 1.514106691 |
| ETV3 | ZNF273 | 1.504238706 |
| ETV3 | ATF1 | 1.480617849 |
| ETV3 | GLE1 | 1.453226875 |
| ETV3 | DSP | 1.402213283 |
| ETV3 | S100A8 | 1.36682584 |
| ETV3 | PRMT6 | 1.331973248 |
| ETV3 | HMGA1 | 1.329248211 |
| ETV3 | CLTB | 1.277306659 |
| ETV3 | NCKAP1 | 1.243032564 |
| ETV3 | ST5 | 1.237210507 |
| ETV3 | TSTD1 | 1.232738349 |
| ETV3 | CSNK1A1 | 1.209677375 |
| ETV3 | LYN | 1.146013567 |
| ETV3 | PTDSS1 | 1.135428812 |
| ETV3 | POLR3B | 1.113411907 |
| ETV3 | NDUFA4 | 1.107069942 |
| ETV3 | RPL21 | 1.09452551 |
| ETV3 | SSR2 | 1.058498712 |
| ETV3 | PAPSS1 | 1.025670887 |
| ETV3 | KRTDAP | 1.024192595 |
| ETV3 | UBL5 | 1.020753842 |
| ETV3 | ZNF217 | 1.014270559 |
| ETV3 | ETV3 | 1 |
| ETV3 | LEPROTL1 | 0.962881096 |
| ETV3 | FOS | 0.960007712 |
| ETV3 | HDAC2 | 0.940317082 |
| ETV3 | PSMB7 | 0.916243969 |
| ETV3 | MAP7 | 0.913047397 |
| ETV3 | FOXK1 | 0.912074387 |
| ETV3 | RTN4 | 0.907119986 |
| ETV3 | RHOQ | 0.897762052 |
| ETV3 | UBE2L3 | 0.88492572 |
| ETV3 | CYB5R3 | 0.874294492 |
| ETV3 | SNHG6 | 0.873706317 |
| ETV3 | PLK1 | 0.870493747 |
| ETV3 | IGF1R | 0.858252019 |
| ETV3 | UQCRQ | 0.842286494 |
| ETV3 | EIF5A | 0.838420697 |
| ETV3 | ADRM1 | 0.832000312 |
| ETV3 | BZW1 | 0.829065861 |
| ETV3 | ABI1 | 0.822134141 |
| ETV3 | COX5B | 0.82102505 |
| ETV3 | EIF3H | 0.814795239 |
| ETV3 | MRPL14 | 0.802402177 |
| ETV3 | KIN | 0.787203579 |
| ETV3 | DMKN | 0.778389269 |
| ETV3 | TFPI2 | 0.778035536 |
| ETV3 | FAM110A | 0.764047714 |
| ETV3 | COX4I1 | 0.752022637 |
| ETV3 | HSP90AA1 | 0.74593617 |
| ETV3 | TP53I11 | 0.739369946 |
| ETV3 | XRRA1 | 0.731151285 |
| ETV3 | RPL29 | 0.724485226 |
| ETV3 | SMARCA1 | 0.686110984 |
| ETV3 | CLUAP1 | 0.669026308 |
| ETV3 | MRPL47 | 0.667118514 |
| ETV3 | RASL11A | 0.658885395 |
| ETV3 | CSGALNACT2 | 0.656941322 |
| ETV3 | MLLT3 | 0.653981791 |
| ETV3 | POLR2F | 0.651884805 |
| ETV3 | RING1 | 0.64856961 |
| ETV3 | RGS16 | 0.639031302 |
| ETV3 | SYN1 | 0.628611355 |
| ETV3 | ACTR8 | 0.625736823 |
| ETV3 | SH3BGRL3 | 0.62296341 |
| ETV3 | GTPBP1 | 0.614056921 |
| ETV3 | OLFML2B | 0.563301068 |
| ETV3 | SSSCA1 | 0.56209671 |
| ETV3 | MEF2C | 0.560023292 |
| ETV3 | DUSP5 | 0.557796225 |
| ETV3 | LY6D | 0.554591626 |
| ETV3 | CYB5A | 0.546105491 |
| ETV3 | COMMD3 | 0.541652825 |
| ETV3 | MRPL18 | 0.536802468 |
| ETV3 | SLC4A1AP | 0.53460978 |
| ETV3 | FABP5 | 0.524265674 |
| ETV3 | AGA | 0.523915026 |
| ETV3 | IER3 | 0.523046665 |
| ETV3 | GSTM3 | 0.522029509 |
| ETV3 | DNAJB1 | 0.520924249 |
| ETV3 | RPL41 | 0.520386591 |
| ETV3 | MAP1LC3B | 0.517610565 |
| ETV3 | MMP15 | 0.510256716 |
| ETV3 | SNRPC | 0.505017714 |
| ETV3 | DCTN2 | 0.503195412 |
| ETV3 | PCDH11X | 0.50190938 |
| ETV3 | ISLR | 0.500771711 |
| ETV3 | SDCCAG8 | 0.500451301 |
| ETV3 | CLIP1 | 0.499095108 |
| ETV3 | ZCWPW2 | 0.497779747 |
| ETV3 | VIPR2 | 0.496625227 |
| ETV3 | SEC61G | 0.490941027 |
| ETV3 | MT2A | 0.483757294 |
| ETV3 | FRS3 | 0.482402245 |
| ETV3 | CTTN | 0.47942785 |
| ETV3 | CIR1 | 0.479260166 |
| ETV3 | FYCO1 | 0.478650531 |
| ETV3 | CIB2 | 0.477979769 |
| ETV3 | PNPLA8 | 0.47797969 |
| ETV3 | SAV1 | 0.472267106 |
| ETV3 | NOL12 | 0.470617349 |
| ETV3 | ID4 | 0.464855939 |
| ETV3 | SERP1 | 0.461188299 |
| ETV3 | FKBP1A | 0.451457172 |
| ETV3 | LRRC29 | 0.438253534 |
| ETV3 | TXNDC12 | 0.437849559 |
| ETV3 | FKBP7 | 0.434602028 |
| ETV3 | LRRC41 | 0.425723156 |
| ETV3 | TAF4 | 0.412374984 |
| ETV3 | WNT5A | 0.405156092 |
| ETV3 | DBR1 | 0.402963146 |
| ETV3 | PLXNA2 | 0.393906437 |
| ETV3 | CCNYL1 | 0.391092806 |
| ETV3 | BARD1 | 0.375808988 |
| ETV3 | PIK3C2A | 0.360705656 |
| ETV3 | CYTH1 | 0.359709256 |
| ETV3 | NPEPL1 | 0.359618119 |
| ETV3 | NOC3L | 0.354173084 |
| ETV3 | MANBAL | 0.352300655 |
| ETV3 | PPIG | 0.340728916 |
| ETV3 | KIAA0319L | 0.335563338 |
| ETV3 | PGGT1B | 0.327602837 |
| ETV3 | SOS2 | 0.326912654 |
| ETV3 | ASB6 | 0.321177879 |
| ETV3 | SHARPIN | 0.318894268 |
| ETV3 | PHKB | 0.306643055 |
| ETV3 | ZNF197 | 0.30440813 |
| ETV3 | CNTN2 | 0.293761158 |
| ETV3 | BCOR | 0.289072246 |
| ETV3 | ARL8A | 0.280870248 |
| ETV3 | IMMP2L | 0.275683311 |
| ETV3 | MGMT | 0.27363186 |
| ETV3 | MTUS1 | 0.271284873 |
| ETV3 | CPT2 | 0.268799235 |
| ETV3 | SSBP4 | 0.264234336 |
| ETV3 | CTNND1 | 0.260063206 |
| ETV3 | ZNF274 | 0.252595812 |
| ETV3 | PMVK | 0.250062188 |
| ETV3 | ZNF780B | 0.246572453 |
| ETV3 | EIF2B2 | 0.246224352 |
| ETV3 | ADAT1 | 0.244632035 |
| ETV3 | SORBS2 | 0.244442769 |
| ETV3 | CLDN5 | 0.235770791 |
| ETV3 | ZNF276 | 0.229205091 |
| ETV3 | PGPEP1 | 0.229127719 |
| ETV3 | PRMT3 | 0.226785053 |
| ETV3 | AAAS | 0.225933054 |
| ETV3 | VPS45 | 0.221494985 |
| ETV3 | ZNF524 | 0.21961797 |
| ETV3 | RAB27B | 0.217062353 |
| ETV3 | VCPIP1 | 0.215954379 |
| ETV3 | MITF | 0.213789614 |
| ETV3 | SLC39A8 | 0.2122005 |
| ETV3 | ZNF395 | 0.211855615 |
| ETV3 | MEGF8 | 0.207182027 |
| ETV3 | TAOK3 | 0.20599421 |
| ETV3 | MARK2 | 0.204460622 |
| ETV3 | FUK | 0.203748848 |
| ETV3 | EHD3 | 0.200732272 |
| ETV3 | PHF11 | 0.19962148 |
| ETV3 | LGALS8 | 0.189994466 |
| ETV3 | ZNF394 | 0.18871044 |
| ETV3 | LPIN2 | 0.187823868 |
| ETV3 | ZC3H4 | 0.185495345 |
| ETV3 | MEGF9 | 0.184386536 |
| ETV3 | SIRT7 | 0.183869168 |
| ETV3 | CDH13 | 0.182705845 |
| ETV3 | CAPZA1 | 0.180700941 |
| ETV3 | VPS41 | 0.180341178 |
| ETV3 | SOCS2 | 0.169804235 |
| ETV3 | NOC2L | 0.167977964 |
| ETV3 | BCL2L11 | 0.158291745 |
| ETV3 | MAX | 0.155350743 |
| ETV3 | CCDC102A | 0.153373295 |
| ETV3 | KIF14 | 0.153238373 |
| ETV3 | AP3M1 | 0.151645609 |
| ETV3 | FUCA1 | 0.148241048 |
| ETV3 | OVOL1 | 0.144818778 |
| ETV3 | RBM23 | 0.142342365 |
| ETV3 | TAF13 | 0.142035592 |
| ETV3 | ZNF235 | 0.141068123 |
| ETV3 | RGL2 | 0.134006337 |
| ETV3 | FUBP3 | 0.126789109 |
| ETV3 | ARHGAP44 | 0.123425045 |
| ETV3 | NSUN5 | 0.122128273 |
| ETV3 | CCDC115 | 0.094574857 |
| ETV3 | SRC | 0.082989751 |
| ETV3 | PLCD3 | 0.075210365 |
| ETV3 | NFKBIE | 0.055880159 |
| ETV3 | PDGFD | 0.016262231 |
| ETV3 | USP8 | 0.010899802 |
| ETV3 | RAB9A | 0.006286951 |
| ETV3 | FAM46C | 0.005527055 |
| ETV3 | POLRMT | 0.004682988 |
| ETV3 | ZNF263 | 0.003057611 |
| ETV3 | YIPF2 | 0.001388602 |
| ETV3 | TOM1 | 0.000134326 |
| ETV3 | ZNF655 | 0.000129616 |
| ETV3 | OSBP | 2.36E-05 |
| ETV4 | NR1H2 | 1.268816012 |
| ETV4 | STIM2 | 0.850537018 |
| ETV4 | OSR1 | 0.847080588 |
| ETV4 | NFIB | 0.473497648 |
| ETV4 | DENR | 0.235797611 |
| ETV4 | SSBP2 | 0.235670765 |
| ETV4 | EIF4G3 | 0.213421351 |
| ETV4 | BOC | 0.208104714 |
| ETV4 | NFKB2 | 0.111100431 |
| ETV4 | PPFIA2 | 0.062000108 |
| ETV5 | LMNB1 | 5.950722814 |
| ETV5 | ANPEP | 2.274866212 |
| ETV5 | ZCCHC11 | 2.183656081 |
| ETV5 | HUNK | 1.948629436 |
| ETV5 | FST | 1.526445407 |
| ETV5 | TTC26 | 1.3621264 |
| ETV5 | KCND3 | 1.360452179 |
| ETV5 | RILPL2 | 1.359553673 |
| ETV5 | ZBTB4 | 1.271923167 |
| ETV5 | ZNHIT6 | 1.242437986 |
| ETV5 | SLC39A9 | 1.222118274 |
| ETV5 | NETO2 | 1.194708977 |
| ETV5 | DRAM1 | 1.192663963 |
| ETV5 | SLIT2 | 1.168873391 |
| ETV5 | PDGFB | 1.053883129 |
| ETV5 | NGDN | 0.995049408 |
| ETV5 | ZNF827 | 0.938604908 |
| ETV5 | UGCG | 0.935342661 |
| ETV5 | ARSA | 0.854697763 |
| ETV5 | KIAA1522 | 0.833228663 |
| ETV5 | GJB2 | 0.771550914 |
| ETV5 | SAMD8 | 0.769794619 |
| ETV5 | NDUFB5 | 0.74982507 |
| ETV5 | CELF4 | 0.716269936 |
| ETV5 | WLS | 0.694795641 |
| ETV5 | SEC23IP | 0.638870181 |
| ETV5 | KRIT1 | 0.558447425 |
| ETV5 | WDR45 | 0.510350846 |
| ETV5 | PPIP5K1 | 0.460971706 |
| ETV5 | CDC42EP2 | 0.43244669 |
| ETV5 | TMEM150A | 0.422035345 |
| ETV5 | PCGF2 | 0.418056147 |
| ETV5 | ORC2 | 0.410441443 |
| ETV5 | FOXP2 | 0.39263092 |
| ETV5 | CLK2 | 0.363054365 |
| ETV5 | NFKB2 | 0.351474011 |
| ETV5 | WSB2 | 0.349089854 |
| ETV5 | TFAM | 0.337608981 |
| ETV5 | DNAJB14 | 0.328427435 |
| ETV5 | RAB11FIP5 | 0.316766136 |
| ETV5 | ZNF496 | 0.274714324 |
| ETV5 | UBE2M | 0.270015477 |
| ETV5 | SLCO3A1 | 0.247933665 |
| ETV5 | MFN1 | 0.242633452 |
| ETV5 | ZBTB7A | 0.221499953 |
| ETV5 | ZDHHC8 | 0.211642271 |
| ETV5 | INTS3 | 0.182633409 |
| ETV5 | ZBTB16 | 0.18219572 |
| ETV5 | MAML1 | 0.180457944 |
| ETV5 | MTA2 | 0.177222479 |
| ETV5 | THAP5 | 0.158023544 |
| ETV5 | EBF2 | 0.153818588 |
| ETV5 | S1PR2 | 0.151753808 |
| ETV5 | CAMK1D | 0.151319453 |
| ETV5 | SETD3 | 0.14549469 |
| ETV5 | YES1 | 0.140749627 |
| ETV5 | INPPL1 | 0.135295694 |
| ETV5 | ACBD5 | 0.134455197 |
| ETV5 | RNF38 | 0.125714328 |
| ETV5 | NR1H2 | 0.119228679 |
| ETV5 | GPR85 | 0.113514391 |
| ETV5 | KCTD2 | 0.092259704 |
| ETV5 | KBTBD2 | 0.08291987 |
| ETV5 | CLIC6 | 0.077505843 |
| ETV5 | NIF3L1 | 0.063401217 |
| ETV5 | SH2D2A | 0.052873307 |
| ETV5 | NOL6 | 0.05022832 |
| ETV6 | GSTM3 | 5.976387457 |
| ETV6 | SUN2 | 4.627268425 |
| ETV6 | HBEGF | 3.157314282 |
| ETV6 | CD59 | 3.153276722 |
| ETV6 | WDR7 | 2.754802408 |
| ETV6 | SETBP1 | 2.713104915 |
| ETV6 | TJP1 | 2.444535521 |
| ETV6 | PHLDB1 | 2.334645085 |
| ETV6 | SPTAN1 | 2.202043681 |
| ETV6 | GLT8D2 | 1.809583087 |
| ETV6 | ARL13B | 1.758057058 |
| ETV6 | EHD4 | 1.702475977 |
| ETV6 | EIF3M | 1.611845962 |
| ETV6 | RALGAPA1 | 1.473156677 |
| ETV6 | PCDH19 | 1.379613957 |
| ETV6 | AHNAK | 1.359929249 |
| ETV6 | SUGT1 | 1.334792786 |
| ETV6 | BIRC6 | 1.329534819 |
| ETV6 | MAPK11 | 1.241313793 |
| ETV6 | CCDC97 | 1.208365568 |
| ETV6 | SUPT5H | 1.146926253 |
| ETV6 | C7orf43 | 1.092988879 |
| ETV6 | MIDN | 1.084957654 |
| ETV6 | EIF4B | 1.045320708 |
| ETV6 | ADCY3 | 1.041888046 |
| ETV6 | CHORDC1 | 1.015504078 |
| ETV6 | ETV6 | 1 |
| ETV6 | DGAT1 | 0.995507223 |
| ETV6 | MTMR11 | 0.991045987 |
| ETV6 | LRRC8A | 0.943587082 |
| ETV6 | FOXJ2 | 0.873754238 |
| ETV6 | EFHC1 | 0.872380891 |
| ETV6 | PLCB4 | 0.78051168 |
| ETV6 | SRFBP1 | 0.739836795 |
| ETV6 | LNPEP | 0.710908504 |
| ETV6 | PHC2 | 0.694461397 |
| ETV6 | ARHGAP26 | 0.685532678 |
| ETV6 | BTBD9 | 0.684945265 |
| ETV6 | UBN2 | 0.675252214 |
| ETV6 | PLAT | 0.61738128 |
| ETV6 | FSD1 | 0.603990588 |
| ETV6 | IL17D | 0.601894931 |
| ETV6 | SUPT7L | 0.565376322 |
| ETV6 | ERP27 | 0.554372999 |
| ETV6 | ZNF234 | 0.458995841 |
| ETV6 | HOXD1 | 0.444204362 |
| ETV6 | PHRF1 | 0.42933571 |
| ETV6 | SPTLC2 | 0.423194072 |
| ETV6 | HOXA4 | 0.275637395 |
| ETV7 | CTSH | 8.751597955 |
| ETV7 | DDOST | 4.819149953 |
| ETV7 | CP | 4.383191574 |
| ETV7 | VCAM1 | 3.302364421 |
| ETV7 | TMEM214 | 2.534927167 |
| ETV7 | ZNF600 | 2.22364861 |
| ETV7 | G3BP1 | 2.120723582 |
| ETV7 | TNFSF10 | 2.086345325 |
| ETV7 | IFI6 | 2.031154311 |
| ETV7 | HSPBP1 | 1.967374736 |
| ETV7 | FAM3A | 1.921506647 |
| ETV7 | LLGL2 | 1.87818447 |
| ETV7 | ERF | 1.713204057 |
| ETV7 | ZNF143 | 1.595968113 |
| ETV7 | PUSL1 | 1.59464358 |
| ETV7 | GLYR1 | 1.427378071 |
| ETV7 | CASP1 | 1.393255229 |
| ETV7 | C2orf42 | 1.376686764 |
| ETV7 | LITAF | 1.325572455 |
| ETV7 | MRPL16 | 1.29018205 |
| ETV7 | FAM46A | 1.263578707 |
| ETV7 | SUMO3 | 1.229211309 |
| ETV7 | DECR1 | 1.212580464 |
| ETV7 | PSD | 1.21205372 |
| ETV7 | C7 | 1.191340178 |
| ETV7 | PGF | 1.186971498 |
| ETV7 | FBXO48 | 1.141487365 |
| ETV7 | DPH5 | 1.122501392 |
| ETV7 | PTK2 | 1.054797557 |
| ETV7 | GDI1 | 1.054715722 |
| ETV7 | ETV7 | 1 |
| ETV7 | TP63 | 0.97562269 |
| ETV7 | RCC2 | 0.962610175 |
| ETV7 | TPRKB | 0.959551528 |
| ETV7 | MRPL51 | 0.956851056 |
| ETV7 | TSEN34 | 0.950934418 |
| ETV7 | CTSS | 0.935897006 |
| ETV7 | AKT1S1 | 0.928890452 |
| ETV7 | AP3D1 | 0.924116497 |
| ETV7 | PITPNA | 0.893215423 |
| ETV7 | SRSF8 | 0.891269204 |
| ETV7 | RTN4RL2 | 0.883547723 |
| ETV7 | C1QTNF6 | 0.870980776 |
| ETV7 | PSME2 | 0.837483645 |
| ETV7 | MDH2 | 0.832726746 |
| ETV7 | TMEM167A | 0.822759791 |
| ETV7 | UNC13C | 0.787593873 |
| ETV7 | PLD2 | 0.720865311 |
| ETV7 | LSM3 | 0.705330219 |
| ETV7 | NTHL1 | 0.674268639 |
| ETV7 | MRPL54 | 0.643400823 |
| ETV7 | VPS36 | 0.627279757 |
| ETV7 | TAF11 | 0.616106065 |
| ETV7 | OLFM2 | 0.570758651 |
| ETV7 | COX6A1 | 0.563227464 |
| ETV7 | NUDT5 | 0.52853749 |
| ETV7 | EVI5L | 0.491279062 |
| ETV7 | ERRFI1 | 0.480465101 |
| ETV7 | TRA2B | 0.479968623 |
| ETV7 | NIPA2 | 0.479803539 |
| ETV7 | ROCK1 | 0.476464836 |
| ETV7 | ATXN7L3B | 0.456122446 |
| ETV7 | SRBD1 | 0.450649181 |
| ETV7 | TRAM1 | 0.435575738 |
| ETV7 | CCDC122 | 0.402850248 |
| ETV7 | ARL2BP | 0.394366847 |
| ETV7 | IQSEC2 | 0.393993596 |
| ETV7 | BIRC3 | 0.358091165 |
| ETV7 | DCP2 | 0.32724556 |
| ETV7 | CD200R1 | 0.324502656 |
| ETV7 | NPHP4 | 0.321006473 |
| ETV7 | ABCB7 | 0.318999027 |
| ETV7 | RRP15 | 0.317375005 |
| ETV7 | ZNF529 | 0.30637921 |
| ETV7 | ZDHHC8 | 0.299022924 |
| ETV7 | EPSTI1 | 0.294511685 |
| ETV7 | CD40 | 0.291874048 |
| ETV7 | UNC50 | 0.284960563 |
| ETV7 | ANGEL1 | 0.274239366 |
| ETV7 | ENOSF1 | 0.251597195 |
| ETV7 | TGIF2 | 0.248495554 |
| ETV7 | ABI1 | 0.238293881 |
| ETV7 | TPP2 | 0.238231579 |
| ETV7 | ATP6V1C1 | 0.238071197 |
| ETV7 | SP140L | 0.231946184 |
| ETV7 | CCDC50 | 0.22354809 |
| ETV7 | ANK1 | 0.223155561 |
| ETV7 | GRB2 | 0.221845506 |
| ETV7 | EPAS1 | 0.221185145 |
| ETV7 | MRPS5 | 0.214976065 |
| ETV7 | CACNB2 | 0.212096398 |
| ETV7 | UGDH | 0.201875091 |
| ETV7 | FLI1 | 0.191337272 |
| ETV7 | UNC45A | 0.173799092 |
| ETV7 | HOPX | 0.157682098 |
| ETV7 | NR1H2 | 0.151620006 |
| ETV7 | RB1CC1 | 0.148996459 |
| ETV7 | ALAD | 0.146591516 |
| ETV7 | B4GALT7 | 0.145426374 |
| ETV7 | TNFRSF1B | 0.14485291 |
| ETV7 | ZNF358 | 0.12731394 |
| ETV7 | SSTR2 | 0.108500555 |
| ETV7 | EN1 | 0.107177976 |
| ETV7 | VPS52 | 0.105978121 |
| ETV7 | DDX49 | 0.104246265 |
| ETV7 | DTNA | 0.104157662 |
| ETV7 | NR2F6 | 0.099656708 |
| ETV7 | ZNF611 | 0.089409117 |
| ETV7 | PIGU | 0.088595068 |
| ETV7 | HOXB6 | 0.082964826 |
| ETV7 | SPESP1 | 0.081753921 |
| ETV7 | ALPK2 | 0.076527901 |
| ETV7 | ALKBH1 | 0.076249061 |
| ETV7 | EME2 | 0.047600606 |
| ETV7 | CC2D1B | 0.045674995 |
| ETV7 | MKS1 | 0.043967318 |
| ETV7 | GNA14 | 0.029484961 |
| EZH2 | KIF11 | 10.0115483 |
| EZH2 | NCAPG | 9.896425476 |
| EZH2 | MKI67 | 9.663230083 |
| EZH2 | ILF3 | 9.409329939 |
| EZH2 | HMGB3 | 7.024707729 |
| EZH2 | MELK | 6.892558214 |
| EZH2 | MEF2C | 6.500811412 |
| EZH2 | CCNA2 | 6.369750829 |
| EZH2 | ILF2 | 6.115917322 |
| EZH2 | TMEM106C | 5.743324805 |
| EZH2 | KIFC1 | 5.508797775 |
| EZH2 | ZWINT | 5.342007958 |
| EZH2 | HELLS | 5.338261303 |
| EZH2 | STMN1 | 5.224167941 |
| EZH2 | PKMYT1 | 5.178891233 |
| EZH2 | COL11A1 | 5.1142422 |
| EZH2 | KIF2C | 4.983116965 |
| EZH2 | H2AFV | 4.914836832 |
| EZH2 | HMGB2 | 4.850762742 |
| EZH2 | MAD2L1 | 4.566277185 |
| EZH2 | NUF2 | 4.518993024 |
| EZH2 | KIF15 | 4.438414302 |
| EZH2 | HAPLN1 | 4.423441829 |
| EZH2 | H2AFZ | 4.330847346 |
| EZH2 | TK1 | 4.24530881 |
| EZH2 | TOP2A | 4.201754803 |
| EZH2 | SPC25 | 4.11914095 |
| EZH2 | CEP55 | 3.942625643 |
| EZH2 | MCM10 | 3.776520189 |
| EZH2 | NCAPH | 3.658900442 |
| EZH2 | HAT1 | 3.583812354 |
| EZH2 | CDCA5 | 3.575942944 |
| EZH2 | TPR | 3.32038994 |
| EZH2 | MND1 | 3.117780707 |
| EZH2 | CCDC34 | 2.990652882 |
| EZH2 | SNRPA | 2.956153189 |
| EZH2 | TWISTNB | 2.926697802 |
| EZH2 | PSRC1 | 2.92385906 |
| EZH2 | SYNCRIP | 2.915819276 |
| EZH2 | HADH | 2.833572883 |
| EZH2 | PRC1 | 2.628614228 |
| EZH2 | HIF1A | 2.627649857 |
| EZH2 | LRRC47 | 2.426847262 |
| EZH2 | ERI3 | 2.404058461 |
| EZH2 | RBBP7 | 2.39273359 |
| EZH2 | PSMC6 | 2.39210251 |
| EZH2 | ATXN10 | 2.36737274 |
| EZH2 | NCAPD2 | 2.345279546 |
| EZH2 | APH1B | 2.322340685 |
| EZH2 | CANT1 | 2.265535224 |
| EZH2 | TADA3 | 2.24409443 |
| EZH2 | FAM136A | 2.229943926 |
| EZH2 | CDCA8 | 2.157628006 |
| EZH2 | ARHGAP15 | 2.145566959 |
| EZH2 | RANBP1 | 2.134647452 |
| EZH2 | SNRPC | 2.12771829 |
| EZH2 | FBXO7 | 2.122138136 |
| EZH2 | ASF1B | 2.102271578 |
| EZH2 | HMGB1 | 2.096183962 |
| EZH2 | KPNA2 | 2.09255383 |
| EZH2 | SRSF2 | 2.08874503 |
| EZH2 | PAQR4 | 2.064029531 |
| EZH2 | FBXO5 | 1.957261747 |
| EZH2 | PARD3 | 1.938270596 |
| EZH2 | GINS2 | 1.924154591 |
| EZH2 | GRTP1 | 1.909257485 |
| EZH2 | NCAPG2 | 1.905380954 |
| EZH2 | MSH2 | 1.900450775 |
| EZH2 | WDR6 | 1.894775075 |
| EZH2 | FAM111B | 1.892605364 |
| EZH2 | ZNF367 | 1.850591055 |
| EZH2 | PLEKHF2 | 1.827978058 |
| EZH2 | AKAP10 | 1.812554484 |
| EZH2 | ANP32E | 1.810235846 |
| EZH2 | YTHDC1 | 1.804039402 |
| EZH2 | SMC6 | 1.781647829 |
| EZH2 | TYMS | 1.77417281 |
| EZH2 | YIF1A | 1.774047879 |
| EZH2 | SCFD2 | 1.751834132 |
| EZH2 | DEK | 1.720452154 |
| EZH2 | PEX3 | 1.691207933 |
| EZH2 | NCL | 1.681082507 |
| EZH2 | SNRPD3 | 1.668096528 |
| EZH2 | MDH1 | 1.658833344 |
| EZH2 | NDUFA10 | 1.649077355 |
| EZH2 | EMP2 | 1.610957409 |
| EZH2 | MASTL | 1.606850091 |
| EZH2 | GNB4 | 1.602719191 |
| EZH2 | ARMC5 | 1.559079004 |
| EZH2 | MESP1 | 1.535840441 |
| EZH2 | FAM111A | 1.531801774 |
| EZH2 | CDC6 | 1.512926785 |
| EZH2 | CTPS2 | 1.511654815 |
| EZH2 | CDKN2D | 1.51093627 |
| EZH2 | DERL3 | 1.484975324 |
| EZH2 | BOLA3 | 1.437420773 |
| EZH2 | E2F8 | 1.396205611 |
| EZH2 | FUNDC1 | 1.392406215 |
| EZH2 | PTTG1 | 1.363053888 |
| EZH2 | PPP1CC | 1.343062507 |
| EZH2 | PCBP1 | 1.323423392 |
| EZH2 | SYNGR1 | 1.317087413 |
| EZH2 | FUT11 | 1.303860502 |
| EZH2 | PGAM5 | 1.244443369 |
| EZH2 | PDIK1L | 1.243658373 |
| EZH2 | USP1 | 1.233361599 |
| EZH2 | UCKL1 | 1.220696877 |
| EZH2 | RANBP9 | 1.186967248 |
| EZH2 | PLK2 | 1.182390496 |
| EZH2 | RCN2 | 1.169101728 |
| EZH2 | KIAA1586 | 1.162297846 |
| EZH2 | TUBA1B | 1.15373533 |
| EZH2 | EDC3 | 1.152130583 |
| EZH2 | NDUFS8 | 1.150206698 |
| EZH2 | LDHB | 1.149520004 |
| EZH2 | ILK | 1.126880694 |
| EZH2 | RBMX | 1.125966769 |
| EZH2 | MIR155HG | 1.122964895 |
| EZH2 | PALLD | 1.117753328 |
| EZH2 | TFPT | 1.103242603 |
| EZH2 | YWHAE | 1.101658196 |
| EZH2 | SRSF10 | 1.086951011 |
| EZH2 | CRISPLD2 | 1.085442692 |
| EZH2 | ADA | 1.083648785 |
| EZH2 | CENPK | 1.069461477 |
| EZH2 | ATAD2 | 1.061622411 |
| EZH2 | UNG | 1.05299378 |
| EZH2 | SKA3 | 1.046985901 |
| EZH2 | CASP7 | 1.038737959 |
| EZH2 | AKAP8 | 1.016600953 |
| EZH2 | CEP70 | 1.010454826 |
| EZH2 | MON1A | 1.004585381 |
| EZH2 | ZNF561 | 1.000410248 |
| EZH2 | EZH2 | 1 |
| EZH2 | B3GNT2 | 0.990684243 |
| EZH2 | CYB5R2 | 0.989494325 |
| EZH2 | HOXB4 | 0.980685459 |
| EZH2 | SMC2 | 0.976316994 |
| EZH2 | RBP4 | 0.961224043 |
| EZH2 | XRCC5 | 0.95216549 |
| EZH2 | HNRNPA3 | 0.93621594 |
| EZH2 | CYP51A1 | 0.922713277 |
| EZH2 | LMF1 | 0.913039229 |
| EZH2 | PIGU | 0.892610876 |
| EZH2 | VSNL1 | 0.891800119 |
| EZH2 | GAMT | 0.875128327 |
| EZH2 | PTGES3 | 0.874534256 |
| EZH2 | VPS37C | 0.870425454 |
| EZH2 | GPX7 | 0.865008699 |
| EZH2 | HOMER2 | 0.858910623 |
| EZH2 | MARK3 | 0.85710968 |
| EZH2 | NDUFB11 | 0.855290294 |
| EZH2 | ALDH7A1 | 0.850938647 |
| EZH2 | PCBP2 | 0.847688221 |
| EZH2 | CCNB2 | 0.837145224 |
| EZH2 | UQCRC1 | 0.836385665 |
| EZH2 | CFDP1 | 0.824864098 |
| EZH2 | CENPA | 0.82050192 |
| EZH2 | RFC3 | 0.816248611 |
| EZH2 | IL20RB | 0.816247726 |
| EZH2 | TTC13 | 0.809835324 |
| EZH2 | CCNF | 0.8029295 |
| EZH2 | CHD3 | 0.799020244 |
| EZH2 | PTMA | 0.791822156 |
| EZH2 | SIVA1 | 0.780089789 |
| EZH2 | IVNS1ABP | 0.766547361 |
| EZH2 | ATF6B | 0.763210164 |
| EZH2 | MYBL1 | 0.736355599 |
| EZH2 | GNAO1 | 0.735772614 |
| EZH2 | SNRPB | 0.734599323 |
| EZH2 | UBE2I | 0.734119625 |
| EZH2 | AGFG1 | 0.730511429 |
| EZH2 | PRPF4 | 0.72822107 |
| EZH2 | YWHAZ | 0.72713349 |
| EZH2 | SMC1A | 0.725978179 |
| EZH2 | ARID4B | 0.718735468 |
| EZH2 | CHCHD10 | 0.718247832 |
| EZH2 | PKNOX1 | 0.714505753 |
| EZH2 | HNRNPA0 | 0.7104666 |
| EZH2 | MRPS18C | 0.709658002 |
| EZH2 | FBXO16 | 0.705661799 |
| EZH2 | RWDD1 | 0.701745127 |
| EZH2 | RUNX3 | 0.700179031 |
| EZH2 | COQ7 | 0.699761979 |
| EZH2 | SUMO1 | 0.69956371 |
| EZH2 | PAFAH1B3 | 0.697282624 |
| EZH2 | CENPM | 0.696154944 |
| EZH2 | MYO9B | 0.692654685 |
| EZH2 | IDH2 | 0.692302255 |
| EZH2 | ID4 | 0.691006311 |
| EZH2 | ETF1 | 0.689369735 |
| EZH2 | SPCS3 | 0.686577162 |
| EZH2 | KIF5B | 0.678661608 |
| EZH2 | PRPS2 | 0.676357655 |
| EZH2 | DAP | 0.67389493 |
| EZH2 | ID1 | 0.649605345 |
| EZH2 | CCT7 | 0.638466219 |
| EZH2 | DCAF12 | 0.636232384 |
| EZH2 | RNF4 | 0.626827002 |
| EZH2 | SNAI2 | 0.62386143 |
| EZH2 | TUBB6 | 0.623457842 |
| EZH2 | DUSP4 | 0.620951242 |
| EZH2 | MZT1 | 0.620481557 |
| EZH2 | CACNA1A | 0.61703572 |
| EZH2 | NID2 | 0.613688818 |
| EZH2 | TAF9B | 0.612117445 |
| EZH2 | ANKRD50 | 0.61051576 |
| EZH2 | PGD | 0.61014031 |
| EZH2 | SRSF4 | 0.608226309 |
| EZH2 | MRPL51 | 0.607610563 |
| EZH2 | TINF2 | 0.606762349 |
| EZH2 | RBBP9 | 0.604063569 |
| EZH2 | UBE2T | 0.602268612 |
| EZH2 | DDX54 | 0.598535327 |
| EZH2 | COX19 | 0.595326095 |
| EZH2 | HECTD1 | 0.593718804 |
| EZH2 | SLC25A40 | 0.592404906 |
| EZH2 | ZNF655 | 0.578838614 |
| EZH2 | EIF2S1 | 0.575727524 |
| EZH2 | HDGF | 0.575220714 |
| EZH2 | KHDRBS1 | 0.574539457 |
| EZH2 | KDELR3 | 0.572382435 |
| EZH2 | WDHD1 | 0.569733079 |
| EZH2 | MFSD11 | 0.567345318 |
| EZH2 | RALY | 0.566407705 |
| EZH2 | KPNB1 | 0.565735997 |
| EZH2 | TUBB | 0.562330144 |
| EZH2 | TRA2B | 0.55913739 |
| EZH2 | PGRMC1 | 0.55870576 |
| EZH2 | THADA | 0.557996931 |
| EZH2 | GOLM1 | 0.55450415 |
| EZH2 | RAB34 | 0.543694803 |
| EZH2 | GPSM2 | 0.537440744 |
| EZH2 | ATP5S | 0.533664252 |
| EZH2 | HMGN2 | 0.53219293 |
| EZH2 | CALM3 | 0.520252426 |
| EZH2 | CDV3 | 0.515796168 |
| EZH2 | ANAPC11 | 0.50922197 |
| EZH2 | DDX55 | 0.499121674 |
| EZH2 | SIRT2 | 0.498035713 |
| EZH2 | RPP25 | 0.493505265 |
| EZH2 | C4orf3 | 0.487523966 |
| EZH2 | RRM1 | 0.487375806 |
| EZH2 | FBXL5 | 0.482946136 |
| EZH2 | CSNK1A1 | 0.479481906 |
| EZH2 | YRDC | 0.479051592 |
| EZH2 | TRMT2A | 0.476705217 |
| EZH2 | PRDX2 | 0.476348964 |
| EZH2 | HSPD1 | 0.472744737 |
| EZH2 | ZNF7 | 0.472193288 |
| EZH2 | UBE2Q2P1 | 0.469220221 |
| EZH2 | MGAT1 | 0.468930173 |
| EZH2 | SLC16A9 | 0.465214425 |
| EZH2 | KCNIP1 | 0.464095531 |
| EZH2 | SUGP1 | 0.453508759 |
| EZH2 | CDK5RAP1 | 0.452817101 |
| EZH2 | RAPH1 | 0.452175178 |
| EZH2 | ATP6V1H | 0.448473224 |
| EZH2 | C11orf58 | 0.448422632 |
| EZH2 | ABHD3 | 0.447331794 |
| EZH2 | TMEM209 | 0.439866958 |
| EZH2 | GINS4 | 0.438846976 |
| EZH2 | DEF8 | 0.415073886 |
| EZH2 | ESYT1 | 0.413770034 |
| EZH2 | ZNF227 | 0.411045865 |
| EZH2 | PKD2 | 0.408018834 |
| EZH2 | RPIA | 0.397836626 |
| EZH2 | LRRC1 | 0.388984993 |
| EZH2 | SPATA20 | 0.381202741 |
| EZH2 | TEX14 | 0.367684874 |
| EZH2 | TTF2 | 0.364518759 |
| EZH2 | ORC6 | 0.361851937 |
| EZH2 | LIN52 | 0.359923905 |
| EZH2 | PRIM1 | 0.344133753 |
| EZH2 | LCOR | 0.343812204 |
| EZH2 | MKL1 | 0.34210657 |
| EZH2 | FMNL3 | 0.341577698 |
| EZH2 | RFXAP | 0.339747988 |
| EZH2 | HMG20B | 0.332177668 |
| EZH2 | DUS3L | 0.330088599 |
| EZH2 | PPP2R3C | 0.321080946 |
| EZH2 | SLC29A4 | 0.320334841 |
| EZH2 | TMPO | 0.316395453 |
| EZH2 | IQGAP2 | 0.314038867 |
| EZH2 | TLE2 | 0.313478652 |
| EZH2 | KDELC1 | 0.309642044 |
| EZH2 | NCLN | 0.302036122 |
| EZH2 | CCNE2 | 0.301836912 |
| EZH2 | NEURL1B | 0.300184624 |
| EZH2 | UBL3 | 0.298402369 |
| EZH2 | CPD | 0.297594636 |
| EZH2 | FNBP1L | 0.296117955 |
| EZH2 | GNL3L | 0.294747128 |
| EZH2 | TPD52L1 | 0.293672268 |
| EZH2 | NPAT | 0.291090995 |
| EZH2 | PRKACB | 0.285806351 |
| EZH2 | DHFR | 0.279392577 |
| EZH2 | IRF6 | 0.278117684 |
| EZH2 | ABAT | 0.2771354 |
| EZH2 | DYNC1I2 | 0.276887617 |
| EZH2 | CLN6 | 0.275690721 |
| EZH2 | NEK2 | 0.274060736 |
| EZH2 | EXD2 | 0.271180189 |
| EZH2 | SND1 | 0.26847893 |
| EZH2 | LIG1 | 0.268475909 |
| EZH2 | LASP1 | 0.267572271 |
| EZH2 | NDUFV3 | 0.266837792 |
| EZH2 | ICMT | 0.2667271 |
| EZH2 | DLG1 | 0.264143525 |
| EZH2 | TJP1 | 0.264098005 |
| EZH2 | KIF22 | 0.263555585 |
| EZH2 | TMEM134 | 0.263404879 |
| EZH2 | SOCS6 | 0.262838626 |
| EZH2 | BFAR | 0.259768915 |
| EZH2 | ZMYND11 | 0.259456898 |
| EZH2 | FKBP11 | 0.258637727 |
| EZH2 | CHFR | 0.256739954 |
| EZH2 | XPNPEP2 | 0.254641802 |
| EZH2 | DGCR8 | 0.251322984 |
| EZH2 | SPTAN1 | 0.250625203 |
| EZH2 | CNOT6 | 0.247664312 |
| EZH2 | EPB41 | 0.245289615 |
| EZH2 | CKS1B | 0.244018749 |
| EZH2 | KLF16 | 0.243658838 |
| EZH2 | MRAP2 | 0.241647508 |
| EZH2 | DOPEY1 | 0.236668906 |
| EZH2 | TGFB1 | 0.235261253 |
| EZH2 | MTMR10 | 0.233445347 |
| EZH2 | HACL1 | 0.233227076 |
| EZH2 | FUT8 | 0.232865725 |
| EZH2 | MXD3 | 0.232107155 |
| EZH2 | SAE1 | 0.227404078 |
| EZH2 | TMEM65 | 0.225749573 |
| EZH2 | STRN4 | 0.221946489 |
| EZH2 | KIAA1522 | 0.220247757 |
| EZH2 | TMEM68 | 0.216636686 |
| EZH2 | EEA1 | 0.214511645 |
| EZH2 | CENPJ | 0.213099525 |
| EZH2 | SLC12A7 | 0.209958305 |
| EZH2 | CLCN5 | 0.205679246 |
| EZH2 | NXPH4 | 0.205485999 |
| EZH2 | RALGAPA1 | 0.204308915 |
| EZH2 | MYO1E | 0.204189305 |
| EZH2 | MPDU1 | 0.203231595 |
| EZH2 | PDE8A | 0.20314536 |
| EZH2 | TRIM24 | 0.202528925 |
| EZH2 | JARID2 | 0.201584859 |
| EZH2 | SIPA1 | 0.199575688 |
| EZH2 | ERC1 | 0.198809433 |
| EZH2 | CSTF3 | 0.19879129 |
| EZH2 | LRRC42 | 0.198302888 |
| EZH2 | CLDN15 | 0.198070993 |
| EZH2 | RAD18 | 0.196291239 |
| EZH2 | HNRNPH3 | 0.195930557 |
| EZH2 | CPSF1 | 0.19284485 |
| EZH2 | ATIC | 0.192271667 |
| EZH2 | TTLL7 | 0.190023453 |
| EZH2 | CDC27 | 0.189146679 |
| EZH2 | MED1 | 0.184519823 |
| EZH2 | SNRK | 0.18439416 |
| EZH2 | KIAA1549 | 0.183512372 |
| EZH2 | PELO | 0.182535301 |
| EZH2 | DUSP10 | 0.180492714 |
| EZH2 | TINAGL1 | 0.179084515 |
| EZH2 | HPS3 | 0.178007999 |
| EZH2 | ACD | 0.177381519 |
| EZH2 | DGKZ | 0.176587912 |
| EZH2 | KIAA0586 | 0.174462744 |
| EZH2 | EHD4 | 0.171014203 |
| EZH2 | DNM3 | 0.167123813 |
| EZH2 | LPCAT4 | 0.164682692 |
| EZH2 | SAMD5 | 0.164162049 |
| EZH2 | AAAS | 0.163489701 |
| EZH2 | PRPSAP2 | 0.16233443 |
| EZH2 | EHMT2 | 0.162255279 |
| EZH2 | FAM3A | 0.161877858 |
| EZH2 | MRPL48 | 0.160229818 |
| EZH2 | PODXL | 0.159702333 |
| EZH2 | PDHA1 | 0.158269134 |
| EZH2 | RNASEH1 | 0.158229536 |
| EZH2 | PRPSAP1 | 0.157039894 |
| EZH2 | B3GALNT1 | 0.15611217 |
| EZH2 | TLE4 | 0.153117123 |
| EZH2 | ALMS1 | 0.152944785 |
| EZH2 | DOK6 | 0.152805445 |
| EZH2 | ATP13A3 | 0.152234029 |
| EZH2 | MINK1 | 0.151467239 |
| EZH2 | POLR3GL | 0.151148687 |
| EZH2 | TDG | 0.151098582 |
| EZH2 | CENPO | 0.149428296 |
| EZH2 | INHBB | 0.14816325 |
| EZH2 | YEATS4 | 0.144885429 |
| EZH2 | STK38L | 0.142748868 |
| EZH2 | RPS6KA2 | 0.142620251 |
| EZH2 | POLG | 0.141830106 |
| EZH2 | PRRG3 | 0.141190558 |
| EZH2 | TMEM2 | 0.13969236 |
| EZH2 | IREB2 | 0.13941582 |
| EZH2 | PPP1R8 | 0.139117227 |
| EZH2 | B3GALT6 | 0.138645409 |
| EZH2 | HEATR6 | 0.136175488 |
| EZH2 | PPCDC | 0.135999128 |
| EZH2 | YTHDC2 | 0.134940028 |
| EZH2 | LRAT | 0.133359198 |
| EZH2 | MTIF2 | 0.131801652 |
| EZH2 | PROCA1 | 0.127498783 |
| EZH2 | MYOCD | 0.125873258 |
| EZH2 | MCM3 | 0.125088609 |
| EZH2 | TAGAP | 0.123072985 |
| EZH2 | STX16 | 0.122982044 |
| EZH2 | TMEM38B | 0.115974446 |
| EZH2 | NUP35 | 0.114533303 |
| EZH2 | ZNF766 | 0.113462143 |
| EZH2 | CXCR4 | 0.111981127 |
| EZH2 | INO80C | 0.110899254 |
| EZH2 | PLXNB1 | 0.106591535 |
| EZH2 | RCCD1 | 0.106556875 |
| EZH2 | RGL2 | 0.101784755 |
| EZH2 | ANKRD9 | 0.101578823 |
| EZH2 | LMO7 | 0.100706838 |
| EZH2 | TCF7 | 0.099982768 |
| EZH2 | C8orf76 | 0.098568216 |
| EZH2 | MCM2 | 0.098037384 |
| EZH2 | SEMA4A | 0.09783283 |
| EZH2 | RPP14 | 0.096788781 |
| EZH2 | ZNF846 | 0.094039023 |
| EZH2 | MTHFD1L | 0.093778533 |
| EZH2 | DCLK2 | 0.08714824 |
| EZH2 | NFAT5 | 0.086527171 |
| EZH2 | RCC1 | 0.084891503 |
| EZH2 | NPY1R | 0.082925565 |
| EZH2 | N4BP2 | 0.082070241 |
| EZH2 | LNX1 | 0.081229715 |
| EZH2 | MYCN | 0.078972804 |
| EZH2 | HOXA9 | 0.078375521 |
| EZH2 | TOM1L1 | 0.070478973 |
| EZH2 | PCDHB4 | 0.066859238 |
| EZH2 | SPATA7 | 0.066396897 |
| EZH2 | SDHAP1 | 0.061380975 |
| EZH2 | DLEU2 | 0.060221259 |
| EZH2 | LIG3 | 0.057941698 |
| EZH2 | LPAR3 | 0.057667769 |
| EZH2 | TADA2A | 0.056865806 |
| EZH2 | ZNF300 | 0.054959559 |
| EZH2 | ELF4 | 0.052453161 |
| EZH2 | C17orf75 | 0.04965731 |
| EZH2 | OIP5 | 0.04802055 |
| EZH2 | NBEAL2 | 0.04638835 |
| EZH2 | LRP5L | 0.045398807 |
| EZH2 | FAM122B | 0.037164681 |
| EZH2 | GJC1 | 0.036645303 |
| EZH2 | SLC13A2 | 0.034269348 |
| EZH2 | GALNT3 | 0.033010165 |
| EZH2 | PFAS | 0.03013558 |
| EZH2 | USP27X | 0.020250014 |
| EZH2 | ZNF232 | 0.010786773 |
| EZH2 | C8orf37 | 0.008531456 |
| EZH2 | PCIF1 | 0.004245482 |
| EZH2 | C19orf12 | 0.001133844 |
| EZH2 | ANKS1A | 0.000510421 |
| EZH2 | RAB30 | 2.06E-05 |
| EZH2 | SPC24 | 2.71E-06 |
| EZH2 | PGBD4 | 8.79E-11 |
| FLI1 | PSMB4 | 3.824571611 |
| FLI1 | TRIM69 | 3.030640647 |
| FLI1 | IFT46 | 2.98720669 |
| FLI1 | TIMM10 | 2.885245882 |
| FLI1 | TBC1D8B | 2.580530444 |
| FLI1 | EXT1 | 2.5298609 |
| FLI1 | CYLD | 2.519886464 |
| FLI1 | AURKAIP1 | 2.352798824 |
| FLI1 | PSMA1 | 2.284138981 |
| FLI1 | CDC73 | 2.006317158 |
| FLI1 | TBC1D1 | 1.941993141 |
| FLI1 | LDB1 | 1.900057014 |
| FLI1 | ANP32A | 1.874014472 |
| FLI1 | SLC29A1 | 1.851104181 |
| FLI1 | ABHD5 | 1.640098163 |
| FLI1 | USE1 | 1.621655691 |
| FLI1 | PICALM | 1.605781704 |
| FLI1 | SCAND1 | 1.56595556 |
| FLI1 | GTF2A2 | 1.54240801 |
| FLI1 | RIOK3 | 1.358770436 |
| FLI1 | HIGD2A | 1.271870221 |
| FLI1 | ACTR1B | 1.216725852 |
| FLI1 | TMEM154 | 1.202248342 |
| FLI1 | MARK3 | 1.186714766 |
| FLI1 | XRCC5 | 1.18006273 |
| FLI1 | VCAM1 | 1.159768636 |
| FLI1 | PCGF6 | 1.155085807 |
| FLI1 | EMID1 | 1.089642259 |
| FLI1 | TBXA2R | 1.085899681 |
| FLI1 | ARGLU1 | 1.085183556 |
| FLI1 | DCK | 1.075007924 |
| FLI1 | PHKG1 | 1.030851855 |
| FLI1 | CRABP1 | 1.027704196 |
| FLI1 | PLXDC1 | 1.026143786 |
| FLI1 | OMA1 | 1.020121815 |
| FLI1 | FLI1 | 1 |
| FLI1 | GNAI3 | 0.988794983 |
| FLI1 | MTIF3 | 0.983601992 |
| FLI1 | FNBP4 | 0.971033875 |
| FLI1 | PTS | 0.950996146 |
| FLI1 | DPAGT1 | 0.899310166 |
| FLI1 | BANP | 0.89827203 |
| FLI1 | P4HB | 0.893967571 |
| FLI1 | RCAN3 | 0.880260018 |
| FLI1 | TMEM2 | 0.860391395 |
| FLI1 | VAPA | 0.842635358 |
| FLI1 | TTC1 | 0.823855176 |
| FLI1 | BHLHE41 | 0.804881136 |
| FLI1 | HDAC2 | 0.794019228 |
| FLI1 | KIAA0040 | 0.782791933 |
| FLI1 | ATG2A | 0.777568459 |
| FLI1 | VPS4B | 0.777335307 |
| FLI1 | MRPL40 | 0.745056209 |
| FLI1 | NR2C2 | 0.736621555 |
| FLI1 | ITFG1 | 0.735746303 |
| FLI1 | RAD50 | 0.690091477 |
| FLI1 | GPX7 | 0.689628914 |
| FLI1 | NLRX1 | 0.666639287 |
| FLI1 | NFE2L2 | 0.64308973 |
| FLI1 | ITGB5 | 0.586636459 |
| FLI1 | TAX1BP3 | 0.586418873 |
| FLI1 | LGALS8 | 0.585560828 |
| FLI1 | TSNAX | 0.580862363 |
| FLI1 | ITGB1 | 0.552129397 |
| FLI1 | ZNF513 | 0.543015098 |
| FLI1 | ACAA1 | 0.526088063 |
| FLI1 | CD74 | 0.507132606 |
| FLI1 | UFC1 | 0.504623751 |
| FLI1 | INTS6 | 0.503469144 |
| FLI1 | ETV7 | 0.502792264 |
| FLI1 | ARL2 | 0.483426576 |
| FLI1 | TRIM37 | 0.468629631 |
| FLI1 | TPBG | 0.460878819 |
| FLI1 | WNT11 | 0.445457003 |
| FLI1 | CAPZA2 | 0.432829286 |
| FLI1 | ZNF765 | 0.399226979 |
| FLI1 | GRAMD1B | 0.387022719 |
| FLI1 | ADAMTS10 | 0.3563598 |
| FLI1 | MAP4K3 | 0.346584489 |
| FLI1 | HERC4 | 0.329580886 |
| FLI1 | MAP4 | 0.319089588 |
| FLI1 | MFF | 0.317495037 |
| FLI1 | ZNF558 | 0.316768698 |
| FLI1 | CASP2 | 0.316190243 |
| FLI1 | RERE | 0.315226973 |
| FLI1 | CRKL | 0.315164927 |
| FLI1 | HIST1H4H | 0.314084571 |
| FLI1 | MTF2 | 0.301915304 |
| FLI1 | TUBE1 | 0.282730072 |
| FLI1 | TGFBRAP1 | 0.281769569 |
| FLI1 | RPF2 | 0.265682276 |
| FLI1 | TIPRL | 0.262878953 |
| FLI1 | DERL2 | 0.262791176 |
| FLI1 | MBOAT2 | 0.252954635 |
| FLI1 | AGFG2 | 0.250711558 |
| FLI1 | DCAF4 | 0.250057161 |
| FLI1 | ATXN10 | 0.246261832 |
| FLI1 | CITED4 | 0.241694035 |
| FLI1 | LCORL | 0.231636152 |
| FLI1 | CSNK1D | 0.216656578 |
| FLI1 | C16orf91 | 0.215465732 |
| FLI1 | RUVBL1 | 0.201990033 |
| FLI1 | NDUFA4L2 | 0.201187421 |
| FLI1 | POLDIP2 | 0.199008027 |
| FLI1 | PEX2 | 0.198230241 |
| FLI1 | POP4 | 0.184562853 |
| FLI1 | HOXC6 | 0.184429047 |
| FLI1 | WDR70 | 0.181420406 |
| FLI1 | KEAP1 | 0.176340844 |
| FLI1 | ALDH16A1 | 0.146010556 |
| FLI1 | MAPK11 | 0.142907156 |
| FLI1 | RHOT2 | 0.132923303 |
| FLI1 | RHOD | 0.129331196 |
| FLI1 | CCDC69 | 0.128048647 |
| FLI1 | KLHL8 | 0.107446835 |
| FLI1 | CLIP3 | 0.097607778 |
| FLI1 | DECR2 | 0.085715229 |
| FLI1 | CDH2 | 0.023056684 |
| FOS | DUSP1 | 71.05243334 |
| FOS | JUN | 51.51708018 |
| FOS | ZFP36 | 49.1183451 |
| FOS | DNAJB1 | 49.01529213 |
| FOS | IER2 | 48.59063379 |
| FOS | PPP1R15A | 47.89884656 |
| FOS | HSPA1A | 47.0485245 |
| FOS | GADD45B | 39.27830833 |
| FOS | JUNB | 36.43209537 |
| FOS | ATF3 | 35.96814857 |
| FOS | CYR61 | 34.55637117 |
| FOS | FOSB | 33.77733976 |
| FOS | EGR1 | 32.53673498 |
| FOS | SOCS3 | 29.4923037 |
| FOS | HES1 | 28.35658014 |
| FOS | HSP90AA1 | 27.11226754 |
| FOS | NR4A1 | 26.68951096 |
| FOS | IER3 | 23.19076416 |
| FOS | HSPA8 | 22.76793961 |
| FOS | JUND | 19.71643886 |
| FOS | ID3 | 18.52479511 |
| FOS | DDIT4 | 17.44462584 |
| FOS | RGS16 | 16.77529874 |
| FOS | MYC | 15.35795943 |
| FOS | H3F3B | 14.84837841 |
| FOS | ZFP36L1 | 13.95973476 |
| FOS | ID1 | 12.91610279 |
| FOS | KLF10 | 11.58644043 |
| FOS | TSC22D1 | 11.53091098 |
| FOS | PHLDA1 | 10.46329805 |
| FOS | UBB | 9.778429752 |
| FOS | ADM | 9.553216919 |
| FOS | CITED2 | 9.505578527 |
| FOS | KLF4 | 8.696263605 |
| FOS | EGR2 | 8.049905465 |
| FOS | ADAMTS1 | 7.746042606 |
| FOS | HSPB1 | 7.693617482 |
| FOS | GEM | 7.612477646 |
| FOS | BHLHE40 | 7.555507092 |
| FOS | C11orf96 | 7.541333957 |
| FOS | ID2 | 7.510292186 |
| FOS | SNAI1 | 7.288252866 |
| FOS | BRD2 | 7.128353993 |
| FOS | CDKN1A | 6.706029174 |
| FOS | EIF1 | 6.616501187 |
| FOS | CTGF | 6.392777647 |
| FOS | ZFP36L2 | 6.250425952 |
| FOS | ID4 | 6.112282707 |
| FOS | CEBPB | 5.895024263 |
| FOS | NFIL3 | 5.66822685 |
| FOS | SERTAD1 | 5.551569324 |
| FOS | IRF1 | 5.393587698 |
| FOS | CSRNP1 | 5.247269176 |
| FOS | PLK2 | 5.15489698 |
| FOS | IER5L | 4.810906652 |
| FOS | GADD45A | 4.265696445 |
| FOS | HSPD1 | 4.188369319 |
| FOS | ZEB2 | 4.042137903 |
| FOS | BAMBI | 3.968678435 |
| FOS | SAT1 | 3.916742786 |
| FOS | MFAP5 | 3.910800818 |
| FOS | IER5 | 3.83632788 |
| FOS | KLF6 | 3.697310364 |
| FOS | BTG1 | 3.627895043 |
| FOS | CRYAB | 3.61460087 |
| FOS | RRBP1 | 3.570760978 |
| FOS | NKD1 | 3.544220947 |
| FOS | HEXIM1 | 3.349451436 |
| FOS | RRAD | 3.296178713 |
| FOS | RHOB | 3.25394241 |
| FOS | H2AFZ | 3.248659876 |
| FOS | ARRDC3 | 3.178019769 |
| FOS | SOCS1 | 3.01367384 |
| FOS | RND3 | 2.991476162 |
| FOS | HSP90AB1 | 2.952809885 |
| FOS | MALAT1 | 2.927231877 |
| FOS | PPP1R15B | 2.910611048 |
| FOS | DST | 2.865409866 |
| FOS | NOTCH4 | 2.760996737 |
| FOS | CXCL1 | 2.717810329 |
| FOS | TTYH2 | 2.640841275 |
| FOS | TUBB2A | 2.6307344 |
| FOS | PJA2 | 2.558407073 |
| FOS | EMP1 | 2.530901852 |
| FOS | GAK | 2.526375255 |
| FOS | AHNAK | 2.47809129 |
| FOS | DNAJB4 | 2.449151892 |
| FOS | PLCL1 | 2.357495161 |
| FOS | MYOCD | 2.336222609 |
| FOS | PPTC7 | 2.32417769 |
| FOS | SOX4 | 2.295425104 |
| FOS | LENG8 | 2.243803126 |
| FOS | SRSF7 | 2.216486318 |
| FOS | RGS2 | 2.110823238 |
| FOS | COL13A1 | 2.091955481 |
| FOS | EEF1A1 | 2.037574571 |
| FOS | FERMT3 | 2.009894793 |
| FOS | CXCL3 | 2.004578004 |
| FOS | GNAS | 1.99948676 |
| FOS | FLCN | 1.995003582 |
| FOS | SNPH | 1.991795427 |
| FOS | TNFAIP3 | 1.981156519 |
| FOS | UBE2D3 | 1.979920298 |
| FOS | DDIT3 | 1.953096248 |
| FOS | FRAS1 | 1.951157603 |
| FOS | CHMP6 | 1.934105371 |
| FOS | CDC26 | 1.906332433 |
| FOS | PIM1 | 1.871013314 |
| FOS | BEND3 | 1.863449258 |
| FOS | H1F0 | 1.863209633 |
| FOS | PPDPF | 1.860458961 |
| FOS | SLC30A9 | 1.82427664 |
| FOS | ARFIP2 | 1.772491401 |
| FOS | FAM53B | 1.744622887 |
| FOS | CD55 | 1.7427922 |
| FOS | APOE | 1.737843103 |
| FOS | HSPH1 | 1.729887805 |
| FOS | NFKBIZ | 1.721854775 |
| FOS | MECOM | 1.712166671 |
| FOS | NEAT1 | 1.665774544 |
| FOS | STK3 | 1.657553821 |
| FOS | FAM19A5 | 1.630631078 |
| FOS | ARPC2 | 1.619284327 |
| FOS | PNRC1 | 1.604461168 |
| FOS | MEG3 | 1.583134861 |
| FOS | RNF2 | 1.548884201 |
| FOS | LAMB1 | 1.520817149 |
| FOS | RPL13A | 1.518983586 |
| FOS | PITPNM2 | 1.492998285 |
| FOS | NOC4L | 1.485834349 |
| FOS | CCNG2 | 1.485444991 |
| FOS | UBC | 1.483370449 |
| FOS | SQSTM1 | 1.457998763 |
| FOS | C11orf65 | 1.455449597 |
| FOS | CYGB | 1.452673997 |
| FOS | ZBED5 | 1.433266463 |
| FOS | CENPW | 1.413292317 |
| FOS | CCDC130 | 1.40404435 |
| FOS | TSC22D2 | 1.399203293 |
| FOS | TTC9C | 1.396433734 |
| FOS | MYL12B | 1.386448231 |
| FOS | CCNL1 | 1.381057334 |
| FOS | SORCS1 | 1.377444901 |
| FOS | CCAR1 | 1.370818951 |
| FOS | IGFBP6 | 1.368893034 |
| FOS | ST3GAL2 | 1.356419795 |
| FOS | SLC38A2 | 1.347397495 |
| FOS | PUM1 | 1.338425054 |
| FOS | UBA52 | 1.323498031 |
| FOS | TRIM39 | 1.299853256 |
| FOS | TRPS1 | 1.261211141 |
| FOS | DNAJA1 | 1.257906639 |
| FOS | OCIAD1 | 1.249283495 |
| FOS | AKAP12 | 1.243979864 |
| FOS | LOX | 1.232047634 |
| FOS | LTBP1 | 1.229873083 |
| FOS | MIR155HG | 1.228255231 |
| FOS | DAB2 | 1.209774602 |
| FOS | KPNB1 | 1.20212028 |
| FOS | VCAM1 | 1.193834269 |
| FOS | STK10 | 1.192837313 |
| FOS | PSMB1 | 1.188407637 |
| FOS | BAG3 | 1.176383169 |
| FOS | SYT8 | 1.167446574 |
| FOS | RFTN2 | 1.1666743 |
| FOS | RHOC | 1.138323498 |
| FOS | STAT3 | 1.117763205 |
| FOS | KCTD1 | 1.117672215 |
| FOS | SGPP2 | 1.109949244 |
| FOS | HAS2 | 1.097994295 |
| FOS | LGALS7 | 1.097340505 |
| FOS | SCN5A | 1.089085164 |
| FOS | SPRR1B | 1.081972354 |
| FOS | KCTD17 | 1.066576693 |
| FOS | KIFC1 | 1.062962191 |
| FOS | ALS2CL | 1.061415726 |
| FOS | UBE2S | 1.055727098 |
| FOS | ARHGAP11A | 1.012300777 |
| FOS | FGF7 | 1.003357613 |
| FOS | FOS | 1 |
| FOS | GTF2H1 | 0.992716905 |
| FOS | CACNA1G | 0.961200243 |
| FOS | LBH | 0.958510654 |
| FOS | ARL4D | 0.957583236 |
| FOS | DNMT3A | 0.951576879 |
| FOS | TRAPPC6B | 0.944484176 |
| FOS | CSNK1D | 0.942092648 |
| FOS | CALR | 0.937329671 |
| FOS | ADAM23 | 0.926350227 |
| FOS | LAMP2 | 0.910936239 |
| FOS | SLC4A7 | 0.90563412 |
| FOS | DLL4 | 0.901821841 |
| FOS | SOX2 | 0.8985046 |
| FOS | UAP1L1 | 0.887971627 |
| FOS | TUBGCP5 | 0.876282865 |
| FOS | COX4I1 | 0.873569019 |
| FOS | VEGFA | 0.865513509 |
| FOS | PMAIP1 | 0.865264308 |
| FOS | KRT14 | 0.865015458 |
| FOS | KRTDAP | 0.854274524 |
| FOS | CALD1 | 0.851966213 |
| FOS | ELL | 0.84622444 |
| FOS | KLF9 | 0.837253152 |
| FOS | ARL6IP5 | 0.832870361 |
| FOS | PRNP | 0.82532968 |
| FOS | PRKAG2 | 0.818435566 |
| FOS | COQ9 | 0.817789912 |
| FOS | ZNF8 | 0.817025607 |
| FOS | CTSB | 0.81088566 |
| FOS | TRIM47 | 0.809727497 |
| FOS | CSTA | 0.80972584 |
| FOS | LAMC1 | 0.80911291 |
| FOS | CYBRD1 | 0.805874351 |
| FOS | OGFR | 0.800925628 |
| FOS | LYSMD4 | 0.799181938 |
| FOS | CDH1 | 0.794521731 |
| FOS | CYB5B | 0.790560561 |
| FOS | ATOH8 | 0.787378058 |
| FOS | IRF2BP1 | 0.779497985 |
| FOS | PDK4 | 0.772724045 |
| FOS | ZMYND8 | 0.770060813 |
| FOS | CSGALNACT1 | 0.763072443 |
| FOS | SERTAD3 | 0.75917186 |
| FOS | NUDC | 0.756941932 |
| FOS | CASC3 | 0.756316205 |
| FOS | MACROD2 | 0.737323028 |
| FOS | TTC39A | 0.730997225 |
| FOS | ZNF423 | 0.724858911 |
| FOS | PANK1 | 0.724602953 |
| FOS | KRT17 | 0.713868115 |
| FOS | NUDCD2 | 0.710040201 |
| FOS | TWISTNB | 0.709353887 |
| FOS | SND1 | 0.706530678 |
| FOS | LRRC4C | 0.703535724 |
| FOS | NR4A2 | 0.6990051 |
| FOS | WWC2 | 0.698560847 |
| FOS | COPZ2 | 0.692255343 |
| FOS | TRIM68 | 0.685704852 |
| FOS | SBSN | 0.680991935 |
| FOS | SLC12A6 | 0.679448319 |
| FOS | MTMR12 | 0.677059939 |
| FOS | ATF4 | 0.666161417 |
| FOS | SH3KBP1 | 0.662932779 |
| FOS | ZFAND5 | 0.661933306 |
| FOS | LGALS7B | 0.656775998 |
| FOS | STRN3 | 0.656261894 |
| FOS | ARHGEF38 | 0.655874152 |
| FOS | EVI5 | 0.646424433 |
| FOS | HSPB6 | 0.646222315 |
| FOS | DPP3 | 0.640674134 |
| FOS | LAMA5 | 0.632595247 |
| FOS | SCG2 | 0.631232078 |
| FOS | C10orf55 | 0.629471012 |
| FOS | AURKA | 0.626858825 |
| FOS | ZNF608 | 0.626057497 |
| FOS | IGFBP2 | 0.624439052 |
| FOS | ANO4 | 0.623708246 |
| FOS | FDPS | 0.622380836 |
| FOS | PERP | 0.620677998 |
| FOS | SHC3 | 0.617426791 |
| FOS | ERLIN2 | 0.614786119 |
| FOS | SHANK3 | 0.612130624 |
| FOS | SERPINE1 | 0.611830897 |
| FOS | CDCA3 | 0.609408079 |
| FOS | LAPTM5 | 0.60803735 |
| FOS | UBE4A | 0.607910817 |
| FOS | HERPUD1 | 0.607022231 |
| FOS | RPS6KA3 | 0.60621774 |
| FOS | DMKN | 0.604653705 |
| FOS | PRKAB2 | 0.604264727 |
| FOS | MYL5 | 0.602149139 |
| FOS | AFAP1L2 | 0.60015538 |
| FOS | ANGPTL2 | 0.597121985 |
| FOS | FZD2 | 0.592680706 |
| FOS | CCDC89 | 0.589650637 |
| FOS | BDNF | 0.587634671 |
| FOS | ARHGAP19 | 0.58619506 |
| FOS | PPP1R10 | 0.585118849 |
| FOS | FBXO11 | 0.583782342 |
| FOS | TCF12 | 0.583099465 |
| FOS | PACSIN3 | 0.582710399 |
| FOS | COL4A1 | 0.582606981 |
| FOS | C15orf41 | 0.581031908 |
| FOS | ZNF706 | 0.580135603 |
| FOS | PBK | 0.57871172 |
| FOS | NFIC | 0.577926106 |
| FOS | LY6D | 0.57531738 |
| FOS | SERPINE2 | 0.574384652 |
| FOS | BUD31 | 0.574039365 |
| FOS | ANKRD49 | 0.573230266 |
| FOS | ABCA8 | 0.570965289 |
| FOS | OAF | 0.567040769 |
| FOS | NCOA7 | 0.560841916 |
| FOS | CELSR1 | 0.554550093 |
| FOS | FST | 0.554198231 |
| FOS | ZNF711 | 0.55282414 |
| FOS | MRC2 | 0.550451944 |
| FOS | ANXA6 | 0.547294525 |
| FOS | ANXA1 | 0.542187813 |
| FOS | SGSM3 | 0.540253704 |
| FOS | IRF6 | 0.539004457 |
| FOS | SOD1 | 0.538832552 |
| FOS | RTN4 | 0.533738928 |
| FOS | MBD6 | 0.532326085 |
| FOS | RPL9 | 0.529868224 |
| FOS | PPHLN1 | 0.529501629 |
| FOS | CNKSR3 | 0.528830182 |
| FOS | GPNMB | 0.52080861 |
| FOS | HLA-F | 0.518038912 |
| FOS | SEMA4C | 0.517679808 |
| FOS | GPAM | 0.516180341 |
| FOS | ABCC3 | 0.515251093 |
| FOS | LSP1 | 0.513852483 |
| FOS | RPL21 | 0.510625138 |
| FOS | C6 | 0.50992418 |
| FOS | CDK14 | 0.507275645 |
| FOS | G3BP1 | 0.505102489 |
| FOS | RBBP6 | 0.503654899 |
| FOS | WFDC2 | 0.503316688 |
| FOS | ADD3 | 0.501022977 |
| FOS | PLEKHA4 | 0.497964997 |
| FOS | CAPN2 | 0.497496055 |
| FOS | MYEF2 | 0.496670679 |
| FOS | DCTN1 | 0.495618081 |
| FOS | HNRNPD | 0.493134507 |
| FOS | SLC41A1 | 0.490883182 |
| FOS | SF3A1 | 0.489554967 |
| FOS | FABP5 | 0.488864848 |
| FOS | TIMP3 | 0.485808517 |
| FOS | EIF5 | 0.484906882 |
| FOS | TNXB | 0.484013746 |
| FOS | PKIA | 0.48385903 |
| FOS | APP | 0.48355922 |
| FOS | TSPO | 0.483145082 |
| FOS | CDNF | 0.481539278 |
| FOS | RPL13 | 0.478707321 |
| FOS | RPAP1 | 0.477939752 |
| FOS | LPAR3 | 0.475402093 |
| FOS | GPX4 | 0.474541709 |
| FOS | YPEL1 | 0.47233384 |
| FOS | RNF157 | 0.471969768 |
| FOS | PTGER4 | 0.471728932 |
| FOS | HNRNPC | 0.469052599 |
| FOS | AFF2 | 0.468108108 |
| FOS | YTHDC2 | 0.464003741 |
| FOS | SYN1 | 0.46006768 |
| FOS | SULF2 | 0.459707203 |
| FOS | NPTXR | 0.45865658 |
| FOS | RAB40C | 0.454097117 |
| FOS | SPATA17 | 0.4523411 |
| FOS | RPS29 | 0.452184232 |
| FOS | TSC22D3 | 0.44945234 |
| FOS | EDN2 | 0.449212601 |
| FOS | PDLIM2 | 0.443979062 |
| FOS | RCC1 | 0.443953878 |
| FOS | UBN2 | 0.443544757 |
| FOS | STARD8 | 0.441330026 |
| FOS | TXN | 0.44113891 |
| FOS | NRP1 | 0.440714921 |
| FOS | CYP4F12 | 0.440636907 |
| FOS | RPL38 | 0.439641363 |
| FOS | RSPO1 | 0.43963503 |
| FOS | POLG2 | 0.4393323 |
| FOS | CIRBP | 0.43852452 |
| FOS | PCBP3 | 0.436102535 |
| FOS | IDH3G | 0.434185626 |
| FOS | BOC | 0.42640377 |
| FOS | GLS | 0.42635048 |
| FOS | CCDC146 | 0.424951598 |
| FOS | USP21 | 0.420477453 |
| FOS | AKIRIN2 | 0.410286426 |
| FOS | AEN | 0.408036828 |
| FOS | TYRO3 | 0.406745552 |
| FOS | HECW2 | 0.406217639 |
| FOS | DIRAS1 | 0.405149053 |
| FOS | ROGDI | 0.40238433 |
| FOS | C12orf65 | 0.399404663 |
| FOS | TOM1L2 | 0.392998048 |
| FOS | ELAC2 | 0.392334802 |
| FOS | CBX8 | 0.388454567 |
| FOS | NUS1 | 0.387359903 |
| FOS | ANKRD16 | 0.383203216 |
| FOS | EMX2 | 0.382113427 |
| FOS | SMAD3 | 0.377049139 |
| FOS | C17orf53 | 0.373428364 |
| FOS | RNF26 | 0.370421602 |
| FOS | SLC6A8 | 0.369401208 |
| FOS | TLE3 | 0.365185832 |
| FOS | CHST6 | 0.364377716 |
| FOS | TCEANC | 0.363877484 |
| FOS | AXIN1 | 0.361877345 |
| FOS | SKA2 | 0.360831197 |
| FOS | TMEM45B | 0.360296621 |
| FOS | KLHL21 | 0.358735857 |
| FOS | C8orf44 | 0.354409523 |
| FOS | NOTCH3 | 0.351431789 |
| FOS | GPR176 | 0.349071032 |
| FOS | LASP1 | 0.348412349 |
| FOS | ALDH1A3 | 0.347491561 |
| FOS | PLEKHM1 | 0.346167676 |
| FOS | TLE2 | 0.343267211 |
| FOS | DNM2 | 0.341421853 |
| FOS | TSPYL2 | 0.338283215 |
| FOS | NUMB | 0.337662991 |
| FOS | SYDE1 | 0.337422181 |
| FOS | MAP3K14 | 0.336733656 |
| FOS | ATF7IP | 0.3333462 |
| FOS | SRF | 0.332954502 |
| FOS | IMMT | 0.331055053 |
| FOS | BCL9 | 0.330496162 |
| FOS | DHODH | 0.329702219 |
| FOS | PMS2P1 | 0.323542987 |
| FOS | SAMD13 | 0.321464555 |
| FOS | GGPS1 | 0.317765963 |
| FOS | CLEC16A | 0.317662412 |
| FOS | ZFYVE9 | 0.313864553 |
| FOS | CDH23 | 0.312554588 |
| FOS | TESK2 | 0.311926058 |
| FOS | AP4M1 | 0.31137949 |
| FOS | AMBRA1 | 0.309097081 |
| FOS | FAM86DP | 0.307583277 |
| FOS | MMP9 | 0.30627613 |
| FOS | SP2 | 0.303143169 |
| FOS | STK11 | 0.30208418 |
| FOS | IQCB1 | 0.301821145 |
| FOS | PHKG2 | 0.301207278 |
| FOS | CCNYL1 | 0.300175625 |
| FOS | ARMC1 | 0.299841611 |
| FOS | GNE | 0.295283128 |
| FOS | PMM1 | 0.29325114 |
| FOS | SH2B3 | 0.288529831 |
| FOS | PABPN1 | 0.288198484 |
| FOS | TMEM53 | 0.28790215 |
| FOS | ITGA6 | 0.2876352 |
| FOS | GPC1 | 0.287068054 |
| FOS | CTNNAL1 | 0.285960309 |
| FOS | TEF | 0.285806475 |
| FOS | TUT1 | 0.283011888 |
| FOS | CADM4 | 0.280909833 |
| FOS | PLCD1 | 0.278373243 |
| FOS | BAHD1 | 0.277290203 |
| FOS | PNN | 0.276885972 |
| FOS | DYNC1H1 | 0.27676729 |
| FOS | GAL | 0.27641501 |
| FOS | PAIP2B | 0.273451407 |
| FOS | BCL11B | 0.271513724 |
| FOS | LPIN1 | 0.270337044 |
| FOS | GOLPH3L | 0.269921695 |
| FOS | DOCK1 | 0.269858988 |
| FOS | SLC25A28 | 0.269289426 |
| FOS | GSTO2 | 0.269088862 |
| FOS | GTF3C5 | 0.268202191 |
| FOS | ARNTL2 | 0.266642951 |
| FOS | PKP3 | 0.26533661 |
| FOS | FAM43A | 0.264275155 |
| FOS | BCAT2 | 0.263896966 |
| FOS | FXYD6 | 0.263649277 |
| FOS | PILRB | 0.263382694 |
| FOS | HNRNPUL1 | 0.261660078 |
| FOS | FHIT | 0.257936897 |
| FOS | PPP2R5D | 0.257371629 |
| FOS | WTIP | 0.256251881 |
| FOS | ZNF385A | 0.254907886 |
| FOS | FAM46C | 0.254417431 |
| FOS | PRKCD | 0.254270154 |
| FOS | PLK3 | 0.253374274 |
| FOS | SOCS5 | 0.253075753 |
| FOS | HCP5 | 0.251198047 |
| FOS | ELMO1 | 0.250546544 |
| FOS | CETN3 | 0.250307899 |
| FOS | ZBTB7A | 0.249299966 |
| FOS | LPP | 0.249208631 |
| FOS | LDB1 | 0.248732097 |
| FOS | ALDH1A2 | 0.247530981 |
| FOS | USP16 | 0.24670035 |
| FOS | PPP3CA | 0.245500611 |
| FOS | GTF3C3 | 0.244699961 |
| FOS | PSAT1 | 0.244218243 |
| FOS | TTC3P1 | 0.240199862 |
| FOS | ANAPC10 | 0.240191176 |
| FOS | SSBP3 | 0.24014096 |
| FOS | CCDC140 | 0.23851212 |
| FOS | ELK1 | 0.236161691 |
| FOS | STAG3L3 | 0.236061574 |
| FOS | LMO2 | 0.233772615 |
| FOS | CCDC151 | 0.233467519 |
| FOS | RPS6KA2 | 0.232768273 |
| FOS | ZCCHC14 | 0.232459073 |
| FOS | LNPEP | 0.230478269 |
| FOS | KLF11 | 0.23043543 |
| FOS | PPP2R5C | 0.230261335 |
| FOS | OSBPL6 | 0.230055311 |
| FOS | BCL9L | 0.229919452 |
| FOS | CD109 | 0.22979782 |
| FOS | KCTD6 | 0.227443026 |
| FOS | MN1 | 0.226769975 |
| FOS | NDRG4 | 0.226558187 |
| FOS | DDX6 | 0.225464817 |
| FOS | HIST1H2BG | 0.223965686 |
| FOS | TOB2 | 0.22376021 |
| FOS | DHCR24 | 0.223419577 |
| FOS | FAR1 | 0.223300857 |
| FOS | KDM4A | 0.221562529 |
| FOS | MSL3 | 0.22147317 |
| FOS | F12 | 0.220607539 |
| FOS | FCHSD1 | 0.220431435 |
| FOS | RYK | 0.220341029 |
| FOS | NCDN | 0.217945628 |
| FOS | CTNND1 | 0.21783218 |
| FOS | DHX34 | 0.217227096 |
| FOS | CDS2 | 0.216576011 |
| FOS | OGT | 0.216226637 |
| FOS | MRPS30 | 0.21621331 |
| FOS | RAP2A | 0.214874051 |
| FOS | SERPINI1 | 0.214229849 |
| FOS | ETS1 | 0.213266289 |
| FOS | RAP1GAP | 0.212500874 |
| FOS | STK38L | 0.210238638 |
| FOS | WDR54 | 0.208348906 |
| FOS | MRGPRF | 0.207707138 |
| FOS | RBM4 | 0.206706383 |
| FOS | VPS4B | 0.206276843 |
| FOS | LDLR | 0.205986012 |
| FOS | ERCC8 | 0.205699251 |
| FOS | MR1 | 0.205355953 |
| FOS | RCL1 | 0.204917028 |
| FOS | CYP2R1 | 0.204417054 |
| FOS | YPEL4 | 0.202645535 |
| FOS | UBE2F | 0.202510605 |
| FOS | RBM6 | 0.202379234 |
| FOS | ZNF516 | 0.202329637 |
| FOS | LRRC20 | 0.201610533 |
| FOS | DLEU1 | 0.200406993 |
| FOS | AKAP1 | 0.198741133 |
| FOS | PRR12 | 0.197333512 |
| FOS | DNAJC4 | 0.196510978 |
| FOS | REC8 | 0.195324422 |
| FOS | HMBS | 0.194859799 |
| FOS | PNPO | 0.194172346 |
| FOS | SLC30A1 | 0.192246504 |
| FOS | ELP4 | 0.191100703 |
| FOS | SPTLC2 | 0.190567382 |
| FOS | PDE1B | 0.189360654 |
| FOS | NPW | 0.189076398 |
| FOS | CKB | 0.188591027 |
| FOS | AP2A1 | 0.186929344 |
| FOS | TINAGL1 | 0.186521306 |
| FOS | PLEKHA5 | 0.18560993 |
| FOS | MMP28 | 0.184893641 |
| FOS | EHD4 | 0.184607326 |
| FOS | PPIP5K1 | 0.184304053 |
| FOS | MAPK6 | 0.18410191 |
| FOS | KDM4B | 0.181898103 |
| FOS | CCDC120 | 0.181692855 |
| FOS | DBP | 0.180348891 |
| FOS | USP46 | 0.177455128 |
| FOS | ASB1 | 0.176043246 |
| FOS | STARD3NL | 0.175712459 |
| FOS | SP1 | 0.174999759 |
| FOS | RAB6A | 0.174300909 |
| FOS | ANKRD39 | 0.172984156 |
| FOS | HOOK2 | 0.172130325 |
| FOS | WNT16 | 0.170912456 |
| FOS | ETV1 | 0.169343245 |
| FOS | LMOD1 | 0.169324127 |
| FOS | ZNF827 | 0.16906748 |
| FOS | E4F1 | 0.167997217 |
| FOS | SNHG10 | 0.167359885 |
| FOS | PLEC | 0.167330803 |
| FOS | CDH6 | 0.165957619 |
| FOS | DCTN4 | 0.165753788 |
| FOS | DMPK | 0.165238609 |
| FOS | ARL15 | 0.164045994 |
| FOS | HIST1H2BB | 0.163771919 |
| FOS | GJB3 | 0.163455546 |
| FOS | ABCD1 | 0.161331869 |
| FOS | TUG1 | 0.160879814 |
| FOS | CNOT1 | 0.160425637 |
| FOS | WNT3 | 0.160422497 |
| FOS | ATP1B1 | 0.159748207 |
| FOS | EFNA3 | 0.158211581 |
| FOS | SMPDL3A | 0.156435643 |
| FOS | CHN1 | 0.155873072 |
| FOS | MPND | 0.153548704 |
| FOS | FXR2 | 0.15339093 |
| FOS | VAV3 | 0.153070818 |
| FOS | DOC2B | 0.151714301 |
| FOS | JUP | 0.150892596 |
| FOS | KLHL20 | 0.148215578 |
| FOS | AP3M2 | 0.146010534 |
| FOS | SBNO2 | 0.145992871 |
| FOS | SDSL | 0.143115735 |
| FOS | TYW1 | 0.139631071 |
| FOS | BRPF3 | 0.138933627 |
| FOS | MAP3K8 | 0.138086268 |
| FOS | SLC19A2 | 0.135751335 |
| FOS | WDR81 | 0.135514552 |
| FOS | GSK3B | 0.134193188 |
| FOS | CCDC102A | 0.133732833 |
| FOS | RARA | 0.131728335 |
| FOS | ZBTB38 | 0.130820175 |
| FOS | CNTN1 | 0.124725353 |
| FOS | STAG3L2 | 0.12212194 |
| FOS | PRSS22 | 0.121343178 |
| FOS | PWWP2A | 0.117732442 |
| FOS | SNX33 | 0.11744691 |
| FOS | SNRNP40 | 0.116911501 |
| FOS | DLG4 | 0.115027841 |
| FOS | HOXC6 | 0.110141222 |
| FOS | CCDC57 | 0.109036258 |
| FOS | PHYHIP | 0.104759131 |
| FOS | TUFT1 | 0.101455781 |
| FOS | SF3B4 | 0.100518665 |
| FOS | WFS1 | 0.095328193 |
| FOS | FDXR | 0.086780075 |
| FOS | PLOD2 | 0.081903905 |
| FOS | PRUNE2 | 0.070013866 |
| FOS | PAN2 | 0.05367348 |
| FOS | SLC25A29 | 0.053020535 |
| FOS | PDE2A | 0.045376542 |
| FOS | LMCD1 | 0.035475532 |
| FOS | SIN3A | 0.027260283 |
| FOS | PLAGL1 | 0.022463113 |
| FOS | ARAF | 0.022130922 |
| FOS | CLDN1 | 0.014974713 |
| FOS | CSE1L | 0.007675903 |
| FOS | MCM7 | 0.006736461 |
| FOS | GOLT1B | 0.006713862 |
| FOS | PIGS | 0.005664208 |
| FOS | GLA | 0.005507628 |
| FOS | CELF1 | 0.004379727 |
| FOS | XPO1 | 0.004022381 |
| FOS | KDELC2 | 0.001130276 |
| FOS | CPXM2 | 0.000935791 |
| FOS | ASAP2 | 0.000931561 |
| FOS | N4BP1 | 0.000553196 |
| FOS | ZFP91 | 0.000406542 |
| FOS | LAD1 | 0.000179543 |
| FOS | RHOQ | 0.000139128 |
| FOS | HSPA1L | 3.15E-05 |
| FOS | PSMD11 | 1.28E-05 |
| FOS | SMAD7 | 6.34E-06 |
| FOS | HHEX | 6.86E-08 |
| FOS | ZNF775 | 5.80E-08 |
| FOS | GIT1 | 4.95E-08 |
| FOS | CEP68 | 6.77E-09 |
| FOS | ZNF727 | 3.82E-09 |
| FOS | ZNF253 | 2.73E-09 |
| FOS | ZNF324B | 3.55E-11 |
| FOS | RND1 | 1.27E-17 |
| FOS | TRIM16L | 2.29E-18 |
| FOS | SH3RF2 | 1.73E-18 |
| FOS | ZSWIM4 | 1.29E-18 |
| FOS | PBX3 | 1.22E-18 |
| FOS | HOMER2 | 1.05E-18 |
| FOS | TGFB2 | 4.18E-19 |
| FOS | HS3ST2 | 3.08E-19 |
| FOS | PPP1R1C | 2.49E-19 |
| FOSB | ATF3 | 36.29394446 |
| FOSB | ZFP36 | 32.14929001 |
| FOSB | JUN | 29.4792639 |
| FOSB | EGR1 | 29.18573106 |
| FOSB | NR4A1 | 26.82101372 |
| FOSB | HSP90AA1 | 24.34016816 |
| FOSB | PPP1R15A | 22.41423456 |
| FOSB | HSPA1A | 22.26623625 |
| FOSB | DNAJB1 | 21.63971492 |
| FOSB | HSPA1B | 21.2249222 |
| FOSB | FOS | 20.6644089 |
| FOSB | JUND | 18.4079916 |
| FOSB | JUNB | 15.90393411 |
| FOSB | ID3 | 14.75003548 |
| FOSB | HSPH1 | 13.651875 |
| FOSB | IRF1 | 13.62999903 |
| FOSB | DUSP1 | 13.36656047 |
| FOSB | NFKBIZ | 13.2969104 |
| FOSB | BAG3 | 12.56240603 |
| FOSB | PNRC1 | 12.52158395 |
| FOSB | EIF4A3 | 12.46531399 |
| FOSB | CTGF | 12.36252441 |
| FOSB | ZFP36L1 | 12.05376006 |
| FOSB | HSP90AB1 | 11.58968055 |
| FOSB | SOCS3 | 10.84148329 |
| FOSB | EGR2 | 10.75283688 |
| FOSB | KLF4 | 10.59561559 |
| FOSB | IER2 | 10.59302759 |
| FOSB | HSPA8 | 10.57412978 |
| FOSB | DNAJA1 | 10.08529372 |
| FOSB | RGS16 | 9.931447128 |
| FOSB | CYR61 | 9.831677184 |
| FOSB | MYC | 9.249031175 |
| FOSB | GADD45B | 9.189795465 |
| FOSB | BRD2 | 8.750969818 |
| FOSB | KLF6 | 8.656916207 |
| FOSB | KLF2 | 8.432747323 |
| FOSB | RND3 | 8.415921302 |
| FOSB | SRSF7 | 7.612920774 |
| FOSB | KLF10 | 7.548339669 |
| FOSB | PHLDA1 | 7.240287479 |
| FOSB | CDKN1A | 6.988078742 |
| FOSB | RHOB | 6.892804625 |
| FOSB | BHLHE40 | 6.874433319 |
| FOSB | SLC38A2 | 6.870326743 |
| FOSB | CCNL1 | 6.869963956 |
| FOSB | ADAMTS1 | 6.689439539 |
| FOSB | HES1 | 6.52595475 |
| FOSB | IER3 | 6.455745039 |
| FOSB | H3F3B | 6.212924048 |
| FOSB | TCF12 | 6.078942245 |
| FOSB | SAT1 | 5.909590509 |
| FOSB | TNFAIP3 | 5.820783027 |
| FOSB | DDIT4 | 5.61073275 |
| FOSB | FYN | 5.396907668 |
| FOSB | MCL1 | 5.251102864 |
| FOSB | UBB | 5.221045366 |
| FOSB | TUBB2A | 5.040104236 |
| FOSB | PHPT1 | 5.021155218 |
| FOSB | EMP1 | 5.010814357 |
| FOSB | CXCL1 | 4.904668647 |
| FOSB | ERRFI1 | 4.889057348 |
| FOSB | ARL6IP5 | 4.735848581 |
| FOSB | MAFF | 4.514961094 |
| FOSB | IL6 | 4.496957988 |
| FOSB | VCAM1 | 4.198793169 |
| FOSB | BRD1 | 4.162553851 |
| FOSB | MGST3 | 3.881022573 |
| FOSB | TWF2 | 3.805879939 |
| FOSB | SRSF3 | 3.694185093 |
| FOSB | NEAT1 | 3.672471905 |
| FOSB | CSRNP1 | 3.670816731 |
| FOSB | CRISPLD1 | 3.632360085 |
| FOSB | IER5 | 3.528468176 |
| FOSB | DDX3X | 3.482033802 |
| FOSB | ID1 | 3.4604111 |
| FOSB | C11orf96 | 3.37208517 |
| FOSB | CC2D1B | 3.199082818 |
| FOSB | ABCC5 | 3.18753871 |
| FOSB | EIF1 | 3.042491535 |
| FOSB | SAMD9 | 2.991553917 |
| FOSB | PPIL2 | 2.987764225 |
| FOSB | CEBPB | 2.857072859 |
| FOSB | SRSF2 | 2.827706781 |
| FOSB | GNPDA1 | 2.824647104 |
| FOSB | DDX5 | 2.728625046 |
| FOSB | ID4 | 2.695334477 |
| FOSB | EIF4A1 | 2.611709712 |
| FOSB | EIF5 | 2.60844233 |
| FOSB | GEM | 2.593065058 |
| FOSB | CDC14A | 2.576893147 |
| FOSB | BTG1 | 2.521322464 |
| FOSB | CXCL3 | 2.439103781 |
| FOSB | ADM | 2.435697934 |
| FOSB | ZFAND5 | 2.409662398 |
| FOSB | FARP2 | 2.383040414 |
| FOSB | FAM3C | 2.370790348 |
| FOSB | PBXIP1 | 2.328496385 |
| FOSB | PRC1 | 2.23415587 |
| FOSB | LRRC4C | 2.21574834 |
| FOSB | NFIL3 | 2.21026745 |
| FOSB | KIF3A | 2.165832115 |
| FOSB | NR4A2 | 2.117200871 |
| FOSB | SERPINE1 | 2.107575844 |
| FOSB | RGS2 | 2.091547759 |
| FOSB | SPICE1 | 2.088504121 |
| FOSB | DNAJB4 | 2.074335333 |
| FOSB | GOLGA8B | 2.065770641 |
| FOSB | MAFB | 2.036884521 |
| FOSB | TRA2B | 2.016395272 |
| FOSB | MVP | 1.921682179 |
| FOSB | UBE2S | 1.919494565 |
| FOSB | INPPL1 | 1.871902673 |
| FOSB | CD83 | 1.868527313 |
| FOSB | DDIT3 | 1.857265291 |
| FOSB | LONP2 | 1.839449811 |
| FOSB | MYADM | 1.838215974 |
| FOSB | PPDPF | 1.781407438 |
| FOSB | FKBP3 | 1.775429392 |
| FOSB | MSX1 | 1.751614159 |
| FOSB | IL6ST | 1.748731612 |
| FOSB | HEXIM1 | 1.748560792 |
| FOSB | LTA4H | 1.736887272 |
| FOSB | CAST | 1.716389839 |
| FOSB | UBC | 1.702186748 |
| FOSB | SGK1 | 1.659702673 |
| FOSB | DCUN1D5 | 1.657711903 |
| FOSB | CCNK | 1.646158211 |
| FOSB | CXCL16 | 1.638120243 |
| FOSB | TGFBR1 | 1.599215642 |
| FOSB | BBS7 | 1.536956305 |
| FOSB | KRT16 | 1.535294814 |
| FOSB | PRR7 | 1.522541977 |
| FOSB | CLEC11A | 1.46799997 |
| FOSB | CRB3 | 1.462006394 |
| FOSB | ITGB1 | 1.445209446 |
| FOSB | RBFOX2 | 1.423625021 |
| FOSB | LIPE | 1.422876911 |
| FOSB | MSC | 1.409359128 |
| FOSB | SQSTM1 | 1.384682606 |
| FOSB | LMNA | 1.373112904 |
| FOSB | ARHGEF7 | 1.346054326 |
| FOSB | CTSO | 1.335272443 |
| FOSB | ARPC2 | 1.329326606 |
| FOSB | DUSP5 | 1.311133004 |
| FOSB | RBM39 | 1.30923752 |
| FOSB | AHNAK2 | 1.306519329 |
| FOSB | GTF2H2C | 1.277626803 |
| FOSB | SOX2 | 1.260544352 |
| FOSB | ANTXR2 | 1.243042406 |
| FOSB | PTHLH | 1.234265236 |
| FOSB | PMVK | 1.232745396 |
| FOSB | ODF2L | 1.229031967 |
| FOSB | DBF4 | 1.219717282 |
| FOSB | CBFA2T2 | 1.21800992 |
| FOSB | ZNF703 | 1.202047634 |
| FOSB | TRAPPC3 | 1.179677722 |
| FOSB | COTL1 | 1.176586075 |
| FOSB | COL16A1 | 1.156851491 |
| FOSB | SERTAD1 | 1.143818503 |
| FOSB | MON2 | 1.135980095 |
| FOSB | TPBG | 1.134694666 |
| FOSB | PAF1 | 1.12418427 |
| FOSB | ANKRD49 | 1.099957291 |
| FOSB | ZWILCH | 1.083503929 |
| FOSB | AQP3 | 1.07737445 |
| FOSB | ZFAND2A | 1.064912852 |
| FOSB | CTNNBIP1 | 1.061665817 |
| FOSB | FLRT2 | 1.048532636 |
| FOSB | TGIF1 | 1.037274846 |
| FOSB | GTF2IRD1 | 1.018251917 |
| FOSB | FOSB | 1 |
| FOSB | AHCYL2 | 0.989554764 |
| FOSB | TSC22D1 | 0.986541735 |
| FOSB | NUMB | 0.965841369 |
| FOSB | RRAD | 0.962234771 |
| FOSB | CHST1 | 0.937151332 |
| FOSB | LAMC1 | 0.934864752 |
| FOSB | TPM4 | 0.927685764 |
| FOSB | SPATS2 | 0.910733087 |
| FOSB | MYL12B | 0.895602523 |
| FOSB | PGBD4 | 0.894580778 |
| FOSB | VCAN | 0.893002038 |
| FOSB | INSIG1 | 0.892800289 |
| FOSB | ATG3 | 0.881152096 |
| FOSB | SPTBN1 | 0.871525153 |
| FOSB | EGR3 | 0.866700761 |
| FOSB | FOXO3 | 0.849893952 |
| FOSB | LTBP1 | 0.839604788 |
| FOSB | SREBF2 | 0.820701922 |
| FOSB | UBE2O | 0.789988129 |
| FOSB | RPLP2 | 0.784833593 |
| FOSB | NCOA6 | 0.766358568 |
| FOSB | KLF11 | 0.766078043 |
| FOSB | SHANK3 | 0.764369554 |
| FOSB | OAF | 0.745708605 |
| FOSB | KIFC1 | 0.742483589 |
| FOSB | CH25H | 0.717232403 |
| FOSB | INHBA | 0.715673613 |
| FOSB | HAS2 | 0.709042256 |
| FOSB | RAI1 | 0.707600642 |
| FOSB | KLF9 | 0.700252539 |
| FOSB | KRT17 | 0.691651219 |
| FOSB | DENND4B | 0.678968622 |
| FOSB | GOLGA3 | 0.67352432 |
| FOSB | INSIG2 | 0.669785785 |
| FOSB | GADD45A | 0.662899221 |
| FOSB | HMOX1 | 0.661694183 |
| FOSB | AKIRIN2 | 0.653093901 |
| FOSB | SBSN | 0.648783339 |
| FOSB | ZSCAN2 | 0.648267645 |
| FOSB | ARHGEF2 | 0.640416552 |
| FOSB | PFN1 | 0.637363658 |
| FOSB | UBE4A | 0.633399508 |
| FOSB | EYA4 | 0.627405824 |
| FOSB | ZNF219 | 0.616186518 |
| FOSB | GLCCI1 | 0.611227346 |
| FOSB | BAMBI | 0.589858528 |
| FOSB | CA8 | 0.58733388 |
| FOSB | KLF12 | 0.574556964 |
| FOSB | RBBP6 | 0.566267333 |
| FOSB | LARP7 | 0.565410029 |
| FOSB | SGPP2 | 0.547432761 |
| FOSB | PDE2A | 0.537238491 |
| FOSB | VIPR2 | 0.52592698 |
| FOSB | MSI2 | 0.523257206 |
| FOSB | SLC35E4 | 0.52317648 |
| FOSB | ANXA1 | 0.523109601 |
| FOSB | ATP2C1 | 0.522951249 |
| FOSB | POU2F1 | 0.515376743 |
| FOSB | CYLD | 0.500800592 |
| FOSB | ZEB2 | 0.49504321 |
| FOSB | PAQR7 | 0.493990945 |
| FOSB | MYL5 | 0.493823545 |
| FOSB | LIF | 0.486423854 |
| FOSB | SGSM2 | 0.481206201 |
| FOSB | LRRC14 | 0.479325253 |
| FOSB | PTN | 0.479137312 |
| FOSB | CELF1 | 0.476592985 |
| FOSB | CFL2 | 0.472345666 |
| FOSB | SLC20A2 | 0.470832765 |
| FOSB | MME | 0.469923503 |
| FOSB | FAM19A2 | 0.469418817 |
| FOSB | NOLC1 | 0.46619131 |
| FOSB | ADCK5 | 0.464088114 |
| FOSB | NDRG1 | 0.461517814 |
| FOSB | MED30 | 0.459796314 |
| FOSB | RNF157 | 0.458680669 |
| FOSB | ACYP2 | 0.446414934 |
| FOSB | PGS1 | 0.440688862 |
| FOSB | HAMP | 0.436084742 |
| FOSB | PLEKHG5 | 0.431245461 |
| FOSB | CDC37L1 | 0.428771649 |
| FOSB | ZFAND2B | 0.420018455 |
| FOSB | GULP1 | 0.419691243 |
| FOSB | RIPK2 | 0.418812818 |
| FOSB | GPBP1 | 0.414449573 |
| FOSB | DAPK1 | 0.410087981 |
| FOSB | C16orf72 | 0.409664502 |
| FOSB | STAU1 | 0.404948942 |
| FOSB | FOXJ2 | 0.397133001 |
| FOSB | CCAR1 | 0.396317947 |
| FOSB | CDS1 | 0.394545434 |
| FOSB | ENOX1 | 0.391506296 |
| FOSB | CLCN3 | 0.39023957 |
| FOSB | GFRA1 | 0.389291657 |
| FOSB | ZNF200 | 0.386236853 |
| FOSB | TMEM2 | 0.376774568 |
| FOSB | CCDC68 | 0.373404682 |
| FOSB | ZNF608 | 0.372460073 |
| FOSB | SPATA2 | 0.370839913 |
| FOSB | ECE1 | 0.370504709 |
| FOSB | LGI2 | 0.36951221 |
| FOSB | HSD11B1 | 0.36620704 |
| FOSB | STX11 | 0.363117274 |
| FOSB | CCDC61 | 0.361818205 |
| FOSB | EXOC6 | 0.360730612 |
| FOSB | PLEK2 | 0.358517558 |
| FOSB | CLTC | 0.357288229 |
| FOSB | ARL5B | 0.355134197 |
| FOSB | PLK2 | 0.355042527 |
| FOSB | CDC42EP4 | 0.354858303 |
| FOSB | S1PR2 | 0.352806416 |
| FOSB | VCL | 0.348150096 |
| FOSB | DDX19A | 0.346555046 |
| FOSB | RIPK4 | 0.345780424 |
| FOSB | FLI1 | 0.345634624 |
| FOSB | NOG | 0.344453903 |
| FOSB | GTF2H2 | 0.343629363 |
| FOSB | MKL1 | 0.336968736 |
| FOSB | ARL4D | 0.335156842 |
| FOSB | HHIP | 0.332405408 |
| FOSB | GNAO1 | 0.331234171 |
| FOSB | TMEM100 | 0.329685866 |
| FOSB | ZNF552 | 0.328871474 |
| FOSB | RALGPS2 | 0.326049179 |
| FOSB | ZNF460 | 0.324816435 |
| FOSB | CHODL | 0.323681027 |
| FOSB | CMKLR1 | 0.323322775 |
| FOSB | DAAM2 | 0.322417938 |
| FOSB | SH2B3 | 0.322216094 |
| FOSB | SNRNP27 | 0.322203095 |
| FOSB | PRSS22 | 0.317399514 |
| FOSB | PAN2 | 0.317129129 |
| FOSB | KCND3 | 0.315679901 |
| FOSB | GTF3C1 | 0.315475547 |
| FOSB | SEMA4B | 0.31499225 |
| FOSB | RAB30 | 0.312939041 |
| FOSB | ENAH | 0.312029885 |
| FOSB | ESYT1 | 0.311585062 |
| FOSB | USP48 | 0.310712079 |
| FOSB | CDK6 | 0.310413787 |
| FOSB | USP3 | 0.310226323 |
| FOSB | CALCRL | 0.309169693 |
| FOSB | FAM19A5 | 0.308178015 |
| FOSB | HOXB5 | 0.306734805 |
| FOSB | RPP38 | 0.306035225 |
| FOSB | SIAH1 | 0.305852348 |
| FOSB | LMO3 | 0.301703708 |
| FOSB | LONRF3 | 0.296914086 |
| FOSB | LTN1 | 0.293586102 |
| FOSB | ZNF141 | 0.293537474 |
| FOSB | VDR | 0.293520446 |
| FOSB | CWC22 | 0.292101962 |
| FOSB | RAD9A | 0.291436859 |
| FOSB | C1orf21 | 0.290914727 |
| FOSB | PARG | 0.289388311 |
| FOSB | RORA | 0.287996703 |
| FOSB | VASH1 | 0.287922884 |
| FOSB | ZC3H4 | 0.28694014 |
| FOSB | SLC38A6 | 0.286575546 |
| FOSB | TAF7 | 0.286354373 |
| FOSB | DNMT3A | 0.286302493 |
| FOSB | CCDC130 | 0.286265224 |
| FOSB | AR | 0.285561567 |
| FOSB | SGIP1 | 0.281003777 |
| FOSB | BTF3L4 | 0.280534065 |
| FOSB | GSC | 0.279014921 |
| FOSB | STYXL1 | 0.27892583 |
| FOSB | TADA2B | 0.278442799 |
| FOSB | CD36 | 0.276127855 |
| FOSB | RSPO3 | 0.276001781 |
| FOSB | DUSP6 | 0.275804806 |
| FOSB | UBN2 | 0.274476498 |
| FOSB | STX5 | 0.269172184 |
| FOSB | DSEL | 0.267377809 |
| FOSB | TRIM8 | 0.265834163 |
| FOSB | IFRD1 | 0.26534433 |
| FOSB | SSH1 | 0.263615785 |
| FOSB | TAOK1 | 0.263229184 |
| FOSB | SLC2A1 | 0.262622364 |
| FOSB | CDR2L | 0.261649648 |
| FOSB | CLIP4 | 0.260934835 |
| FOSB | ASTE1 | 0.260766088 |
| FOSB | MRPS17 | 0.26023927 |
| FOSB | HOXD1 | 0.257639845 |
| FOSB | PEX12 | 0.256013364 |
| FOSB | ARL13B | 0.254540444 |
| FOSB | PCSK2 | 0.247460334 |
| FOSB | ENG | 0.246755334 |
| FOSB | FOSL2 | 0.24672035 |
| FOSB | HIST1H4E | 0.245397193 |
| FOSB | HDC | 0.242828079 |
| FOSB | REV1 | 0.242563047 |
| FOSB | SPG21 | 0.242373079 |
| FOSB | STX7 | 0.237295043 |
| FOSB | KIAA0141 | 0.236237911 |
| FOSB | HPCAL1 | 0.236005227 |
| FOSB | GABARAPL1 | 0.23508096 |
| FOSB | LYN | 0.230139243 |
| FOSB | NOVA1 | 0.228331808 |
| FOSB | WDR82 | 0.227741568 |
| FOSB | EIF5AL1 | 0.227168257 |
| FOSB | GNL2 | 0.226943778 |
| FOSB | KIAA0556 | 0.226695249 |
| FOSB | SENP2 | 0.225758411 |
| FOSB | PXN | 0.225663335 |
| FOSB | TBC1D22B | 0.223615897 |
| FOSB | SUV39H1 | 0.22315562 |
| FOSB | C5orf51 | 0.222762908 |
| FOSB | CTNNAL1 | 0.222093007 |
| FOSB | RDH10 | 0.221366553 |
| FOSB | DHCR7 | 0.221339852 |
| FOSB | ELOVL6 | 0.218848325 |
| FOSB | FLT1 | 0.218736326 |
| FOSB | PPP1R10 | 0.218386204 |
| FOSB | LRCH1 | 0.21536196 |
| FOSB | SLC23A2 | 0.215305448 |
| FOSB | SCAF1 | 0.215149703 |
| FOSB | PPP1R12A | 0.21375194 |
| FOSB | FAM57A | 0.212485522 |
| FOSB | ACAP1 | 0.212111551 |
| FOSB | PPP2R2A | 0.212086423 |
| FOSB | CNTN1 | 0.211927597 |
| FOSB | AFF4 | 0.211915793 |
| FOSB | PITPNM2 | 0.2111877 |
| FOSB | HTATSF1 | 0.210520322 |
| FOSB | RNF19A | 0.210321118 |
| FOSB | LAMA3 | 0.208029372 |
| FOSB | PER1 | 0.206051893 |
| FOSB | SATB1 | 0.204974845 |
| FOSB | ATP1B3 | 0.204658194 |
| FOSB | PCSK6 | 0.204165112 |
| FOSB | MPP2 | 0.203201215 |
| FOSB | ACHE | 0.192860043 |
| FOSB | MAN1A2 | 0.191750247 |
| FOSB | ZNF423 | 0.191489675 |
| FOSB | LETM2 | 0.191176304 |
| FOSB | PEX11G | 0.190177032 |
| FOSB | SRC | 0.18928572 |
| FOSB | TMEM47 | 0.188942542 |
| FOSB | AUTS2 | 0.180345532 |
| FOSB | CTCF | 0.180344996 |
| FOSB | RBM23 | 0.179752449 |
| FOSB | CLDN4 | 0.179371541 |
| FOSB | TMEM51 | 0.179334395 |
| FOSB | SPTAN1 | 0.177825604 |
| FOSB | ODC1 | 0.175989859 |
| FOSB | CCNY | 0.175308478 |
| FOSB | C1orf35 | 0.171757129 |
| FOSB | OTUD6B | 0.171114834 |
| FOSB | CSNK1G3 | 0.170751626 |
| FOSB | CBLL1 | 0.170338776 |
| FOSB | ZNF720 | 0.169344074 |
| FOSB | GIMAP7 | 0.167286942 |
| FOSB | ZNF548 | 0.165996773 |
| FOSB | RNF11 | 0.164882549 |
| FOSB | OSBPL5 | 0.161948201 |
| FOSB | TUFT1 | 0.15996256 |
| FOSB | ITGA5 | 0.159209213 |
| FOSB | FOXP1 | 0.158393309 |
| FOSB | AASDHPPT | 0.157713283 |
| FOSB | THOC1 | 0.156936131 |
| FOSB | UNK | 0.156360689 |
| FOSB | MIDN | 0.156090096 |
| FOSB | RORB | 0.154141372 |
| FOSB | RARA | 0.153168996 |
| FOSB | CCDC120 | 0.153018458 |
| FOSB | FILIP1 | 0.150012095 |
| FOSB | SCYL2 | 0.149998502 |
| FOSB | FAM24B | 0.147163869 |
| FOSB | KPNA4 | 0.146942644 |
| FOSB | ARC | 0.145239645 |
| FOSB | SHC2 | 0.142822788 |
| FOSB | UBN1 | 0.141291392 |
| FOSB | PDZD2 | 0.140473667 |
| FOSB | ZDHHC8 | 0.139927043 |
| FOSB | JUP | 0.138355027 |
| FOSB | ELK3 | 0.13116293 |
| FOSB | PTPRS | 0.130617728 |
| FOSB | CSK | 0.130315665 |
| FOSB | PDE7A | 0.129221747 |
| FOSB | TANC1 | 0.12872778 |
| FOSB | EFNA3 | 0.121895711 |
| FOSB | IGFL1 | 0.12066873 |
| FOSB | MYO1B | 0.118211714 |
| FOSB | CAB39L | 0.113993227 |
| FOSB | AKIRIN1 | 0.106561619 |
| FOSB | ZFYVE27 | 0.102439306 |
| FOSB | PRKD1 | 0.100438241 |
| FOSB | RCCD1 | 0.093485762 |
| FOSB | TM4SF18 | 0.08961704 |
| FOSB | DPM1 | 0.087492859 |
| FOSB | THADA | 0.086352741 |
| FOSB | UBE2QL1 | 0.084571614 |
| FOSB | ADCYAP1 | 0.082842309 |
| FOSB | CPEB1 | 0.075655723 |
| FOSB | C10orf55 | 0.072555388 |
| FOSB | ZNF543 | 0.072036684 |
| FOSB | ICA1 | 0.070586266 |
| FOSB | STC2 | 0.065682819 |
| FOSB | ELF4 | 0.062933142 |
| FOSB | TMEM59L | 0.059639928 |
| FOSB | ARHGEF5 | 0.050734308 |
| FOSB | CACNA2D3 | 0.03495815 |
| FOSB | CCDC82 | 0.012345268 |
| FOSB | RIOK3 | 0.006841842 |
| FOSB | NUP214 | 0.004340982 |
| FOSB | LFNG | 0.00309723 |
| FOSB | CHD6 | 0.001929263 |
| FOSB | STARD10 | 0.001828671 |
| FOSB | IRF6 | 0.001090634 |
| FOSB | DENND4A | 0.001049428 |
| FOSB | DNTTIP1 | 0.000566391 |
| FOSB | PDE4D | 0.000285042 |
| FOSB | KRT31 | 8.08E-05 |
| FOSB | PRX | 5.35E-05 |
| FOSB | ATF5 | 7.74E-06 |
| FOSB | LMCD1 | 4.97E-06 |
| FOSB | CCDC144B | 3.77E-06 |
| FOSB | LCP1 | 5.61E-07 |
| FOSB | RAP1GAP | 2.61E-07 |
| FOSB | ZNF687 | 8.22E-08 |
| FOSB | RALGAPA2 | 6.87E-08 |
| FOSB | DLAT | 2.07E-09 |
| FOSB | KRT80 | 3.01E-10 |
| FOSB | ERMP1 | 9.67E-11 |
| FOSB | BBC3 | 1.77E-18 |
| FOSB | IL22RA1 | 1.43E-18 |
| FOSB | USP24 | 1.40E-18 |
| FOSB | CLK4 | 1.37E-18 |
| FOSB | CADM4 | 9.91E-19 |
| FOSB | RFFL | 7.84E-19 |
| FOSB | GCNT2 | 6.03E-19 |
| FOSB | FAM133A | 4.80E-19 |
| FOSB | SMARCE1 | 4.12E-19 |
| FOSB | STARD8 | 2.58E-19 |
| FOSB | GOLGA7B | 1.99E-19 |
| FOSL1 | KRT17 | 12.52157096 |
| FOSL1 | SFN | 8.323405056 |
| FOSL1 | KRT16 | 6.010654192 |
| FOSL1 | NAMPT | 5.861728254 |
| FOSL1 | TNFAIP3 | 5.725657542 |
| FOSL1 | MAFF | 5.584109641 |
| FOSL1 | HNRNPA0 | 5.238708703 |
| FOSL1 | IRF1 | 5.208892517 |
| FOSL1 | BICD2 | 5.173720497 |
| FOSL1 | TUBB2A | 5.084360278 |
| FOSL1 | SOD2 | 4.223480199 |
| FOSL1 | NFKBIZ | 4.217137819 |
| FOSL1 | ATF3 | 4.184178916 |
| FOSL1 | CCL20 | 4.172399405 |
| FOSL1 | POGLUT1 | 4.118229556 |
| FOSL1 | KRT14 | 4.045797286 |
| FOSL1 | MT2A | 3.90743106 |
| FOSL1 | CDKN1A | 3.480078091 |
| FOSL1 | RPL14 | 3.454379967 |
| FOSL1 | UBIAD1 | 3.287505897 |
| FOSL1 | IRF6 | 3.153446247 |
| FOSL1 | PMAIP1 | 3.091014833 |
| FOSL1 | RPL35 | 3.05706975 |
| FOSL1 | MT1X | 3.031551782 |
| FOSL1 | BLOC1S1 | 3.030067288 |
| FOSL1 | TACSTD2 | 2.967809428 |
| FOSL1 | NFKBIA | 2.862525191 |
| FOSL1 | MYCBP2 | 2.763962698 |
| FOSL1 | PERP | 2.602188861 |
| FOSL1 | AQP3 | 2.574535364 |
| FOSL1 | GSTP1 | 2.370821918 |
| FOSL1 | KRT5 | 2.340830553 |
| FOSL1 | PAWR | 2.263289848 |
| FOSL1 | RNF26 | 2.246533376 |
| FOSL1 | ZNF524 | 2.208297701 |
| FOSL1 | RAPGEFL1 | 2.030537305 |
| FOSL1 | PABPC1 | 2.005530008 |
| FOSL1 | CYCS | 1.859527267 |
| FOSL1 | DMKN | 1.817046192 |
| FOSL1 | HNRNPK | 1.801841919 |
| FOSL1 | NOP10 | 1.77425138 |
| FOSL1 | COX8A | 1.667536746 |
| FOSL1 | PRNP | 1.665130996 |
| FOSL1 | IL6 | 1.599824703 |
| FOSL1 | PGAP2 | 1.546400911 |
| FOSL1 | PNRC1 | 1.545714073 |
| FOSL1 | NONO | 1.512463493 |
| FOSL1 | COX7B | 1.457467832 |
| FOSL1 | HADHA | 1.448054546 |
| FOSL1 | FAM96A | 1.440479416 |
| FOSL1 | SERPINA12 | 1.438473015 |
| FOSL1 | PPP1R15A | 1.438056431 |
| FOSL1 | RPL36AL | 1.42896263 |
| FOSL1 | ZNF107 | 1.412426243 |
| FOSL1 | TOLLIP | 1.408982191 |
| FOSL1 | DHDDS | 1.401678125 |
| FOSL1 | DSP | 1.39167683 |
| FOSL1 | IPP | 1.385268022 |
| FOSL1 | PROCR | 1.361015335 |
| FOSL1 | EIF1B | 1.304214011 |
| FOSL1 | JUN | 1.276996323 |
| FOSL1 | WDR60 | 1.263071086 |
| FOSL1 | URB1 | 1.255380863 |
| FOSL1 | ATP1B3 | 1.238334131 |
| FOSL1 | MAFA | 1.221611646 |
| FOSL1 | CALML5 | 1.216295399 |
| FOSL1 | KRT1 | 1.199064689 |
| FOSL1 | HOXB6 | 1.135606475 |
| FOSL1 | SIAH2 | 1.129632538 |
| FOSL1 | UFC1 | 1.09890734 |
| FOSL1 | ANKRD37 | 1.081089121 |
| FOSL1 | CAPG | 1.041237323 |
| FOSL1 | ZNF331 | 1.035218567 |
| FOSL1 | MXD1 | 1.010037859 |
| FOSL1 | HSD17B8 | 1.006459011 |
| FOSL1 | GJB6 | 1.002233978 |
| FOSL1 | FOSL1 | 1 |
| FOSL1 | HSP90AA1 | 0.968126594 |
| FOSL1 | RAN | 0.90531011 |
| FOSL1 | NFIL3 | 0.90218306 |
| FOSL1 | EML1 | 0.89304949 |
| FOSL1 | LDHA | 0.89148179 |
| FOSL1 | S100A6 | 0.85695809 |
| FOSL1 | RPS4X | 0.842963137 |
| FOSL1 | THY1 | 0.839577046 |
| FOSL1 | AMMECR1L | 0.834061542 |
| FOSL1 | MT1M | 0.832634099 |
| FOSL1 | PHLDA2 | 0.819185983 |
| FOSL1 | NEAT1 | 0.813105783 |
| FOSL1 | EEF1D | 0.810756063 |
| FOSL1 | PSMB5 | 0.80745573 |
| FOSL1 | KRTDAP | 0.801088485 |
| FOSL1 | WEE1 | 0.797033137 |
| FOSL1 | OTUD6B | 0.787764547 |
| FOSL1 | POP1 | 0.782756198 |
| FOSL1 | FGFR3 | 0.763695117 |
| FOSL1 | ZFP36 | 0.746035002 |
| FOSL1 | RRM2 | 0.743480239 |
| FOSL1 | RPS21 | 0.741816645 |
| FOSL1 | RPP14 | 0.72936426 |
| FOSL1 | KLF4 | 0.708359481 |
| FOSL1 | DST | 0.699945961 |
| FOSL1 | LGALS7 | 0.689148851 |
| FOSL1 | TSPAN3 | 0.686628761 |
| FOSL1 | STRBP | 0.678388244 |
| FOSL1 | FAU | 0.673833232 |
| FOSL1 | CYB5A | 0.664548141 |
| FOSL1 | LAPTM4B | 0.658620655 |
| FOSL1 | HSDL2 | 0.648679313 |
| FOSL1 | MRPL32 | 0.634447734 |
| FOSL1 | NOL7 | 0.62330865 |
| FOSL1 | C6orf141 | 0.623054373 |
| FOSL1 | FGFBP1 | 0.621572634 |
| FOSL1 | COX6A1 | 0.616638258 |
| FOSL1 | RPL9 | 0.610920427 |
| FOSL1 | ATP1A1 | 0.609367239 |
| FOSL1 | VPS53 | 0.598863124 |
| FOSL1 | PCOLCE | 0.597123126 |
| FOSL1 | SRSF7 | 0.593770218 |
| FOSL1 | NOP58 | 0.59282339 |
| FOSL1 | LY6D | 0.568581235 |
| FOSL1 | TXN | 0.565427271 |
| FOSL1 | SVEP1 | 0.563333881 |
| FOSL1 | CCND1 | 0.562245925 |
| FOSL1 | CCDC59 | 0.559481234 |
| FOSL1 | STEAP1 | 0.558511179 |
| FOSL1 | F2RL1 | 0.558305687 |
| FOSL1 | SAP18 | 0.558303841 |
| FOSL1 | GPR137 | 0.540011655 |
| FOSL1 | NDUFB11 | 0.5292867 |
| FOSL1 | CD55 | 0.525008922 |
| FOSL1 | PHLDA1 | 0.519454419 |
| FOSL1 | HSPA6 | 0.515388827 |
| FOSL1 | PRSS23 | 0.507722486 |
| FOSL1 | RPSA | 0.50441125 |
| FOSL1 | CALM2 | 0.502495812 |
| FOSL1 | FUCA2 | 0.501274063 |
| FOSL1 | RDH12 | 0.49754998 |
| FOSL1 | GJB5 | 0.496791052 |
| FOSL1 | ANXA1 | 0.496042544 |
| FOSL1 | SLC25A39 | 0.493663344 |
| FOSL1 | SPARC | 0.483995796 |
| FOSL1 | PRDM10 | 0.475545297 |
| FOSL1 | SBSN | 0.470788805 |
| FOSL1 | FN1 | 0.470661079 |
| FOSL1 | XBP1 | 0.464824402 |
| FOSL1 | SPINT2 | 0.464165307 |
| FOSL1 | LGALS1 | 0.459631679 |
| FOSL1 | BANF1 | 0.459401993 |
| FOSL1 | RPL24 | 0.458549299 |
| FOSL1 | TTC4 | 0.457440358 |
| FOSL1 | FABP5 | 0.45743013 |
| FOSL1 | DNAJB4 | 0.447687132 |
| FOSL1 | COL6A3 | 0.445732588 |
| FOSL1 | HSPD1 | 0.441888301 |
| FOSL1 | MPPE1 | 0.440433535 |
| FOSL1 | RNF103 | 0.440066249 |
| FOSL1 | DVL1 | 0.430942101 |
| FOSL1 | ZNF577 | 0.428684511 |
| FOSL1 | CBX8 | 0.424658853 |
| FOSL1 | DYNLL2 | 0.413733849 |
| FOSL1 | RNF144B | 0.409821387 |
| FOSL1 | KLF13 | 0.404561738 |
| FOSL1 | TOM1L1 | 0.40304412 |
| FOSL1 | HSPB7 | 0.378819863 |
| FOSL1 | RNF138 | 0.378776999 |
| FOSL1 | RBM42 | 0.376941709 |
| FOSL1 | KLF5 | 0.358772293 |
| FOSL1 | STK38 | 0.357111293 |
| FOSL1 | ORMDL3 | 0.353535495 |
| FOSL1 | KLHL22 | 0.346155822 |
| FOSL1 | ANXA2 | 0.337819019 |
| FOSL1 | GAS1 | 0.336742269 |
| FOSL1 | MAPRE2 | 0.336408753 |
| FOSL1 | SERPINB2 | 0.33358044 |
| FOSL1 | ALG6 | 0.329301392 |
| FOSL1 | EIF3L | 0.327972762 |
| FOSL1 | TSG101 | 0.326531083 |
| FOSL1 | RNF19B | 0.323658148 |
| FOSL1 | WDR18 | 0.321544291 |
| FOSL1 | VRK1 | 0.317404679 |
| FOSL1 | SLC7A1 | 0.317336997 |
| FOSL1 | ZC3H15 | 0.309672503 |
| FOSL1 | IFFO2 | 0.302948938 |
| FOSL1 | USP8 | 0.299353687 |
| FOSL1 | SDR16C5 | 0.294745332 |
| FOSL1 | ANKHD1 | 0.292885923 |
| FOSL1 | GMPR2 | 0.290947101 |
| FOSL1 | RFFL | 0.290062667 |
| FOSL1 | YPEL2 | 0.289920149 |
| FOSL1 | MAPRE3 | 0.286593288 |
| FOSL1 | PNPLA2 | 0.284727121 |
| FOSL1 | LUM | 0.283071125 |
| FOSL1 | PSMA2 | 0.281589792 |
| FOSL1 | UBE2W | 0.279896802 |
| FOSL1 | LHFPL2 | 0.278310697 |
| FOSL1 | MTUS1 | 0.277255145 |
| FOSL1 | SMG7 | 0.276698953 |
| FOSL1 | UQCRFS1 | 0.276392138 |
| FOSL1 | EPS8L2 | 0.276360782 |
| FOSL1 | CKB | 0.275674868 |
| FOSL1 | PPM1F | 0.272413115 |
| FOSL1 | RUSC1 | 0.269572754 |
| FOSL1 | RMND5B | 0.268876368 |
| FOSL1 | ARPC1A | 0.264872077 |
| FOSL1 | TDP1 | 0.258807995 |
| FOSL1 | NSF | 0.256432154 |
| FOSL1 | ULBP1 | 0.255739587 |
| FOSL1 | WWOX | 0.253743776 |
| FOSL1 | BLCAP | 0.251345253 |
| FOSL1 | IMMT | 0.250042227 |
| FOSL1 | NLRP1 | 0.246652858 |
| FOSL1 | CKS2 | 0.241351086 |
| FOSL1 | PHF23 | 0.241259286 |
| FOSL1 | CADM4 | 0.240951192 |
| FOSL1 | SERF2 | 0.240392604 |
| FOSL1 | C1orf21 | 0.237684054 |
| FOSL1 | STX5 | 0.23629115 |
| FOSL1 | RNF111 | 0.235440113 |
| FOSL1 | BAG2 | 0.234576009 |
| FOSL1 | GLG1 | 0.234571778 |
| FOSL1 | B3GAT3 | 0.234320072 |
| FOSL1 | SHB | 0.2312442 |
| FOSL1 | UTP23 | 0.228564138 |
| FOSL1 | UBLCP1 | 0.225626259 |
| FOSL1 | CYS1 | 0.207159018 |
| FOSL1 | OCIAD2 | 0.205862384 |
| FOSL1 | RHOV | 0.199449722 |
| FOSL1 | SLCO4A1 | 0.192653807 |
| FOSL1 | CYLD | 0.192522049 |
| FOSL1 | SENP2 | 0.191598502 |
| FOSL1 | DUSP23 | 0.190895951 |
| FOSL1 | CLDN1 | 0.190387677 |
| FOSL1 | IL15 | 0.178072481 |
| FOSL1 | MAD1L1 | 0.175354983 |
| FOSL1 | CST6 | 0.174211371 |
| FOSL1 | MAL2 | 0.172489709 |
| FOSL1 | ADAMTS9 | 0.171678549 |
| FOSL1 | KHNYN | 0.170829558 |
| FOSL1 | PPP1R13L | 0.170759024 |
| FOSL1 | CXCL16 | 0.164452538 |
| FOSL1 | SCARB1 | 0.160557419 |
| FOSL1 | ABCB8 | 0.160507334 |
| FOSL1 | OVOL1 | 0.157958878 |
| FOSL1 | AAGAB | 0.156933578 |
| FOSL1 | FAM102A | 0.150232431 |
| FOSL1 | NOL3 | 0.1349933 |
| FOSL1 | TOPBP1 | 0.132837475 |
| FOSL1 | SEMA6B | 0.129641976 |
| FOSL1 | SYN1 | 0.129625874 |
| FOSL1 | C1orf74 | 0.125228008 |
| FOSL1 | RAB6A | 0.125081308 |
| FOSL1 | LRRC49 | 0.121818261 |
| FOSL1 | ZADH2 | 0.121406098 |
| FOSL1 | TYRO3 | 0.118670291 |
| FOSL1 | ALDH2 | 0.091088563 |
| FOSL1 | BNC1 | 0.090895799 |
| FOSL1 | PTP4A3 | 0.086381802 |
| FOSL1 | PRG4 | 0.076908182 |
| FOSL1 | HAS3 | 0.0644103 |
| FOSL1 | HSPA7 | 0.061367011 |
| FOSL1 | SEMA4A | 0.060113851 |
| FOSL1 | SCYL3 | 0.050324259 |
| FOSL1 | TMC8 | 0.044236278 |
| FOSL1 | ITGA3 | 0.031268212 |
| FOSL1 | CBX6 | 0.018519768 |
| FOSL1 | UCK2 | 0.007880843 |
| FOSL1 | SLC25A23 | 0.001264726 |
| FOSL1 | ATF2 | 0.000506328 |
| FOSL1 | SPATA5 | 1.46E-18 |
| FOSL2 | CD44 | 10.84313052 |
| FOSL2 | RPS18 | 9.594898008 |
| FOSL2 | RPL37A | 6.500613233 |
| FOSL2 | VIM | 6.352395236 |
| FOSL2 | RPL31 | 4.771765508 |
| FOSL2 | SRP9 | 4.26277402 |
| FOSL2 | WNT2B | 4.121928477 |
| FOSL2 | NAP1L1 | 3.893368699 |
| FOSL2 | ICAM1 | 2.243818541 |
| FOSL2 | GMPPB | 2.184729983 |
| FOSL2 | OSTF1 | 2.147139794 |
| FOSL2 | GADD45A | 2.07578401 |
| FOSL2 | DPT | 2.053509073 |
| FOSL2 | PDE4A | 2.020121359 |
| FOSL2 | GAS1 | 2.011768823 |
| FOSL2 | FSCN1 | 1.969913423 |
| FOSL2 | SOCS3 | 1.958232035 |
| FOSL2 | VAPA | 1.906313168 |
| FOSL2 | GSTP1 | 1.890161677 |
| FOSL2 | TIMM10 | 1.834331582 |
| FOSL2 | NIPAL2 | 1.7821339 |
| FOSL2 | PSMA7 | 1.720289063 |
| FOSL2 | PTCH2 | 1.672241702 |
| FOSL2 | LRRC58 | 1.652301527 |
| FOSL2 | NUDCD2 | 1.623097784 |
| FOSL2 | PLBD2 | 1.610757703 |
| FOSL2 | RPL23 | 1.609131306 |
| FOSL2 | DIO2 | 1.58627786 |
| FOSL2 | CTDSP2 | 1.585539156 |
| FOSL2 | FXYD3 | 1.585071941 |
| FOSL2 | CDC45 | 1.584399085 |
| FOSL2 | BCKDHB | 1.582854539 |
| FOSL2 | FTSJ3 | 1.562947739 |
| FOSL2 | POLR3GL | 1.555124939 |
| FOSL2 | ITSN2 | 1.545080782 |
| FOSL2 | RPL39 | 1.491377022 |
| FOSL2 | ZNF318 | 1.490073014 |
| FOSL2 | ITPR2 | 1.483975418 |
| FOSL2 | BIK | 1.478451706 |
| FOSL2 | KLF4 | 1.405384863 |
| FOSL2 | CKAP2 | 1.389749241 |
| FOSL2 | ASXL2 | 1.341404935 |
| FOSL2 | RGS10 | 1.339214038 |
| FOSL2 | CYR61 | 1.329441747 |
| FOSL2 | ZFAND5 | 1.322511146 |
| FOSL2 | CYC1 | 1.320007697 |
| FOSL2 | RPL13A | 1.308322872 |
| FOSL2 | LARP6 | 1.30111132 |
| FOSL2 | THBS1 | 1.26560642 |
| FOSL2 | C1orf109 | 1.248834523 |
| FOSL2 | RIN1 | 1.201292646 |
| FOSL2 | CDK10 | 1.192992739 |
| FOSL2 | INPP5A | 1.188100121 |
| FOSL2 | MYC | 1.186565979 |
| FOSL2 | SLC4A7 | 1.164425709 |
| FOSL2 | EIF5 | 1.162421066 |
| FOSL2 | AKTIP | 1.151184917 |
| FOSL2 | NCKAP5 | 1.145901232 |
| FOSL2 | BRF2 | 1.140283172 |
| FOSL2 | ANXA5 | 1.128847462 |
| FOSL2 | TUBGCP5 | 1.123938024 |
| FOSL2 | TSPO | 1.110172129 |
| FOSL2 | CDYL | 1.09596316 |
| FOSL2 | COPA | 1.073246778 |
| FOSL2 | CELSR2 | 1.069849907 |
| FOSL2 | C9orf72 | 1.065319803 |
| FOSL2 | RPL7 | 1.059222448 |
| FOSL2 | XRN2 | 1.057273182 |
| FOSL2 | JAM3 | 1.048918854 |
| FOSL2 | ACTR3 | 1.040041048 |
| FOSL2 | ZNF703 | 1.036324143 |
| FOSL2 | MAEA | 1.031777004 |
| FOSL2 | TNC | 1.022773402 |
| FOSL2 | CLIC1 | 1.017450414 |
| FOSL2 | SIRT1 | 1.013148535 |
| FOSL2 | MT1E | 1.003029342 |
| FOSL2 | FOSL2 | 1 |
| FOSL2 | HIF1A | 0.984216979 |
| FOSL2 | HSP90AB1 | 0.980256377 |
| FOSL2 | CAP1 | 0.963937362 |
| FOSL2 | WTAP | 0.963892944 |
| FOSL2 | DUSP10 | 0.960701348 |
| FOSL2 | RPS3 | 0.936518377 |
| FOSL2 | ASNSD1 | 0.932871683 |
| FOSL2 | SSR4 | 0.927371252 |
| FOSL2 | CUL1 | 0.926011781 |
| FOSL2 | RPL21 | 0.916854029 |
| FOSL2 | CSGALNACT1 | 0.916314705 |
| FOSL2 | PCOLCE | 0.910283649 |
| FOSL2 | FGGY | 0.900817728 |
| FOSL2 | RNF14 | 0.897478968 |
| FOSL2 | NOTCH3 | 0.89412658 |
| FOSL2 | CROCC | 0.882433027 |
| FOSL2 | CD74 | 0.874107725 |
| FOSL2 | SOD2 | 0.871415563 |
| FOSL2 | PTTG1IP | 0.870150809 |
| FOSL2 | KLF9 | 0.864811842 |
| FOSL2 | SDCBP | 0.856692866 |
| FOSL2 | EDA2R | 0.855588179 |
| FOSL2 | TRA2B | 0.853522763 |
| FOSL2 | PRNP | 0.851862438 |
| FOSL2 | TWIST2 | 0.84947517 |
| FOSL2 | MT1X | 0.818912266 |
| FOSL2 | UBXN1 | 0.818361351 |
| FOSL2 | FZD6 | 0.817951372 |
| FOSL2 | PGRMC1 | 0.814386617 |
| FOSL2 | CNOT6L | 0.812697917 |
| FOSL2 | RANBP10 | 0.809137739 |
| FOSL2 | MAPRE1 | 0.806824681 |
| FOSL2 | CDCA5 | 0.786102948 |
| FOSL2 | PHF10 | 0.783946218 |
| FOSL2 | ZFP36L2 | 0.782948182 |
| FOSL2 | ETNK1 | 0.76849824 |
| FOSL2 | PHF21A | 0.767481243 |
| FOSL2 | CXCL2 | 0.760161398 |
| FOSL2 | FOXN2 | 0.751718194 |
| FOSL2 | FOSB | 0.745681766 |
| FOSL2 | SYDE2 | 0.743922151 |
| FOSL2 | MAFG | 0.708391685 |
| FOSL2 | PHIP | 0.702688616 |
| FOSL2 | TAF6L | 0.694036099 |
| FOSL2 | C11orf65 | 0.686247129 |
| FOSL2 | BAG3 | 0.679516385 |
| FOSL2 | RIMKLB | 0.679196153 |
| FOSL2 | MRPL55 | 0.678485649 |
| FOSL2 | ASS1 | 0.676853535 |
| FOSL2 | NAMPT | 0.662995993 |
| FOSL2 | GPBP1 | 0.659617804 |
| FOSL2 | GREM1 | 0.65653765 |
| FOSL2 | VGLL4 | 0.64730007 |
| FOSL2 | COX7C | 0.643955566 |
| FOSL2 | RPS23 | 0.629550335 |
| FOSL2 | C1orf123 | 0.620209826 |
| FOSL2 | MSX2 | 0.618814626 |
| FOSL2 | HNRNPC | 0.613987957 |
| FOSL2 | DYNLL1 | 0.612960767 |
| FOSL2 | RPS9 | 0.609370068 |
| FOSL2 | NUP205 | 0.607581152 |
| FOSL2 | OSBPL8 | 0.606342753 |
| FOSL2 | TRAK2 | 0.605011059 |
| FOSL2 | TMEM43 | 0.604854205 |
| FOSL2 | FAM168B | 0.60471664 |
| FOSL2 | TACSTD2 | 0.602250773 |
| FOSL2 | COX8A | 0.601490598 |
| FOSL2 | ARHGAP26 | 0.598645516 |
| FOSL2 | SLIT3 | 0.594158277 |
| FOSL2 | ZSCAN21 | 0.594028522 |
| FOSL2 | DDX3X | 0.593893057 |
| FOSL2 | KCNAB2 | 0.590237214 |
| FOSL2 | DCTN3 | 0.58299112 |
| FOSL2 | FNIP2 | 0.579076045 |
| FOSL2 | WDR1 | 0.574021211 |
| FOSL2 | PLXNC1 | 0.573161938 |
| FOSL2 | FGL2 | 0.569081406 |
| FOSL2 | ANXA1 | 0.568440238 |
| FOSL2 | PRMT1 | 0.567977174 |
| FOSL2 | MCL1 | 0.567203459 |
| FOSL2 | CCL19 | 0.5671198 |
| FOSL2 | TUBA4A | 0.565076948 |
| FOSL2 | RERE | 0.562129846 |
| FOSL2 | AMDHD2 | 0.560752263 |
| FOSL2 | HS3ST3A1 | 0.559944109 |
| FOSL2 | ADAMTSL2 | 0.558982 |
| FOSL2 | DLG4 | 0.55720135 |
| FOSL2 | THUMPD3 | 0.553755147 |
| FOSL2 | DGAT2 | 0.551945842 |
| FOSL2 | FCER1A | 0.550091367 |
| FOSL2 | GEM | 0.540843124 |
| FOSL2 | TANC1 | 0.539794747 |
| FOSL2 | ELF1 | 0.532008815 |
| FOSL2 | GTPBP2 | 0.531713675 |
| FOSL2 | PIM3 | 0.529919284 |
| FOSL2 | RPS4X | 0.528260893 |
| FOSL2 | RNASE1 | 0.527393914 |
| FOSL2 | CPEB2 | 0.526623044 |
| FOSL2 | HCFC1 | 0.517478401 |
| FOSL2 | PAK6 | 0.516692571 |
| FOSL2 | KLHL23 | 0.510808921 |
| FOSL2 | SMUG1 | 0.509735418 |
| FOSL2 | RND3 | 0.506155204 |
| FOSL2 | SPIN1 | 0.505591532 |
| FOSL2 | C1QTNF6 | 0.501104749 |
| FOSL2 | NONO | 0.486249363 |
| FOSL2 | NCL | 0.484059049 |
| FOSL2 | GAS7 | 0.480459723 |
| FOSL2 | LRRC26 | 0.477661466 |
| FOSL2 | PRPF38B | 0.472383539 |
| FOSL2 | TUBB2A | 0.471194886 |
| FOSL2 | ADAMTS6 | 0.469831516 |
| FOSL2 | PSMD13 | 0.466250087 |
| FOSL2 | RPS29 | 0.465444266 |
| FOSL2 | TADA1 | 0.464464077 |
| FOSL2 | SMARCA5 | 0.462615654 |
| FOSL2 | GCNT2 | 0.458191854 |
| FOSL2 | CXCL12 | 0.457275124 |
| FOSL2 | PFN1 | 0.457087132 |
| FOSL2 | SIRPA | 0.449531181 |
| FOSL2 | RPL19 | 0.448786478 |
| FOSL2 | S100A10 | 0.447411309 |
| FOSL2 | PSMB4 | 0.446084036 |
| FOSL2 | ST6GAL1 | 0.445737331 |
| FOSL2 | REEP4 | 0.440314381 |
| FOSL2 | CASK | 0.439928041 |
| FOSL2 | RGS16 | 0.438371714 |
| FOSL2 | PRPF19 | 0.43831778 |
| FOSL2 | FEZ1 | 0.437605218 |
| FOSL2 | FBXW4 | 0.43467711 |
| FOXC1 | PCYT1A | 6.566825705 |
| FOXC1 | PDIA5 | 5.722846654 |
| FOXC1 | PCMTD2 | 4.364411802 |
| FOXC1 | SCMH1 | 4.331280214 |
| FOXC1 | PDK4 | 4.16299667 |
| FOXC1 | HLA-F | 3.993517108 |
| FOXC1 | FAM19A5 | 3.944048158 |
| FOXC1 | CEP164 | 3.915387718 |
| FOXC1 | POMGNT1 | 3.740923073 |
| FOXC1 | GPC4 | 3.615506904 |
| FOXC1 | GOSR1 | 3.477731973 |
| FOXC1 | MYH14 | 3.438523528 |
| FOXC1 | TNIP2 | 3.385246811 |
| FOXC1 | CACHD1 | 3.356313173 |
| FOXC1 | SUPT5H | 3.289744427 |
| FOXC1 | DDX52 | 3.14403698 |
| FOXC1 | TSPAN4 | 3.064360085 |
| FOXC1 | NDUFS1 | 2.70790038 |
| FOXC1 | SORBS2 | 2.50001207 |
| FOXC1 | MKRN1 | 2.324031594 |
| FOXC1 | PDLIM7 | 2.284765833 |
| FOXC1 | CLDN4 | 2.218142326 |
| FOXC1 | AP1B1 | 2.168295526 |
| FOXC1 | ADPRHL1 | 2.08294336 |
| FOXC1 | MAPRE3 | 2.046193386 |
| FOXC1 | CYTL1 | 1.956559319 |
| FOXC1 | CYHR1 | 1.920478405 |
| FOXC1 | MEGF9 | 1.714320445 |
| FOXC1 | STK4 | 1.646414971 |
| FOXC1 | IRF1 | 1.62776177 |
| FOXC1 | RPS6KB1 | 1.616365796 |
| FOXC1 | RASA3 | 1.474428328 |
| FOXC1 | ZNF395 | 1.431938409 |
| FOXC1 | TSHZ2 | 1.314254833 |
| FOXC1 | NR4A2 | 1.286465563 |
| FOXC1 | A4GALT | 1.250940807 |
| FOXC1 | HEATR5A | 1.240483646 |
| FOXC1 | ZNF622 | 1.20971157 |
| FOXC1 | SMYD3 | 1.197854977 |
| FOXC1 | ZNF605 | 1.162258609 |
| FOXC1 | OLFM2 | 1.139425241 |
| FOXC1 | HOMER1 | 1.111795497 |
| FOXC1 | CNKSR3 | 1.101395872 |
| FOXC1 | ATF7IP | 1.098254278 |
| FOXC1 | VGLL3 | 1.085481823 |
| FOXC1 | FNTB | 1.079817226 |
| FOXC1 | FOXC1 | 1 |
| FOXC1 | MTX2 | 0.963480706 |
| FOXC1 | SNX33 | 0.938833435 |
| FOXC1 | LOX | 0.936638554 |
| FOXC1 | PRNP | 0.877072548 |
| FOXC1 | CPNE1 | 0.790421407 |
| FOXC1 | TNFAIP2 | 0.779880555 |
| FOXC1 | ACTR3C | 0.771209124 |
| FOXC1 | ACTN4 | 0.758150584 |
| FOXC1 | PRDM1 | 0.743809163 |
| FOXC1 | CDKN1C | 0.69831556 |
| FOXC1 | KIAA1217 | 0.663551193 |
| FOXC1 | POGK | 0.661439868 |
| FOXC1 | CRBN | 0.658352477 |
| FOXC1 | SRGN | 0.593706623 |
| FOXC1 | NUP54 | 0.583661946 |
| FOXC1 | NFKBIZ | 0.559404824 |
| FOXC1 | NDUFA4L2 | 0.506906489 |
| FOXC1 | FAM24B | 0.489726553 |
| FOXC1 | CHRDL1 | 0.480130987 |
| FOXC1 | SMOC2 | 0.475228813 |
| FOXC1 | SDC4 | 0.474920287 |
| FOXC1 | AIG1 | 0.443754396 |
| FOXC1 | ACSL1 | 0.440370032 |
| FOXC1 | RADIL | 0.439957307 |
| FOXC1 | INTS6 | 0.436033702 |
| FOXC1 | SNX2 | 0.368039133 |
| FOXC1 | GALC | 0.364997711 |
| FOXC1 | TRDMT1 | 0.335466938 |
| FOXC1 | MAPK6 | 0.329503927 |
| FOXC1 | SEMA6D | 0.317903634 |
| FOXC1 | NR2F2 | 0.306958023 |
| FOXC1 | ARHGAP32 | 0.298740855 |
| FOXC1 | RNF2 | 0.285668305 |
| FOXC1 | SLC35C2 | 0.283476632 |
| FOXC1 | GPSM2 | 0.280772098 |
| FOXC1 | PALM | 0.267937123 |
| FOXC1 | EGFLAM | 0.255941696 |
| FOXC1 | C17orf58 | 0.251902574 |
| FOXC1 | MNS1 | 0.232227249 |
| FOXC1 | SLITRK5 | 0.219847289 |
| FOXC1 | PCK2 | 0.21066888 |
| FOXC1 | LRRC2 | 0.209918906 |
| FOXC1 | PRRG1 | 0.207252569 |
| FOXC1 | CROCC | 0.206842079 |
| FOXC1 | EFNA1 | 0.200885288 |
| FOXC1 | UBE4A | 0.191767643 |
| FOXC1 | KLHL20 | 0.191126511 |
| FOXC1 | TRIM9 | 0.180535963 |
| FOXC1 | SMAD6 | 0.1756797 |
| FOXC1 | ENTPD5 | 0.165695992 |
| FOXC1 | GPATCH8 | 0.162562579 |
| FOXC1 | VWA5A | 0.149578349 |
| FOXC1 | CTDSP2 | 0.14484979 |
| FOXC1 | ZC3H6 | 0.143125711 |
| FOXC1 | MRPS9 | 0.141932161 |
| FOXC1 | NR2C2 | 0.127585033 |
| FOXC1 | CCDC149 | 0.115479662 |
| FOXC1 | SDK1 | 0.111384451 |
| FOXC1 | EZH1 | 0.096085951 |
| FOXC1 | PPM1D | 0.08003923 |
| FOXC1 | PROM2 | 0.073574105 |
| FOXC1 | PRKG2 | 0.070825005 |
| FOXC1 | SGMS1 | 0.05878746 |
| FOXC1 | CYSLTR1 | 0.032686439 |
| FOXC1 | LST1 | 0.022747137 |
| FOXC1 | RAB40B | 3.55E-05 |
| FOXC2 | HMGB2 | 4.601357469 |
| FOXC2 | MRPS14 | 2.794618272 |
| FOXC2 | CHRDL1 | 2.020299917 |
| FOXC2 | ZNF516 | 1.743153192 |
| FOXC2 | BNC2 | 1.66552977 |
| FOXC2 | KLF5 | 1.643999475 |
| FOXC2 | ITGA6 | 1.458249165 |
| FOXC2 | STARD13 | 1.173164932 |
| FOXC2 | SMAD4 | 1.13840952 |
| FOXC2 | INO80E | 0.93086945 |
| FOXC2 | TJP1 | 0.78765985 |
| FOXC2 | AP2S1 | 0.77265779 |
| FOXC2 | CSRP1 | 0.720089351 |
| FOXC2 | AKAP13 | 0.650056576 |
| FOXC2 | PKDCC | 0.632021584 |
| FOXC2 | RASSF8 | 0.333532073 |
| FOXC2 | ZFHX4 | 0.281604182 |
| FOXC2 | STK38L | 0.255511937 |
| FOXC2 | NT5C2 | 0.207865813 |
| FOXC2 | ZBTB40 | 0.069290266 |
| FOXD3 | FOXD3 | 1 |
| FOXD3 | KLHL24 | 0.886433167 |
| FOXD3 | GAS6 | 0.359830976 |
| FOXD3 | DEDD | 0.355613987 |
| FOXD3 | CTSO | 0.331819743 |
| FOXD3 | HIPK2 | 0.294220588 |
| FOXD3 | NCOA2 | 0.28320767 |
| FOXD3 | TTC39C | 0.220996399 |
| FOXF2 | SIAH1 | 5.076380653 |
| FOXF2 | MAP4 | 3.56430533 |
| FOXF2 | PLXNB2 | 2.433139979 |
| FOXF2 | PAIP1 | 1.868450822 |
| FOXF2 | EDNRA | 1.669321995 |
| FOXF2 | KAT2B | 1.64821902 |
| FOXF2 | ANO6 | 1.526391798 |
| FOXF2 | KANK2 | 1.508245013 |
| FOXF2 | ABCA1 | 1.156865996 |
| FOXF2 | HPRT1 | 1.151215112 |
| FOXF2 | AP1G1 | 1.132782529 |
| FOXF2 | RARB | 1.130175138 |
| FOXF2 | GTF2A2 | 1.090996966 |
| FOXF2 | MMP2 | 1.071480192 |
| FOXF2 | FOXP1 | 1.067168743 |
| FOXF2 | MORF4L2 | 1.041463287 |
| FOXF2 | FOXF2 | 1 |
| FOXF2 | ZNF280D | 0.940032713 |
| FOXF2 | CEP170 | 0.881914457 |
| FOXF2 | RAB3GAP2 | 0.848428439 |
| FOXF2 | SORBS3 | 0.824335792 |
| FOXF2 | SEL1L3 | 0.815828605 |
| FOXF2 | MYO6 | 0.719652606 |
| FOXF2 | VCL | 0.71342719 |
| FOXF2 | PRICKLE2 | 0.708038723 |
| FOXF2 | PPP1R13B | 0.674792835 |
| FOXF2 | KIF2A | 0.666153556 |
| FOXF2 | PCM1 | 0.648292813 |
| FOXF2 | CSAD | 0.611635572 |
| FOXF2 | NRSN2 | 0.590995081 |
| FOXF2 | FRMD6 | 0.588033299 |
| FOXF2 | ANKRD28 | 0.559062419 |
| FOXF2 | COQ10B | 0.540462603 |
| FOXF2 | PCDH9 | 0.536383104 |
| FOXF2 | ZNF791 | 0.529189952 |
| FOXF2 | CACYBP | 0.474583473 |
| FOXF2 | KCTD1 | 0.466575225 |
| FOXF2 | CBWD1 | 0.465957251 |
| FOXF2 | GORAB | 0.465346753 |
| FOXF2 | PCYT1A | 0.456895678 |
| FOXF2 | TNRC6B | 0.452358668 |
| FOXJ2 | ATP10D | 2.891564375 |
| FOXJ2 | GALK2 | 2.743020035 |
| FOXJ2 | TSC22D1 | 2.740431069 |
| FOXJ2 | DUSP14 | 2.170320218 |
| FOXJ2 | ANP32A | 1.99115639 |
| FOXJ2 | FARS2 | 1.964749449 |
| FOXJ2 | PHLDB2 | 1.769956897 |
| FOXJ2 | UCHL3 | 1.765639821 |
| FOXJ2 | NR4A3 | 1.61963095 |
| FOXJ2 | KIFC3 | 1.230640443 |
| FOXJ2 | ADM | 1.174606762 |
| FOXJ2 | MDFIC | 1.166122301 |
| FOXJ2 | IL13RA1 | 1.162024513 |
| FOXJ2 | FOXJ2 | 1 |
| FOXJ2 | TRIM8 | 0.910026812 |
| FOXJ2 | PTH1R | 0.651457827 |
| FOXJ2 | RFX8 | 0.533990531 |
| FOXJ2 | PAMR1 | 0.406295965 |
| FOXJ2 | PITX2 | 0.385251305 |
| FOXJ2 | ANO4 | 0.278581666 |
| FOXJ2 | NEDD4 | 0.269597794 |
| FOXJ2 | ZMIZ1 | 0.245026864 |
| FOXJ2 | LRP5L | 0.238162244 |
| FOXJ2 | SPTAN1 | 0.236987001 |
| FOXJ2 | DCAF11 | 0.229069749 |
| FOXJ2 | FMR1 | 0.19658549 |
| FOXJ2 | KDM6A | 0.192528667 |
| FOXJ2 | ADCY3 | 0.186454138 |
| FOXJ2 | TBL1X | 0.162631758 |
| FOXJ2 | WDR26 | 0.14252009 |
| FOXJ2 | ORAI1 | 0.111013629 |
| FOXJ2 | NNT | 0.109405932 |
| FOXJ2 | NF1 | 0.087499767 |
| FOXJ2 | TMEFF2 | 0.08603084 |
| FOXJ2 | CNKSR3 | 0.065793982 |
| FOXJ2 | PLCB2 | 0.041184612 |
| FOXJ2 | ZNF691 | 0.037802824 |
| FOXJ2 | GPRC5C | 0.02752828 |
| FOXJ2 | ZNF710 | 0.021161097 |
| FOXK1 | MAPKAP1 | 3.797746625 |
| FOXK1 | PHIP | 2.741948992 |
| FOXK1 | IMMP2L | 2.451280343 |
| FOXK1 | FYCO1 | 2.23978353 |
| FOXK1 | CA5B | 2.102576052 |
| FOXK1 | H2AFV | 2.048613236 |
| FOXK1 | DNMBP | 2.005836032 |
| FOXK1 | NRG1 | 1.873875765 |
| FOXK1 | CGNL1 | 1.718780524 |
| FOXK1 | BTBD1 | 1.718543461 |
| FOXK1 | NDRG2 | 1.710235507 |
| FOXK1 | SF1 | 1.689033561 |
| FOXK1 | PRNP | 1.664923296 |
| FOXK1 | MBTPS1 | 1.635302492 |
| FOXK1 | NLGN4X | 1.610917638 |
| FOXK1 | SLC41A2 | 1.520232744 |
| FOXK1 | PCMTD2 | 1.506655785 |
| FOXK1 | HELZ | 1.420087475 |
| FOXK1 | C19orf47 | 1.398663213 |
| FOXK1 | OTUD4 | 1.347214475 |
| FOXK1 | AVPI1 | 1.345098061 |
| FOXK1 | MSH2 | 1.343403726 |
| FOXK1 | TNFAIP6 | 1.320693004 |
| FOXK1 | PLOD2 | 1.313139586 |
| FOXK1 | ELOVL1 | 1.281748965 |
| FOXK1 | PEX2 | 1.279756499 |
| FOXK1 | CAMK2N1 | 1.138901628 |
| FOXK1 | ETV1 | 1.138088329 |
| FOXK1 | EEF2 | 1.084902333 |
| FOXK1 | TBC1D15 | 1.071926137 |
| FOXK1 | FOXK1 | 1 |
| FOXK1 | GRHPR | 0.989549106 |
| FOXK1 | KLF9 | 0.945538343 |
| FOXK1 | FMO1 | 0.943961666 |
| FOXK1 | HSPBAP1 | 0.943927858 |
| FOXK1 | ETV3 | 0.942380785 |
| FOXK1 | PPIG | 0.923525243 |
| FOXK1 | ENDOD1 | 0.873012657 |
| FOXK1 | MSI2 | 0.865363618 |
| FOXK1 | SULF2 | 0.8025738 |
| FOXK1 | SP1 | 0.799610487 |
| FOXK1 | RIN3 | 0.782584426 |
| FOXK1 | ACVR1B | 0.775953981 |
| FOXK1 | TMEM200A | 0.770460728 |
| FOXK1 | CNOT6L | 0.739525207 |
| FOXK1 | HECW2 | 0.73646388 |
| FOXK1 | FOXS1 | 0.699228969 |
| FOXK1 | LTV1 | 0.665525124 |
| FOXK1 | LMCD1 | 0.66455221 |
| FOXK1 | BCL9 | 0.663550766 |
| FOXK1 | KCND3 | 0.659905915 |
| FOXK1 | MRPL19 | 0.654122206 |
| FOXK1 | TLE3 | 0.634517669 |
| FOXK1 | AGTRAP | 0.626411205 |
| FOXK1 | SMS | 0.599116512 |
| FOXK1 | SGK1 | 0.591100661 |
| FOXK1 | NCK1 | 0.588515979 |
| FOXK1 | KCNK2 | 0.551321123 |
| FOXK1 | ENPP2 | 0.549273383 |
| FOXK1 | NRP2 | 0.548074755 |
| FOXK1 | LSM14A | 0.544428266 |
| FOXK1 | H2AFY | 0.544138433 |
| FOXK1 | TMTC1 | 0.538159622 |
| FOXK1 | AKAP12 | 0.524718429 |
| FOXK1 | FRK | 0.51951387 |
| FOXK1 | HIPK3 | 0.511682757 |
| FOXK1 | RARA | 0.502734935 |
| FOXK1 | IMPDH1 | 0.49259629 |
| FOXK1 | ZSCAN2 | 0.492217172 |
| FOXK1 | SDCBP | 0.491194067 |
| FOXK1 | BICC1 | 0.482837154 |
| FOXK1 | MTMR1 | 0.477754025 |
| FOXK1 | LRRC58 | 0.47211254 |
| FOXK1 | CDON | 0.470592945 |
| FOXK1 | GLT8D2 | 0.462786062 |
| FOXK1 | SCRG1 | 0.452068111 |
| FOXK1 | PDK4 | 0.448232296 |
| FOXK1 | SRP54 | 0.446553546 |
| FOXK1 | CSNK1E | 0.44403738 |
| FOXK1 | ATF2 | 0.437398466 |
| FOXK1 | WNT2B | 0.413443825 |
| FOXK1 | ITGA1 | 0.396875984 |
| FOXK1 | THSD7A | 0.3639422 |
| FOXK1 | ATXN7L1 | 0.359080157 |
| FOXK1 | BCAR3 | 0.356522232 |
| FOXK1 | ARNT | 0.353714966 |
| FOXK1 | P2RY14 | 0.347731251 |
| FOXK1 | STXBP5 | 0.33220718 |
| FOXK1 | PDLIM3 | 0.319555341 |
| FOXK1 | RABGAP1L | 0.305717062 |
| FOXK1 | MTSS1 | 0.305147037 |
| FOXK1 | NR1D1 | 0.303214854 |
| FOXK1 | TRDMT1 | 0.290852986 |
| FOXK1 | LGR4 | 0.276834022 |
| FOXK1 | TBPL1 | 0.274944642 |
| FOXK1 | PAWR | 0.274442019 |
| FOXK1 | ZCCHC11 | 0.265685115 |
| FOXK1 | SRPK2 | 0.260627009 |
| FOXK1 | PPP2R2A | 0.256799389 |
| FOXK1 | SDHB | 0.243365394 |
| FOXK1 | MRAP2 | 0.242275942 |
| FOXK1 | EHD2 | 0.235241815 |
| FOXK1 | MBNL2 | 0.222176863 |
| FOXK1 | MLLT1 | 0.213003382 |
| FOXK1 | UTRN | 0.209397408 |
| FOXK1 | DAAM1 | 0.208469289 |
| FOXK1 | DDAH1 | 0.18263913 |
| FOXK1 | NOVA1 | 0.180071294 |
| FOXK1 | ANKRD44 | 0.169723056 |
| FOXK1 | TMEM17 | 0.155026934 |
| FOXK1 | SNX4 | 0.143844677 |
| FOXK1 | STX16 | 0.142295528 |
| FOXK1 | PLEKHO1 | 0.136348651 |
| FOXK1 | ST6GAL1 | 0.123830069 |
| FOXK1 | NSMCE1 | 0.122231948 |
| FOXK1 | SMAD6 | 0.120794004 |
| FOXK1 | ARL6IP6 | 0.120782355 |
| FOXK1 | CYP4F12 | 0.091047108 |
| FOXK1 | LRRK2 | 0.067130616 |
| FOXK1 | GSTCD | 0.066959032 |
| FOXK1 | GCA | 0.050692036 |
| FOXL1 | OMA1 | 2.810217908 |
| FOXL1 | LRCH2 | 2.087403622 |
| FOXL1 | KLHL7 | 1.634480864 |
| FOXL1 | ATP1B2 | 1.277432841 |
| FOXL1 | FOXL1 | 1 |
| FOXL1 | FMO1 | 0.893402513 |
| FOXL1 | RRP1 | 0.800262679 |
| FOXL1 | STAMBPL1 | 0.743997956 |
| FOXL1 | GEM | 0.740995438 |
| FOXL1 | SETD7 | 0.517684464 |
| FOXL1 | RHOBTB3 | 0.500479722 |
| FOXL1 | TRPC6 | 0.247223029 |
| FOXL1 | VWA5A | 0.17508463 |
| FOXL1 | CDKN1B | 0.172659683 |
| FOXL1 | DUSP11 | 0.158372149 |
| FOXL1 | SLC25A25 | 0.129378691 |
| FOXL1 | SRPK1 | 0.106053878 |
| FOXL1 | PORCN | 0.075535703 |
| FOXL2 | ECSIT | 2.258800876 |
| FOXL2 | HOXD9 | 1.529878292 |
| FOXL2 | PTH1R | 1.449505931 |
| FOXL2 | RRN3 | 1.423414624 |
| FOXL2 | POLE3 | 0.875415464 |
| FOXL2 | HOXD8 | 0.728731566 |
| FOXL2 | SBNO2 | 0.641462912 |
| FOXL2 | DIO2 | 0.560403995 |
| FOXL2 | FREM1 | 0.530262236 |
| FOXL2 | BRMS1L | 0.433903684 |
| FOXM1 | TPX2 | 14.63121606 |
| FOXM1 | CENPF | 12.75971118 |
| FOXM1 | KIF18B | 11.85565015 |
| FOXM1 | PBK | 6.087390793 |
| FOXM1 | DIAPH3 | 5.954256681 |
| FOXM1 | SHCBP1 | 3.211346154 |
| FOXM1 | KIF14 | 2.275204969 |
| FOXM1 | NFYA | 1.095817153 |
| FOXN2 | PATZ1 | 3.249523613 |
| FOXN2 | DDHD1 | 3.140182693 |
| FOXN2 | DENND5A | 3.095980063 |
| FOXN2 | FOXD1 | 2.977494062 |
| FOXN2 | TRMT112 | 2.686457845 |
| FOXN2 | ABR | 1.825527033 |
| FOXN2 | ATF4 | 1.809221079 |
| FOXN2 | AP1M2 | 1.706599138 |
| FOXN2 | SKA3 | 1.326290067 |
| FOXN2 | R3HDM1 | 1.295562121 |
| FOXN2 | MAP1LC3A | 1.21075247 |
| FOXN2 | EIF4B | 0.908592985 |
| FOXN2 | STARD3NL | 0.836974978 |
| FOXN2 | NDUFAB1 | 0.824512885 |
| FOXN2 | PRRX1 | 0.818758726 |
| FOXN2 | NTRK2 | 0.808847879 |
| FOXN2 | KCNA5 | 0.728178025 |
| FOXN2 | SOX4 | 0.652875044 |
| FOXN2 | SLC25A12 | 0.645891672 |
| FOXN2 | ATF3 | 0.607226703 |
| FOXN2 | BRWD3 | 0.573807015 |
| FOXN2 | ICK | 0.567131869 |
| FOXN2 | CPEB1 | 0.561444477 |
| FOXN2 | AP4E1 | 0.551118334 |
| FOXN2 | SYT17 | 0.55092509 |
| FOXN2 | UBE2G2 | 0.548936751 |
| FOXN2 | NDUFA1 | 0.519647876 |
| FOXN2 | PTGS2 | 0.461103312 |
| FOXN2 | HNRNPD | 0.441625859 |
| FOXN2 | CDC26 | 0.441568869 |
| FOXN2 | G3BP2 | 0.439039664 |
| FOXN3 | RALBP1 | 4.051747082 |
| FOXN3 | CLIP2 | 2.664646586 |
| FOXN3 | TP53INP2 | 2.261613237 |
| FOXN3 | NEK7 | 2.25155959 |
| FOXN3 | STXBP6 | 2.206911824 |
| FOXN3 | CDK8 | 2.204240612 |
| FOXN3 | NEXN | 2.171167932 |
| FOXN3 | SYTL2 | 1.922813427 |
| FOXN3 | FOXN3 | 1 |
| FOXO1 | SERPINE2 | 4.238536695 |
| FOXO1 | CITED2 | 3.577353509 |
| FOXO1 | FGFR1 | 2.589516502 |
| FOXO1 | MSX1 | 2.559328276 |
| FOXO1 | EMCN | 2.309058309 |
| FOXO1 | CDKN1C | 1.980683751 |
| FOXO1 | MRPS21 | 1.92360593 |
| FOXO1 | CDC14B | 1.815879019 |
| FOXO1 | PDPN | 1.812136948 |
| FOXO1 | GOLIM4 | 1.726339937 |
| FOXO1 | KLF9 | 1.562121607 |
| FOXO1 | SFT2D1 | 1.483212528 |
| FOXO1 | LAMA2 | 1.358625935 |
| FOXO1 | OAF | 1.328546864 |
| FOXO1 | EIF5 | 1.304837751 |
| FOXO1 | HSF2 | 1.222156956 |
| FOXO1 | PACRG | 1.175932398 |
| FOXO1 | RXRA | 1.175650206 |
| FOXO1 | SFXN3 | 1.067613885 |
| FOXO1 | SBDSP1 | 1.066025054 |
| FOXO1 | MYADM | 1.059127632 |
| FOXO1 | EMP1 | 1.048851658 |
| FOXO1 | PRKAR1A | 1.007480549 |
| FOXO1 | FOXO1 | 1 |
| FOXO1 | HES1 | 0.99350557 |
| FOXO1 | KDM2A | 0.986142167 |
| FOXO1 | FAP | 0.939295254 |
| FOXO1 | MEG3 | 0.934028244 |
| FOXO1 | KLF10 | 0.931775812 |
| FOXO1 | CNBP | 0.921697784 |
| FOXO1 | FAM13C | 0.908421746 |
| FOXO1 | LRRC3 | 0.895909123 |
| FOXO1 | COLEC12 | 0.888884985 |
| FOXO1 | CEBPB | 0.874835128 |
| FOXO1 | NFIX | 0.757224049 |
| FOXO1 | RSPO1 | 0.752151354 |
| FOXO1 | IRF1 | 0.703516897 |
| FOXO1 | JUND | 0.690667334 |
| FOXO1 | SOCS3 | 0.662132702 |
| FOXO1 | LEPROT | 0.556744363 |
| FOXO1 | TCF4 | 0.546377511 |
| FOXO1 | TIMP2 | 0.536533081 |
| FOXO1 | PUM1 | 0.522049419 |
| FOXO1 | SLC7A8 | 0.512567011 |
| FOXO1 | PIM1 | 0.473126278 |
| FOXO1 | JUNB | 0.457007244 |
| FOXO1 | DDIT4 | 0.454576143 |
| FOXO1 | SOX2 | 0.431856419 |
| FOXO1 | CAMKK2 | 0.420490288 |
| FOXO1 | RHOH | 0.410641792 |
| FOXO1 | FAM76A | 0.39896044 |
| FOXO1 | DGKA | 0.383833544 |
| FOXO1 | NRP2 | 0.365587767 |
| FOXO1 | PANK1 | 0.336805384 |
| FOXO1 | DLG4 | 0.322834554 |
| FOXO1 | NDE1 | 0.316331592 |
| FOXO1 | PPFIA2 | 0.294488932 |
| FOXO1 | PSKH1 | 0.286413151 |
| FOXO1 | FNIP2 | 0.282891048 |
| FOXO1 | IGF1 | 0.282369995 |
| FOXO1 | ASXL1 | 0.275194406 |
| FOXO1 | ZNF282 | 0.274729075 |
| FOXO1 | COMMD10 | 0.256044721 |
| FOXO1 | ARHGAP18 | 0.252672038 |
| FOXO1 | PCDH9 | 0.243716914 |
| FOXO1 | KCNK2 | 0.240884952 |
| FOXO1 | GPBP1 | 0.234366252 |
| FOXO1 | DUSP3 | 0.227369976 |
| FOXO1 | FOXP1 | 0.224852886 |
| FOXO1 | TACC1 | 0.219347872 |
| FOXO1 | KIF26B | 0.218875811 |
| FOXO1 | AHCYL2 | 0.215766035 |
| FOXO1 | GPSM1 | 0.212282758 |
| FOXO1 | ANKRD28 | 0.207349462 |
| FOXO1 | FRY | 0.198429794 |
| FOXO1 | VGLL3 | 0.197549473 |
| FOXO1 | CCDC92 | 0.196188047 |
| FOXO1 | BCL6 | 0.189107196 |
| FOXO1 | TP53INP1 | 0.185928063 |
| FOXO1 | DCAF11 | 0.184182337 |
| FOXO1 | UST | 0.17747617 |
| FOXO1 | HOXB7 | 0.173305197 |
| FOXO1 | PAK1 | 0.156828619 |
| FOXO1 | ARMC2 | 0.150673111 |
| FOXO1 | LAG3 | 0.150168088 |
| FOXO1 | SULT1A1 | 0.147625393 |
| FOXO1 | TMEM132C | 0.147588008 |
| FOXO1 | FAM53C | 0.141827897 |
| FOXO1 | CCND2 | 0.136732166 |
| FOXO1 | RAB40B | 0.135383782 |
| FOXO1 | IRX1 | 0.134498645 |
| FOXO1 | CACNA1G | 0.130048266 |
| FOXO1 | WDFY3 | 0.124752995 |
| FOXO1 | ELL | 0.116171765 |
| FOXO1 | SP7 | 0.113091696 |
| FOXO1 | COL13A1 | 0.107631862 |
| FOXO1 | TMEM187 | 0.056058413 |
| FOXO1 | MAP3K2 | 0.010137755 |
| FOXO1 | AHDC1 | 0.007120535 |
| FOXO1 | PCGF5 | 0.005286248 |
| FOXO1 | TRIB2 | 0.002634826 |
| FOXO3 | ZFAND5 | 9.24221668 |
| FOXO3 | ZFP36L2 | 7.895189043 |
| FOXO3 | PNRC1 | 4.891463104 |
| FOXO3 | BCL2 | 4.608843736 |
| FOXO3 | HSPB1 | 4.59895799 |
| FOXO3 | PIK3R1 | 4.415704319 |
| FOXO3 | DUSP4 | 4.119095845 |
| FOXO3 | ACTG1 | 4.110069848 |
| FOXO3 | APOD | 3.353108409 |
| FOXO3 | TSC22D3 | 3.044318245 |
| FOXO3 | COL5A2 | 2.957346083 |
| FOXO3 | ARID5B | 2.901511301 |
| FOXO3 | NUCKS1 | 2.786872024 |
| FOXO3 | MEG3 | 2.675331031 |
| FOXO3 | PTPRK | 2.527823725 |
| FOXO3 | KLF9 | 2.464736537 |
| FOXO3 | DDX3X | 2.348986979 |
| FOXO3 | HNRNPA1 | 2.299815419 |
| FOXO3 | MT2A | 2.286472766 |
| FOXO3 | ZNF263 | 2.246956236 |
| FOXO3 | EXTL3 | 2.194483525 |
| FOXO3 | SYNE1 | 2.027319817 |
| FOXO3 | EMP1 | 1.942478299 |
| FOXO3 | THUMPD1 | 1.913061966 |
| FOXO3 | ITGB1 | 1.903829662 |
| FOXO3 | SFRP1 | 1.869926286 |
| FOXO3 | IGF1 | 1.854683388 |
| FOXO3 | ICA1L | 1.849304855 |
| FOXO3 | CNKSR2 | 1.823223863 |
| FOXO3 | MCCC1 | 1.74529327 |
| FOXO3 | KLF4 | 1.740092423 |
| FOXO3 | ARSJ | 1.736860818 |
| FOXO3 | PPP1R1B | 1.594479952 |
| FOXO3 | NFKBIZ | 1.566437308 |
| FOXO3 | TAF7 | 1.530568252 |
| FOXO3 | AHNAK | 1.504399071 |
| FOXO3 | TBX3 | 1.499278152 |
| FOXO3 | DNAJC13 | 1.479233497 |
| FOXO3 | SETDB1 | 1.477184088 |
| FOXO3 | TSC22D1 | 1.47553544 |
| FOXO3 | UBE2D3 | 1.435783929 |
| FOXO3 | EIF3E | 1.431147466 |
| FOXO3 | H1F0 | 1.42002781 |
| FOXO3 | FAM129A | 1.418743222 |
| FOXO3 | NACA | 1.409454228 |
| FOXO3 | CCNI | 1.39839962 |
| FOXO3 | MTMR3 | 1.390375578 |
| FOXO3 | MGLL | 1.38594097 |
| FOXO3 | CBX4 | 1.383429321 |
| FOXO3 | SDHC | 1.350144608 |
| FOXO3 | P2RY14 | 1.310610654 |
| FOXO3 | SCUBE2 | 1.309676645 |
| FOXO3 | CBLL1 | 1.291186005 |
| FOXO3 | MYC | 1.286445328 |
| FOXO3 | PHLDA1 | 1.275759974 |
| FOXO3 | MTMR10 | 1.263905433 |
| FOXO3 | PPDPF | 1.251443238 |
| FOXO3 | MAPRE2 | 1.238593261 |
| FOXO3 | CAV1 | 1.228159126 |
| FOXO3 | RAVER2 | 1.198283461 |
| FOXO3 | WWTR1 | 1.196489933 |
| FOXO3 | PTPRG | 1.187083286 |
| FOXO3 | DCD | 1.185222379 |
| FOXO3 | ANKRD12 | 1.161172403 |
| FOXO3 | RBFOX2 | 1.139069554 |
| FOXO3 | INSR | 1.130886706 |
| FOXO3 | GADD45G | 1.124518476 |
| FOXO3 | TUBA1A | 1.119679981 |
| FOXO3 | UQCRQ | 1.119473806 |
| FOXO3 | MBD1 | 1.105603321 |
| FOXO3 | HDAC9 | 1.093507936 |
| FOXO3 | TXN | 1.067214097 |
| FOXO3 | GREB1 | 1.063508039 |
| FOXO3 | HCLS1 | 1.031128324 |
| FOXO3 | RRBP1 | 1.028059922 |
| FOXO3 | CHST10 | 1.027020201 |
| FOXO3 | FOXO3 | 1 |
| FOXO3 | CXCR4 | 0.991791223 |
| FOXO3 | ACVR2A | 0.990087112 |
| FOXO3 | FICD | 0.988522819 |
| FOXO3 | MT1X | 0.979469229 |
| FOXO3 | MLLT3 | 0.975208025 |
| FOXO3 | RPS26 | 0.960504908 |
| FOXO3 | PRRX1 | 0.950664681 |
| FOXO3 | FAM107A | 0.943895581 |
| FOXO3 | DCLK1 | 0.941589598 |
| FOXO3 | RHOB | 0.939503572 |
| FOXO3 | NEGR1 | 0.937673375 |
| FOXO3 | PLEKHH2 | 0.931228478 |
| FOXO3 | ZFHX3 | 0.927336462 |
| FOXO3 | BHLHE22 | 0.914307972 |
| FOXO3 | RXRA | 0.91047075 |
| FOXO3 | DNAJB12 | 0.905818138 |
| FOXO3 | RHOJ | 0.879839104 |
| FOXO3 | CDKN1A | 0.875761227 |
| FOXO3 | SPRY1 | 0.87281004 |
| FOXO3 | CNOT2 | 0.864242465 |
| FOXO3 | NBPF3 | 0.857221965 |
| FOXO3 | SKP1 | 0.8353457 |
| FOXO3 | TNN | 0.832274161 |
| FOXO3 | H3F3B | 0.811401672 |
| FOXO3 | PGBD4 | 0.807070689 |
| FOXO3 | DDX5 | 0.80550794 |
| FOXO3 | TCF4 | 0.782214515 |
| FOXO3 | ZC3H14 | 0.779781599 |
| FOXO3 | SOCS3 | 0.779364735 |
| FOXO3 | CPE | 0.765282638 |
| FOXO3 | KIAA0040 | 0.764100233 |
| FOXO3 | ANXA6 | 0.733162893 |
| FOXO3 | NTRK2 | 0.732012501 |
| FOXO3 | ABCA6 | 0.715369123 |
| FOXO3 | PFN1 | 0.687796441 |
| FOXO3 | COL6A3 | 0.673614076 |
| FOXO3 | PRDX6 | 0.670971833 |
| FOXO3 | C12orf29 | 0.670149349 |
| FOXO3 | TBL1X | 0.65055651 |
| FOXO3 | APAF1 | 0.649340808 |
| FOXO3 | PPM1G | 0.648159907 |
| FOXO3 | MFSD8 | 0.643055295 |
| FOXO3 | ID3 | 0.623711463 |
| FOXO3 | NAB2 | 0.616186472 |
| FOXO3 | SOCS5 | 0.615899494 |
| FOXO3 | MAFF | 0.604549596 |
| FOXO3 | RBMS3 | 0.60228468 |
| FOXO3 | KLF2 | 0.594562691 |
| FOXO3 | ATAD2 | 0.581530735 |
| FOXO3 | PPIL4 | 0.574907122 |
| FOXO3 | PSMA1 | 0.569072363 |
| FOXO3 | ABL1 | 0.559793993 |
| FOXO3 | SERPING1 | 0.557668025 |
| FOXO3 | MPG | 0.555640195 |
| FOXO3 | SNAI2 | 0.555618626 |
| FOXO3 | GLUL | 0.550989327 |
| FOXO3 | RAP2A | 0.550696686 |
| FOXO3 | SETMAR | 0.547790665 |
| FOXO3 | FOXL1 | 0.546314671 |
| FOXO3 | CENPF | 0.541057836 |
| FOXO3 | LAMA4 | 0.532967619 |
| FOXO3 | TRMT11 | 0.528275475 |
| FOXO3 | ZC3H12B | 0.513769532 |
| FOXO3 | NFASC | 0.502321077 |
| FOXO3 | PDLIM3 | 0.500292706 |
| FOXO3 | CACNA2D2 | 0.498898498 |
| FOXO3 | SOX15 | 0.496500722 |
| FOXO3 | ID1 | 0.495192736 |
| FOXO3 | CCDC47 | 0.489379947 |
| FOXO3 | JUND | 0.471372495 |
| FOXO3 | LAMC3 | 0.470061002 |
| FOXO3 | PLSCR4 | 0.468208317 |
| FOXO3 | DBT | 0.467105711 |
| FOXO3 | PDGFRA | 0.464354255 |
| FOXO3 | THBD | 0.461888613 |
| FOXO3 | DGAT1 | 0.456778893 |
| FOXO3 | OS9 | 0.449561429 |
| FOXO3 | BAG3 | 0.445318872 |
| FOXO3 | APLP2 | 0.443242648 |
| FOXO3 | EGR1 | 0.442919606 |
| FOXO3 | ZNF644 | 0.441897744 |
| FOXO3 | CD9 | 0.441294107 |
| FOXO3 | RBM43 | 0.433487017 |
| FOXO3 | WNK1 | 0.428487794 |
| FOXO3 | NRP1 | 0.426328846 |
| FOXO3 | ZNF821 | 0.407039625 |
| FOXO3 | PDHB | 0.366297203 |
| FOXO3 | AMD1 | 0.355863658 |
| FOXO3 | UBE2B | 0.351781688 |
| FOXO3 | PRLR | 0.317375063 |
| FOXO3 | C1QTNF2 | 0.294492403 |
| FOXO3 | MSX1 | 0.275430067 |
| FOXO3 | FRYL | 0.269867328 |
| FOXO3 | CLDN11 | 0.261279412 |
| FOXO3 | CAPN1 | 0.250054787 |
| FOXO3 | LTBP3 | 0.232340443 |
| FOXO3 | WLS | 0.225575574 |
| FOXO3 | ZMYM4 | 0.214016151 |
| FOXO3 | KDM6A | 0.204632994 |
| FOXO3 | SRF | 0.189027839 |
| FOXO3 | INPP5F | 0.181295159 |
| FOXO3 | ZNF292 | 0.179870321 |
| FOXO3 | PSD3 | 0.136365897 |
| FOXO3 | LNPEP | 0.12604797 |
| FOXO3 | SATB2 | 0.122925892 |
| FOXO3 | NMU | 0.122497282 |
| FOXO3 | DUSP7 | 0.089092937 |
| FOXO3 | XPO7 | 0.084733205 |
| FOXO3 | SLC6A9 | 0.06180418 |
| FOXO3 | MSTO1 | 0.055700376 |
| FOXO3 | RNF38 | 0.038376247 |
| FOXO3 | C12orf75 | 0.036367726 |
| FOXO3 | EMILIN3 | 0.030578737 |
| FOXO3 | PART1 | 0.030429218 |
| FOXO3 | MAFA | 0.027447385 |
| FOXO3 | PCDH10 | 2.66E-19 |
| FOXO3 | LTB4R2 | 2.55E-19 |
| FOXO6 | PDK4 | 3.539860956 |
| FOXO6 | SPG21 | 2.780868548 |
| FOXO6 | CLN5 | 2.194288275 |
| FOXO6 | FBXO21 | 1.5150115 |
| FOXO6 | SEL1L3 | 1.446710145 |
| FOXO6 | BEND6 | 1.217167629 |
| FOXO6 | SLC39A10 | 1.210772204 |
| FOXO6 | ST3GAL5 | 1.154370443 |
| FOXO6 | FBXL3 | 1.14621768 |
| FOXO6 | CSRNP3 | 1.005462467 |
| FOXO6 | GNB1 | 0.818723859 |
| FOXO6 | KIAA1217 | 0.81750038 |
| FOXO6 | ID4 | 0.79274025 |
| FOXO6 | PLSCR1 | 0.753633401 |
| FOXO6 | NDFIP1 | 0.626838966 |
| FOXO6 | DAAM1 | 0.537602802 |
| FOXO6 | RNF2 | 0.479087361 |
| FOXP1 | CTHRC1 | 14.41972142 |
| FOXP1 | POSTN | 6.026757709 |
| FOXP1 | SULF2 | 5.552324192 |
| FOXP1 | HHEX | 4.349801104 |
| FOXP1 | AQP3 | 3.603017176 |
| FOXP1 | UGCG | 2.731453509 |
| FOXP1 | SLC35D1 | 2.646387524 |
| FOXP1 | MEOX2 | 2.538049213 |
| FOXP1 | C12orf57 | 2.358305691 |
| FOXP1 | TPM1 | 2.305506897 |
| FOXP1 | DPP9 | 2.257007237 |
| FOXP1 | HOXB7 | 2.229486435 |
| FOXP1 | HELZ | 2.215222105 |
| FOXP1 | COG3 | 2.149654815 |
| FOXP1 | KIT | 2.140338453 |
| FOXP1 | PLEKHA1 | 1.946019587 |
| FOXP1 | CEP120 | 1.911040413 |
| FOXP1 | KPNA3 | 1.905093325 |
| FOXP1 | USP1 | 1.854383208 |
| FOXP1 | PKN2 | 1.780242306 |
| FOXP1 | ANKH | 1.745746218 |
| FOXP1 | PGS1 | 1.676995939 |
| FOXP1 | ADAR | 1.602259135 |
| FOXP1 | EML2 | 1.551973732 |
| FOXP1 | SOX9 | 1.388004363 |
| FOXP1 | LAMA4 | 1.090163378 |
| FOXP1 | COL5A2 | 1.081747529 |
| FOXP1 | FOXP1 | 1 |
| FOXP1 | PDGFRB | 0.960197255 |
| FOXP1 | AZIN1 | 0.943600547 |
| FOXP1 | TRMT61A | 0.853792438 |
| FOXP1 | ZNF534 | 0.814271645 |
| FOXP1 | COMP | 0.808617484 |
| FOXP1 | KLF4 | 0.792727194 |
| FOXP1 | MIDN | 0.689640969 |
| FOXP1 | PDPN | 0.675688722 |
| FOXP1 | CPM | 0.661573357 |
| FOXP1 | PDK4 | 0.648799947 |
| FOXP1 | TLE3 | 0.648211575 |
| FOXP1 | DDX5 | 0.61349069 |
| FOXP1 | SULF1 | 0.609389519 |
| FOXP1 | FBXL22 | 0.60262872 |
| FOXP1 | CYGB | 0.572738101 |
| FOXP1 | PRICKLE2 | 0.517744559 |
| FOXP1 | EPAS1 | 0.50325235 |
| FOXP1 | HSPB1 | 0.446646326 |
| FOXP2 | PRKAR2B | 4.25743888 |
| FOXP2 | USP12 | 3.80333203 |
| FOXP2 | PDLIM1 | 3.23968673 |
| FOXP2 | OTUD3 | 2.310822369 |
| FOXP2 | TAPBP | 2.251662643 |
| FOXP2 | AMOTL1 | 2.063753275 |
| FOXP2 | STRN3 | 2.042665094 |
| FOXP2 | PPARA | 1.984040127 |
| FOXP2 | HIBADH | 1.951582826 |
| FOXP2 | PRICKLE1 | 1.929129526 |
| FOXP2 | IQSEC1 | 1.884890102 |
| FOXP2 | LIMK2 | 1.848163576 |
| FOXP2 | CTDSPL2 | 1.777673536 |
| FOXP2 | BCL2L11 | 1.761503314 |
| FOXP2 | GRK5 | 1.68683006 |
| FOXP2 | NTN1 | 1.652767141 |
| FOXP2 | YTHDF3 | 1.647122057 |
| FOXP2 | FHOD1 | 1.606110737 |
| FOXP2 | MYO6 | 1.593527617 |
| FOXP2 | CAPN1 | 1.56436293 |
| FOXP2 | RIT1 | 1.501281863 |
| FOXP2 | PPP1R13B | 1.488243526 |
| FOXP2 | PAFAH1B1 | 1.463355161 |
| FOXP2 | DIAPH2 | 1.420756714 |
| FOXP2 | FBXO8 | 1.406051204 |
| FOXP2 | NINJ1 | 1.398147705 |
| FOXP2 | FGD6 | 1.391697792 |
| FOXP2 | LYSMD2 | 1.315958593 |
| FOXP2 | ICAM1 | 1.296746316 |
| FOXP2 | GGA2 | 1.29087166 |
| FOXP2 | MIDN | 1.267823838 |
| FOXP2 | CBR4 | 1.252425462 |
| FOXP2 | SOCS2 | 1.234446902 |
| FOXP2 | PRPSAP1 | 1.086939943 |
| FOXP2 | MASP1 | 1.046219103 |
| FOXP2 | IFNGR2 | 1.027684036 |
| FOXP2 | FOXP2 | 1 |
| FOXP2 | CLIC6 | 0.956993396 |
| FOXP2 | EMP2 | 0.925098596 |
| FOXP2 | TFCP2 | 0.871522658 |
| FOXP2 | TGOLN2 | 0.866792083 |
| FOXP2 | CHURC1 | 0.855060997 |
| FOXP2 | EPHB3 | 0.807885978 |
| FOXP2 | CYBRD1 | 0.803048778 |
| FOXP2 | LZTS2 | 0.789157765 |
| FOXP2 | SCGB2A1 | 0.739719298 |
| FOXP2 | ANPEP | 0.719499857 |
| FOXP2 | DNAJC13 | 0.715956592 |
| FOXP2 | LTBP2 | 0.690074827 |
| FOXP2 | TMEM100 | 0.671538207 |
| FOXP2 | MAMLD1 | 0.644214609 |
| FOXP2 | ZBTB10 | 0.620640345 |
| FOXP2 | HUNK | 0.557947099 |
| FOXP2 | TGIF1 | 0.553525582 |
| FOXP2 | TNC | 0.540533812 |
| FOXP2 | LITAF | 0.520279272 |
| FOXP2 | TNS1 | 0.510390332 |
| FOXP2 | VEZF1 | 0.503161355 |
| FOXP2 | LRRC15 | 0.499558012 |
| FOXP2 | CALCOCO1 | 0.470842869 |
| FOXP2 | CDC37L1 | 0.466545048 |
| FOXP2 | UBQLN2 | 0.459746163 |
| FOXP2 | TNMD | 0.459021118 |
| FOXP2 | LNPEP | 0.453327694 |
| FOXP2 | PIAS3 | 0.44840382 |
| FOXP2 | PTCH1 | 0.435127924 |
| FOXP2 | PLXNA2 | 0.38271875 |
| FOXP2 | OLFM2 | 0.326563415 |
| FOXP2 | SLC7A7 | 0.23261222 |
| FOXP2 | TGFBR3 | 0.223688323 |
| FOXP2 | TCF7L2 | 0.189912869 |
| FOXP2 | STX3 | 0.171014181 |
| FOXP2 | FHL2 | 0.147982075 |
| FOXP2 | KIF3C | 0.056768431 |
| FOXP2 | SPATA2 | 0.038514216 |
| FOXQ1 | CSRP2 | 2.633536826 |
| FOXQ1 | EFNA1 | 2.180058407 |
| FOXQ1 | THSD4 | 2.080250207 |
| FOXQ1 | FAM83C | 1.534208558 |
| FOXQ1 | VAT1 | 1.446739563 |
| FOXQ1 | PDE2A | 1.298678135 |
| FOXQ1 | GEM | 1.022489636 |
| FOXQ1 | SMG1 | 0.818526871 |
| FOXQ1 | COMMD3 | 0.8012963 |
| FOXQ1 | IMP3 | 0.795031966 |
| FOXQ1 | MGST3 | 0.670297164 |
| FOXQ1 | NCK2 | 0.663470502 |
| FOXQ1 | DUSP14 | 0.634050487 |
| FOXQ1 | CXCL14 | 0.565090407 |
| FOXQ1 | MAN1A2 | 0.484097554 |
| FOXS1 | CYGB | 16.05723547 |
| FOXS1 | TSPAN8 | 8.747985886 |
| FOXS1 | ZDHHC9 | 4.01290525 |
| FOXS1 | CSNK1E | 2.677811692 |
| FOXS1 | PCDHB2 | 2.524367787 |
| FOXS1 | LAMA4 | 2.030530626 |
| GABPA | AMOTL1 | 4.546383831 |
| GABPA | MYL6B | 4.339154394 |
| GABPA | DUSP4 | 4.026184376 |
| GABPA | PHIP | 3.232334341 |
| GABPA | KCNMA1 | 3.096499398 |
| GABPA | LMNB1 | 2.913810254 |
| GABPA | ZFAND5 | 2.77310662 |
| GABPA | RBFOX2 | 2.568066313 |
| GABPA | MAPRE2 | 2.396617194 |
| GABPA | MCC | 2.375084736 |
| GABPA | BNIP2 | 2.328269727 |
| GABPA | BCL3 | 2.325813529 |
| GABPA | ZMYM5 | 2.00857179 |
| GABPA | PTK2B | 1.906329722 |
| GABPA | PTPRG | 1.887837696 |
| GABPA | PPP1R1B | 1.875840172 |
| GABPA | MAPK3 | 1.841248284 |
| GABPA | RAVER2 | 1.82533634 |
| GABPA | NAB2 | 1.750167708 |
| GABPA | HAGHL | 1.673026355 |
| GABPA | ZNF263 | 1.672656697 |
| GABPA | ZNF426 | 1.666040272 |
| GABPA | RHOJ | 1.664802353 |
| GABPA | HDAC9 | 1.663100267 |
| GABPA | SCUBE2 | 1.653396977 |
| GABPA | SLTM | 1.617824647 |
| GABPA | MBD1 | 1.546520375 |
| GABPA | BCL2 | 1.527363972 |
| GABPA | TAF5L | 1.517132778 |
| GABPA | ACVR2A | 1.502203642 |
| GABPA | CBLL1 | 1.473263384 |
| GABPA | MLLT3 | 1.448543882 |
| GABPA | MRPS21 | 1.442878375 |
| GABPA | CDK1 | 1.426070438 |
| GABPA | BEX5 | 1.378091687 |
| GABPA | NFKBIL1 | 1.359920275 |
| GABPA | FOXO3 | 1.295518136 |
| GABPA | BHLHE22 | 1.270626259 |
| GABPA | STX7 | 1.178356226 |
| GABPA | INSR | 1.171787029 |
| GABPA | RASA2 | 1.169685607 |
| GABPA | EXTL3 | 1.169669311 |
| GABPA | ACSS3 | 1.104191733 |
| GABPA | FICD | 1.101443417 |
| GABPA | TCF7 | 1.090387547 |
| GABPA | ZMYM2 | 1.075097766 |
| GABPA | UBE2R2 | 1.073537767 |
| GABPA | FOXN3 | 1.043230499 |
| GABPA | RIC8B | 1.039012759 |
| GABPA | EBF2 | 1.009525652 |
| GABPA | NFIX | 1.007094622 |
| GABPA | GABPA | 1 |
| GABPA | NBPF3 | 0.959884203 |
| GABPA | ICA1L | 0.952037833 |
| GABPA | CBX4 | 0.942938618 |
| GABPA | ZNF557 | 0.915873619 |
| GABPA | ABCB9 | 0.904236652 |
| GABPA | PPIL4 | 0.893211881 |
| GABPA | MTUS1 | 0.882765264 |
| GABPA | WWC3 | 0.87168582 |
| GABPA | ZNF600 | 0.862819223 |
| GABPA | PPP4R2 | 0.81917912 |
| GABPA | MED23 | 0.780061624 |
| GABPA | THBD | 0.776557049 |
| GABPA | KIFC1 | 0.771914529 |
| GABPA | PCNP | 0.75169263 |
| GABPA | SOCS5 | 0.745215029 |
| GABPA | ZYX | 0.730548552 |
| GABPA | DDX42 | 0.707996191 |
| GABPA | EIF2AK2 | 0.680984747 |
| GABPA | RER1 | 0.632829166 |
| GABPA | AMD1 | 0.616844694 |
| GABPA | PPM1G | 0.580624947 |
| GABPA | SMARCAD1 | 0.578558427 |
| GABPA | TRMT11 | 0.574931363 |
| GABPA | SLC39A13 | 0.543631622 |
| GABPA | TGS1 | 0.527652573 |
| GABPA | LTBP2 | 0.522667715 |
| GABPA | RWDD2A | 0.511469559 |
| GABPA | LNPEP | 0.504482109 |
| GABPA | PTCH1 | 0.491366771 |
| GABPA | SORT1 | 0.486468245 |
| GABPA | SRSF5 | 0.48272319 |
| GABPA | PANK4 | 0.476292903 |
| GABPA | TNFRSF1A | 0.471634944 |
| GABPA | FOXP2 | 0.457743006 |
| GABPA | MAP4K4 | 0.448991321 |
| GABPA | PLXNA1 | 0.44431706 |
| GABPA | MID1 | 0.444021189 |
| GABPA | ROCK1 | 0.430788099 |
| GABPA | YTHDC1 | 0.415191612 |
| GABPA | SV2B | 0.335159524 |
| GABPA | RXRA | 0.326506474 |
| GABPA | OXNAD1 | 0.297193731 |
| GABPA | TBX3 | 0.288776526 |
| GABPA | NCOR2 | 0.260597766 |
| GABPA | MBNL3 | 0.256023458 |
| GABPA | EAF1 | 0.233089194 |
| GABPA | PRKAG2 | 0.199693644 |
| GABPA | PPARGC1B | 0.171735694 |
| GABPA | DENND3 | 0.152868714 |
| GABPA | ZNF608 | 0.148942032 |
| GABPA | FAM149B1 | 0.145101481 |
| GABPA | SLC12A7 | 0.119636549 |
| GABPA | ZBTB16 | 0.117184348 |
| GABPA | COG2 | 0.090743623 |
| GABPA | USP2 | 0.053424794 |
[truncated: 118,656 more chars]
